# Transcriptional profiling of left ventricle and peripheral blood mononuclear cells in a rat model of postinfarction heart failure

**DOI:** 10.1186/1755-8794-6-49

**Published:** 2013-11-08

**Authors:** Dorota Tulacz, Urszula Mackiewicz, Michal Maczewski, Agata Maciejak, Monika Gora, Beata Burzynska

**Affiliations:** 1 Institute of Biochemistry and Biophysics Polish Academy of Sciences, Pawinskiego 5a, 02-106 Warsaw, Poland; 2 Department of Clinical Physiology, Medical Centre of Postgraduate Education, Marymoncka 99/103, 02-813 Warsaw, Poland

**Keywords:** Left ventricular remodelling, Heart failure, Myocardial infarction, Extracellular matrix, Gene expression profiling, Microarrays, Ceruloplasmin, Tetraspanin 12

## Abstract

**Background:**

Myocardial infarction (MI) often results in left ventricular (LV) remodeling followed by heart failure (HF). It is of great clinical importance to understand the molecular mechanisms that trigger transition from compensated LV injury to HF and to identify relevant diagnostic biomarkers. The aim of this study was to investigate gene expression in the LV and to evaluate their reflection in peripheral blood mononuclear cells (PBMCs).

**Methods:**

MI was induced in rats by ligation of the proximal left coronary artery. Rats with small, moderate, and large MI size were included into the experiment two months after the operation. The development of heart failure was estimated by echocardiography and catheterization. Microarrays were used to compare the LV and PBMCs transcriptomes of control and experimental animals.

**Results:**

Only rats with a large MI developed extensive LV remodeling and heart failure. 840 transcripts were altered in LV of failing hearts, and especially numerous were those associated with the extracellular matrix. In contrast, no significant gene expression changes were seen in LVs of rats with moderate or small MI that had compensated LV injury. We showed that ceruloplasmin was similarly overexpressed in the heart and blood in response to HF, whereas downregulation of tetraspanin 12 was significant only in the PBMCs.

**Conclusion:**

A large size of infarcted area is critical for progression of LV remodeling and HF development, associated with altered gene expression in the heart. Ceruloplasmin and tetraspanin 12 are potential convenient markers in readily obtainable PBMCs.

## Background

Left ventricular (LV) remodelling is crucial in the development of heart failure (HF) and is associated with increased mortality after myocardial infarction (MI). It is characterized by complex structural alterations connected with infarct expansion, scar formation, wall thinning, progressive chamber dilation and hypertrophy [1]. Initially all these changes help maintain normal cardiac function but in time they become maladaptive and can be responsible for systolic dysfunction and progressive decompensation of LV [2]. LV remodelling involves numerous cellular changes, but is essentially connected with dynamic alterations in the extracellular matrix (ECM) [3]. Proper organization and composition of the ECM is necessary for structural (size, shape) and mechanical (strength, contractility) support, and also for communication between different cardiac cells [4]. Numerous transcriptional studies have been performed to investigate alterations in cardiac gene expression related to the progression of heart failure [5-7]. To investigate the mechanisms of heart failure animal models are mostly used (rodents [8-10], dogs [11], swine [12]) as well as human heart tissues [13,14] from patients with severe failing hearts classified to transplantation. Studies on human HF usually concentrate on the end-stage of heart failure and drug-treated myocardium. Therefore, they miss factors that uniquely mark the transition of LV hypertrophy to heart failure, making animal heart failure models irreplaceable.

Peripheral blood mononuclear cells (PMBCs), being easily obtainable, could be used as a proxy to study changes associated with heart diseases. Blood permeates the heart with infiltration of leukocytes into the muscle tissue under the certain conditions. Thus, PBMCs contribute to the inflammatory response in the infarcted myocardium and are also considered a source of factors crucial in matrix remodelling, such as matrix metalloproteinase 9 [15].

The aim of the present study was to investigate gene expression in the left ventricle in a well validated model of post-infarcted HF and to evaluate their reflection in PBMCs. We decided to use the rat model since it is the most commonly used animal model of myocardial infarction and heart failure and had convincingly been shown to mimick the mechanisms of human HF.

## Methods

### Rat myocardial infarction model and samples processing

The study conformed to the *Guide for the Care and Use of Laboratory Animals* by the US National Institutes of Health (NIH publication No. 85–23, revised 1996) and was approved by the local Ethics Committee.

Myocardial infarction (MI) was induced in 40 male Wistar rats (275–300 g) by ligation of the proximal left coronary artery, as described previously [16]. In brief, rats were anaesthetised with ketamine HCl and xylazine (100 mg/5 mg/kg body weight), left thoracotomy was performed and a silk suture was tightly tied around the proximal left coronary artery, approximately 2 mm from its origin. The sham-operated group (control group, n = 6) was subjected to the same protocol, except that the suture was not tied.

Two months after the operation the rats were anaesthetised with ketamine HCl and xylazine (75 mg/3.5 mg/kg body weight) and echocardiography was performed to evaluate the LV function and dimensions and to estimate the infarct size. Later a micromanometer-tipped catheter (Millar Instruments) was advanced through the right carotid artery into the LV for recording of LV pressures. Finally, the heart was dissected and blood samples were obtained. Whole blood was collected via cardiac puncture under anaesthesia and transferred to EDTA-K2 collection tubes (Medlab Products, Warsaw, Poland). PBMCs (peripheral blood mononuclear cells) were isolated by centrifugation through HISTOPAQUE® 1083 (Sigma-Aldrich, St Louis, MO, USA) according to the manufacturer's instructions and resuspended in RNA*later®* Solution (Ambion Inc., Austin, TX, USA). LV from rats with induced MI was divided into a well visible fibrotic scar tissue and apparently healthy myocardial tissue. For further examinations, whole LV from sham-operated rats and the morphologically unaffected part of LV from MI rats were placed in RNA*later®* Solution and stored at -80°C until RNA extraction.

For determination of infarct size regional LV wall motion abnormalities were quantitated as described previously [16]. The contractility of twelve wall segments visualized in the midpapillary short-axis view and eleven segments visualized in the long-axis view was graded as 1 (normal) or 0 (abnormal) and the total wall motion index (WMI) was calculated. Normal hearts had WMI = 23. Our previous results [16] revealed that WMI closely correlated with infarct size, thus post-MI rats were divided into three groups: large MI (L-MI, ≥ 40% of LV area, n = 5), moderate MI (M-MI, 20-39% of LV area, n = 6) and small MI (S-MI, <20% of LV area, n = 6).

All results are given as means ± SEM. The statistical significance was determined by one-way ANOVA followed by a Dunnett test in case of significance, with the Statistica software package (version 6.0, StatSoft, Poland). The difference was considered significant at *P <* 0.05.

### RNA isolation

Total RNA was isolated from PBMCs and heart samples using MagNA Pure Compact System (Roche Diagnostics GmbH, Germany) according to the manufacturer's recommendations. Heart tissues were homogenized using MagNA Lyser Instrument (Roche Diagnostics GmbH, Germany). The amount of isolated total RNA was quantified by UV absorption (Nanodrop, LabTech International, UK). The quality of these samples was determined using an Agilent 2100 Bioanalizer^©^ and RNA 6000 Nano Kit (Agilent, Santa Clara, CA, USA). For all RNA samples the RIN (RNA integrity number) value was above eight.

### cDNA microarrays

GeneChip® Rat Gene 1.0 ST Arrays (Affymetrix, Santa Clara, CA, USA) were used. RNA from LV and PBMCs samples obtained from L-MI and sham-operated rats were processed according to standard Affymetrix manufacturer's protocols. First, the samples were reverse transcribed and amplified, then fragmented and biotinylated. Hybridization to the GeneChips® was conducted for 16 h at 45°C. After hybridization the microarrays were washed and stained on a fluidics station and scanned in an Affymetrix GCS 3000 GeneArray Scanner.

### Data analysis of microarrays

After a robust multiarray average (RMA) normalization of microarray data, differences in gene expression levels were calculated by comparing the group with myocardial infarction with the sham-operated group. Lists of differentially expressed genes, principal component analysis (PCA) and hierarchical clustering were generated using the Partek Genomics Suite software (Partek Inc, St. Louis, MO, USA). The fold change (FC) of gene expression ratios ≥1.3 and *P* ≤ 0.05 corrected by false discovery rate (FDR) were set as significant criteria for heart tissue. For PBMCs the significance criteria were set at FC ≥ 1.2 and *P* ≤ 0.05, since alterations in RNA levels are usually lower in PBMCs than in other tissues [17]. Results were additionally annotated using Gene Annotator (http://rgd.mcw.edu/rgdweb/ga/start.jsp) and BLAST: Basic Local Alignment Search Tool (http://blast.ncbi.nlm.nih.gov/). Gene ontology analysis was done using the AmiGO's Term Enrichment tool (version 1.8, http://amigo.geneontology.org) and the DAVID Gene Functional Classification Tool online (http://david.abcc.ncifcrf.gov). The Ingenuity Pathways Analysis software (IPA, Ingenuity® Systems, http://www.ingenuity.com) was used to generate statistically relevant molecular interaction networks associated with the lists of differentially expressed genes. The raw data reported in this paper have been deposited in the Gene Expression Omnibus (GEO) database with the accession number GSE47495.

### Quantitative real-time RT-qPCR

The real-time reverse transcription-polymerase chain reaction (RT-qPCR) was used to validate the microarray data. Each total RNA sample (400 ng) was reverse transcribed using QuantiTect Reverse Transcription (Qiagen, Germany) according to the manufacturer's recommendations. Primer sets for selected genes were designed using Clone Manager Suite software; their sequences and reaction conditions are available in additional files (Additional file 1). Specificity of the amplified product was demonstrated by melting curve analysis and agarose gel electrophoresis (data not shown). Each sample was run in triplicate in 96-well plates using LightCycler®480 and LightCycler®480 FastStart SYBR Green I Master (Roche Diagnostics GmbH, Germany). Quantification cycles (Cq) were calculated using the fit point method (LightCycler®480 Software, Version 1.5 provided by Roche). The fold change in gene expression levels, corrected by efficiency, was analyzed using REST-MCS©-version 2 software [18]. The expression data were normalized to the reference glyceraldehyde-3-phosphate dehydrogenase (*Gapdh*) and hypoxanthine-guanine phosphoribosyltransferase (*Hprt1*) genes, which in a GeNorm algorithm-based selection [19] were the most stable among the four that we tested (M-value; the highest M-value means the least stable transcript): *Hprt* and *Gapdh -*0.464, TATA box binding protein (*Tbp*) – 0.615, polymerase (RNA) II (DNA directed) polypeptide A, transcript variant 2 (*Polr2a*) – 0.692. All experiments (sample collection, preparation and storage, primer design, qPCR normalization) were performed according to the MIQE guidelines [20].

## Results

### Physiological characteristic of rats

Two months after MI induction rats with large MI (L-MI, mean infarct size 49.8%) had extensive LV dilation, ejection fraction (EF) reduction and a highly elevated left ventricular end-diastolic pressure (LVEDP), a recognized marker of heart failure in the rat MI model (Table 1). On the other hand the rats with small (S-MI) or moderate myocardial infarction (M-MI) (mean infarct size 10.5% and 25.8%, respectively) had only mild LV dilation and EF reduction, but normal LVEDP.

**Table 1:** Body weight and echocardiographic parameters in post-myocardial infarction and sham-operated rats 2 months after surgery

	**Sham, n = 6**	**Infarct**		
		**Small, n=6**	**Moderate, n=6**	**Large, n=5**
BW [g]	419 ± 14	411 ± 8	424 ± 22	377 ± 4^*^
Infarct size [%]	0	10.5 ± 1.6^***^	25.8 ± 1.6^***^	49.8 ± 1.5^***^
LVEDD [mm^2^]	58.3 ± 1.0	63.8 ± 2.1	67.5 ± 1.1^***^	99 ± 6.3^***^
LVESD [mm^2^]	26.5 ± 0.7	35 ± 1.5^**^	39.5 ± 0.8^***^	76 ± 6.0^***^
EF [%]	54.5 ± 0.9	45.2 ± 0.7^***^	41.5 ± 0.5^***^	23.2 ± 2.8^***^
LVEDP [mm Hg]	6.0 ± 0.3	5.8 ± 0.3	6.8 ± 0.3	21.1 ± 0.5^***^

BW - body weight, LVEDD - left ventricular end-diastolic diameter, LVESD - left ventricular end-systolic diameter, EF- ejection fraction, LVEDP - left ventricular end-diastolic pressure.

Data are presented as mean ± SEM. *P < 0.05, **P < 0.01, ***P < 0.001.

This indicates that while the rats with the large MI developed extensive LV remodelling and heart failure, the rats with small or moderate infarction developed only mild LV remodelling and a condition that could be called compensated LV injury.

### Microarrays results from heart tissues

**Figure 1:**
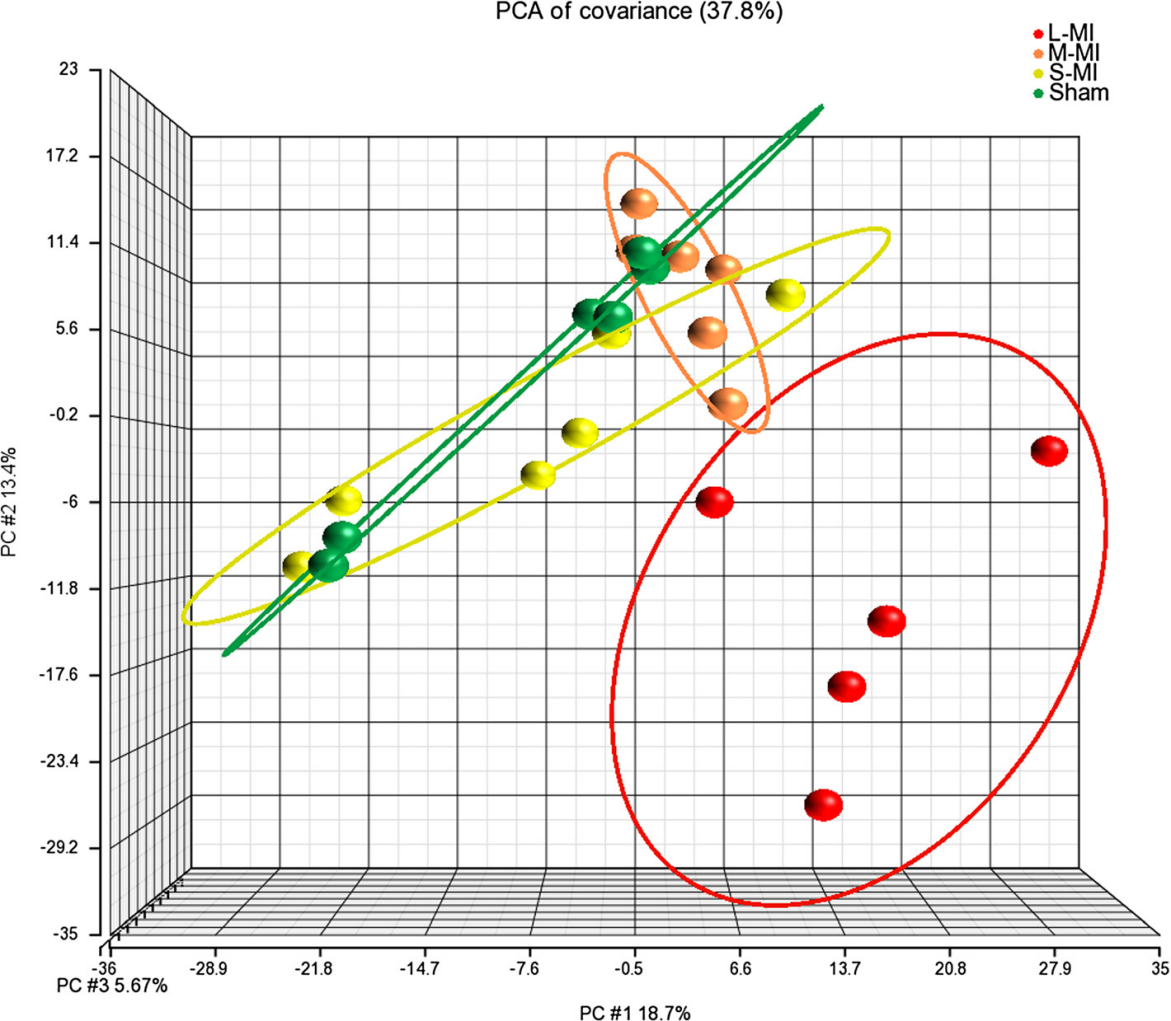
**Principal component analysis of left ventricular gene expression profiles.** PCA plot shows the first three principal components of microarray data in respect to their correlation. Sham - sham-operated; S-MI - small-size infarction; M-MI - moderate-size infarction; L-MI - large-size infarction.

The microarray data after normalization were subjected to PCA analysis to visualize gene expression differences between control and treated animals. The PCA plot (Figure 1) demonstrates a distinct separation between sham-operated rats and rats with L-MI in contrast to animals with S-MI and M-MI, which clustered together with the sham group.

Genes expressed differentially between groups were determined using the cutoff criteria described in Materials and Methods. The expression of 840 transcripts was altered in L-MI rats 2 months after the operation. Consistent with the results of the PCA analysis, no gene expression differences were identified between rats with S-MI or M-MI and the control group. From the total 840 transcripts altered in the L-MI group, 814 (553 - upregulated, 261 - downregulated) were supported with gene annotations provided by RefSeq, GeneBank (http://www.ncbi.nlm.nih.gov) or Ensembl (http://www.ensembl.org). Their full list is available in additional files (Additional file 2).

**Figure 2:**
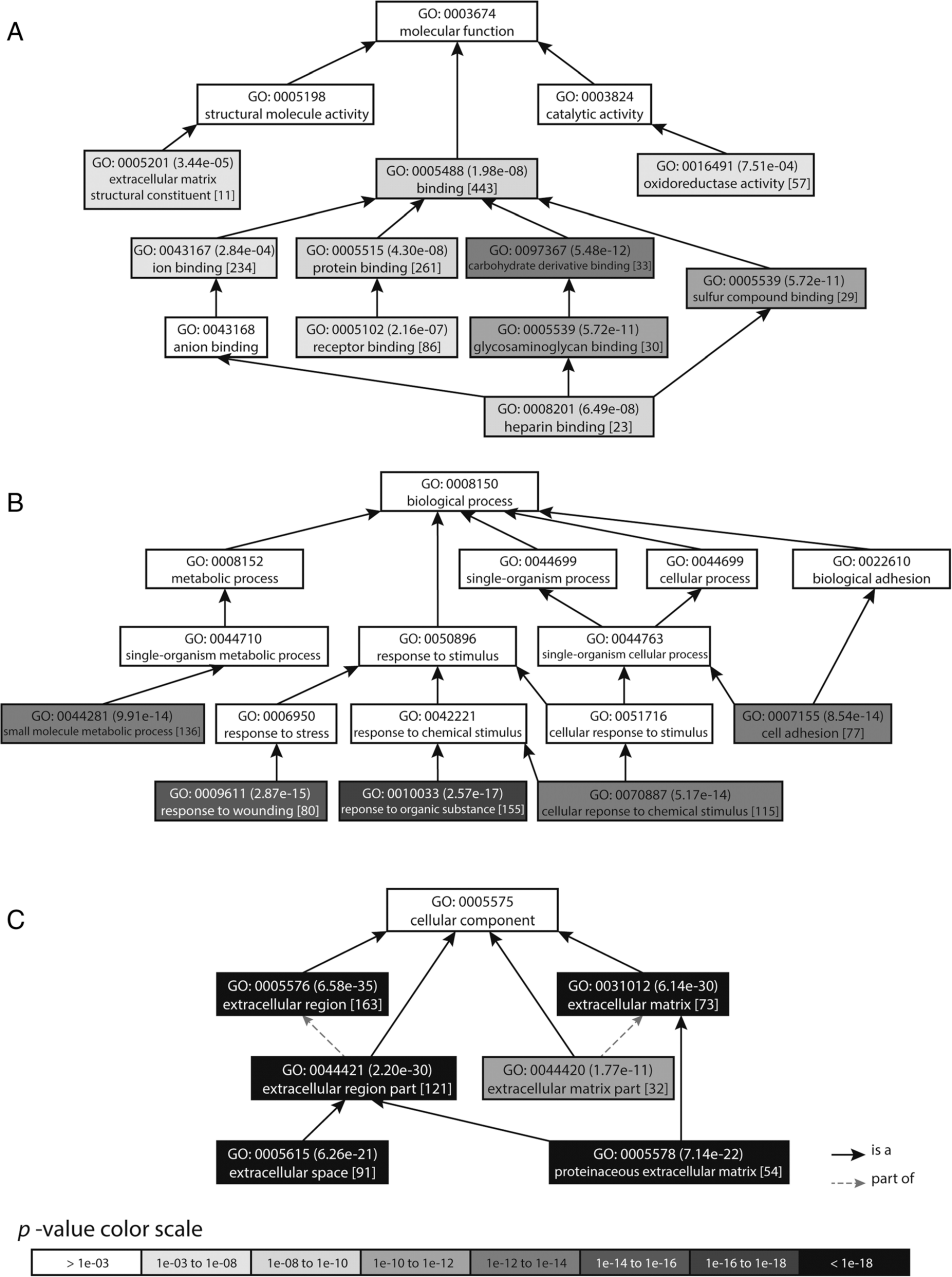
**Enriched GO categories among differentially expressed genes in large-size infarction compared to sham-operated rats.** Expression level were determined in left ventricle. Graphs show top results for the biological process **(A)**, molecular function **(B)** and cellular component **(C)**. In round brackets the *p*-value obtained from the AmiGO is shown, in square brackets the number of differentially expressed genes related to a particular GO term.

The genes identified as significantly altered in the L-MI group were further subjected to a gene ontology analysis (AmiGO) to find significant shared Gene Ontology terms. Figure 2 presents only the GO terms with the most significant *p*-values, whereas in additional files we present complete results (Additional file 3). The analysis revealed that the molecular function of these genes is essentially connected to GO:0005488 binding and particularly to GO:0097367 carbohydrate derivative binding, GO:0005539 glycosaminoglycan binding, GO:1901681 sulfur compound binding, GO:0008201 heparin binding, GO:0005102 receptor binding and GO:0005515 protein binding. The biological processes are mainly associated with response to stimuli: GO:0010033 response to organic substance, GO:0009611 response to wounding and GO:0070887 cellular response to chemical stimulus. Cell adhesion GO:0007155 and small molecule metabolic process GO:0044281 were also at the top of significantly altered biological processes. Cellular component classification showed that most of the differently expressed genes were involved in GO:0005576 extracellular region (GO:0044421 extracellular region part, GO:0005615 extracellular space) and GO:0031012 extracellular matrix (GO:0005578 proteinaceous extracellular matrix, GO:0044420 extracellular matrix part).

**Figure 3:**
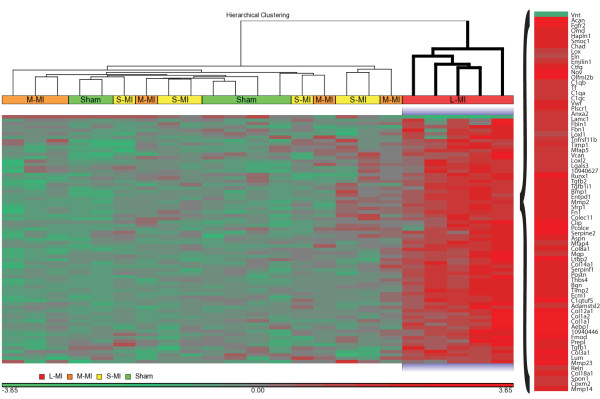
**Heat map representing hierarchical clustering of 73 differentially expressed genes related to extracellular matrix (GO:0031012).** Each column corresponds to a single microarray whereas each row represents expression profile of a single gene. Red stand for the positive values in the gene expression and green for the negative ones. Sham - sham-operated; S-MI - small-size infarction; M-MI - moderate-size infarction; L-MI - large-size infarction.

Extracellular matrix remodelling is the critical event in pathology of HF. For that reason, a focused hierarchical clustering analysis was done for transcripts annotated as GO:0031012 extracellular matrix (Additional file 4) to show their expression profiles in LV samples from individual rats (Figure 3). This analysis revealed a clearly distinct gene expression profile of the selected genes in rats with L-MI that clustered separately from the other groups. Interestingly, all these genes were upregulated in the L-MI group, with one exception - vitronectin (*Vnt*), although they are involved in both ECM deposition and degradation.

### Microarray results from PBMCs

**Figure 4:**
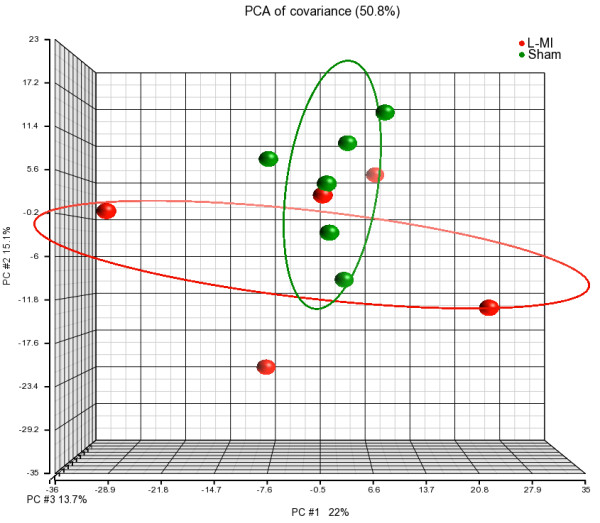
**Principal component analysis of PBMCs gene expression profiles.** PCA plot shows the first three principal components of microarray data in respect to their correlation. Sham - sham-operated; L-MI - large-size infarction.

PBMCs are considered to be an ideal surrogate tissue to reflect changes occurring in major human organs [14]. Therefore, a further microarray analysis was performed to evaluate if and how the progressive heart failure in rats with L-MI affected the gene expression patterns in PBMCs. Global transcriptomic profiles analysed by PCA demonstrated a separation between the sham-operated rats and rats with L-MI that was less clear-cut than that found for the heart tissue (Figure 4). A list of differentially expressed genes was determined with the cutoff criteria described in Materials and Methods. The expression of 72 (38 - upregulated, 34 - downregulated) well-annotated transcripts was altered in PBMCs in the L-MI rats 2 months after the operation in comparison with sham-operated rats (Additional file 5). The AmiGO analysis showed no significantly enriched GO terms, while the DAVID Functional Annotation Tool identified several categories. The molecular function of those genes is mainly connected to GO:0005488 binding, especially GO:0005529 sugar and GO:0005537 mannose binding. Biological processes are associated with response to stimuli: GO:0014070 response to organic cyclic substance and GO:0009607 response to biotic stimulus (GO:0009617 response to bacterium stimuli, GO:0051007 response to other organism). Cellular component classification showed that most of those genes were connected with GO:0016020 membrane. Complete results are shown in Additional file 6.

A comparison of the list of differentially expressed genes in LV tissue and PBMCs from rats with L-MI revealed five transcripts (*Klra7 -* killer cell lectin-like receptor, subfamily A, member 7; *Clr7 -* C-type lectin-related protein 7; *Cp -* ceruloplasmin; *Ptgs2 -* prostaglandin-endoperoxide synthase 2 and *Tspan12 -* tetraspanin 12) similarly altered in both tissues (Additional file 7).

### Validation of microarrays with RT-qPCR

Reverse transcription-qPCR was used to validate the microarray data. Several genes important for heart functioning were assayed and the results are demonstrated in Tables 2 and 3.

**Table 2:** Comparison of results from microarrays and RT-qPCR

	**Large-MI**						**Moderate-MI**			**Small-MI**		
	**Results from RT-qPCR**			**Results from microarrays**			**Results from RT-qPCR**			**Results from RT-qPCR**		
**Gene** ** symbol**	**Fold change**	***P*-value**	**Significance**	**Fold change**	***P*-value**	**Significance**	**Fold change**	***P*-value**	**Significance**	**Fold change**	***P*-value**	**Significance**
Biomarkers in heart failure												
***End1***	**1.9**	0.043	**	**1.3**	0.005	**	**-1.1**	0.412	ns	**-1.3**	0.147	ns
***Nppa***	**19.2**	0.001	***	**3.7**	1.3e-5	***	**1.1**	0.884	ns	**1.2**	0.694	ns
***Nppb***	**2.2**	0.001	***	**1.4**	0.14	ns	**-1.1**	0.849	ns	**-1.5**	0.496	ns
Others related to cardiac hypertrophy and left ventricular remodelling												
***Corin***	**2.6**	0.020	*	**1.4**	3e-5	***	**1**	0.788	ns	**1**	0.934	ns
***Hcn4***	**3.6**	0.013	*	**2.1**	0.001	***	**1**	0.951	ns	**-1.4**	0.343	ns
***Pparg***	**-1.7**	0.017	**	**-1.4**	1.4e-4	***	**1.1**	0.643	ns	**1**	0.865	ns
***Tgfβ1***	**1.4**	0.071	ns	**1.3**	1.6e-5	***	**1.1**	0.613	ns	**1.1**	0.712	ns
***Tgfβ2***	**3.1**	0.010	**	**2**	7.8e-5	***	**-1.1**	0.762	ns	**1**	0.874	ns
ECM – Extracellular matrix components												
ECM structural components												
***Col1a1***	**3.9**	0.003	**	**2.3**	6.1e-6	***	**1**	0.767	ns	**1**	0.852	ns
***Col1a2***	**2.3**	0.011	**	**1.6**	5e-5	***	**-1.1**	0.685	ns	**1**	0.852	ns
***Col3a1***	**2.1**	0.017	**	**1.5**	1.3e-5	***	**-1.1**	0.579	ns	**1.1**	0.619	ns
***Col8a1***	**5.1**	0.001	***	**2.1**	3.1e-8	***	**1**	0.880	ns	**1**	0.390	ns
***Col12a1***	**5.1**	0.001	***	**2.9**	3.4e-8	***	**1**	0.912	ns	**-1.1**	0.215	ns
***Col14a1***	**2.4**	0.001	***	**2**	1.0e-7	***	**1.1**	0.628	ns	**1.1**	0.891	ns
***Col18a1***	**2.2**	0.001	***	**1.3**	7.6e-5	***	**-1.1**	0.668	ns	**-1.1**	0.310	ns
***Eln***	**2.2**	0.001	***	**1.4**	1.8e-5	***	**1.2**	0.304	ns	**1.1**	0.689	ns
***Fn1***	**3.8**	0.005	**	**2.9**	6.2e-8	***	**1.2**	0.384	ns	**1.1**	0522	ns
***Lamc1***	**1.3**	0.014	**	**1.3**	5.1e-7	***	**1.1**	0.451	ns	**1**	0.773	ns
***Prg4***	**2.1**	0.004	**	**1.7**	2.4e-5	***	**1**	0.907	ns	**-1.1**	0.430	ns
***Vtn***	**-1.4**	0.015	*	**-1.4**	2.3e-6	***	**1**	0.874	ns	**-1.1**	0.167	ns
Matricellular proteins												
***Mmp2***	**1.6**	0.001	***	**1.4**	7.5e-7	***	**1**	0.855	ns	**1.1**	0.343	ns
***Mmp9***	**2.7**	0.351	ns ^†^	**1.73**	0.037	*	**1.2**	0.660	ns	**1**	0.871	ns
***Mmp13***	**62.2**	0.001	*** ^†^	**2**	0.021	*	**-1.2**	0.358	ns	**1**	0.862	ns
***Mmp14***	**2.3**	0.019	**	**1.3**	0.002	**	**1.1**	0.529	ns	**-1.1**	0.465	ns
***Mmp16***	**2.9**	0.020	**	**1.3**	0.006	***	**-1.4**	0.204	ns	**-1.3**	0.334	ns
***Mmp23***	**1.8**	0.027	**	**1.3**	1.0e-4	***	**-1.1**	0.572	ns	**-1.2**	0.438	ns
***Postn***	**20.8**	0.001	***	**8**	1.2e-9	***	**1**	0.988	ns	**1.1**	0.738	ns
***Spp1***	**52.3**	0.003	**	**4.1**	2.3e-5	***	**2.7**	0.157	ns	**2.3**	0.212	ns
***Thbs4***	**19.4**	0.003	**	**6.1**	8e-9	***	**-1.7**	0.272	ns	**-1.3**	0.348	ns
***Timp1***	**3**	0.008	**	**2.4**	2.8e-7	***	**1.2**	0.287	ns	**1.4**	0.041	*
***Timp2***	**1.7**	0.037	**	**1.5**	4.6e-7	***	**-1.1**	0.666	ns	**1**	0.936	ns
***Timp3***	**1.9**	0.043	*	**1.2**	7.8e-5	***	**-1.4**	0.288	ns	**-1.3**	0.321	ns
***Timp4***	**-1.5**	0.151	ns	**-1.4**	0.006	**	**-1.3**	0.307	ns	**-1.1**	0.716	ns

Expression level of selected genes was measured 2 months after operation in left ventricle tissues of all infarcted rats and compared to sham-operated group (statistical significance: *P < 0.05, **P < 0.01, ***P < 0.001, ns = non significant, † expression level in sham-operated rats close to the limit of detection). Results were normalized to *Gapdh* and *Hprt*.

**Table 3:** Comparison of results from microarrays and RT-qPCR of genes altered similarly in LVs and PBMCs

		**Large-MI vs Sham in PBMCs**				**Large-MI vs Sham in LVs**			
		**Results from RT-qPCR**		**Results from microarrays**		**Results from RT-qPCR**		**Results from microarrays**	
**Gene symbol**	**GenBank ID**	**Fold change**	***P*-value**	**Fold change**	***P*-value**	**Fold change**	***P*-value**	**Fold change**	***P*-value**
**Clr7**	**EU128749.1**	**1.4**	0.129^ns^	**1.6**	0.014*	**1.5**	0.046*	**1.7**	2.4e-6***
**Cp**	**NM_012532**	**2.3**	0.039*	**1.2**	0.019*	**3.3**	0.001**	**2.3**	1.9e-9***
**Klra7**	**XM_578407.4**	**1.6**	0.112^ns^	**1.4**	0.048*	**2.3**	0.001**	**1.2**	3.3e-4***
**Ptgs2**	**NM_017232**	**2.4**	0.073^near^	**1.3**	0.037*	**3.5**	0.001**	**2.2**	9.9e-6***
**Tspan12**	**NM_001015026**	**-3.22**	0.019*	**-3.0**	0.011*	**1.1**	0.734^ns^	**-1.4**	8.0e-6***

Statistical significance: *P < 0.05, **P < 0.01, ***P < 0.001, ns = non significant, near = near significance. Results were normalized to *Gapdh* and *Hprt*.

First, to verify the main conclusion drawn from the microarray results for LV samples, the expression levels of genes coding for cardiac neurohormones (*Nppa -* natriuretic peptide A precursor*, Nppb -* natriuretic peptide B precursor*, Edn1 -* endothelin 1) and the enzyme responsible for converting pro-ANP and pro-BNP to mature ANP and BNP (*Corin -* corin, a serine peptidase) were determined. We confirmed that those genes were upregulated in the L-MI group in contrast to the S-MI and M-MI groups, which indicated that only in the L-MI group the myocardial infarction progressed to heart failure.

We also tested transcript levels of other genes known to be associated with cardiac hypertrophy and left ventricular remodelling. Expression of these transcripts was altered significantly in our model only in rats with a large MI.

Further, changes in the expression level of genes connected with cardiac extracellular matrix were confirmed. In addition to the genes whose upregulation was indicated by microarray data, additional matrix metalloproteinase and tissue inhibitor of metalloproteinase genes (*Mmp9, Mmp13, Mmp16, Mmp23, Timp3, Timp4*) were checked. The levels of the *Mmp9* and *Mmp13* transcripts were close to the detection limit of the platform (Cq values around cycle 35) in sham-operated, S-MI, and M-MI rats, but were substantially higher (cycle 32 and 29 respectively) in the L-MI group, indicating their up-regulation after large infarction.

Overall, the RT-qPCR results were qualitatively consistent with the results of the microarray analysis. However, the RT-qPCR analysis tended to give higher up-regulation levels than those calculated from the microarray data. The highest change was found for periostin (*Postn*), osteopontin (*Spp1*) and thrombospondin 4 (*Thbs4*) (20.8, 52.3, and 19.4 -fold, respectively).

In addition, RT-qPCR was used to quantitate the five transcripts found earlier to be differentially expressed following HF in both heart and blood. The results are shown in Table 3 for L-MI and in Additional file 8 for all examined MI groups. Overexpression of ceruloplasmin (*Cp*) was significant (*P <* 0.05) in both tissues, whereas significant downregulation of tetraspanin 12 (*Tspan12*) was confirmed in PBMCs but not in LV.

## Discussion

The transition from LV injury to heart failure is poorly understood. Only a small percentage of patients develop heart failure immediately after MI, but with time many more eventually progress to heart failure. What triggers this process remains a matter of speculation. Therefore it could be of great clinical relevance to understand the differences between stable compensated LV injury and heart failure at the gene expression level and to identify potential triggers of the decompensation of compensated LV injury.

The rat MI model has been used extensively to study the pathophysiology of cardiovascular disease owing to its good reproducibility and similarity to the course of the human disease [21]. It has previously been shown in this model that large MI results in progressive LV remodelling involving LV dilatation, hypertrophy and systolic failure as well as progressive increase of serum BNP (natriuretic peptide B) concentration, while smaller infarctions result in limited LV injury that is not progressive [16]. Furthermore, measurements of LV wall stress indicate that while in large MI the wall stress progressively increases after the infarction, in smaller infarcts its progression is stopped fairly early after the infarction [22].

In the present study, we analyzed echocardiographic parameters and gene expression changes in rats with a wide range of myocardial infarct size two months after MI induction. Both echocardiographic parameters and expression data of well-known HF biomarkers (*Nppa -* natriuretic peptide A precursor*, Nppb -* natriuretic peptide B precursor*, Edn1 -* endothelin 1 [23,24]) supported the finding that only animals with large MI developed extensive LV remodelling and heart failure, while intermediate or small MI resulted in compensated LV injury. We confirmed a significant up-regulation of *Corin* gene expression in rats with large MI that was shown earlier for the LV myocardium of a rat model of heart failure induced by ligation of the left coronary artery [25]. Interestingly, *Corin* gene expression was decreased in the atrium in HF rats [26]. Recently, levels of circulating plasma corin were found to be diminished in patients with heart failure, but not in patients with acute MI [27,28]. These findings indicate that distinct expression profiles of *Corin* in ventricular and atrial tissues and reduced levels of soluble corin protein in the plasma may be important in the pathogenesis of HF. In addition, we noted in the failing heart a high increase of the expression levels of trombospondin 4 (*Thbs4*), osteopontin (*Ssp1*) and periostin (*Postn*), in support of previously published results indicating that these proteins could have high therapeutic and diagnostic potential in HF [6,29].

Analysis of transcriptome profiles revealed markedly altered gene expression in the left ventricular tissues in rats with large MI. In contrast, two months after MI induction rats with moderate or small MI did not differ significantly from the sham operated ones in this regard. The transcripts altered in the L-MI group were related to diverse GO categories, of which the most striking was upregulation of extracellular matrix genes. It is well established that an imbalance between ECM components and disturbances in collagen metabolism are crucial in the progression of heart failure [30,31]. Therefore, detailed understanding of the alterations of key matrix proteins is essential in deciphering the mechanisms of LV remodelling after MI.

Matrix metalloproteinases (MMPs), also known as matrixins, are proteinases crucial in both normal tissue remodelling (development, wound healing) and in pathological conditions (arthritis, scar formation after MI, LV remodelling). There is ample evidence that MMPs modulate myocardial remodelling underlying heart failure progression [32,33]. From our results *Mmp2*, *Mmp14*, *Mmp16* and *Mmp23* appeared to be constitutively expressed in the heart and overexpresed in volume overload conditions, whereas *Mmp9* and *Mmp13* transcripts were abundant only in the failing heart. To the best of our knowledge, this is the first report of an elevated expression of *Mmp16* ("membrane type", synonym MT3-MMP) connected with heart failure. The interstitial tissue homeostasis is determined by an interplay between MMPs and their tissue post-translational inhibitors (TIMP). TIMPs not only bind the active MMP domain, but can also interact with the pro-MMP domain which can facilitate MMP activity [33]. MMP/TIMP profiles can be informative in the context of HF. Our study showed significant overexpression of *Timp1*, -*2* and -*3,* similar to the end-stage human heart failure [31]. An upregulation of *Timp1* was also found in studies on the transition from hypertrophy to HF in rats [34], and an increased serum level of TIMP1 protein was related with HF progression [35] and mortality risk [36].

Peripheral blood mononuclear cells (PBMCs) are more easily accessible than a tissue biopsy. Numerous studies have shown that circulating blood cells are involved in the pathogenesis of many diseases and may serve as biomarkers of pathological changes occurring in other tissues [17,37]. PBMCs also contribute to LV remodelling by expressing key proteins, such as matrix metalloproteinase 9 [15]. In the present study we did not observe significant changes in the expression of MMPs or TIMPs in PBMCs (data not shown), but found other gene alterations of interest.

Our analysis indicated five HF-affected genes in the both examined tissues, but only one - ceruloplasmin (*Cp*) - showed statistically significant changes in expression (Table 3). Ceruloplasmin is a plasma metalloprotein synthesized in hepatocytes and in activated monocytes/macrophages and secreted into the serum, with a copper ion incorporated during biosynthesis. It acts as an antioxidant through its ferroxidase activity and also regulates nitric oxide homeostasis in the blood plasma by converting NO to NO_2_, thus an elevated level of CP may lead to decreased NO bioavailability and endovascular dysfunction [38]. Ceruloplasmin level increases in various acute inflammatory conditions, including acute graft-versus-host-disease (aGvHD) after allogeneic hematopoietic stem cell transplantation [39]*,* cancer [40], and cardiovascular disease [41]*.* Recent studies indicate serum CP as an independent cardiac risk factor [42] and show its association with heart failure [43,44]. Our results strongly support those findings, since large infarction in rats raised the level of *Cp* mRNA in the heart and PBMCs. We showed that both PBMCs and failing myocardium could be a major source of ceruloplasmin that is subsequently found in the serum of patients with heart failure. Accordingly, we propose that CP is important in the progression of heart failure due to its property of an acute phase protein, antioxidant function and regulation of nitric oxide homeostasis.

**Figure 5:**
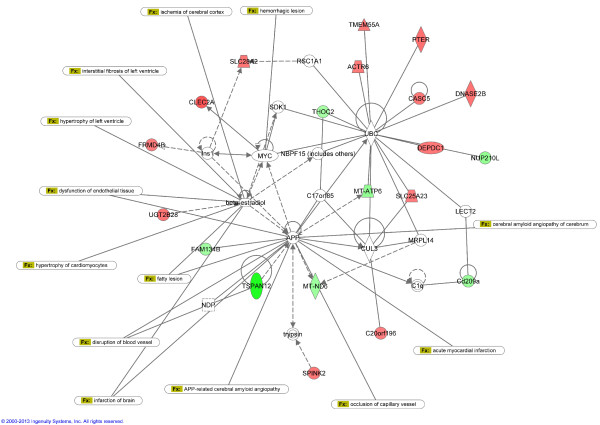
**Highest scoring interaction network generated by Ingenuity Pathway Analysis for genes differentially expressed in PBMCs.** The top network classified as “Cardiovascular System Development and Function, Cellular Function and Maintenance, Cellular Growth and Proliferation” is overlaid for cardiovascular diseases. Genes or gene products are represented as nodes, and the biological relationship between two nodes is represented as a line. Continuous lines indicate direct interactions, while dashed lines indicate indirect relationships. Upregulated and downregulated genes are shown in red and green shading respectively, with colour intensity related to the fold change in expression. White symbols indicate molecules absent in the analysed dataset but related with the dataset genes.

Another interesting gene responding to HF was tetraspanin 12 (*Tspan12*). In contrast to *Cp*, it was downregulated substantially in the PBMCs but showed no response in the heart tissue. Members of the tetraspanin superfamily regulate key cellular processes such as adhesion, migration, and fusion. Tetraspanins play particularly important roles in cancer, by modulating tumor cell growth. Tetraspanins are expressed in vascular and haematopoietic cells and are involved in both physiological and pathological processes related to angiogenesis, vascular injury, thrombosis, and haemostasis. Previous studies suggest that tetraspanins directly regulate the development and functioning of the vascular system and the pathogenesis of vascular diseases [45]. In addition, mutations in the *Tspan12* gene are associated with familial exudative vitreoretinopathy, a human disease caused by failure of peripheral retinal vascularization. Our results for the first time linked directly *Tspan12* downregulation with heart failure development. To better understand the biological relevance of this finding we performed an Ingenuity Pathway Analysis of the molecular interaction networks for the genes differentially expressed in PBMCs. The major functional network with the highest score (IPA score = 47) and with 20 up- or downregulated genes, including *Tspan 12*, was associated with Cardiovascular System Development and Function, Cellular Function and Maintenance, Cellular Growth and Proliferation (Figure 5). This network analysis indicates that altered expression of tetraspanin 12 and other identified molecules may result in a dysfunction of the cardiovascular system by disturbance of their direct and indirect functional interactions. Moreover, tetraspanin 12 was found recently to regulate the activity of membrane metalloproteinases, MT1-MMP/MMP14 [46] and ADAM10 (disintegrin and metalloproteinase domain-containing protein 10) [47]. Affected functions of MT1-MMP/MMP14 influence fibronectin proteolysis and extracellular matrix degradation. ADAM10 is responsible for the proteolytic release of the ectodomains of numerous protein substrates, including specific ligands of EGFR (epidermal growth factor receptor) regulating the subsequent EGFR-dependent signal transduction pathways involved in cardiac hypertrophy [48]. Interestingly, ADAM10 mediates also cleavage of corin that may be important in regulating corin activity and hence ANP processing [49].

On the other hand, it is well known that heart remodeling after myocardial infarction, especially after a large one, is accompanied by extensive neurohumoral activation. An increased plasma norepinephrine concentration due to a marked increase in the sympathetic nerve activity is a common finding in experimental heart failure models as well as in humans. Moreover, the increased wall stress after myocardial infarction and increased plasma norepinephrine level result in the activation of the renin-angiotensin-aldosteron system (RAAS) followed by an increase of the renin and angiotensin II plasma levels. Additionally, increased levels of vasopressin and endothelin have been found in heart failure. [50]. It is possible that these neurohormones circulating in the plasma could influence the expression of diverse genes in PMBCs through activation of the receptors present on the surface of these cells. Besides, PBMCs are mononuclear cells recruited into the infarcted myocardium [15] and the invading monocytes differentiate into macrophages also during tissue repair after myocardial infarction [51]. We therefore hypothesize that the altered expression of *Tspan12* in PBMCs may influence the pathological processes occurring in the failing heart, especially that tetraspanins are present not only on the plasma membrane but also in exosomes, which are often released by cells as messenger vehicles for intercellular communication [52].

## Conclusions

We showed that a large size of infarcted area is critical for progression of LV remodelling and HF development, associated with altered gene expression in the heart. In contrast, compensated LV injury does not lead to significant changes of the transcriptome as evaluated two months after the injury. Transcriptional profiling of failing myocardium and peripheral blood mononuclear cells simultaneously is a useful approach for robust markers of heart failure. We identified genes (*Cp*, *Tspan12*) whose expression is altered in the readily obtainable blood cells in response to progressive heart failure. To our knowledge, this is the first report on a possible association of *Tspan12* expression with HF development.

## Competing interests

The authors declare that they have no competing interests.

## Author’ contributions

MG, BB, UM and MM conceived and designed the experiments. UM, MM carried out the animal work and co-wrote the paper. MG and BB performed the microarray experiments. DT and AM performed the RT-qPCR experiments. DT analyzed the data and wrote the paper. MG, BB critically reviewed the manuscript. All authors read and approved the final manuscript.

## Pre-publication history

The pre-publication history for this paper can be accessed here:

http://www.biomedcentral.com/1755-8794/6/49/prepub

## Supplementary Material

Additional file 1 Primer sequences and parameters used to validate the microarray analysis by RT-qPCR. Click here for file

Additional file 2 Transcripts differentially expressed in LVs between rats with large-sized infarction and sham-operated ones. Click here for file

Additional file 3 AmiGO Term Enrichment analysis of differentially expressed transcripts in LVs between rats with large-size of infarction and sham-operated ones. Click here for file

Additional file 4 Differentially expressed genes related to GO:0031012: extracellular matrix. Click here for file

Additional file 5 Transcripts differentially expressed in PBMCs between rats with large-size of infarction and sham-operated ones. Click here for file

Additional file 6 Chart report from DAVID analysis to determine GO term enrichment of BP (biological process), MF (molecular function) and CC (cellular component) and their associated genes differentially expressed in PBMCs between L-MI and sham operated group. Click here for file

Additional file 7 Genes that were altered similarly in LVs and PBMCs - microarray results. Click here for file

Additional file 8 Comparison of results from RT-qPCR of genes that were altered similarly in LVs and PBMCs. Click here for file
