# Supplementary material for: Transcriptional profiling of left ventricle and peripheral blood mononuclear cells in a rat model of postinfarction heart failure
Source: BMC Med Genomics. 2013 Nov 8;6:49. doi: 10.1186/1755-8794-6-49 (PMC4226214; doi:10.1186/1755-8794-6-49)
Supplement: Additional file 1 — Primer sequences and parameters used to validate the microarray analysis by RT-qPCR. [file 1755-8794-6-49-S1.doc]

**Additional file 1. Primer sequences and reaction parameters used to validate the microarray analysis by RT-qPCR**

| **Gene symbol** | **Gene name** | **GenBank ID**  **for mRNA record** | **Sequence of primer** | **Amplicon size (bp)** | **Target exons** | **Efficiency** | **Tm (˚C)** | **Extension time** |
| --- | --- | --- | --- | --- | --- | --- | --- | --- |
| *Gapdh* | glyceraldehyde-3-phosphate dehydrogenase | NM_017008.4 | F:TTGTCAGCAATGCATCCTGC  R:CGGCATGTCAGATCCACAAC | 281 | 5-6 | 1.89 | 60 | 12‘‘ |
| *Hprt1* | hypoxanthine phosphoribosyltransferase 1 | NM_012583.2 | F: CTGACCTGCTGGATTACA  R: CCGTTGACTGGTCATTAC | 108 | 3-4 | 2.01 | 56 | 7‘‘ |
| *Nppa* | natriuretic peptide A precursor | NM_012612.2 | F:AGAGCGGACTAGGCTGCAAC  R:GGCAATGCGACCAAGCTGTG | 116 | 2-3 | 2.06 | 56 | 7‘‘ |
| *Nppb* | natriuretic peptide B precursor | NM_031545.1 | F:CAGAAGCTGCTGGAGCTGAT  R:AGGCGCTGTCTTGAGACCTA | 121 | 2 | 2.02 | 56 | 7‘‘ |
| *Hcn4* | hyperpolarization activated cyclic nucleotide-gated potassium channel 4 | NM_021658.1 | F:AGGAGGTTGCAGTCCTTGAT  R:ATCCAGGAGGTGCAGAGAAT | 133 | 8 | 1.90 | 55 | 7‘‘ |
| *Corin* | corin, serine peptidase | NM_182473.1 | F:AAGTTCGAGTGCCTGGTGTT  R:TGATGTTCACGCAGGTGCTT | 153 | 3-4 | 2.06 | 56 | 8‘‘ |
| *End1* | endothelin 1 | NM_012548.2 | F:AGAAGGTTGGAGGCCATCAG  R:AGCATGGAGAGCGCAGAGTT | 186 | 4-5 | 2.10 | 56 | 9‘‘ |
| *Pparg* | peroxisome proliferator-activated receptor gamma | NM_001145367.1 | F:CTGCTCCACACTATGAAGAC  R:TGTGGCCTGTTGTAGAGTTG | 160 | 4-5 | 2.01 | 51 | 9‘‘ |
| *Col1a1* | collagen, type I, alpha 1 | NM_053304.1 | F:CCTCTGTGCCTCAGAAGAAC  R:CGGAACCTTCGCTTCCATAC | 117 | 49-50 | 1.93 | 53 | 8‘‘ |
| *Col1a2* | collagen, type I, alpha 2 | NM_053356.1 | F:CCAGGCCAACAAGCATGTCT  R:TTGGCTAGCAGGCGCATGA | 126 | 50-51 | 1.90 | 53 | 8‘‘ |
| *Col3a1* | collagen, type III, alpha 1 | NM_032085.1 | F:ACAGATGCTGGTGCTGAGAA  R:GAAGGCCAGCTGTACATCA | 120 | 49-50 | 1.95 | 53 | 8‘‘ |
| *Col8a1* | collagen, type VIII, alpha 1 | NM_001107100.1 | F:ATGGAGTGAAGCCTCCACAT  R:TACAGTCAGCTCGGCAGTAA | 92 | 4 | 1.95 | 53 | 7‘‘ |
| *Col12a1* | collagen, type XII, alpha 1 | XM_243912.6 | F:GACCATGCAGAAGGCAATCC  R:TCTGGCTGCAGGTTCCTAAG | 122 | 32-33 | 1.89 | 53 | 8‘‘ |
| *Col14a1* | collagen, type XIV, alpha 1 | NM_001130548.1 | F:TTGCCTCTGGTTGGAGAGTT  R:ACCGGAATCCACATCAGCTT | 146 | 15-16 | 1.99 | 53 | 7‘‘ |
| *Col18a1* | collagen, type XVIII, alpha 1 | NM_053489.2 | F:GCGAGCAGATGACATCTTGG  R:GGCCGGTGTTCATGAGAACT | 117 | 43-44 | 1.84 | 50 | 7‘‘ |
| *Fn1* | fibronectin 1 | NM_019143.2 | F:GACGCCATTCCAGGAGAGTT  R:AGTCAGAGTCGCACTGGTAG | 112 | 40-41 | 1.90 | 53 | 8‘‘ |
| *Lamc1* | laminin, gamma 1 | NM_053966.2 | F:TAACACGGCTGGCTTCTACT  R:CTGCTGCTGTACTGTACCAT | 124 | 14-15 | 2.00 | 53 | 7‘‘ |
| *Eln* | elastin | NM_012722.1 | F:CAAGGCAGGCCAGTATGGTT  R:GTGCCAGGACCAGTTCCAAT | 123 | 24-25 | 2.04 | 52 | 9‘‘ |
| *Vtn* | vitronectin | NM_019156.2 | F:CAGAGCTGCTGTGTTGACTA  R:CCAGTGCTGGTACTGTTCTT | 131 | 2-3 | 2.00 | 52 | 9‘‘ |
| *Tgfβ1* | transforming growth factor, beta 1 | NM_021578.2 | F:GGCACCATCCATGACATGAA  R:TCTGTGGAGCTGAAGCAGTA | 128 | 5-6 | 1.96 | 53 | 8‘‘ |
| *Tgfβ2* | transforming growth factor, beta 2 | NM_031131.1 | F:CCAAGGCCAGAGTGGCTGAA  R:GCGTCTGTCACGTCGAAGGA | 148 | 3-4 | 1.92 | 53 | 8‘‘ |
| *Timp1* | TIMP metallopeptidase inhibitor 1 | NM_053819.1 | F:CATCGAGACCACCTTATACC  R:TATCCACAGAGGCTCTCCAT | 129 | 3-4 | 2.03 | 52 | 8‘‘ |
| *Timp2* | TIMP metallopeptidase inhibitor 2 | NM_021989.2 | F:AAGGACCTGACAAGGACATC  R:TGCATCTTGCCATCTCCTTC | 130 | 3-4 | 1.90 | 52 | 8‘‘ |
| *Timp3* | TIMP metallopeptidase inhibitor 3 | NM_012886.2 | F:GGCACTCTGGTCTACACTAT  R:CTGTCAGCAGGTACTGGTAT | 145 | 2-3 | 1.90 | 52 | 8‘‘ |
| *Timp4* | TIMP metallopeptidase inhibitor 4 | NM_001109393.1 | F:GGCCAGATTCTCAGTGATGGA  R:CGGCACTGCATAGCAAGTGGT | 150 | 4-5 | 1.88 | 52 | 8‘‘ |
| *Mmp2* | matrix metallopeptidase 2 | NM_031054.2 | F:CCAAGAACTTCCGACTATCC  R:CCAGTACCAGTGTCAGTATC | 91 | 9-10 | 1.92 | 49 | 7‘‘ |
| *Mmp9* | matrix metallopeptidase 9 | NM_031055.1 | F:CCAGGAGTCTGGATAAGTTG  R:GATCCACCTTCTGAGACTTC | 138 | 11-12 | 2.00** | 50 | 8‘‘ |
| *Mmp13* | matrix metallopeptidase 13 | NM_133530.1 | F:CTCTATGGTCCAGGAGATGA  R:CAGACGCCAGAAGAATCTGT | 141 | 5-7 | 2.00** | 49 | 8‘‘ |
| *Mmp14* | matrix metallopeptidase 14 | NM_031056.1 | F:ACCGCTTCAATGAGGAGTTC  R:TGGCTCTACCTTCAGCTTCT | 179 | 9-10 | 2.00 | 60 | 10‘‘ |
| *Mmp16* | matrix metallopeptidase 16 | NM_080776.1 | F:CTCCACCTACAAGACCTCTAC  R:GCTCCTGGATAGGAAGGTCTG | 124 | 14 | 2.00 | 54 | 7‘‘ |
| *Mmp23* | matrix metallopeptidase 23 | NM_053606.2 | F:ACAGAGACCGCCTGGAATGC  R:TCAGCGTGTAGCGGCGTCTT | 121 | 1-2 | 2.00 | 54 | 7‘‘ |
| *Spp1* | osteopontin, secreted phosphoprotein 1 | NM_012881.2 | F:GAGGTGATAGCTTGGCTTAC  R:TTGATAGCCTCATCGGACTC | 133 | 5-6 | 2.03 | 53 | 8‘‘ |
| *Postn* | periostin, osteoblast specific factor | NM_001108550.1 | F:GGCGTCATTCACGTTGTGGA  R:GTGCTGCCACGAACGAACTT | 125 | 14-16 | 2.00 | 50 | 7‘‘ |
| *Thbs4* | thrombospondin 4 | NM_017133.1 | F:GCGATGAATGCGATGACGATG  R:TGACCTTGTCCTGGTCGAAGT | 159 | 16-17 | 1.99 | 56 | 8‘‘ |
| *Prg4* | proteoglycan 4 | NM_001105962.2 | F: AGAGACGCCACCTGCAACTG  R: TCGTGCGAAGGACTCGAAGC | 123 | 3-4 | 2.09 | 52 | 8‘‘ |
| Clr7 | C-type lectin-related protein 7 | EU128749.1 | F: TTCTGCAAGGCACAAGAGTC  R: GTGCAAGCCAATCCAGTAGT | 105 | 4-5 | 1.90 | 50 | 10’’ |
| Cp | ceruloplasmin | NM_012532 | F: AACGCCTGGAACCTGGTTAC  R: CAGCCAGACTTAGTCTCTTG | 105 | 18-19 | 1.99 | 50 | 10’’ |
| Klra7 | killer cell lectin-like receptor, subfamily A, member 7 | XM_578407.4 | F: AGTCTTCAGGGCGGCAGAAC  R: AACAGCTACCAGCCGAAGGG | 140 | 3-4 | 1.88 | 52 | 10’’ |
| Ptgs2 | prostaglandin-endoperoxide synthase 2 | NM_017232 | F: TCCTCCTTGAACACGGACTT  R: ACTGCTTGTACAGCGATTGG | 103 | 8-9 | 1.76 | 50 | 10’’ |
| Tspan12 | tetraspanin 12 | NM_001015026 | F: GACAAGGGTAGAAGAGGCGG  R: ATTCCTTTTCACGGTCCCAC | 124 | 3-4 | 2.1 | 55 | 9’’ |
