# Supplementary material for: Transcriptional profiling of left ventricle and peripheral blood mononuclear cells in a rat model of postinfarction heart failure
Source: BMC Med Genomics. 2013 Nov 8;6:49. doi: 10.1186/1755-8794-6-49 (PMC4226214; doi:10.1186/1755-8794-6-49)
Supplement: Additional file 2 — Transcripts differentially expressed in LVs between rats with large-sized infarction and sham-operated ones. [file 1755-8794-6-49-S2.doc]

**Additional file 2: Transcripts differentially expressed in LVs between rats with large-size of infarction and sham-operated ones. P-value have been adjusted using False Discovery Rate (FDR) of 5%**

| No. | Transcript Cluster ID | Gene_assignment | Gene Symbol | p-value | Fold-Change |
| --- | --- | --- | --- | --- | --- |
| Adnnotated transcripts ID | | | | | |
| 1 | 10815369 | NM_001108550 // Postn // periostin, osteoblast specific factor // 2q26 // 361945 | Postn | 1.23232e-009 | 7.99435 |
| 2 | 10820434 | ENSRNOT00000065224 // Thbs4 // thrombospondin 4 | Thbs4 | 8.81769e-009 | 6.1197 |
| 3 | 10719728 | NM_012506 // Atp1a3 // ATPase, Na+/K+ transporting, alpha 3 polypeptide | Atp1a3 | 6.03216e-006 | 5.68027 |
| 4 | 10750505 | NM_013002 // Pcp4 // Purkinje cell protein 4 | Pcp4 | 4.73627e-008 | 5.10882 |
| 5 | 10706059 | NM_001198590 // Sbsn // suprabasin | Sbsn | 1.56844e-007 | 4.20014 |
| 6 | 10720829 | NM_053469 // Hamp // hepcidin antimicrobial peptide | Hamp | 3.49189e-005 | 4.08725 |
| 7 | 10940654 | NM_012881 // secreted phosphoprotein 1, osteopontin | Spp1 | 2.24579e-005 | 4.04574 |
| 8 | 10873899 | NM_012612 // Nppa // natriuretic peptide precursor A | Nppa | 1.30049e-005 | 3.70691 |
| 9 | 10792344 | ENSRNOT00000024128 // Sfrp1 // secreted frizzled-related protein 1 | Sfrp1 | 1.25497e-009 | 3.4753 |
| 10 | 10816144 | NM_001100700 // Sfrp2 // secreted frizzled-related protein 2 | Sfrp2 | 1.94875e-007 | 3.38669 |
| 11 | 10764069 | NM_053560 // Chi3l1 // chitinase 3-like 1 | Chi3l1 | 5.66273e-008 | 3.23778 |
| 12 | 10717233 | NM_022266 // Ctgf // connective tissue growth factor | Ctgf | 6.73292e-006 | 3.11136 |
| 13 | 10842043 | NM_031590 // Wisp2 // WNT1 inducible signaling pathway protein 2 | Wisp2 | 1.13639e-008 | 3.08331 |
| 14 | 10927361 | NM_001108211 // Ankrd23 // ankyrin repeat domain 23 | Ankrd23 | 8.78625e-006 | 3.05089 |
| 15 | 10917183 | NM_031521 // Ncam1 // neural cell adhesion molecule 1 | Ncam1 | 0.000933812 | 3.01414 |
| 16 | 10928761 | NM_019143 // Fn1 // fibronectin 1 | Fn1 | 6.14622e-008 | 2.93094 |
| 17 | 10918869 | ENSRNOT00000051159 // Col12a1 // collagen, type XII, alpha 1 | Col12a1 | 3.37048e-008 | 2.91044 |
| 18 | 10935041 | ENSRNOT00000046091 // LOC680319 // hypothetical protein LOC680319 | LOC680319 | 5.0485e-005 | 2.89719 |
| 19 | 10917883 | NM_001012125 // Loxl1 // lysyl oxidase-like 1 | Loxl1 | 6.55422e-008 | 2.85464 |
| 20 | 10853091 | NM_030840 // Slc26a5 // solute carrier family 26, member 5 (prestin) | Slc26a5 | 2.03772e-006 | 2.75546 |
| 21 | 10940627 | NM_001013062 // thrombospondin 1 | Thbs1 | 4.7904e-006 | 2.7268 |
| 22 | 10839434 | NM_022182 // Fgf7 // fibroblast growth factor 7 | Fgf7 | 0.000942496 | 2.70899 |
| 23 | 10891165 | NM_021586 // Ltbp2 // latent transforming growth factor beta binding protein 2 | Ltbp2 | 7.85083e-009 | 2.60937 |
| 24 | 10928614 | NM_001077656 // Myl1 // myosin, light polypeptide 1 | Myl1 | 2.39782e-005 | 2.60387 |
| 25 | 10746327 | NM_031601 // Cacna1g // calcium channel, voltage-dependent, T type, alpha 1G subunit | Cacna1g | 2.22944e-008 | 2.46089 |
| 26 | 10828854 | NM_001170481 // Pi16 // peptidase inhibitor 16 | Pi16 | 2.2355e-007 | 2.43525 |
| 27 | 10759173 | NM_001013230 // Selplg // selectin P ligand | Selplg | 0.000587212 | 2.38776 |
| 28 | 10910926 | NM_001108161 // Cilp // cartilage intermediate layer protein, nucleotide pyrophohydrolase | Cilp | 1.44333e-005 | 2.36627 |
| 29 | 10824140 | NM_001107702 // Fcrls // Fc receptor-like S, scavenger receptor | Fcrls | 5.46461e-008 | 2.36498 |
| 30 | 10936482 | NM_053819 // Timp1 // TIMP metallopeptidase inhibitor 1 | Timp1 | 2.79585e-007 | 2.34969 |
| 31 | 10725778 | NM_053611 // Nupr1 // nuclear protein, transcriptional regulator, 1 | Nupr1 | 1.5239e-008 | 2.33264 |
| 32 | 10903725 | NM_012870 // Tnfrsf11b // tumor necrosis factor receptor superfamily, member 11b | Tnfrsf11b | 2.02959e-008 | 2.32907 |
| 33 | 10737532 | NM_053304 // Col1a1 // collagen, type I, alpha 1 | Col1a1 | 6.06025e-006 | 2.31371 |
| 34 | 10858499 | NM_001108644 // Mfap5 // microfibrillar associated protein 5 | Mfap5 | 7.56962e-009 | 2.29512 |
| 35 | 10814430 | NM_012532 // Cp // ceruloplasmin | Cp | 1.8502e-009 | 2.28215 |
| 36 | 10709788 | NM_001108497 // RGD1306959 // similar to C11orf17 protein | RGD1306959 | 1.36877e-006 | 2.25782 |
| 37 | 10849327 | NM_031825 // Fbn1 // fibrillin 1 | Fbn1 | 5.21593e-007 | 2.2484 |
| 38 | 10848038 | NM_001025042 // Fibin // fin bud initiation factor homolog (zebrafish) | Fibin | 1.90798e-008 | 2.2344 |
| 39 | 10820282 | NM_001170558 // Vcan // versican | Vcan | 1.92515e-006 | 2.23098 |
| 40 | 10935890 | NM_017087 // Bgn // biglycan | Bgn | 8.8887e-010 | 2.21586 |
| 41 | 10764050 | NM_080698 // Fmod // fibromodulin | Fmod | 1.47187e-005 | 2.21249 |
| 42 | 10881624 | ENSRNOT00000012726// LOC100366245 // rCG30616-like | LOC100366245 | 0.000607704 | 2.20312 |
| 43 | 10791000 | NM_001106074 // Crlf1 // cytokine receptor-like factor 1 | Crlf1 | 2.74564e-007 | 2.19666 |
| 44 | 10834005 | NM_013115 // Ptgfr // prostaglandin F receptor | Ptgfr | 0.000162192 | 2.16641 |
| 45 | 10764551 | NM_017232 // Ptgs2 // prostaglandin-endoperoxide synthase 2 | Ptgs2 | 9.90462e-006 | 2.16193 |
| 46 | 10910473 | NM_021658 // Hcn4 // hyperpolarization activated cyclic nucleotide-gated potassium chanel 4 | Hcn4 | 0.00148625 | 2.14458 |
| 47 | 10841693 | NM_017208 // Lbp // lipopolysaccharide binding protein | Lbp | 2.57588e-006 | 2.13422 |
| 48 | 10750685 | NM_001107100 // Col8a1 // collagen, type VIII, alpha 1 | Col8a1 | 3.06533e-008 | 2.12977 |
| 49 | 10775731 | NM_001017496 // Cxcl13 // chemokine (C-X-C motif) ligand 13 | Cxcl13 | 0.00381517 | 2.1206 |
| 50 | 10917205 | [GENSCAN00000013683](https://www.affymetrix.com/analysis/netaffx/exon/rna.affx?pk=135088560) |  | 6.00384e-005 | 2.11625 |
| 51 | 10817419 | NM_031560 // Ctsk // cathepsin K | Ctsk | 0.000163111 | 2.10489 |
| 52 | 10922964 | ENSRNOT00000036990 // RGD1305645 // similar to RIKEN cDNA 1500015O10 | RGD1305645 | 1.24265e-006 | 2.09293 |
| 53 | 10857278 | ENSRNOT00000009696 // Fbln2 // fibulin 2 | Fbln2 | 2.68478e-007 | 2.08862 |
| 54 | 10912255 | NM_175869 // Plod2 // procollagen lysine, 2-oxoglutarate 5-dioxygenase 2 | Plod2 | 4.19657e-008 | 2.06955 |
| 55 | 10857314 | NM_017206 // Slc6a6 // solute carrier family 6 (neurotransmitter transporter, taurine) member 6 | Slc6a6 | 1.58887e-007 | 2.04249 |
| 56 | 10801483 | NM_001106148 // Myot // myotilin | Myot | 0.00010421 | 2.02709 |
| 57 | 10817057 | NM_012618 // S100a4 // S100 calcium-binding protein A4 | S100a4 | 7.36496e-010 | 2.02054 |
| 58 | 10770577 | NM_031131 // Tgfb2 // transforming growth factor, beta 2 | Tgfb2 | 7.8036e-005 | 2.01264 |
| 59 | 10822242 | NM_019292 // Car3 // carbonic anhydrase 3 | Car3 | 0.00390685 | 1.98265 |
| 60 | 10902547 | NM_012771 // Lyz2 // lysozyme 2 | Lyz2 | 2.08475e-006 | 1.97899 |
| 61 | 10808177 | NM_001108899 // Clec3a // C-type lectin domain family 3, member a | Clec3a | 0.00224715 | 1.97262 |
| 62 | 10889446 | BC158664 // Colec11 // collectin sub-family member 11 | Colec11 | 3.64341e-007 | 1.96674 |
| 63 | 10736697 | NM_031530 // Ccl2 // chemokine (C-C motif) ligand 2 | Ccl2 | 0.00393348 | 1.95704 |
| 64 | 10896558 | NM_001130548 // Col14a1 // collagen, type XIV, alpha 1 | Col14a1 | 1.01042e-007 | 1.95689 |
| 65 | 10940446 | NM_012834 // cartilage oligomeric matrix protein | Comp | 1.06381e-005 | 1.95396 |
| 66 | 10724895 | NM_001106286 // Lyve1 // lymphatic vessel endothelial hyaluronan receptor 1 | Lyve1 | 4.06744e-006 | 1.95253 |
| 67 | 10767388 | NM_022269 // Cd55 // Cd55 molecule | Cd55 | 1.92456e-006 | 1.95248 |
| 68 | 10794734 | NM_021698 // F13a1 // coagulation factor XIII, A1 polypeptide | F13a1 | 2.48449e-006 | 1.94618 |
| 69 | 10809428 | NM_017327 // Gnao1 // guanine nucleotide binding protein (G protein), alpha activating activity polypeptide O | Gnao1 | 1.07997e-007 | 1.94327 |
| 70 | 10865369 | NM_032060 // C3ar1 // complement component 3a receptor 1 | C3ar1 | 1.96963e-006 | 1.93402 |
| 71 | 10724967 | NM_138519 // Dkk3 // dickkopf homolog 3 (Xenopus laevis) | Dkk3 | 1.06174e-008 | 1.92534 |
| 72 | 10929458 | NM_001127492 // Sphkap // SPHK1 interactor, AKAP domain containing | Sphkap | 0.00101178 | 1.92367 |
| 73 | 10759177 | NM_001107155 // Tmem119 // transmembrane protein 119 | Tmem119 | 7.04228e-006 | 1.91316 |
| 74 | 10768269 | NM_130409 // Cfh // complement factor H | Cfh | 9.21304e-006 | 1.9099 |
| 75 | 10880727 | NM_019262 // C1qb // complement component 1, q subcomponent, B chain | C1qb | 3.13341e-006 | 1.90866 |
| 76 | 10916379 | FQ223043 // TL0ADA16YA23 | TL0ADA16YA23 | 4.73835e-008 | 1.90807 |
| 77 | 10880731 | NM_001008524 // C1qc // complement component 1, q subcomponent, C chain | C1qc | 1.10845e-005 | 1.89957 |
| 78 | 10749762 | NM_199082 // Sectm1b // secreted and transmembrane 1B | Sectm1b | 3.58705e-006 | 1.89359 |
| 79 | 10825992 | NM_001108563 // Sypl2 // synaptophysin-like 2 // 2q34 // 362018 /// ENSRNOT00000 | Sypl2 | 1.45768e-005 | 1.88932 |
| 80 | 10744939 | NM_177927 // Serpinf1 // serine (or cysteine) peptidase inhibitor, clade F, member 1 | Serpinf1 | 2.74527e-008 | 1.87879 |
| 81 | 10737426 | NM_001017479 // Tmem100 // transmembrane protein 100 | Tmem100 | 6.40813e-005 | 1.87211 |
| 82 | 10906926 | NM_001013222 // Rnd1 // Rho family GTPase 1 | Rnd1 | 3.88598e-005 | 1.86936 |
| 83 | 10744127 | NM_001031638 // Cd68 // Cd68 molecule | Cd68 | 6.7222e-007 | 1.8647 |
| 84 | 10921047 | NM_078621 // Ccbp2 // chemokine binding protein 2 | Ccbp2 | 1.08495e-006 | 1.85698 |
| 85 | 10783880 | NM_031659 // Tgm1 // transglutaminase 1, K polypeptide | Tgm1 | 0.000630135 | 1.85257 |
| 86 | 10834670 | ENSRNOT00000036995 // Adamtsl2 // ADAMTS-like 2 | Adamtsl2 | 3.07191e-007 | 1.84132 |
| 87 | 10797660 | NM_001014008 // Aspn // asporin | Aspn | 1.61654e-007 | 1.8357 |
| 88 | 10877217 | NM_147215 // Obp3 // alpha-2u globulin PGCL4 | Obp3 | 6.28654e-006 | 1.83175 |
| 89 | 10920981 | NM_133534 // Cx3cr1 // chemokine (C-X3-C motif) receptor 1 | Cx3cr1 | 3.43646e-005 | 1.82257 |
| 90 | 10936899 | NM_023965 // Cybb // cytochrome b-245, beta polypeptide | Cybb | 5.99108e-007 | 1.82172 |
| 91 | 10813253 | NM_176074 // C6 // complement component 6 | C6 | 0.00060949 | 1.81979 |
| 92 | 10859296 | NM_012843 // Emp1 // epithelial membrane protein 1 | Emp1 | 5.23085e-007 | 1.81723 |
| 93 | 10909407 | NM_012673 // Thy1 // Thy-1 cell surface antigen | Thy1 | 9.47447e-006 | 1.81657 |
| 94 | 10855449 | NM_133298 // Gpnmb // glycoprotein (transmembrane) nmb | Gpnmb | 2.09615e-005 | 1.8076 |
| 95 | 10754218 | NM_024369 // Fstl1 // follistatin-like 1 | Fstl1 | 4.47953e-009 | 1.80384 |
| 96 | 10880833 | NM_001108688 // Cda // cytidine deaminase | Cda | 0.000159008 | 1.79825 |
| 97 | 10796476 | NM_001106123 // Mrc1 // mannose receptor, C type 1 | Mrc1 | 2.10566e-007 | 1.79342 |
| 98 | 10724111 | NM_001106283 // Folr2 // folate receptor 2 (fetal) | Folr2 | 9.06213e-006 | 1.78154 |
| 99 | 10825022 | NM_053882 // Ecm1 // extracellular matrix protein 1 | Ecm1 | 4.72019e-007 | 1.77236 |
| 100 | 10817429 | NM_017320 // Ctss // cathepsin S | Ctss | 5.69846e-007 | 1.77222 |
| 101 | 10937725 | NM_031761 // Figf // c-fos induced growth factor | Figf | 1.38826e-005 | 1.77019 |
| 102 | 10880734 | NM_001008515 // C1qa // complement component 1, q subcomponent, A chain | C1qa | 1.71445e-005 | 1.76752 |
| 103 | 10776739 | NM_017237 // Uchl1 // ubiquitin carboxyl-terminal esterase L1 (ubiquitin thiolesterase) | Uchl1 | 5.47357e-006 | 1.76517 |
| 104 | 10858559 | NM_001005891 // Clec4a3 // C-type lectin domain family 4, member a3 | Clec4a3 | 2.13019e-006 | 1.76517 |
| 105 | 10761025 | ENSRNOT00000039797 // LOC685020 // similar to paired immunoglobin-like type 2 receptor | LOC685020 | 0.000280294 | 1.76419 |
| 106 | 10909328 | NM_139097 // Scn3b // sodium channel, voltage-gated, type III, beta | Scn3b | 2.33389e-005 | 1.7603 |
| 107 | 10799548 | ENSRNOT00000024110 // Ccdc3 // coiled-coil domain containing 3 | Ccdc3 | 5.78679e-005 | 1.75447 |
| 108 | 10729913 | NM_001191636 // Myof // myoferlin | Myof | 3.67633e-008 | 1.75388 |
| 109 | 10728930 | XM_002725787 // LOC690930 // similar to membrane-spanning 4-domains, subfamily A | LOC690930 | 0.000125327 | 1.75317 |
| 110 | 10929288 | NM_019197 // Serpine2 // serine (or cysteine) peptidase inhibitor, clade E, member 2 | Serpine2 | 1.16775e-005 | 1.75071 |
| 111 | 10933345 | NM_001097582 // Tlr7 // toll-like receptor 7 | Tlr7 | 1.57748e-005 | 1.74435 |
| 112 | 10916247 | NM_199398 // Panx3 // pannexin 3 | Panx3 | 0.00463055 | 1.74131 |
| 113 | 10859090 | ENSRNOT00000049620 // LOC689800 // similar to osteoclast inhibitory lectin | LOC689800 | 2.40628e-006 | 1.73519 |
| 114 | 10804463 | NM_017061 // Lox // lysyl oxidase | Lox | 2.46257e-006 | 1.72888 |
| 115 | 10865329 | NM_012907 // Apobec1 // apolipoprotein B mRNA editing enzyme, catalytic polypeptid | Apobec1 | 2.16782e-005 | 1.72858 |
| 116 | 10768412 | NM_001105962 // Prg4 // proteoglycan 4 | Prg4 | 0.00096692 | 1.72504 |
| 117 | 10789929 | NM_133307 // Prkcd // protein kinase C, delta | Prkcd | 7.19889e-006 | 1.72245 |
| 118 | 10899465 | NM_013104 // Igfbp6 // insulin-like growth factor binding protein 6 | Igfbp6 | 4.28902e-006 | 1.7197 |
| 119 | 10882882 | NM_001191930 // Uap1 // UDP-N-acteylglucosamine pyrophosphorylase 1 // 13q24 // | Uap1 | 1.05809e-0062 | 1.71873 |
| 120 | 10721339 | NM_001012459 // Clec11a // C-type lectin domain family 11, member a | Clec11a | 0.000432475 | 1.71816 |
| 121 | 10737513 | NM_019164 // Chad // chondroadherin | Chad | 0.00282446 | 1.71542 |
| 122 | 10788427 | NM_001191939 // Msr1 // macrophage scavenger receptor 1 | Msr1 | 1.05176e-005 | 1.71363 |
| 123 | 10804245 | NM_012934 // Dpysl3 // dihydropyrimidinase-like 3 | Dpysl3 | 4.54809e-006 | 1.71201 |
| 124 | 10803991 | NM_021744 // Cd14 // CD14 molecule | Cd14 | 2.43523e-005 | 1.71047 |
| 125 | 10865585 | NM_012705 // Cd4 // Cd4 molecule | Cd4 | 6.35193e-007 | 1.70792 |
| 126 | 10931222 | NM_001007557 // Emr1 // EGF-like module containing, mucin-like, hormone receptor | Emr1 | 3.55646e-006 | 1.70546 |
| 127 | 10835339 | ENSRNOT00000011676 // LOC688582 // Uncharacterized protein | LOC688582 | 5.13084e-006 | 1.70408 |
| 128 | 10858626 | NM_001107887 // Cd163 // CD163 molecule | Cd163 | 0.000315247 | 1.70376 |
| 129 | 10748861 | NM_001202463 // Cd300 molecule-like family member E, pseudogene 1 | Cd300le-ps1 | 0.000446364 | 1.70324 |
| 130 | 10835322 | XR_085779 // LOC688582 // similar to hemicentin 1 // 3p12 // 688582 | LOC688582 | 8.47179e-006 | 1.70252 |
| 131 | 10866507 | NM_001173509 // Art4 // ADP-ribosyltransferase 4 | Art4 | 0.00103331 | 1.6924 |
| 132 | 10862842 | NM_031644 // Hpgds // hematopoietic prostaglandin D synthase | Hpgds | 0.000129822 | 1.69187 |
| 133 | 10802065 | NM_001029901 // Csf1r // colony stimulating factor 1 receptor | Csf1r | 8.77999e-007 | 1.69026 |
| 134 | 10868416 | ENSRNOT00000047893 // RGD1559864 // similar to mKIAA1045 protein | RGD1559864 | 3.59074e-006 | 1.68325 |
| 135 | 10820145 | ENSRNOT00000039652 // RGD1562101 // similar to very large G-protein coupled receptor | RGD1562101 | 9.77082e-005 | 1.68165 |
| 136 | 10926245 | NM_001106884 // Trem2 // triggering receptor expressed on myeloid cells 2 | Trem2 | 5.29053e-006 | 1.68132 |
| 137 | 10733139 | NM_001002819 // Gfpt2 // glutamine-fructose-6-phosphate transaminase 2 | Gfpt2 | 0.000200199 | 1.67754 |
| 138 | 10779673 | NM_031832 // Lgals3 // lectin, galactoside-binding, soluble, 3 | Lgals3 | 5.78048e-006 | 1.67512 |
| 139 | 10873336 | NM_001013428 // Pla2g2d // phospholipase A2, group IID | Pla2g2d | 0.0003352 | 1.67322 |
| 140 | 10798702 | NM_017128 // Inhba // inhibin beta-A | Inhba | 3.15584e-006 | 1.67041 |
| 141 | 10765746 | NM_001105971 // Slamf9 // SLAM family member 9 | Slamf9 | 1.34473e-005 | 1.66971 |
| 142 | 10723576 | NM_001007691 // Prss23 // protease, serine, 23 | Prss23 | 1.40526e-005 | 1.66806 |
| 143 | 10769788 | NM_053843 // Fcgr2a // Fc fragment of IgG, low affinity IIa, receptor (CD32) | Fcgr2a | 9.52829e-006 | 1.66785 |
| 144 | 10909646 | NM_145717 // Fxyd2 // FXYD domain-containing ion transport regulator 2 | Fxyd2 | 1.90876e-005 | 1.66372 |
| 145 | 10846740 | NM_001100527 // Frzb // frizzled-related protein | Frzb | 0.00134689 | 1.65748 |
| 146 | 10781304 | NM_001106047 // Loxl2 // lysyl oxidase-like 2 | Loxl2 | 1.5788e-007 | 1.65636 |
| 147 | 10795989 | NM_206847 // Pfkp // phosphofructokinase, platelet | Pfkp | 3.31263e-008 | 1.65472 |
| 148 | 10720859 | NM_021909 // Fxyd5 // FXYD domain-containing ion transport regulator 5 | Fxyd5 | 2.5144e-007 | 1.65391 |
| 149 | 10890860 | NM_031005 // Actn1 // actinin, alpha 1 | Actn1 | 3.0039e-005 | 1.6533 |
| 150 | 10863777 | NM_001044249 // Antxr1 // anthrax toxin receptor 1 | Antxr1 | 6.26963e-006 | 1.65148 |
| 151 | 10900782 | NM_053629 // Fstl3 // follistatin-like 3 (secreted glycoprotein) | Fstl3 | 0.000124999 | 1.64973 |
| 152 | 10740869 | NM_181086 // Tnfrsf12a // tumor necrosis factor receptor superfamily, member 12a | Tnfrsf12a | 2.1981e-005 | 1.64945 |
| 153 | 10940549 | NM_053619 // complement component 5a receptor 1 | C5ar1 | 2.77309e-005 | 1.64762 |
| 154 | 10866512 | NM_012862 // Mgp // matrix Gla protein | Mgp | 5.70129e-009 | 1.64017 |
| 155 | 10876935 | NM_001127565 // RGD1562988 // similar to EHM2 | RGD1562988 | 7.47898e-009 | 1.63752 |
| 156 | 10823555 | NM_001014790 // Rarres1 // retinoic acid receptor responder (tazarotene induced) | Rarres1 | 3.74304e-005 | 1.63639 |
| 157 | 10917034 | NM_031549 // Tagln // transgelin | Tagln | 1.50609e-005 | 1.63475 |
| 158 | 10774163 | NM_001135915 // LOC688553 // hypothetical protein LOC688553 | LOC688553 | 4.54624e-005 | 1.63277 |
| 159 | 10821991 | NM_012868 // Npr3 // natriuretic peptide receptor C/guanylate cyclase C | Npr3 | 0.00287426 | 1.62797 |
| 160 | 10866019 | NM_001173386 // Clec7a // C-type lectin domain family 7, member a | Clec7a | 4.61881e-005 | 1.62606 |
| 161 | 10895585 | ENSRNOT00000007338 // Lrrc10 // leucine-rich repeat-containing 10 | Lrrc10 | 1.26134e-005 | 1.62475 |
| 162 | 10846301 | NM_001127481 // Pde11a // phosphodiesterase 11A | Pde11a | 0.000276853 | 1.62211 |
| 163 | 10735959 | NM_001105812 // Slc43a2 // solute carrier family 43, member 2 | Slc43a2 | 0.000187782 | 1.61912 |
| 164 | 10729269 | NM_012904 // Anxa1 // annexin A1 | Anxa1 | 5.13528e-006 | 1.61688 |
| 165 | 10719530 | NM_138828 // Apoe // apolipoprotein E | Apoe | 3.21652e-005 | 1.61105 |
| 166 | 10702913 | NM_001038615 // Fndc1 // fibronectin type III domain containing 1 | Fndc1 | 7.97407e-005 | 1.61088 |
| 167 | 10862634 | NM_001025063 // Scrn1 // secernin 1 | Scrn1 | 3.48829e-005 | 1.60599 |
| 168 | 10759989 | ENSRNOT00000001205 // RGD1307396 // similar to RIKEN cDNA 6330406I15 | RGD1307396 | 0.00032852 | 1.60522 |
| 169 | 10723240 | NM_001106276 // Cpeb1 // cytoplasmic polyadenylation element binding protein 1 | Cpeb1 | 8.42542e-005 | 1.60467 |
| 170 | 10793133 | NM_001109389 // LOC680045 // hypothetical protein LOC680045 | LOC680045 | 0.000305953 | 1.60418 |
| 171 | 10919637 | NM_001013110 // Tf // transferrin | Tf | 1.0152e-005 | 1.59417 |
| 172 | 10931717 | NM_016994 // C3 // complement component 3 | C3 | 0.000233289 | 1.59212 |
| 173 | 10767763 | NM_053385 // Prelp // proline/arginine-rich end leucine-rich repeat protein | Prelp | 2.94075e-006 | 1.59115 |
| 174 | 10930259 | NM_145094 // Rab31 // RAB31, member RAS oncogene family | Rab31 | 2.28149e-006 | 1.59011 |
| 175 | 10936717 | NM_033653 // Maoa // monoamine oxidase A | Maoa | 0.000307067 | 1.58564 |
| 176 | 10790023 | ENSRNOT00000025394 // LOC100363145 // stabilin 1 | LOC100363145 | 1.89997e-006 | 1.58528 |
| 177 | 10780919 | NM_001013433 // Arl11 // ADP-ribosylation factor-like 11 | Arl11 | 2.19161e-005 | 1.58504 |
| 178 | 10847432 | NM_030859 // Mdk // midkine | Mdk | 4.68274e-005 | 1.58338 |
| 179 | 10759999 | NM_017260 // Alox5ap // arachidonate 5-lipoxygenase activating protein | Alox5ap | 2.91878e-007 | 1.5809 |
| 180 | 10853559 | NM_053356 // Col1a2 // collagen, type I, alpha 2 | Col1a2 | 5.62041e-005 | 1.57612 |
| 181 | 10903082 | NM_001130490 // Lrp1 // low density lipoprotein-related protein 1 | Lrp1 | 5.90672e-007 | 1.57587 |
| 182 | 10738209 | NM_012809 // Cnp // 2',3'-cyclic nucleotide 3' phosphodiesterase | Cnp | 1.70058e-006 | 1.57369 |
| 183 | 10912289 | ENSRNOT00000011358 // Slc9a9 // solute carrier family 9 (sodium/hydrogen exchang | Slc9a9 | 3.66695e-007 | 1.57334 |
| 184 | 10825153 | NM_001100836 // Fcgr1a // Fc fragment of IgG, high affinity Ia, receptor (CD64) | Fcgr1a | 1.26961e-005 | 1.57096 |
| 185 | 10708021 | NM_022190 // Acan // aggrecan | Acan | 0.00413875 | 1.57095 |
| 186 | 10761446 | ENSRNOT00000058586 // LOC689257 // similar to G protein-coupled receptor 133 | LOC689257 | 0.00100003 | 1.56862 |
| 187 | 10849156 | ENSRNOT00000064908 // Frmd5 // FERM domain containing 5 | Frmd5 | 4.8143e-005 | 1.56604 |
| 188 | 10911156 | ENSRNOT00000012048 // Fam148b // family with sequence similarity 148, member B | Fam148b | 0.000239692 | 1.56438 |
| 189 | 10797657 | NM_031817 // Omd // osteomodulin | Omd | 1.22814e-005 | 1.56323 |
| 190 | 10892330 | ENSRNOT00000039631 // Ahnak2 // AHNAK nucleoprotein 2 | Ahnak2 | 0.000165576 | 1.55967 |
| 191 | 10863512 | NM_001109398 // Mthfd2 // methylenetetrahydrofolate dehydrogenase (NADP+ dependent) 2 | Mthfd2 | 3.20806e-007 | 1.55959 |
| 192 | 10726230 | NM_054005 // Cuzd1 // CUB and zona pellucida-like domains 1 | Cuzd1 | 1.45606e-006 | 1.55826 |
| 193 | 10918342 | NM_001108769 // Fbxl22 // F-box and leucine-rich repeat protein 22 | Fbxl22 | 0.000138327 | 1.55815 |
| 194 | 10714323 | NM_022407 // Aldh1a1 // aldehyde dehydrogenase 1 family, member A1 | Aldh1a1 | 3.49721e-008 | 1.55671 |
| 195 | 10877307 | NM_203325 // Mup5 // major urinary protein 5 | Mup5 | 0.00178542 | 1.55497 |
| 196 | 10796838 | NM_001107330 // Irx4 // iroquois homeobox 4 | Irx4 | 2.01795e-008 | 1.55151 |
| 197 | 10804480 | NM_001004215 // Ppic // peptidylprolyl isomerase C | Ppic | 7.39461e-005 | 1.55138 |
| 198 | 10939570 | NM_001039016 // Zdhhc9 // zinc finger, DHHC-type containing 9 | Zdhhc9 | 0.000151959 | 1.55091 |
| 199 | 10882391 | NM_031609 // Nbl1 // neuroblastoma, suppression of tumorigenicity 1- | Nbl1 | 3.83238e-005 | 1.54645 |
| 200 | 10858566 | NM_001005880 // Clec4a2 // C-type lectin domain family 4, member a2 | Clec4a2 | 1.77035e-005 | 1.54296 |
| 201 | 10823368 | NM_198768 // Igsf10 // immunoglobulin superfamily, member 10 | Igsf10 | 0.000409841 | 1.541 |
| 202 | 10825994 | NM_001108563 // Sypl2 // synaptophysin-like 2 // 2q34 // 362018 /// ENSRNOT00000 | Sypl2 | 0.000155492 | 1.54059 |
| 203 | 10908776 | ENSRNOT00000007358 // Uncharacterized protein | D4aat1_RAT | 0.00050144 | 1.53978 |
| 204 | 10753222 | NM_017325 // Runx1 // runt-related transcription factor 1 | Runx1 | 6.98171e-006 | 1.53917 |
| 205 | 10851350 | NM_019386 // Tgm2 // transglutaminase 2, C polypeptide | Tgm2 | 2.37694e-006 | 1.53618 |
| 206 | 10823365 | NM_022800 // P2ry12 // purinergic receptor P2Y, G-protein coupled, 12 | P2ry12 | 7.83926e-006 | 1.53336 |
| 207 | 10721361 | NM_001106257 // Mybpc2 // myosin binding protein C, fast-type // 1q22 // 292879 | Mybpc2 | 0.000910314 | 1.53171 |
| 208 | 10866819 | NM_017253 // Bcat1 // branched chain aminotransferase 1, cytosolic | Bcat1 | 2.45335e-005 | 1.53122 |
| 209 | 10763768 | NM_001014843 // Faim3 // Fas apoptotic inhibitory molecule 3 | Faim3 | 2.50021e-005 | 1.53075 |
| 210 | 10912917 | NM_175592 // Cacna2d2 // calcium channel, voltage-dependent, alpha 2/delta subunit 2 | Cacna2d2 | 0.000533404 | 1.52987 |
| 211 | 10760971 | NM_019237 // Pcolce // procollagen C-endopeptidase enhancer | Pcolce | 2.75247e-007 | 1.52959 |
| 212 | 10864848 | NM_012822 // Alox5 // arachidonate 5-lipoxygenase | Alox5 | 0.000107323 | 1.5282 |
| 213 | 10812399 | NM_019189 // Hapln1 // hyaluronan and proteoglycan link protein 1 | Hapln1 | 0.00191204 | 1.52535 |
| 214 | 10712997 | NM_001106325 // Cd248 // CD248 molecule, endosialin | Cd248 | 4.31717e-005 | 1.52497 |
| 215 | 10912161 | NM_012939 // Ctsh // cathepsin H | Ctsh | 1.08186e-010 | 1.52489 |
| 216 | 10764891 | NM_053571 // Sec16b // SEC16 homolog B (S. cerevisiae) | Sec16b | 5.22294e-006 | 1.52384 |
| 217 | 10726255 | NM_001106306 // Cpxm2 // carboxypeptidase X (M14 family), member 2 | Cpxm2 | 0.00374554 | 1.5229 |
| 218 | 10863549 | NM_012893 // Actg2 // actin, gamma 2, smooth muscle, enteric | Actg2 | 0.000226147 | 1.52215 |
| 219 | 10858277 | NM_021595 // Ninj2 // ninjurin 2 | Ninj2 | 0.000237867 | 1.52198 |
| 220 | 10855416 | NM_001039008 // Tmem176a // transmembrane protein 176A | Tmem176a | 1.08452e-005 | 1.52153 |
| 221 | 10888953 | NM_001106710 // Emilin1 // elastin microfibril interfacer 1 | Emilin1 | 2.92703e-006 | 1.52083 |
| 222 | 10852144 | NM_001107807 // Pmepa1 // prostate transmembrane protein, androgen induced | Pmepa1 | 8.93771e-008 | 1.51777 |
| 223 | 10939437 | NM_001037365 // Bex1 // brain expressed gene 1 | Bex1 | 0.000203617 | 1.51723 |
| 224 | 10726172 | NM_012712 // Fgfr2 // fibroblast growth factor receptor 2 | Fgfr2 | 0.00143484 | 1.51584 |
| 225 | 10831628 | NM_198741 // RT1-DMa // RT1 class II, locus Dma | RT1-DMa | 1.59663e-005 | 1.51542 |
| 226 | 10860878 | NM_001004086 // Pon3 // paraoxonase 3 | Pon3 | 0.000741858 | 1.51519 |
| 227 | 10708053 | NM_001106275 // Abhd2 // abhydrolase domain containing 2 // 1q31 // 293050 /// E | Abhd2 | 2.03213e-005 | 1.51414 |
| 228 | 10851947 | NM_031557 // Ptgis // prostaglandin I2 (prostacyclin) synthase | Ptgis | 3.31532e-005 | 1.51367 |
| 229 | 10923270 | NM_001014216 // Obfc2a // oligonucleotide/oligosaccharide-binding fold containin | Obfc2a | 7.18646e-006 | 1.51367 |
| 230 | 10770109 | ENSRNOT00000058058 // Uncharacterized protein | D3zt11 | 0.00400428 | 1.51029 |
| 231 | 10935043 | NM_001127502 // Wbp5 // WW domain binding protein 5 | Wbp5 | 1.01493e-005 | 1.50976 |
| 232 | 10816017 | NM_199105 // Fam198b // family with sequence similarity 198, member B | Fam198b | 1.32366e-006 | 1.50613 |
| 233 | 10812689 | NM_001003401 // Enc1 // ectodermal-neural cortex 1 | Enc1 | 0.000753608 | 1.50592 |
| 234 | 10837621 | NM_001108750 // Cpne8 // copine VIII | Cpne8 | 1.45863e-005 | 1.50393 |
| 235 | 10765096 | ENSRNOT00000039221 // Tnfsf18 // tumor necrosis factor (ligand) superfamily, member 18 | Tnfsf18 | 0.00033735 | 1.50185 |
| 236 | 10898315 | NM_001127547 // Fbln1 // fibulin 1 | Fbln1 | 1.19089e-005 | 1.50028 |
| 237 | 10887549 | BC086564 // RGD1307315 // LOC362793 | RGD1307315 | 3.53966e-006 | 1.4994 |
| 238 | 10748718 | NM_173307 // Abca5 // ATP-binding cassette, sub-family A (ABC1), member 5 | Abca5 | 9.04885e-007 | 1.499 |
| 239 | 10866140 | XM_002726462 // LOC100364751 // immunoreceptor Ly49si3-like | LOC100364751 | 5.677e-005 | 1.49747 |
| 240 | 10765480 | NM_001107195 // Olfml2b // olfactomedin-like 2B | Olfml2b | 0.000393199 | 1.497 |
| 241 | 10885628 | NM_001100863 // Galntl1 // UDP-N-acetyl-alpha-D-galactosamine:polypeptide N-acetylgalactosaminyltransferase-like 1 | Galntl1 | 1.5391e-005 | 1.49616 |
| 242 | 10770082 | NM_001012029 // Ifi204 // interferon activated gene 204 | Ifi204 | 7.73813e-006 | 1.4955 |
| 243 | 10851391 | NM_019316 // Mafb // v-maf musculoaponeurotic fibrosarcoma oncogene homolog B (avian) | Mafb | 7.56154e-006 | 1.49497 |
| 244 | 10928902 | NM_001007713 // Tmbim1 // transmembrane BAX inhibitor motif containing 1 | Tmbim1 | 7.22585e-007 | 1.49322 |
| 245 | 10813361 | NM_024159 // Dab2 // disabled homolog 2 (Drosophila) | Dab2 | 6.6653e-007 | 1.49245 |
| 246 | 10786028 | NM_001109386 // Gpr183 // G protein-coupled receptor 183 | Gpr183 | 7.93825e-005 | 1.49154 |
| 247 | 10734242 | NM_001034124 // Mfap4 // microfibrillar-associated protein 4 | Mfap4 | 5.00403e-006 | 1.49139 |
| 248 | 10858553 | NM_001005890 // Clec4a1 // C-type lectin domain family 4, member a1 | Clec4a1 | 0.000254184 | 1.48934 |
| 249 | 10809688 | NM_053396 // Adcy7 // adenylate cyclase 7 | Adcy7 | 9.22327e-007 | 1.48537 |
| 250 | 10768857 | NM_001109898 // Qsox1 // quiescin Q6 sulfhydryl oxidase 1 | Qsox1 | 0.00373301 | 1.48467 |
| 251 | 10938384 | NM_001106581 // Pdk3 // pyruvate dehydrogenase kinase, isozyme 3 | Pdk3 | 5.92647e-005 | 1.48371 |
| 252 | 10857460 | XM_003754442 // glycosyltransferase 8 domain containing 4 | Glt8d4 | 1.34826e-007 | 1.48172 |
| 253 | 10851952 | NM_001108608 // B4galt5 // UDP-Gal:betaGlcNAc beta 1,4-galactosyltransferase, po | B4galt5 | 0.000163576 | 1.4812 |
| 254 | 10895359 | NM_177425 // Csrp2 // cysteine and glycine-rich protein 2 | Csrp2 | 3.36457e-005 | 1.4802 |
| 255 | 10710154 | ENSRNOT00000025007 // Xylt1 // xylosyltransferase 1 | Xylt1 | 1.05561e-006 | 1.48001 |
| 256 | 10825869 | NM_023981 // Csf1 // colony stimulating factor 1 (macrophage) | Csf1 | 0.000595562 | 1.47921 |
| 257 | 10769825 | NM_001131001 // Fcer1g // Fc fragment of IgE, high affinity I, receptor for; gamma | Fcer1g | 0.000142093 | 1.47912 |
| 258 | 10732750 | NM_031321 // Slit3 // slit homolog 3 (Drosophila) | Slit3 | 0.000108856 | 1.4781 |
| 259 | 10822107 | BC107649 // Fam105a // family with sequence similarity 105, member A | Fam105a | 0.000180673 | 1.47771 |
| 260 | 10791500 | NM_033499 // Scrg1 // stimulator of chondrogenesis 1 // 16p11 // 64458 /// ENSRN | Scrg1 | 0.00118114 | 1.47748 |
| 261 | 10835332 | ENSRNOT00000034092 // Uncharacterized protein | F1M4Y6_RAT | 4.21922e-007 | 1.47682 |
| 262 | 10749484 | NM_021989 // Timp2 // TIMP metallopeptidase inhibitor 2 | Timp2 | 4.63114e-007 | 1.47623 |
| 263 | 10889308 | NM_001034952 // Pqlc3 // PQ loop repeat containing 3 | Pqlc3 | 5.56175e-005 | 1.47379 |
| 264 | 10745670 | NM_001004202 // Ccl6 // chemokine (C-C motif) ligand 6 | Ccl6 | 0.000103658 | 1.47292 |
| 265 | 10770412 | NM_001012150 // Enah // enabled homolog (Drosophila) | Enah | 8.31715e-005 | 1.47105 |
| 266 | 10909442 | NM_001012123 // C1qtnf5 // C1q and tumor necrosis factor related protein 5 | C1qtnf5 | 6.61582e-007 | 1.47017 |
| 267 | 10716880 | NM_001025638 // Stx11 // syntaxin 11 | Stx11 | 5.60026e-005 | 1.47005 |
| 268 | 10782187 | NM_001017505 // Itgbl1 // integrin, beta-like 1 | Itgbl1 | 5.03709e-006 | 1.46921 |
| 269 | 10920754 | ENSRNOT00000039904 // Rbms3 // RNA binding motif, single stranded interacting protein 3 | Rbms3 | 7.26626e-006 | 1.46899 |
| 270 | 10712171 | NM_001106314 // Ifitm1 // interferon induced transmembrane protein 1 | Ifitm1 | 4.19456e-005 | 1.46635 |
| 271 | 10795120 | NM_001191692 // Lrrc16a // leucine rich repeat containing 16A | Lrrc16a | 1.3291e-006 | 1.46577 |
| 272 | 10923052 | NM_032085 // Col3a1 // collagen, type III, alpha 1 | Col3a1 | 1.35234e-005 | 1.46442 |
| 273 | 10705874 | NM_212525 // Tyrobp // Tyro protein tyrosine kinase binding protein | Tyrobp | 8.7029e-006 | 1.46405 |
| 274 | 10867609 | NM_181084 // Tp53inp1 // tumor protein p53 inducible nuclear protein 1 | Tp53inp1 | 6.52488e-008 | 1.46315 |
| 275 | 10724035 | NM_057124 // P2ry6 // pyrimidinergic receptor P2Y, G-protein coupled, 6 | P2ry6 | 3.00251e-005 | 1.46311 |
| 276 | 10862415 | NM_134390 // Tmem176b // transmembrane protein 176B | Tmem176b | 3.51693e-006 | 1.4606 |
| 277 | 10829082 | NM_053502 // Abcg1 // ATP-binding cassette, sub-family G (WHITE), member 1 | Abcg1 | 4.59021e-005 | 1.45875 |
| 278 | 10846854 | NM_199093 // Serping1 // serine (or cysteine) peptidase inhibitor, clade G, member 1 | Serping1 | 2.74598e-006 | 1.4557 |
| 279 | 10769954 | NM_001013231 // Pea15a // phosphoprotein enriched in astrocytes 15A | Pea15a | 1.1969e-005 | 1.4532 |
| 280 | 10851839 | NM_001034927 // Sulf2 // sulfatase 2 | Sulf2 | 1.16224e-005 | 1.45301 |
| 281 | 10820613 | NM_031593 // Sv2c // synaptic vesicle glycoprotein 2c | Sv2c | 0.00136489 | 1.45157 |
| 282 | 10813995 | ENSRNOT00000017372 // Dnah5 // dynein, axonemal, heavy chain 5 | Dnah5 | 0.00392012 | 1.45154 |
| 283 | 10797566 | ENSRNOT00000019473 // S1pr3 // sphingosine-1-phosphate receptor 3 | S1pr3 | 0.00107959 | 1.44992 |
| 284 | 10811452 | NM_001108452 // Cotl1 // coactosin-like 1 (Dictyostelium) | Cotl1 | 2.11738e-005 | 1.44776 |
| 285 | 10872940 | NM_012562 // Fuca1 // fucosidase, alpha-L- 1, tissue | Fuca1 | 2.50304e-007 | 1.44699 |
| 286 | 10772490 | NM_182473 // Corin // corin, serine peptidase | Corin | 3.25897e-005 | 1.44333 |
| 287 | 10719900 | NM_031794 // Axl // Axl receptor tyrosine kinase | Axl | 3.28086e-005 | 1.44172 |
| 288 | 10850025 | NM_001107777 // Siglec1 // sialic acid binding Ig-like lectin 1, sialoadhesin | Siglec1 | 0.00156271 | 1.44139 |
| 289 | 10881253 | NM_001109273 // Tmem51 // transmembrane protein 51 | Tmem51 | 2.5738e-005 | 1.43928 |
| 290 | 10752034 | NM_001107082 // Etv5 // ets variant 5 | Etv5 | 1.20997e-005 | 1.43903 |
| 291 | 10921163 | NM_020542 // Ccr1 // chemokine (C-C motif) receptor 1 | Ccr1 | 0.000378128 | 1.43808 |
| 292 | 10765364 | ENSRNOT00000031367 // Ildr2 // immunoglobulin-like domain containing receptor 2 | Ildr2 | 0.00189937 | 1.43785 |
| 293 | 10829738 | NM_001012076 // Phyhipl // phytanoyl-CoA 2-hydroxylase interacting protein-like | Phyhipl | 0.000777552 | 1.43567 |
| 294 | 10728924 | NM_001006975 // Ms4a6b // membrane-spanning 4-domains, subfamily A, member 6B | Ms4a6b | 0.000298508 | 1.43532 |
| 295 | 10843935 | NM_001109458 // Fam163b // family with sequence similarity 163, member B | Fam163b | 0.000591059 | 1.43484 |
| 296 | 10727260 | NM_171992 // Ccnd1 // cyclin D1 | Ccnd1 | 5.68071e-005 | 1.43478 |
| 297 | 10896541 | NM_030868 // Nov // nephroblastoma overexpressed gene | Nov | 0.000351537 | 1.43374 |
| 298 | 10845859 | NM_030875 // Scn1a // sodium channel, voltage-gated, type I, alpha | Scn1a | 0.00440495 | 1.43356 |
| 299 | 10887622 | NM_001134933 // Crip // cysteine-rich intestinal protein | Crip | 2.17958e-006 | 1.43305 |
| 300 | 10835257 | NM_001105739 // Prrx2 // paired related homeobox 2 | Prrx2 | 5.01462e-005 | 1.43256 |
| 301 | 10768834 | NM_001105992 // Xpr1 // xenotropic and polytropic retrovirus receptor 1 | Xpr1 | 4.8065e-007 | 1.43179 |
| 302 | 10733680 | NM_022525 // Gpx3 // glutathione peroxidase 3 | Gpx3 | 1.14987e-005 | 1.43154 |
| 303 | 10750144 | NM_001100558 // Tiam1 // T-cell lymphoma invasion and metastasis 1 | Tiam1 | 0.000712287 | 1.42985 |
| 304 | 10841579 | NM_001100885 // Myl9 // myosin, light chain 9, regulatory | Myl9 | 0.00136625 | 1.42929 |
| 305 | 10713857 | NM_053445 // Fads1 // fatty acid desaturase 1 | Fads1 | 2.98668e-005 | 1.42752 |
| 306 | 10827916 | NM_013137 // Ddr1 // discoidin domain receptor tyrosine kinase 1 | Ddr1 | 9.7032e-009 | 1.42717 |
| 307 | 10729314 | NM_031776 // Gda // guanine deaminase | Gda | 1.10051e-005 | 1.42699 |
| 308 | 10880404 | NM_053321 // Ptafr // platelet-activating factor receptor | Ptafr | 9.19744e-005 | 1.42682 |
| 309 | 10867329 | NM_001106632 // Mybl1 // myeloblastosis oncogene-like 1 | Mybl1 | 0.000308101 | 1.42678 |
| 310 | 10899644 | NM_001108119 // Nckap1l // NCK associated protein 1 like | Nckap1l | 1.25032e-006 | 1.42644 |
| 311 | 10860499 | NM_001104633 // Sema3d // sema domain, immunoglobulin domain (Ig), short basic d | Sema3d | 0.000504874 | 1.42573 |
| 312 | 10715258 | NM_022587 // Entpd1 // ectonucleoside triphosphate diphosphohydrolase 1 | Entpd1 | 1.37729e-005 | 1.42507 |
| 313 | 10894606 | NM_001108742 // Slc41a2 // solute carrier family 41, member 2 | Slc41a2 | 0.000223507 | 1.42493 |
| 314 | 10903825 | NM_013153 // Has2 // hyaluronan synthase 2 | Has2 | 0.000568088 | 1.42479 |
| 315 | 10818272 | NM_001007012 // Alx3 // aristaless-like homeobox 3 | Alx3 | 0.00480578 | 1.42363 |
| 316 | 10910678 | NM_001108156 // Itga11 // integrin, alpha 11 | Itga11 | 2.58247e-005 | 1.42316 |
| 317 | 10793229 | NM_001007685 // Klhdc8b // kelch domain containing 8B | Klhdc8b | 0.00128591 | 1.42208 |
| 318 | 10728918 | ENSRNOT00000032937 // Ms4a6a // membrane-spanning 4-domains, subfamily A, member 6 | Ms4a6a | 0.00154611 | 1.42168 |
| 319 | 10713833 | NM_134411 // Rab3il1 // RAB3A interacting protein (rabin3)-like 1 | Rab3il1 | 3.54064e-005 | 1.4214 |
| 320 | 10823363 | NM_001002853 // P2ry13 // purinergic receptor P2Y, G-protein coupled, 13 | P2ry13 | 2.49365e-006 | 1.42126 |
| 321 | 10856415 | NM_001013086 // Capg // capping protein (actin filament), gelsolin-like | Capg | 2.48675e-006 | 1.4195 |
| 322 | 10905177 | ENSRNOT00000056266 // Uncharacterized protein | D3zqh2 | 0.00330607 | 1.41911 |
| 323 | 10772857 | ENSRNOT00000050464 // LOC685908 // similar to Discs large homolog 5 | LOC685908 | 5.907e-005 | 1.41895 |
| 324 | 10770759 | NM_001108351 // Sertad4 // SERTA domain containing 4 | Sertad4 | 8.03743e-005 | 1.41875 |
| 325 | 10827079 | NM_053326 // Pdlim5 // PDZ and LIM domain 5 | Pdlim5 | 6.46997e-008 | 1.41825 |
| 326 | 10929177 | NM_001005906 // Chpf // chondroitin polymerizing factor | Chpf | 1.02402e-006 | 1.41802 |
| 327 | 10935555 | NM_001033926 // Fhl1 // four and a half LIM domains 1 | Fhl1 | 6.21346e-005 | 1.41786 |
| 328 | 10877020 | ENSRNOT00000047200 // Svep1 // sushi, von Willebrand factor type A, EGF and pentraxin domain-containing protein 1 | Svep1 | 7.55476e-005 | 1.41773 |
| 329 | 10764460 | NM_198762 // Kcnt2 // potassium channel, subfamily T, member 2 | Kcnt2 | 0.000181731 | 1.41742 |
| 330 | 10897465 | NM_001130577 // Cyth4 // cytohesin 4 | Cyth4 | 5.24536e-005 | 1.41493 |
| 331 | 10769422 | NM_153821 // Prrx1 // paired related homeobox 1 | Prrx1 | 0.000325446 | 1.41479 |
| 332 | 10716480 | NM_030829 // Grk5 // G protein-coupled receptor kinase 5 | Grk5 | 4.83183e-005 | 1.41474 |
| 333 | 10907962 | NM_053559 // Trpc6 // transient receptor potential cation channel, subfamily C | Trpc6 | 0.0001008 | 1.4124 |
| 334 | 10774015 | NM_001100970 // Aebp1 // AE binding protein 1 | Aebp1 | 1.8951e-005 | 1.41087 |
| 335 | 10742402 | NM_053639 // Ltc4s // leukotriene C4 synthase | Ltc4s | 0.000126256 | 1.41053 |
| 336 | 10792304 | NM_024146 // Fgfr1 // Fibroblast growth factor receptor 1 / | Fgfr1 | 8.25921e-008 | 1.40974 |
| 337 | 10825575 | NM_001107708 // Olfml3 // olfactomedin-like 3 | Olfml3 | 4.88153e-006 | 1.40953 |
| 338 | 10934865 | NM_001108243 // Srpx2 // sushi-repeat-containing protein, X-linked 2 | Srpx2 | 0.00017627 | 1.40838 |
| 339 | 10859119 | NM_001134716 // Clec12a // C-type lectin domain family 12, member A | Clec12a | 9.80134e-005 | 1.40837 |
| 340 | 10707597 | NM_001141935 // Atp10a // ATPase, class V, type 10A | Atp10a | 3.46895e-006 | 1.40824 |
| 341 | 10746387 | NM_001024791 // Epn3 // epsin 3 | Epn3 | 0.000121726 | 1.4078 |
| 342 | 10757726 | NM_012722 // Eln // elastin\ | Eln | 1.81801e-005 | 1.40551 |
| 343 | 10865502 | NM_139325 // Eno2 // enolase 2, gamma, neuronal | Eno2 | 7.35153e-006 | 1.40546 |
| 344 | 10714103 | NM_022617 // Mpeg1 // macrophage expressed gene 1 | Mpeg1 | 0.00212338 | 1.40542 |
| 345 | 10812775 | NM_001106405 // Cd180 // CD180 molecule | Cd180 | 0.00420025 | 1.40468 |
| 346 | 10866109 | NM_001009498 // Ly49si2 // immunoreceptor Ly49si2= | Ly49si2 | 0.000846851 | 1.40394 |
| 347 | 10781467 | NM_017254 // Htr2a // 5-hydroxytryptamine (serotonin) receptor 2A | Htr2a | 0.00306897 | 1.40392 |
| 348 | 10715452 | NM_134373 // Avpi1 // arginine vasopressin-induced 1 | Avpi1 | 9.54038e-005 | 1.40188 |
| 349 | 10855062 | NM_053761 // Zyx // zyxin | Zyx | 2.10472e-006 | 1.40154 |
| 350 | 10715431 | NM_134401 // Crtac1 // cartilage acidic protein 1 | Crtac1 | 0.00102157 | 1.40118 |
| 351 | 10817065 | NM_053485 // S100a6 // S100 calcium binding protein A6 | S100a6 | 6.35801e-005 | 1.40077 |
| 352 | 10798610 | NM_053544 // Sfrp4 // secreted frizzled-related protein 4 | Sfrp4 | 0.000380408 | 1.40023 |
| 353 | 10738177 | NM_001014120 // Fkbp10 // FK506 binding protein 10 | Fkbp10 | 5.21321e-005 | 1.39894 |
| 354 | 10923432 | NM_001008527 // Aox3 // aldehyde oxidase 3 | Aox3 | 0.000134223 | 1.3988 |
| 355 | 10874981 | NM_134378 // Sulf1 // sulfatase 1 | Sulf1 | 1.6241e-005 | 1.39791 |
| 356 | 10925636 | NM_017312 // Bok // BCL2-related ovarian killer | Bok | 0.000331792 | 1.39753 |
| 357 | 10721261 | ENSRNOT00000047473 // LOC687856 // similar to Myeloid cell surface antigen CD33 | LOC687856 | 5.21073e-005 | 1.3975 |
| 358 | 10706563 | NM_001009643 // Aspdh // aspartate dehydrogenase domain containing | Aspdh | 0.000413145 | 1.3953 |
| 359 | 10763820 | NM_001109328 // RGD1562617 // similar to RAB7-like protein | RGD1562617 | 0.00106998 | 1.39422 |
| 360 | 10861811 | NM_001108621 // Fam180a // family with sequence similarity 180, member A // 4q22 | Fam180a | 8.04401e-007 | 1.39413 |
| 361 | 10940594 | NM_183330 // cathepsin Z | Ctsz | 1.53001e-007 | 1.39294 |
| 362 | 10709093 | NM_019354 // Ucp2 // uncoupling protein 2 (mitochondrial, proton carrier) | Ucp2 | 0.000655701 | 1.39193 |
| 363 | 10713123 | NM_001191561 // Ltbp3 // latent transforming growth factor beta binding protein | Ltbp3 | 3.63281e-006 | 1.39129 |
| 364 | 10847572 | NM_031797 // Cd82 // Cd82 molecule | Cd82 | 0.000242101 | 1.39063 |
| 365 | 10877251 | NM_198784 // Mup4 // major urinary protein 4 | Mup4 | 0.00229863 | 1.38911 |
| 366 | 10938983 | NM_053641 // Cysltr1 // cysteinyl leukotriene receptor 1 | Cysltr1 | 0.000478437 | 1.38899 |
| 367 | 10861672 | ENSRNOT00000038077 // LOC688956 // hypothetical protein LOC688956 | LOC688956 | 0.00157455 | 1.38876 |
| 368 | 10713682 | NM_001109579 // Lrrn4cl // LRRN4 C-terminal like | Lrrn4cl | 0.00254511 | 1.38746 |
| 369 | 10809540 | NM_031054 // Mmp2 // matrix metallopeptidase 2 | Mmp2 | 7.47691e-007 | 1.3867 |
| 370 | 10797949 | NM_013107 // Bmp6 // bone morphogenetic protein 6 | Bmp6 | 0.000150018 | 1.38582 |
| 371 | 10919041 | NM_001106844 // Fam46a // family with sequence similarity 46, member A | Fam46a | 0.000356045 | 1.38362 |
| 372 | 10779832 | NM_020082 // Rnase4 // ribonuclease, RNase A family 4 | Rnase4 | 4.40335e-006 | 1.38353 |
| 373 | 10860231 | NM_053455 // Fgl2 // fibrinogen-like 2 | Fgl2 | 0.000520151 | 1.38202 |
| 374 | 10728631 | NM_031344 // Fads2 // fatty acid desaturase 2 | Fads2 | 0.000341254 | 1.38141 |
| 375 | 10814119 | NM_001107659 // Sema5a // sema domain, seven thrombospondin repeats (type 1and type 1-like) | Sema5a | 0.000656229 | 1.37997 |
| 376 | 10711268 | NM_012711 // Itgam // integrin, alpha M | Itgam | 0.00204072 | 1.37995 |
| 377 | 10888368 | NM_021767 // Nrxn1 // neurexin 1 | Nrxn1 | 5.83196e-005 | 1.37935 |
| 378 | 10912218 | NM_057194 // Plscr1 // phospholipid scramblase 1 | Plscr1 | 5.33785e-005 | 1.37863 |
| 379 | 10713061 | NM_001005907 // Efemp2 // EGF-containing fibulin-like extracellular matrix protein 2 | Efemp2 | 1.44156e-006 | 1.37786 |
| 380 | 10866970 | NM_012636 // Pthlh // parathyroid hormone-like hormone | Pthlh | 0.000122089 | 1.37775 |
| 381 | 10760196 | NM_019289 // Arpc1b // actin related protein 2/3 complex, subunit 1B | Arpc1b | 5.3419e-006 | 1.37743 |
| 382 | 10876769 | NM_178095 // Abca1 // ATP-binding cassette, sub-family A (ABC1), member 1 | Abca1 | 6.34402e-005 | 1.37694 |
| 383 | 10796770 | NM_001100577 // Apbb1ip // amyloid beta (A4) precursor protein-binding, family B | Apbb1ip | 6.52516e-005 | 1.37664 |
| 384 | 10816725 | ENSRNOT00000027841 // LOC681309 // Uncharacterized protein | LOC681309 | 4.21345e-007 | 1.37664 |
| 385 | 10885680 | NM_001002835 // Smoc1 // SPARC related modular calcium binding 1 | Smoc1 | 4.71424e-005 | 1.37661 |
| 386 | 10805186 | NM_001107376 // Me2 // malic enzyme 2, NAD(+)-dependent, mitochondrial | Me2 | 1.70579e-005 | 1.37617 |
| 387 | 10712477 | NM_001025420 // Lsp1 // lymphocyte-specific protein 1 | Lsp1 | 3.60481e-005 | 1.37615 |
| 388 | 10927251 | NM_001108792 // Bend6 // BEN domain containing 6 | Bend6 | 0.000529048 | 1.37516 |
| 389 | 10718954 | NM_001013894 // Lilrb4 // leukocyte immunoglobulin-like receptor, subfamily B, member 4 | Lilrb4 | 0.000264975 | 1.37501 |
| 390 | 10911287 | NM_019905 // Anxa2 // annexin A2 | Anxa2 | 2.04229e-005 | 1.37475 |
| 391 | 10877262 | NM_147212 // LOC259244 // alpha-2u globulin PGCL3 | LOC259244 | 0.00114931 | 1.37441 |
| 392 | 10856024 | ENSRNOT00000009569 // Prdm5 // PR domain containing 5 | Prdm5 | 6.04184e-005 | 1.37332 |
| 393 | 10933716 | NM_001007667 // Sat1 // spermidine/spermine N1-acetyl transferase 1 | Sat1 | 1.42621e-007 | 1.37307 |
| 394 | 10747633 | NM_001033683 // Vat1 // vesicle amine transport protein 1 homolog (T californica) | Vat1 | 1.49341e-006 | 1.37297 |
| 395 | 10787517 | NM_019216 // Gdf15 // growth differentiation factor 15 | Gdf15 | 0.00037161 | 1.37196 |
| 396 | 10937903 | NM_181366 // Gpr64 // G protein-coupled receptor 64 | Gpr64 | 0.00484983 | 1.37108 |
| 397 | 10772836 | BC079060 // LOC501224 // similar to RIKEN cDNA 2610042L04 | LOC501224 | 0.00217974 | 1.36928 |
| 398 | 10771732 | NM_001012035 // Cdkl2 // cyclin-dependent kinase-like 2 (CDC2-related kinase) | Cdkl2 | 0.000265655 | 1.36924 |
| 399 | 10810631 | NM_001009639 // Tppp3 // tubulin polymerization-promoting protein family member 3 | Tppp3 | 0.000468417 | 1.36825 |
| 400 | 10851724 | NM_019161 // Cdh22 // cadherin 22 | Cdh22 | 0.00018387 | 1.36815 |
| 401 | 10750513 | NM_001002802 // Bace2 // beta-site APP-cleaving enzyme 2 | Bace2 | 5.00092e-007 | 1.3676 |
| 402 | 10897428 | NM_133555 // Csf2rb // colony stimulating factor 2 receptor, beta, low-affinity (granulocyte-macrophage) | Csf2rb | 0.00354521 | 1.36721 |
| 403 | 10872489 | NM_053538 // Laptm5 // lysosomal protein transmembrane 5 | Laptm5 | 4.56664e-005 | 1.36688 |
| 404 | 10895083 | NM_031050 // Lum // lumican | Lum | 0.000138683 | 1.36682 |
| 405 | 10821389 | NM_031750 // Hspb3 // heat shock protein 3 | Hspb3 | 0.000323359 | 1.36659 |
| 406 | 10909639 | NM_022005 // Fxyd6 // FXYD domain-containing ion transport regulator 6 | Fxyd6 | 0.00036234 | 1.36578 |
| 407 | 10899782 | NM_001109484 // Myl6 // myosin, light chain 6, alkali, smooth muscle and non-muscle | Myl6 | 1.00108e-006 | 1.36549 |
| 408 | 10835654 | NM_133569 // Angptl2 // angiopoietin-like 2 | Angptl2 | 9.03563e-005 | 1.36505 |
| 409 | 10908596 | NM_031747 // Cnn1 // calponin 1, basic, smooth muscle | Cnn1 | 0.000497327 | 1.36498 |
| 410 | 10896020 | NM_013082 // Sdc2 // syndecan 2 | Sdc2 | 1.27465e-007 | 1.36293 |
| 411 | 10914618 | NM_053960 // Ccr5 // chemokine (C-C motif) receptor 5 | Ccr5 | 0.00172358 | 1.36235 |
| 412 | 10734382 | NM_017037 // Pmp22 // peripheral myelin protein 22 | Pmp22 | 8.66307e-006 | 1.36209 |
| 413 | 10893373 | NM_001000938 // Olr995 // olfactory receptor 995 | Olr995 | 0.00112228 | 1.36209 |
| 414 | 10868940 | NM_031628 // Nr4a3 // nuclear receptor subfamily 4, group A, member 3 | Nr4a3 | 0.00245282 | 1.36009 |
| 415 | 10817186 | NM_031114 // S100a10 // S100 calcium binding protein A10 | S100a10 | 0.000108062 | 1.35978 |
| 416 | 10940688 | NM_019199 // fibroblast growth factor 18 | Fgf18 | 0.000805341 | 1.35894 |
| 417 | 10798583 | NM_001108415 // Elmo1 // engulfment and cell motility 1 | Elmo1 | 5.78439e-005 | 1.35843 |
| 418 | 10877268 | NM_001033958 // Obp3 // alpha-2u globulin PGCL4 | Obp3 | 0.0023511 | 1.35833 |
| 419 | 10912088 | NM_021576 // Nt5e // 5' nucleotidase, ecto | Nt5e | 3.39393e-008 | 1.35765 |
| 420 | 10874728 | NM_001007002 // Mxra8 // matrix-remodelling associated 8 | Mxra8 | 9.16389e-006 | 1.35739 |
| 421 | 10887538 | NM_001126288 // Pld4 // phospholipase D family, member 4 | Pld4 | 0.000240183 | 1.35658 |
| 422 | 10892332 | ENSRNOT00000051326 // Uncharacterized protein | D3zc00 | 0.000696678 | 1.35653 |
| 423 | 10927562 | NM_001191887 // Aff3 // AF4/FMR2 family, member 3 | Aff3 | 3.51198e-005 | 1.3564 |
| 424 | 10731493 | NM_001105735 // Litaf // lipopolysaccharide-induced TNF factor | Litaf | 1.66709e-005 | 1.35631 |
| 425 | 10928452 | NM_001108798 // Ras association (RalGDS/AF-6) and pleckstrin homology domains 1 | Raph1 | 1.03752e-006 | 1.356 |
| 426 | 10781496 | NM_001012044 // Lcp1 // lymphocyte cytosolic protein 1 | Lcp1 | 0.000972174 | 1.35401 |
| 427 | 10835817 | NM_017043 // Ptgs1 // prostaglandin-endoperoxide synthase 1 | Ptgs1 | 0.000227278 | 1.35399 |
| 428 | 10777116 | NM_001106010 // Gba3 // glucosidase, beta, acid 3 (cytosolic) | Gba3 | 0.00180234 | 1.35396 |
| 429 | 10916493 | NM_053519 // Sorl1 // sortilin-related receptor, LDLR class A repeats-containing | Sorl1 | 0.00403385 | 1.35364 |
| 430 | 10924687 | NM_212526 // Kcne4 // potassium voltage-gated channel, Isk-related subfamily, member 4 | Kcne4 | 0.000116876 | 1.35346 |
| 431 | 10923799 | NM_013121 // Cd28 // Cd28 molecule | Cd28 | 0.000534552 | 1.35306 |
| 432 | 10785063 | NM_031323 // Bmp1 // bone morphogenetic protein 1 | Bmp1 | 2.7309e-005 | 1.35244 |
| 433 | 10856545 | NM_001107866 // Loxl3 // lysyl oxidase-like 3 | Loxl3 | 4.97942e-008 | 1.35199 |
| 434 | 10909874 | NM_019165 // Il18 // interleukin 18 | Il18 | 0.000278557 | 1.35183 |
| 435 | 10905530 | NM_198753 // Rpl3 // ribosomal protein L3 | Rpl3 | 5.21242e-008 | 1.35112 |
| 436 | 10926948 | ENSRNOT00000017592 // LOC100361830 // translocating chain-associating membrane protein | LOC100361830 | 7.69562e-007 | 1.35062 |
| 437 | 10884339 | NM_001163156 // Etv1 // ets variant 1 | Etv1 | 4.54922e-006 | 1.35055 |
| 438 | 10798558 | NM_001191940 // Aoah // acyloxyacyl hydrolase (neutrophil) | Aoah | 1.33906e-005 | 1.35047 |
| 439 | 10921318 | ENSRNOT00000046852 // LOC501091 // similar to Discs large homolog 5 | LOC501091 | 0.00177188 | 1.35028 |
| 440 | 10825809 | NM_012523 // Cd53 // Cd53 molecule | Cd53 | 0.00249078 | 1.35022 |
| 441 | 10919262 | NM_001004249 // Tmed3 // transmembrane emp24 protein transport domain containing | Tmed3 | 1.98172e-005 | 1.34918 |
| 442 | 10907834 | NM_130422 // Casp12 // caspase 12 | Casp12 | 0.000189588 | 1.34881 |
| 443 | 10729390 | NM_001109164 // RGD1560242 // similar to RIKEN cDNA 1700028P14 | RGD1560242 | 0.000436878 | 1.3488 |
| 444 | 10937254 | NM_031084 // Pls3 // plastin 3 (T-isoform) | Pls3 | 1.26434e-005 | 1.34856 |
| 445 | 10818090 | NM_012716 // Slc16a1 // solute carrier family 16, member 1 (monocarboxylic acid) | Slc16a1 | 2.57385e-006 | 1.34824 |
| 446 | 10710647 | NM_012713 // Prkcb // protein kinase C, beta | Prkcb | 2.77835e-005 | 1.34689 |
| 447 | 10898449 | NM_001191991 // Fam19a5 // family with sequence similarity 19 (chemokine (C-C motif)-like), member A5 | Fam19a5 | 2.16053e-005 | 1.34654 |
| 448 | 10861140 | NM_022379 // Tfec // transcription factor EC | Tfec | 0.000170506 | 1.34585 |
| 449 | 10794031 | NM_031024 // Dbn1 // drebrin 1 | Dbn1 | 5.20166e-006 | 1.34487 |
| 450 | 10796564 | NM_001108422 // Plxdc2 // plexin domain containing 2 | Plxdc2 | 7.69333e-006 | 1.3445 |
| 451 | 10861620 | ENSRNOT00000048256 // LOC301748 // similar to RIKEN cDNA 1700001E04 | LOC301748 | 0.00196445 | 1.344 |
| 452 | 10728904 | NM_001106338 // Ms4a7 // membrane-spanning 4-domains, subfamily A, member 7 | Ms4a7 | 0.000326233 | 1.34368 |
| 453 | 10865442 | NM_138900 // C1s // complement component 1, s subcomponent | C1s | 3.45408e-005 | 1.34334 |
| 454 | 10847957 | NM_001109203 // Prrg4 // proline rich Gla (G-carboxyglutamic acid) 4 (transmembrane) | Prrg4 | 0.000564285 | 1.3429 |
| 455 | 10776888 | ENSRNOT00000002883 // Fam114a1 // family with sequence similarity 114, member A1 | Fam114a1 | 0.000131372 | 1.34096 |
| 456 | 10726371 | ENSRNOT00000024974 // Adam12 // ADAM metallopeptidase domain 12 | Adam12 | 0.00377275 | 1.34087 |
| 457 | 10939435 | ENSRNOT00000051516 // LOC679974 // similar to transcription elongation factor A | LOC679974 | 0.00128422 | 1.34015 |
| 458 | 10919226 | XR_146130 // uncharacterized LOC100909889 | LOC100909889 | 0.00169409 | 1.33984 |
| 459 | 10830003 | NM_001007601 // Pcbd1 // pterin-4 alpha-carbinolamine dehydratase/dimerization cofactor of hepatocyte nuclear factor 1 alpha | Pcbd1 | 0.000469096 | 1.33947 |
| 460 | 10936086 | NM_001134599 // Flna // filamin A, alpha | Flna | 2.74584e-005 | 1.33881 |
| 461 | 10780205 | NM_031056 // Mmp14 // matrix metallopeptidase 14 (membrane-inserted) | Mmp14 | 0.00186355 | 1.33807 |
| 462 | 10770070 | NM_001047103 // Cadm3 // cell adhesion molecule 3 | Cadm3 | 2.48943e-005 | 1.33764 |
| 463 | 10861226 | NM_198771 // Fam3c // family with sequence similarity 3, member C | Fam3c | 8.1308e-008 | 1.3367 |
| 464 | 10854645 | NM_012687 // Tbxas1 // thromboxane A synthase 1, platelet | Tbxas1 | 0.00204824 | 1.33668 |
| 465 | 10822583 | NM_001191704 // Fndc3b // fibronectin type III domain containing 3B | Fndc3b | 1.56364e-005 | 1.33546 |
| 466 | 10858886 | ENSRNOT00000026643 // Vwf // von Willebrand factor // 4q42 // 116669 | Vwf | 9.78058e-006 | 1.33454 |
| 467 | 10846340 | NM_001106485 // Fkbp7 // FK506 binding protein 7 | Fkbp7 | 3.79147e-007 | 1.33382 |
| 468 | 10862694 | NM_001109022 // Inmt // indolethylamine N-methyltransferase | Inmt | 0.000610627 | 1.33248 |
| 469 | 10802422 | NM_001025675 // Tubb6 // tubulin, beta 6 | Tubb6 | 0.000303312 | 1.33146 |
| 470 | 10922279 | NM_001109005 // Rab23 // RAB23, member RAS oncogene family | Rab23 | 1.88087e-005 | 1.33142 |
| 471 | 10924853 | NM_001009674 // Itm2c // integral membrane protein 2C | Itm2c | 6.93384e-008 | 1.33086 |
| 472 | 10909338 | NM_173154 // Asam // adipocyte-specific adhesion molecule | Asam | 0.00066793 | 1.33071 |
| 473 | 10870240 | NM_001191867 // Raver2 // ribonucleoprotein, PTB-binding 2 | Raver2 | 4.15852e-005 | 1.33071 |
| 474 | 10749839 | ENSRNOT00000042316 // Vgll3 // vestigial like 3 (Drosophila) | Vgll3 | 0.0033504 | 1.33058 |
| 475 | 10847260 | NM_017269 // Ptprj // protein tyrosine phosphatase, receptor type, J | Ptprj | 2.34194e-006 | 1.33034 |
| 476 | 10770795 | NM_017080 // Hsd11b1 // hydroxysteroid 11-beta dehydrogenase 1 | Hsd11b1 | 1.0962e-006 | 1.33 |
| 477 | 10881211 | NM_001031648 // Efhd2 // EF-hand domain family, member D2= | Efhd2 | 0.000438376 | 1.32917 |
| 478 | 10768979 | NM_031118 // Soat1 // sterol O-acyltransferase 1 | Soat1 | 4.13147e-005 | 1.32859 |
| 479 | 10738591 | NM_172035 // Fzd2 // frizzled homolog 2 (Drosophila) | Fzd2 | 8.93847e-005 | 1.32842 |
| 480 | 10893474 | NM_053542 // Gna15 // guanine nucleotide binding protein, alpha 15 | Gna15 | 0.00225963 | 1.32818 |
| 481 | 10876281 | BC087107 // RGD1311249 // similar to RIKEN cDNA B230312A22 | RGD1311249 | 0.000366873 | 1.32776 |
| 482 | 10877069 | NM_053936 // Lpar1 // lysophosphatidic acid receptor 1 | Lpar1 | 0.00114792 | 1.32765 |
| 483 | 10845681 | NM_138850 // Fap // fibroblast activation protein, alpha | Fap | 0.00197851 | 1.32721 |
| 484 | 10709763 | NM_001106291 // Nlrp10 // NLR family, pyrin domain containing 10 | Nlrp10 | 0.000290354 | 1.32698 |
| 485 | 10712720 | NM_001108513 // Unc93b1 // unc-93 homolog B1 (C. elegans) | Unc93b1 | 6.392e-007 | 1.32693 |
| 486 | 10729693 | NM_053646 // Asah2 // N-acylsphingosine amidohydrolase (non-lysosomal ceramidase 2 | Asah2 | 3.28503e-005 | 1.32675 |
| 487 | 10791893 | NM_001011921 // Pdgfrl // platelet-derived growth factor receptor-like | Pdgfrl | 7.42578e-005 | 1.32595 |
| 488 | 10939430 | NM_001014275 // Tceal8 // transcription elongation factor A (SII)-like 8 | Tceal8 | 4.59543e-006 | 1.32566 |
| 489 | 10781581 | NM_001109912 // Tsc22d1 // TSC22 domain family, member 1 | Tsc22d1 | 1.29448e-009 | 1.32559 |
| 490 | 10876324 | NM_001024345 // Tpm2 // tropomyosin 2, beta | Tpm2 | 0.00107794 | 1.32535 |
| 491 | 10891352 | NM_001037097 // Sptlc2 // serine palmitoyltransferase, long chain base subunit 2 | Sptlc2 | 3.91062e-005 | 1.32383 |
| 492 | 10790963 | ENSRNOT00000031692 // Lrrc25 // leucine rich repeat containing 25 | Lrrc25 | 0.000103966 | 1.32378 |
| 493 | 10918979 | NM_022225 // Htr1b // 5-hydroxytryptamine (serotonin) receptor 1B | Htr1b | 0.000285933 | 1.32342 |
| 494 | 10911145 | NM_001080756 // Car12 // carbonic anyhydrase 12 | Car12 | 0.00135131 | 1.32303 |
| 495 | 10898879 | ENSRNOT00000008159 // Tmem117 // transmembrane protein 117 | Tmem117 | 0.000986524 | 1.32159 |
| 496 | 10830253 | NM_001108933 // Dse // dermatan sulfate epimerase | Dse | 2.72582e-007 | 1.32153 |
| 497 | 10914546 | FQ234406 // TL0AEA59YB17 | TL0AEA59YB17 | 0.0020864 | 1.32093 |
| 498 | 10830957 | NM_001008849 // RT1-M1-2 // RT1 class I, locus M1, gene 2 | RT1-M1-2 | 0.00221221 | 1.32051 |
| 499 | 10703680 | NM_001037357 // Lilrb3l // leukocyte immunoglobulin-like receptor, subfamily B, member 3-like | Lilrb3l | 0.000130093 | 1.32022 |
| 500 | 10905773 | ENSRNOT00000037681 // Nfam1 // NFAT activating protein with ITAM motif | Nfam1 | 0.000284461 | 1.32002 |
| 501 | 10734673 | NM_053484 // Gas7 // growth arrest specific 7 | Gas7 | 0.000156674 | 1.31987 |
| 502 | 10916016 | NM_203337 // St3gal4 // ST3 beta-galactoside alpha-2,3-sialyltransferase 4 | St3gal4 | 1.33827e-005 | 1.3197 |
| 503 | 10761033 | ENSRNOT00000046100 // RGD1566006 // similar to paired immunoglobin-like type 2 receptor | RGD1566006 | 0.000314191 | 1.31949 |
| 504 | 10764228 | NM_001012206 // Phlda3 // pleckstrin homology-like domain, family A, member 3 | Phlda3 | 0.000951132 | 1.31916 |
| 505 | 10768668 | NM_053966 // Lamc1 // laminin, gamma 1 | Lamc1 | 5.06972e-007 | 1.31875 |
| 506 | 10744766 | NM_024399 // Aspa // aspartoacylase | Aspa | 7.67792e-005 | 1.31856 |
| 507 | 10808274 | NM_138889 // Cdh13 // cadherin 13 | Cdh13 | 3.74682e-005 | 1.3174 |
| 508 | 10859660 | NM_001083336 // Stk38l // serine/threonine kinase 38 like | Stk38l | 7.42879e-007 | 1.31728 |
| 509 | 10855549 | NM_001013085 // Snx10 // sorting nexin 10 | Snx10 | 0.0033093 | 1.31717 |
| 510 | 10805527 | NM_001007687 // Cndp1 // carnosine dipeptidase 1 (metallopeptidase M20 family) | Cndp1 | 0.000867349 | 1.31667 |
| 511 | 10738972 | NM_001024687 // Mrc2 // mannose receptor, C type 2 | Mrc2 | 9.06152e-005 | 1.31646 |
| 512 | 10935811 | NM_023091 // Gabre // gamma-aminobutyric acid (GABA) A receptor, epsilon | Gabre | 0.00472712 | 1.31635 |
| 513 | 10853407 | BC061963 // MGC72627 // similar to RIKEN cDNA A330021E22 | MGC72627 | 0.00236708 | 1.31603 |
| 514 | 10791750 | NM_053770 // Sorbs2 // sorbin and SH3 domain containing 2 | Sorbs2 | 5.92737e-005 | 1.31591 |
| 515 | 10918288 | ENSRNOT00000042633 // Plekho2 // pleckstrin homology domain containing, family O | Plekho2 | 0.000266847 | 1.31478 |
| 516 | 10824548 | ENSRNOT00000056606 // LOC685152 // similar to Probable phospholipid-transporting | LOC685152 | 7.1125e-005 | 1.31462 |
| 517 | 10861890 | NM_001012188 // Creb3l2 // cAMP responsive element binding protein 3-like 2 | Creb3l2 | 0.000135212 | 1.31459 |
| 518 | 10781131 | NM_001135855 // Scara5 // scavenger receptor class A, member 5 (putative) | Scara5 | 0.000501534 | 1.31446 |
| 519 | 10804821 | XM_003754442 // piezo-type mechanosensitive ion channel component 2, transcript variant 2 | Piezo2 | 6.40222e-006 | 1.31445 |
| 520 | 10729777 | NM_001025415 // Ch25h // cholesterol 25-hydroxylase | Ch25h | 0.00443863 | 1.31378 |
| 521 | 10851364 | ENSRNOT00000019453 // RGD1563354 // similar to hypothetical protein D630003M21 | RGD1563354 | 9.6965e-005 | 1.31327 |
| 522 | 10785897 | ENSRNOT00000042031 // Uncharacterized protein | D3z8c0 | 0.000355018 | 1.31281 |
| 523 | 10876831 | ENSRNOT00000047225 // Zfp462 // zinc finger protein 462 | Zfp462 | 0.000396636 | 1.31232 |
| 524 | 10705213 | NM_021578 // Tgfb1 // transforming growth factor, beta 1 | Tgfb1 | 1.79043e-005 | 1.31202 |
| 525 | 10802734 | NM_030858 // Smad7 // SMAD family member 7 | Smad7 | 0.00454694 | 1.31117 |
| 526 | 10926277 | NM_001106885 // Trem1 // triggering receptor expressed on myeloid cells 1 | Trem1 | 0.00326612 | 1.31094 |
| 527 | 10807601 | NM_001168674 // Sntb2 // syntrophin, beta 2 | Sntb2 | 4.70919e-005 | 1.31052 |
| 528 | 10882168 | NM_053606 // Mmp23 // matrix metallopeptidase 23 | Mmp23 | 0.000132951 | 1.31047 |
| 529 | 10921899 | NM_053470 // Runx2 // runt-related transcription factor 2 | Runx2 | 1.95348e-006 | 1.30982 |
| 530 | 10799968 | NM_001106127 // Prtfdc1 // phosphoribosyl transferase domain containing 1 | Prtfdc1 | 8.14058e-007 | 1.3098 |
| 531 | 10922113 | NM_001014099 // Paqr8 // progestin and adipoQ receptor family member VIII | Paqr8 | 7.48964e-005 | 1.30975 |
| 532 | 10853020 | NM_080394 // Reln // reelin | Reln | 0.000202246 | 1.30906 |
| 533 | 10866030 | NM_133306 // Olr1 // oxidized low density lipoprotein (lectin-like) receptor 1 | Olr1 | 0.00109043 | 1.30898 |
| 534 | 10714353 | NM_001107596 // Tmem2 // transmembrane protein 2 | Tmem2 | 2.149e-005 | 1.30845 |
| 535 | 10861358 | NM_057201 // Gpr37 // G protein-coupled receptor 37 | Gpr37 | 6.68633e-005 | 1.30784 |
| 536 | 10711364 | NM_001191840 // Tgfb1i1 // transforming growth factor beta 1 induced transcript | Tgfb1i1 | 0.00118498 | 1.30778 |
| 537 | 10847761 | NM_012924 // Cd44 // Cd44 molecule | Cd44 | 0.000996994 | 1.30748 |
| 538 | 10766428 | NM_001145828 // Tlr5 // toll-like receptor 5 | Tlr5 | 0.00176108 | 1.30671 |
| 539 | 10896028 | NM_031640 // Pgcp // plasma glutamate carboxypeptidase | Pgcp | 4.61608e-005 | 1.30666 |
| 540 | 10921041 | ENSRNOT00000014323 // Fam198a // family with sequence similarity 198, member A | Fam198a | 0.000114124 | 1.30662 |
| 541 | 10829378 | NM_053489 // Col18a1 // collagen, type XVIII, alpha 1 | Col18a1 | 7.6146e-005 | 1.3063 |
| 542 | 10936048 | NM_017006 // G6pd // glucose-6-phosphate dehydrogenase | G6pd | 2.47799e-005 | 1.30601 |
| 543 | 10833806 | ENSRNOT00000031047 // Armc2 // armadillo repeat containing 2 | Armc2 | 0.00221831 | 1.30504 |
| 544 | 10781999 | ENSRNOT00000050826 // LOC691984 // similar to Glypican-6 precursor | LOC691984 | 2.2467e-005 | 1.30417 |
| 545 | 10738576 | NM_017113 // Grn // granulin | Grn | 2.10087e-006 | 1.30387 |
| 546 | 10908037 | NM_001108125 // Sesn3 // sestrin 3 | Sesn3 | 9.77976e-005 | 1.30387 |
| 547 | 10821698 | NM_001005384 // Osmr // oncostatin M receptor | Osmr | 0.000350658 | 1.30374 |
| 548 | 10836212 | ENSRNOT00000006433 // RGD1560248 // similar to formin-like 2 isoform B | RGD1560248 | 1.49234e-005 | 1.30351 |
| 549 | 10853347 | NM_012690 // Abcb4 // ATP-binding cassette, sub-family B (MDR/TAP), member 4 | Abcb4 | 3.12115e-006 | 1.30291 |
| 550 | 10821367 | NM_001106411 // Gpx8 // glutathione peroxidase 8 // 2q14 // 294744 /// ENSRNOT00 | Gpx8 | 0.00441934 | 1.30263 |
| 551 | 10746139 | NM_133383 // Scpep1 // serine carboxypeptidase 1 | Scpep1 | 1.7019e-005 | 1.30053 |
| 552 | 10710067 | NM_172067 // Spon1 // spondin 1, extracellular matrix protein | Spon1 | 0.000603473 | 1.30047 |
| 553 | 10867008 | XM_003754442 // hypothetical protein LOC100361596 | LOC100361596 | 2.54293e-005 | 1.3003 |
| 554 | 10729802 | NM_001106373 // Pank1 // pantothenate kinase 1 | Pank1 | 0.000459566 | -1.30009 |
| 555 | 10867165 | NM_001024247 // Lactb2 // lactamase, beta 2 | Lactb2 | 6.00288e-005 | -1.30134 |
| 556 | 10907738 | NM_022676 // Ppp1r1a // protein phosphatase 1, regulatory (inhibitor) subunit 1A | Ppp1r1a | 0.00016961 | -1.30142 |
| 557 | 10706221 | ENSRNOT00000020079 // LOC690000 // similar to CG3740-PA | LOC690000 | 0.000292202 | -1.30162 |
| 558 | 10897272 | ENSRNOT00000056375 // Uncharacterized protein | F1ly58 | 8.26073e-006 | -1.30299 |
| 559 | 10840783 | NM_001037198 // RGD1304644 // similar to RIKEN cDNA 2310046K01 | RGD1304644 | 0.00351023 | -1.30415 |
| 560 | 10927792 | NM_001024787 // Osgepl1 // O-sialoglycoprotein endopeptidase-like 1 | Osgepl1 | 7.2265e-005 | -1.30417 |
| 561 | 10762378 | NM_181638 // Tbx3 // T-box 3 | Tbx3 | 0.00297446 | -1.30443 |
| 562 | 10742645 | NM_019269 // Slc22a5 // solute carrier family 22 (organic cation/carnitine transporter) | Slc22a5 | 0.000104185 | -1.30451 |
| 563 | 10870490 | ENSRNOT00000029228 // Usp24 // ubiquitin specific protease 24 | Usp24 | 0.000986597 | -1.30518 |
| 564 | 10903925 | BC129127 // RGD1563224 // similar to 4930438D12Rik protein | RGD1563224 | 0.000163027 | -1.30531 |
| 565 | 10795108 | NM_001106111 // Acot13 // acyl-CoA thioesterase 13 | Acot13 | 5.42117e-005 | -1.3059 |
| 566 | 10889074 | NM_133618 // Hadhb // hydroxyacyl-Coenzyme A dehydrogenase/3-ketoacyl-Coenzyme A | Hadhb | 3.45216e-006 | -1.30666 |
| 567 | 10786727 | NM_001107295 // Oxnad1 // oxidoreductase NAD-binding domain containing 1 | Oxnad1 | 9.56621e-006 | -1.3074 |
| 568 | 10940517 | NM_001077231 // hypothetical protein LOC686179 | MGC125239 | 3.86662e-005 | -1.30834 |
| 569 | 10759402 | NM_031587 // Pxmp2 // peroxisomal membrane protein 2 | Pxmp2 | 0.000120215 | -1.30845 |
| 570 | 10833837 | NM_001014034 // Qrsl1 // glutaminyl-tRNA synthase (glutamine-hydrolyzing)-like 1 | Qrsl1 | 7.85048e-005 | -1.30851 |
| 571 | 10894660 | NM_001134887 // Nt5dc3 // 5'-nucleotidase domain containing 3 | Nt5dc3 | 1.16369e-005 | -1.30979 |
| 572 | 10784454 | NM_019238 // Fdft1 // farnesyl diphosphate farnesyl transferase 1 | Fdft1 | 1.00663e-005 | -1.31082 |
| 573 | 10816394 | NM_001007637 // mrpl24 // mitochondrial ribosomal protein L24 | mrpl24 | 1.0615e-005 | -1.31094 |
| 574 | 10706884 | NM_022400 // Bcat2 // branched chain aminotransferase 2, mitochondrial | Bcat2 | 6.24117e-007 | -1.31116 |
| 575 | 10829826 | ENSRNOT00000047773 // RGD1566373 // similar to large subunit ribosomal protein L | RGD1566373 | 0.00321995 | -1.31169 |
| 576 | 10714700 | NM_001107582 // Pdcd1lg2 // programmed cell death 1 ligand 2 | Pdcd1lg2 | 0.000319876 | -1.31177 |
| 577 | 10751698 | XM_002724732 // LOC100359995 // rCG63619-like | LOC100359995 | 1.46368e-006 | -1.312 |
| 578 | 10847509 | NM_053777 // Mapk8ip1 // mitogen-activated protein kinase 8 interacting protein | Mapk8ip1 | 2.09238e-006 | -1.31201 |
| 579 | 10705202 | NM_001107493 // Exosc5 // exosome component 5 | Exosc5 | 2.12314e-005 | -1.31205 |
| 580 | 10724578 | AY228140 // Egln1 // EGL nine homolog 1 (C. elegans) | Egln1 | 0.00107734 | -1.31259 |
| 581 | 10770252 | NM_001008293 // Tfb2m // transcription factor B2, mitochondrial | Tfb2m | 3.53599e-005 | -1.31302 |
| 582 | 10887481 | ENSRNOT00000017504 // LOC691485 // hypothetical protein LOC691485 | LOC691485 | 0.000101504 | -1.31339 |
| 583 | 10817547 | NM_001012080 // Hfe2 // hemochromatosis type 2 (juvenile) homolog (human) | Hfe2 | 1.99146e-006 | -1.31453 |
| 584 | 10780393 | NM_001106037 // Fitm1 // fat storage-inducing transmembrane protein 1 | Fitm1 | 6.2453e-006 | -1.31463 |
| 585 | 10855035 | NM_013147 // Clcn1 // chloride channel 1 | Clcn1 | 0.000297776 | -1.31469 |
| 586 | 10814628 | ENSRNOT00000037367 // Similar to Traf2 and NCK interacting kinase, splice variant 4 | D3ze61 | 0.000301526 | -1.31501 |
| 587 | 10890342 | NM_001108028 // L2hgdh // L-2-hydroxyglutarate dehydrogenase | L2hgdh | 4.75164e-005 | -1.31526 |
| 588 | 10795384 | NM_001007625 // Epdr1 // ependymin related protein 1 (zebrafish) | Epdr1 | 0.000117486 | -1.31635 |
| 589 | 10874298 | NM_013214 // Acot7 // acyl-CoA thioesterase 7 | Acot7 | 1.47763e-005 | -1.31656 |
| 590 | 10878797 | NM_016999 // Cyp4b1 // cytochrome P450, family 4, subfamily b, polypeptide 1 | Cyp4b1 | 0.000529089 | -1.31705 |
| 591 | 10884408 | ENSRNOT00000041624 // LOC100359529 // rCG61688-like | LOC100359529 | 0.000122892 | -1.31745 |
| 592 | 10874860 | NM_001108001 // RGD1304931 // similar to RIKEN cDNA 2310042D19 | RGD1304931 | 0.000119056 | -1.31788 |
| 593 | 10837068 | NM_001107735 // Osbpl6 // oxysterol binding protein-like 6 | Osbpl6 | 0.000113735 | -1.31972 |
| 594 | 10871806 | NM_001107979 // Fhl3 // four and a half LIM domains 3 | Fhl3 | 0.000955563 | -1.32024 |
| 595 | 10732644 | NM_022230 // Stc2 // stanniocalcin 2 | Stc2 | 0.000612869 | -1.32076 |
| 596 | 10830030 | NM_001109171 // Lrrc20 // leucine rich repeat containing 20 | Lrrc20 | 2.38875e-006 | -1.321 |
| 597 | 10850576 | ENSRNOT00000055530 // Uncharacterized protein | D4Ad87 | 0.000156568 | -1.32141 |
| 598 | 10762108 | NM_001159625 // Tmem116 // transmembrane protein 116 | Tmem116 | 7.74707e-005 | -1.3215 |
| 599 | 10735940 | NM_001105811 // Rilp // Rab interacting lysosomal protein | Rilp | 0.000118726 | -1.32251 |
| 600 | 10707721 | NM_001106341 // Mcee // methylmalonyl CoA epimerase | Mcee | 0.000119064 | -1.32278 |
| 601 | 10706994 | NM_001107513 // Tmem143 // transmembrane protein 143 | Tmem143 | 1.33423e-008 | -1.32306 |
| 602 | 10716226 | NM_022260 // Casp7 // caspase 7 | Casp7 | 4.50177e-005 | -1.32346 |
| 603 | 10805254 | NM_001107375 // Pex19 // peroxisomal biogenesis factor 19 | Pex19 | 3.59297e-006 | -1.32376 |
| 604 | 10785031 | NM_001107276 // Piwil2 // piwi-like 2 (Drosophila) | Piwil2 | 0.00166384 | -1.32385 |
| 605 | 10820824 | NM_001107648 // Ptcd2 // pentatricopeptide repeat domain 2 | Ptcd2 | 6.13031e-005 | -1.32386 |
| 606 | 10806650 | NM_001013190 // Rad23a // RAD23 homolog A (S. cerevisiae) | Rad23a | 1.54083e-006 | -1.324 |
| 607 | 10863418 | NM_013177 // glutamic-oxaloacetic transaminase 2, mitochondrial (aspartate aminotransferase 2) | Got2 | 0.00264294 | -1.32428 |
| 608 | 10757409 | NM_001044284 // Tsc22d4 // TSC22 domain family, member 4 | Tsc22d4 | 3.81665e-006 | -1.32593 |
| 609 | 10926232 | NM_001134596 // RGD1308299 // similar to chromosome 6 open reading frame 130 | RGD1308299 | 2.86926e-006 | -1.32769 |
| 610 | 10878564 | NM_012930 // Cpt2 // carnitine palmitoyltransferase 2 | Cpt2 | 3.16991e-008 | -1.32808 |
| 611 | 10826474 | NM_012804 // Abcd3 // ATP-binding cassette, sub-family D (ALD), member 3 | Abcd3 | 0.000129717 | -1.32813 |
| 612 | 10852244 | NM_001109564 // Ppp1r3d // protein phosphatase 1, regulatory subunit 3D | Ppp1r3d | 0.000531726 | -1.32841 |
| 613 | 10895391 | ENSRNOT00000008623 // Uncharacterized protein | D3z9e0 | 3.52226e-005 | -1.32849 |
| 614 | 10759034 | NM_022512 // Acads // acyl-Coenzyme A dehydrogenase, C-2 to C-3 short chain | Acads | 1.76385e-006 | -1.3298 |
| 615 | 10871117 | NM_001107959 // Atpaf1 // ATP synthase mitochondrial F1 complex assembly factor 1 | Atpaf1 | 1.58234e-005 | -1.32996 |
| 616 | 10766037 | NM_022678 // Zfp238 // zinc finger protein 238 | Zfp238 | 0.00010055 | -1.33028 |
| 617 | 10814105 | NM_001008770 // Cmbl // carboxymethylenebutenolidase homolog (Pseudomonas) | Cmbl | 1.73152e-005 | -1.33093 |
| 618 | 10859392 | NM_134349 // Mgst1 // microsomal glutathione S-transferase 1 | Mgst1 | 0.00246291 | -1.33155 |
| 619 | 10856436 | NM_053752 // Suclg1 // succinate-CoA ligase, alpha subunit | Suclg1 | 5.99146e-006 | -1.33219 |
| 620 | 10902868 | NM_017302 // Slc16a7 // solute carrier family 16, member 7 (monocarboxylic acid transporter 2) | Slc16a7 | 0.0025601 | -1.3355 |
| 621 | 10805996 | NM_022533 // Pllp // plasma membrane proteolipid (plasmolipin) | Pllp | 0.00473462 | -1.33553 |
| 622 | 10897239 | NM_001103364 // LOC100125385 // hypothetical protein LOC100125385 | LOC100125385 | 2.0091e-006 | -1.3356 |
| 623 | 10718556 | NM_080694 // Cacng6 // calcium channel, voltage-dependent, gamma subunit 6 | Cacng6 | 0.00349506 | -1.33737 |
| 624 | 10848362 | NM_001014181 // Atpbd4 // ATP binding domain 4 | Atpbd4 | 9.435e-006 | -1.33793 |
| 625 | 10791331 | ENSRNOT00000047993 // Uncharacterized protein | D3z7z9 | 0.000618714 | -1.3382 |
| 626 | 10777305 | XM_003754442 // developmental pluripotency-associated protein 3-like | LOC679766 | 0.00300368 | -1.33845 |
| 627 | 10741244 | NM_001024991 // Fahd1 // fumarylacetoacetate hydrolase domain containing 1 | Fahd1 | 1.0897e-005 | -1.33861 |
| 628 | 10788028 | ENSRNOT00000048192 // Uncharacterized protein | D4A8q5 | 0.00242274 | -1.33945 |
| 629 | 10807632 | NM_030586 // Cyb5b // cytochrome b5 type B (outer mitochondrial membrane) | Cyb5b | 4.51717e-006 | -1.34146 |
| 630 | 10896551 | ENSRNOT00000005722 // Depdc6 // DEP domain containing 6 | Depdc6 | 6.66187e-006 | -1.34232 |
| 631 | 10902290 | NM_001191782 // Nav3 // neuron navigator 3 | Nav3 | 0.00130885 | -1.34275 |
| 632 | 10932917 | NM_012621 // Pfkfb1 // 6-phosphofructo-2-kinase/fructose-2,6-biphosphatase 1 | Pfkfb1 | 0.00265283 | -1.34276 |
| 633 | 10910936 | NM_001007803 // Clpx // ClpX caseinolytic peptidase X homolog (E. coli) | Clpx | 9.44262e-007 | -1.34365 |
| 634 | 10894808 | ENSRNOT00000064242 // Anks1b // ankyrin repeat and sterile alpha motif domain containing protein 1B | Anks1b | 0.00129193 | -1.34399 |
| 635 | 10770831 | NM_019190 // Cd46 // CD46 molecule, complement regulatory protein | Cd46 | 5.37882e-007 | -1.34433 |
| 636 | 10714264 | BC091268 // RGD1311863 // similar to RIKEN cDNA 2410127L17 | RGD1311863 | 1.58144e-005 | -1.34438 |
| 637 | 10745757 | NM_001014119 // Dhrs11 // dehydrogenase/reductase (SDR family) member 11 | Dhrs11 | 6.31081e-006 | -1.34731 |
| 638 | 10899756 | NM_001108727 // Coq10a // coenzyme Q10 homolog A (S. cerevisiae) | Coq10a | 4.14165e-005 | -1.34752 |
| 639 | 10812722 | NM_001108543 // Mrps27 // mitochondrial ribosomal protein S27 | Mrps27 | 4.40031e-007 | -1.34808 |
| 640 | 10716995 | NM_001037789 // Ccdc28a // coiled-coil domain containing 28A | Ccdc28a | 0.000659959 | -1.34818 |
| 641 | 10906142 | NM_021746 // Mapk12 // mitogen-activated protein kinase 12 | Mapk12 | 2.80839e-005 | -1.34869 |
| 642 | 10789143 | NM_001047880 // Slc25a15 // solute carrier family 25 (mitochondrial carrier; ornithine transporter) member 15 | Slc25a15 | 2.4181e-005 | -1.3491 |
| 643 | 10719829 | NM_012859 // Lipe // lipase, hormone sensitive | Lipe | 3.18791e-006 | -1.34971 |
| 644 | 10776114 | ENSRNOT00000002667 // Vcsa2 // variable coding sequence A2 | Vcsa2 | 0.00363857 | -1.34987 |
| 645 | 10801929 | NM_001014242 // Isoc1 // isochorismatase domain containing 1 | Isoc1 | 0.000588873 | -1.35019 |
| 646 | 10808553 | ENSRNOT00000020313 // Acsf3 // acyl-CoA synthetase family member 3 | Acsf3 | 1.99612e-005 | -1.35051 |
| 647 | 10786312 | NM_001170749 // Asb14 // ankyrin repeat and SOCS box-containing 14 | Asb14 | 2.02405e-005 | -1.35176 |
| 648 | 10754857 | ENSRNOT00000029572 // RGD1562500 // similar to RIKEN cDNA 2310010M20 | RGD1562500 | 0.00144664 | -1.35225 |
| 649 | 10818606 | NM_001109637 // Lrrc39 // leucine rich repeat containing 39 | Lrrc39 | 7.32316e-007 | -1.35342 |
| 650 | 10734349 | NM_001135033 // Trim16 // tripartite motif-containing 16 | Trim16 | 5.3822e-006 | -1.35497 |
| 651 | 10799158 | NM_001107360 // Dip2c // DIP2 disco-interacting protein 2 homolog C (Drosophila) | Dip2c | 1.84987e-006 | -1.35638 |
| 652 | 10820206 | NM_001109468 // Polr3g // polymerase (RNA) III (DNA directed) polypeptide G | Polr3g | 0.000391432 | -1.35653 |
| 653 | 10870762 | NM_001106675 // Echdc2 // enoyl Coenzyme A hydratase domain containing 2 | Echdc2 | 9.43122e-006 | -1.35825 |
| 654 | 10936520 | NM_001108059 // Elk1 // ELK1, member of ETS oncogene family | Elk1 | 6.68165e-007 | -1.35869 |
| 655 | 10940645 | NM_012936 // crystallin, beta B | Crybb1 | 3.32945e-005 | -1.3593 |
| 656 | 10789470 | ENSRNOT00000026370 // Dcun1d2 // DCN1, defective in cullin neddylation 1, domain | Dcun1d2 | 8.8444e-006 | -1.36128 |
| 657 | 10836271 | NM_031610 // Kcnj3 // potassium inwardly-rectifying channel, subfamily J, memberer 3 | Kcnj3 | 9.15922e-006 | -1.36194 |
| 658 | 10896181 | NM_001012116 // Spag1 // sperm associated antigen 1 | Spag1 | 0.00343236 | -1.36479 |
| 659 | 10854417 | NM_001013084 // Akr1b10 // aldo-keto reductase family 1, member B10 (aldose reductase) | Akr1b10 | 0.000138392 | -1.36487 |
| 660 | 10794769 | NM_001006966 // Peci // peroxisomal D3,D2-enoyl-CoA isomerase | Peci | 9.78627e-006 | -1.3658 |
| 661 | 10853637 | NM_001024318 // Asb4 // ankyrin repeat and SOCS box-containing 4 | Asb4 | 7.14266e-005 | -1.36776 |
| 662 | 10869037 | ENSRNOT00000032726 // RGD1562799 // similar to Cylicin-2 (Cylicin II) | RGD1562799 | 0.00104164 | -1.36844 |
| 663 | 10764972 | NM_001162897 // LOC100302372 // hypothetical protein LOC100302372 | LOC100302372 | 8.71574e-005 | -1.37079 |
| 664 | 10872978 | NM_001015008 // Tcea3 // transcription elongation factor A (SII), 3 | Tcea3 | 4.1643e-006 | -1.37117 |
| 665 | 10870261 | NM_017135 // Ak3l1 // adenylate kinase 3-like 1 | Ak3l1 | 0.000236181 | -1.37149 |
| 666 | 10793218 | NM_001014005 // Tcta // T-cell leukemia translocation altered gene | Tcta | 3.92273e-005 | -1.373 |
| 667 | 10843838 | NM_001107821 // Agpat2 // 1-acylglycerol-3-phosphate O-acyltransferase 2 (lysophosphatidic acid acyltransferase, beta) | Agpat2 | 0.000144764 | -1.37352 |
| 668 | 10872283 | ENSRNOT00000047152 // Fndc5 // fibronectin type III domain containing | Fndc5 | 1.84623e-006 | -1.37359 |
| 669 | 10919897 | NM_001005383 // Acy1 // aminoacylase 1 | Acy1 | 1.48876e-005 | -1.37419 |
| 670 | 10914424 | NM_001106868 // Hhatl // hedgehog acyltransferase-like | Hhatl | 1.28319e-005 | -1.37425 |
| 671 | 10749172 | NM_017340 // Acox1 // acyl-Coenzyme A oxidase 1, palmitoyl | Acox1 | 9.21393e-008 | -1.37638 |
| 672 | 10796027 | NM_001008342 // Akr1cl2 // aldo-keto reductase family 1, member C-like 2 | Akr1cl2 | 2.33776e-005 | -1.37648 |
| 673 | 10889269 | NM_001012111 // Lpin1 // lipin 1 | Lpin1 | 1.00676e-005 | -1.37805 |
| 674 | 10862818 | ENSRNOT00000039316 // RGD1565690 // similar to mKIAA2027 protein | RGD1565690 | 0.000420757 | -1.37936 |
| 675 | 10779002 | NM_001162408 // Dusp13 // dual specificity phosphatase 13 | Dusp13 | 0.000259211 | -1.3806 |
| 676 | 10822929 | NM_181363 // Nudt6 // nudix (nucleoside diphosphate linked moiety X)-type motif 6 | Nudt6 | 0.000226877 | -1.38081 |
| 677 | 10780289 | NM_153315 // Dhrs4 // dehydrogenase/reductase (SDR family) member 4 | Dhrs4 | 5.18442e-007 | -1.38105 |
| 678 | 10740567 | NM_001106974 // A2bp1 // ataxin 2 binding protein 1 | A2bp1 | 9.1593e-006 | -1.38193 |
| 679 | 10850140 | NM_198779 // Gpcpd1 // glycerophosphocholine phosphodiesterase GDE1 homolog (S. cerevisiae) | Gpcpd1 | 0.00472812 | -1.38332 |
| 680 | 10853450 | NM_001100813 // Cldn12 // claudin 12 | Cldn12 | 0.000218502 | -1.38509 |
| 681 | 10732197 | NM_001191589 // Rpl3l // ribosomal protein L3-like | Rpl3l | 4.70137e-006 | -1.38517 |
| 682 | 10853995 | BC097405 // Asb15 // ankyrin repeat and SOCS box-containing protein 15 | Asb15 | 7.98753e-006 | -1.38754 |
| 683 | 10836877 | NM_053826 // Pdk1 // pyruvate dehydrogenase kinase, isozyme 1 | Pdk1 | 2.03959e-007 | -1.3881 |
| 684 | 10765720 | NM_001107375 // Pex19 // peroxisomal biogenesis factor 19 | Pex19 | 2.11828e-006 | -1.38948 |
| 685 | 10838255 | NM_001144862 // LOC691083 // hypothetical protein LOC691083 | LOC691083 | 2.12934e-006 | -1.38951 |
| 686 | 10902112 | NM_001108091 // RGD1307051 // similar to hypothetical protein FLJ21963 | RGD1307051 | 0.00182145 | -1.38967 |
| 687 | 10940564 | NM_133552 // MLX interacting protein-like | Mlxipl | 6.89273e-005 | -1.38992 |
| 688 | 10744348 | NM_012891 // Acadvl // acyl-Coenzyme A dehydrogenase, very long chain | Acadvl | 2.08208e-007 | -1.3905 |
| 689 | 10874118 | NM_001012107 // Gpr157 // G protein-coupled receptor 157 | Gpr157 | 0.000187505 | -1.3913 |
| 690 | 10857984 | NM_001145367 // Pparg // peroxisome proliferator-activated receptor gamma | Pparg | 0.000139645 | -1.39408 |
| 691 | 10802650 | NM_017083 // Myo5b // myosin Vb | Myo5b | 0.00194534 | -1.39802 |
| 692 | 10798624 | ENSRNOT00000046953 // LOC681290 // Tcrg-C protein-like | LOC681290 | 0.00200327 | -1.401 |
| 693 | 10883303 | NM_130826 // Hadha // hydroxyacyl-Coenzyme A dehydrogenase/3-ketoacyl-Coenzyme A | Hadha | 1.00372e-005 | -1.4036 |
| 694 | 10890297 | FQ227960 // TL0AEA11YB07 | TL0AEA11YB07 | 0.000830855 | -1.40497 |
| 695 | 10826828 | NM_057186 // Hadh // hydroxyacyl-Coenzyme A dehydrogenase | Hadh | 7.82405e-008 | -1.40548 |
| 696 | 10756411 | NM_001024968 // Slc46a3 // solute carrier family 46, member 3 | Slc46a3 | 0.000276748 | -1.40726 |
| 697 | 10844183 | NM_001004085 // Crat // carnitine acetyltransferase | Crat | 2.50501e-008 | -1.41023 |
| 698 | 10758697 | NM_020078 // Adam1a // a disintegrin and metallopeptidase domain 1a | Adam1a | 2.24423e-005 | -1.4115 |
| 699 | 10762792 | NM_053922 // Acacb // acetyl-Coenzyme A carboxylase beta | Acacb | 1.59867e-006 | -1.41419 |
| 700 | 10781638 | ENSRNOT00000013780 // RGD1308772 // similar to KIAA0564 protein | RGD1308772 | 9.56718e-006 | -1.41423 |
| 701 | 10733049 | ENSRNOT00000003339 // Trim7 // tripartite motif-containing 7 | Trim7 | 0.000346208 | -1.41423 |
| 702 | 10787611 | NM_001127590 // Slc25a42 // solute carrier family 25, member 42 | Slc25a42 | 2.63409e-005 | -1.41434 |
| 703 | 10913218 | NM_053965 // Slc25a20 // solute carrier family 25 (carnitine/acylcarnitine translocase), member 20 | Slc25a20 | 6.86825e-008 | -1.4151 |
| 704 | 10865105 | NM_170788 // Erc1 // ELKS/RAB6-interacting/CAST family member 1 | Erc1 | 3.19059e-006 | -1.41746 |
| 705 | 10869987 | NM_001107946 // Hook1 // hook homolog 1 (Drosophila) | Hook1 | 0.000180743 | -1.41762 |
| 706 | 10757898 | NM_053734 // Ncf1 // neutrophil cytosolic factor 1 | Ncf1 | 0.000115014 | -1.41958 |
| 707 | 10827454 | NM_016986 // Acadm // acyl-Coenzyme A dehydrogenase, C-4 to C-12 straight chain | Acadm | 2.04188e-007 | -1.42027 |
| 708 | 10940500 | NM_001044259 // Rattus norvegicus F-box protein 31 | Fbxo31 | 1.96305e-006 | -1.42139 |
| 709 | 10928813 | NM_001192002 // Mreg // melanoregulin | Mreg | 0.00204531 | -1.42216 |
| 710 | 10938488 | NM_001037367 // Asb12 // ankyrin repeat and SOCS box-containing 12 | Asb12 | 4.49988e-006 | -1.42382 |
| 711 | 10778038 | NM_001006960 // Mtp18 // mitochondrial protein 18 kDa | Mtp18 | 0.000493622 | -1.42458 |
| 712 | 10808296 | NM_053477 // Mlycd // malonyl-CoA decarboxylase | Mlycd | 1.326e-006 | -1.42632 |
| 713 | 10881140 | NM_001013936 // Slc25a34 // solute carrier family 25, member 34 // 5q36 // 29860 | Slc25a34 | 4.34719e-005 | -1.42647 |
| 714 | 10748991 | NM_001014122 // Mif4gd // MIF4G domain containing | Mif4gd | 1.60986e-005 | -1.42675 |
| 715 | 10815679 | NM_012608 // Mme // membrane metallo endopeptidase | Mme | 0.00145943 | -1.4269 |
| 716 | 10780301 | ENSRNOT00000032252 // LOC361041 // similar to hypothetical protein | LOC361041 | 2.08028e-007 | -1.42727 |
| 717 | 10865855 | NM_001191863 // Fkbp4 // FK506 binding protein 4 | Fkbp4 | 1.06473e-005 | -1.42858 |
| 718 | 10931047 | XM_002727158 // LOC363331 // similar to plasma membrane associated protein, S3-1 | LOC363331 | 7.6176e-007 | -1.42925 |
| 719 | 10871737 | NM_001107976 // Nt5c1a // 5'-nucleotidase, cytosolic IA | Nt5c1a | 2.28353e-005 | -1.42964 |
| 720 | 10758658 | NM_001134537 // RGD1310159 // similar to acetyl-coA dehydrogenase -related | RGD1310159 | 2.22037e-006 | -1.43138 |
| 721 | 10736273 | NM_019156 // Vtn // vitronectin | Vtn | 2.24722e-006 | -1.43319 |
| 722 | 10719863 | NM_012782 // Bckdha // branched chain ketoacid dehydrogenase E1, alpha polypeptid | Bckdha | 2.75672e-007 | -1.43354 |
| 723 | 10861213 | NM_001015026 // Tspan12 // tetraspanin 12 | Tspan12 | 7.98933e-006 | -1.43463 |
| 724 | 10930612 | NC_001665 // Rattus norvegicus strain BN/SsNHsdMCW mitochondrion |  | 3.49312e-005 | -1.43722 |
| 725 | 10788483 | ENSRNOT00000015142 // Lonrf1 // LON peptidase N-terminal domain and ring finger | Lonrf1 | 0.00458643 | -1.43744 |
| 726 | 10881989 | ENSRNOT00000046552 // Rnf207 // ring finger protein 207 | Rnf207 | 0.000141262 | -1.43881 |
| 727 | 10770637 | NM_172042 // Kcnk2 // potassium channel, subfamily K, member 2 | Kcnk2 | 0.000135464 | -1.44165 |
| 728 | 10747813 | NM_012651 // Slc4a1 // solute carrier family 4 (anion exchanger), member 1 | Slc4a1 | 0.00176754 | -1.44545 |
| 729 | 10746399 | NM_001034951 // Acsf2 // acyl-CoA synthetase family member 2 | Acsf2 | 2.71194e-008 | -1.44834 |
| 730 | 10705631 | NM_022594 // Ech1 // enoyl coenzyme A hydratase 1, peroxisomal | Ech1 | 7.43957e-006 | -1.45178 |
| 731 | 10850488 | ENSRNOT00000015637 // RGD1308023 // similar to CG5521-PA | RGD1308023 | 2.12543e-005 | -1.45196 |
| 732 | 10920122 | NM_145776 // Slc38a3 // solute carrier family 38, member 3 | Slc38a3 | 3.09416e-005 | -1.45582 |
| 733 | 10775519 | NM_001025670 // Agpat9 // 1-acylglycerol-3-phosphate O-acyltransferase 9 | Agpat9 | 8.43591e-006 | -1.45931 |
| 734 | 10732068 | NM_017306 // Eci 1 // enoyl-CoA delta isomerase 1 (Eci1), nuclear gene encoding mitochondrial protein | Eci1 | 1.96546e-006 | -1.45999 |
| 735 | 10869267 | NM_001025697 // Hsdl2 // hydroxysteroid dehydrogenase like 2 | Hsdl2 | 4.16258e-006 | -1.46004 |
| 736 | 10861132 | NM_001109222 // Ppp1r3a // protein phosphatase 1, regulatory (inhibitor) subunit 3A | Ppp1r3a | 5.73087e-006 | -1.46658 |
| 737 | 10799622 | NM_001037543 // Cdnf // cerebral dopamine neurotrophic factor | Cdnf | 1.5261e-008 | -1.46906 |
| 738 | 10803003 | NM_001106129 // Zadh2 // zinc binding alcohol dehydrogenase, domain containing 2 | Zadh2 | 6.1769e-006 | -1.47063 |
| 739 | 10898022 | NM_001170542 // Csdc2 // cold shock domain containing C2, RNA binding | Csdc2 | 0.000157154 | -1.47069 |
| 740 | 10791677 | NM_012820 // Acsl1 // acyl-CoA synthetase long-chain family member 1 | Acsl1 | 8.93959e-007 | -1.47307 |
| 741 | 10845647 | NM_012789 // Dpp4 // dipeptidylpeptidase 4 | Dpp4 | 0.000355197 | -1.47586 |
| 742 | 10875126 | NM_001025423 // Adhfe1 // alcohol dehydrogenase, iron containing, 1 | Adhfe1 | 2.09668e-007 | -1.47864 |
| 743 | 10703063 | NM_019230 // Slc22a3 // solute carrier family 22 (extraneuronal monoamine transporter), member 3 | Slc22a3 | 0.000143388 | -1.48326 |
| 744 | 10859919 | NM_001191860 // Actr3b // ARP3 actin-related protein 3 homolog B (yeast) | Actr3b | 0.000410156 | -1.48571 |
| 745 | 10918501 | NM_001108163 // Fam81a // family with sequence similarity 81, member A | Fam81a | 0.00160835 | -1.49769 |
| 746 | 10718696 | NM_001008557 // Znf667 // zinc finger protein 667 | Znf667 | 3.60063e-006 | -1.49802 |
| 747 | 10730830 | ENSRNOT00000029471 // Rccd1 // RCC1 domain containing 1 | Rccd1 | 0.000260778 | -1.49821 |
| 748 | 10867955 | NM_134331 // Epha7 // Eph receptor A7 | Epha7 | 0.00170299 | -1.49858 |
| 749 | 10897360 | NM_031039 // Gpt // glutamic-pyruvate transaminase (alanine aminotransferase) // | Gpt | 1.12289e-005 | -1.50325 |
| 750 | 10709083 | NM_013167 // Ucp3 // uncoupling protein 3 (mitochondrial, proton carrier) | Ucp3 | 0.0018723 | -1.50596 |
| 751 | 10904528 | NM_001130546 // Lynx1 // Ly6/neurotoxin 1 | Lynx1 | 9.97117e-006 | -1.50752 |
| 752 | 10784747 | NM_012767 // Gnrh1 // gonadotropin-releasing hormone 1 (luteinizing-releasing hormone) | Gnrh1 | 8.48504e-005 | -1.5096 |
| 753 | 10912567 | NM_001108180 // Ky // kyphoscoliosis peptidase | Ky | 2.06732e-006 | -1.51118 |
| 754 | 10770313 | NM_001013185 // Cabc1 // chaperone activity of bc1 complex-like, mitochondrial | Cabc1 | 1.86479e-007 | -1.51229 |
| 755 | 10865940 | NM_001010964 // Klrb1a // killer cell lectin-like receptor subfamily B, member 1 | Klrb1a | 0.0012479 | -1.51517 |
| 756 | 10833635 | NM_138831 // Slc16a10 // solute carrier family 16 (monocarboxylic acid transporter | Slc16a10 | 8.22676e-006 | -1.52956 |
| 757 | 10833659 | NM_031011 // Amd1 // adenosylmethionine decarboxylase 1 | Amd1 | 0.000400285 | -1.53323 |
| 758 | 10811160 | NM_001008893 // Ldhd // lactate dehydrogenase D= | Ldhd | 2.45114e-008 | -1.534 |
| 759 | 10876719 | NM_012496 // Aldob // aldolase B, fructose-bisphosphate | Aldob | 1.26135e-005 | -1.5362 |
| 760 | 10730898 | NM_017274 // Gpam // glycerol-3-phosphate acyltransferase, mitochondrial | Gpam | 1.28082e-007 | -1.53655 |
| 761 | 10721344 | NM_001106255 // RGD1309036 // hypothetical LOC292874 | RGD1309036 | 6.92628e-007 | -1.54 |
| 762 | 10752352 | NM_001135778 // Prodh // proline dehydrogenase | Prodh | 0.0014725 | -1.54761 |
| 763 | 10739334 | NM_053703 // Map2k6 // mitogen-activated protein kinase kinase 6 | Map2k6 | 0.000417008 | -1.54935 |
| 764 | 10703850 | NM_001107473 // Zim1 // zinc finger, imprinted 1 | Zim1 | 0.000318791 | -1.55118 |
| 765 | 10852884 | NM_001109216 // Asb10 // ankyrin repeat and SOCS box-containing 10 | Asb10 | 7.57737e-006 | -1.55142 |
| 766 | 10783981 | NM_001108378 // Sdr39u1 // short chain dehydrogenase/reductase family 39U, member 1 | Sdr39u1 | 2.10593e-007 | -1.55214 |
| 767 | 10765124 | NM_030865 // Myoc // myocilin | Myoc | 0.000996816 | -1.55268 |
| 768 | 10720884 | NM_172317 // Fxyd3 // FXYD domain-containing ion transport regulator 3 | Fxyd3 | 7.12078e-005 | -1.55544 |
| 769 | 10817222 | NM_001109189 // Lingo4 // leucine rich repeat and Ig domain containing 4 | Lingo4 | 4.26292e-006 | -1.5573 |
| 770 | 10818097 | NM_001005540 // Ppm1j // protein phosphatase 1J | Ppm1j | 1.20438e-005 | -1.5586 |
| 771 | 10837097 | ENSRNOT00000050135 // Uncharacterized protein | F1ly53 | 6.72554e-005 | -1.56411 |
| 772 | 10813578 | NM_001044252 // RGD1306809 // similar to hypothetical protein FLJ30596 | RGD1306809 | 1.09907e-006 | -1.56767 |
| 773 | 10885851 | NM_138907 // Acot2 // acyl-CoA thioesterase 2 | Acot2 | 4.62713e-006 | -1.57772 |
| 774 | 10905233 | NM_053331 // Txn2 // thioredoxin 2 | Txn2 | 3.23019e-006 | -1.58081 |
| 775 | 10806229 | NM_017004 // Es1 // esterase 1 | Es1 | 4.68026e-005 | -1.58511 |
| 776 | 10859095 | NM_001085403 // Klrb1c // killer cell lectin-like receptor subfamily B member 1C | Klrb1c | 4.40232e-005 | -1.58536 |
| 777 | 10904948 | NM_053437 // Dgat1 // diacylglycerol O-acyltransferase homolog 1 (mouse) | Dgat1 | 1.3932e-006 | -1.58721 |
| 778 | 10836679 | NM_001191901 // Myo3b // myosin IIIB | Myo3b | 0.000956304 | -1.59183 |
| 779 | 10918867 | XM_003754442 // dynein light chain 1-like | LOC100363141 | 0.000249935 | -1.62808 |
| 780 | 10936853 | NM_206950 // Mid1ip1 // MID1 interacting protein 1 | Mid1ip1 | 0.00258396 | -1.63871 |
| 781 | 10702432 | ENSRNOT00000016989 // RGD1311933 // similar to RIKEN cDNA 2310057J18 | RGD1311933 | 0.000273066 | -1.64082 |
| 782 | 10734701 | ENSRNOT00000036752 // Dhrs7c // dehydrogenase/reductase (SDR family) member 7C | Dhrs7c | 1.87296e-007 | -1.64664 |
| 783 | 10738514 | ENSRNOT00000028277 // Cd300lg // Cd300 molecule-like family member G | Cd300lg | 0.000969779 | -1.64853 |
| 784 | 10802407 | NM_172224 // Impa2 // inositol (myo)-1(or 4)-monophosphatase 2 | Impa2 | 2.52953e-006 | -1.64996 |
| 785 | 10763459 | NM_001047865 // Cntnap5a // contactin associated protein-like 5A | Cntnap5a | 3.00211e-005 | -1.65095 |
| 786 | 10752081 | NM_133606 // Ehhadh // enoyl-Coenzyme A, hydratase/3-hydroxyacyl Coenzyme A dehydrogenase | Ehhadh | 6.158e-005 | -1.65963 |
| 787 | 10931038 | NM_001134637 // Plin5 // perilipin 5 | Plin5 | 3.22918e-006 | -1.66312 |
| 788 | 10893899 | NM_012793 // Gamt // guanidinoacetate N-methyltransferase | Gamt | 4.7994e-005 | -1.67199 |
| 789 | 10939564 | NM_031612 // Apln // apelin | Apln | 3.26367e-005 | -1.69239 |
| 790 | 10804552 | NM_001131003 // LOC100174910 // glutaredoxin-like protein | LOC100174910 | 0.000951757 | -1.69528 |
| 791 | 10881766 | NM_001108693 // Rbp7 // retinol binding protein 7, cellular | Rbp7 | 0.000494982 | -1.71556 |
| 792 | 10769998 | NM_012505 // Atp1a2 // ATPase, Na+/K+ transporting, alpha 2 polypeptide | Atp1a2 | 2.22605e-006 | -1.7235 |
| 793 | 10787021 | NM_024378 // Grid1 // glutamate receptor, ionotropic, delta 1 | Grid1 | 0.000158992 | -1.7246 |
| 794 | 10872511 | NM_017209 // Mecr // mitochondrial trans-2-enoyl-CoA reductase | Mecr | 0.000485611 | -1.72775 |
| 795 | 10820308 | NM_001191843 // Atp6ap1l // ATPase, H+ transporting, lysosomal accessory protein | Atp6ap1l | 4.6039e-005 | -1.73853 |
| 796 | 10805092 | XM_001068958 // LOC684108 // hypothetical protein LOC684108 | LOC684108 | 3.67649e-005 | -1.75792 |
| 797 | 10713480 | NM_139337 // Macrod1 // MACRO domain containing 1 | Macrod1 | 2.91176e-009 | -1.76762 |
| 798 | 10737047 | NM_019174 // Car4 // carbonic anhydrase 4 | Car4 | 2.2643e-006 | -1.77344 |
| 799 | 10815652 | NM_012800 // P2ry1 // purinergic receptor P2Y, G-protein coupled, 1 | P2ry1 | 0.000120102 | -1.77865 |
| 800 | 10861224 | [GENSCAN00000019548](https://www.affymetrix.com/analysis/netaffx/exon/rna.affx?pk=135094425) |  | 1.50554e-008 | -1.85537 |
| 801 | 10783713 | NR_037320// microRNA mir-3546 | Mir3546 | 0.00292512 | -1.85906 |
| 802 | 10723866 | NM_001012345 // Dgat2 // diacylglycerol O-acyltransferase homolog 2 (mouse) | Dgat2 | 0.000640476 | -1.86667 |
| 803 | 10875631 | NM_057197 // Decr1 // 2,4-dienoyl CoA reductase 1, mitochondrial | Decr1 | 5.71423e-005 | -1.88074 |
| 804 | 10897062 | NM_001130547 // Gpihbp1 // glycosylphosphatidylinositol anchored high density lipoprotein binding protein 1 | Gpihbp1 | 6.17726e-005 | -1.89156 |
| 805 | 10717359 | NM_021666 // Trdn // triadin | Trdn | 1.62967e-006 | -1.89373 |
| 806 | 10860900 | NM_053551 // Pdk4 // pyruvate dehydrogenase kinase, isozyme 4 | Pdk4 | 0.000144445 | -1.91242 |
| 807 | 10819318 | NM_080399 // Ddit4l // DNA-damage-inducible transcript 4-like | Ddit4l | 4.22599e-006 | -1.93604 |
| 808 | 10817331 | NM_001106449 // Tmod4 // tropomodulin 4 | Tmod4 | 2.06334e-006 | -1.93635 |
| 809 | 10784621 | NM_022936 // Ephx2 // epoxide hydrolase 2, cytoplasmic | Ephx2 | 0.00193917 | -1.93664 |
| 810 | 10934784 | ENSRNOT00000045001 // Pcdh11x // protocadherin 11 X-linked | Pcdh11x | 0.000112235 | -1.93958 |
| 811 | 10909621 | NM_001008880 // Scn4b // sodium channel, voltage-gated, type IV, beta | Scn4b | 3.25135e-006 | -2.37811 |
| 812 | 10876060 | NM_019157 // Aqp7 // aquaporin 7 | Aqp7 | 3.48157e-007 | -2.50929 |
| 813 | 10707303 | ENSRNOT00000042952 // Uncharacterized protein | F1m737 | 4.59413e-006 | -3.85258 |
| 814 | 10712090 | NM_031543 // Cyp2e1 // cytochrome P450, family 2, subfamily e, polypeptide 1 | Cyp2e1 | 0.000121895 | -4.639 |
| ESTs | | | | | |
| 815 | 10722433 | ENSRNOT00000053117 | --- | 0.00119211 | 1,58618 |
| 816 | 10722421 | ENSRNOT00000053882 | --- | 0.0012044 | 1,5607 |
| 817 | 10722441 | ENSRNOT00000052661 | --- | 0.000548303 | 1,54156 |
| 818 | 10722431 | ENSRNOT00000053880 | --- | 0.00140939 | 1,51513 |
| 819 | 10722459 | ENSRNOT00000052564 | --- | 0.00359066 | 1,51312 |
| 820 | 10722429 | ENSRNOT00000053184 | --- | 0.00448084 | 1,50571 |
| 821 | 10722471 | ENSRNOT00000052507 | --- | 0.0013233 | 1,47981 |
| 822 | 10722451 | ENSRNOT00000053019 | --- | 0.00373725 | 1,46816 |
| 823 | 10911849 | [ENSRNOT00000053218](https://www.affymetrix.com/analysis/netaffx/exon/rna.affx?pk=135158044) | --- | 0.00393609 | -1,30804 |
| 824 | 10867975 | [ENSRNOT00000063601](https://www.affymetrix.com/analysis/netaffx/exon/rna.affx?pk=135158738) | --- | 0.00185402 | -1,32826 |
| 825 | 10735369 | [ENSRNOT00000052640](https://www.affymetrix.com/analysis/netaffx/exon/rna.affx?pk=135158412) | --- | 0.00473305 | -1,37777 |
| 826 | 10823172 | [ENSRNOT00000039100](https://www.affymetrix.com/analysis/netaffx/exon/rna.affx?pk=135154070) | --- | 0.00107731 | -1,38163 |
| 827 | 10933995 | [ENSRNOT00000058167](https://www.affymetrix.com/analysis/netaffx/exon/rna.affx?pk=135153639) | --- | 0.000678406 | -1,4849 |
| 828 | 10750898 | [ENSRNOT00000070025](https://www.affymetrix.com/analysis/netaffx/exon/rna.affx?pk=135158226) | --- | 0.00354632 | -1,50272 |
| 829 | 10840502 | [ENSRNOT00000054264](https://www.affymetrix.com/analysis/netaffx/exon/rna.affx?pk=135158178) | --- | 0.000919383 | -1,51254 |
| No assignment transcripts ID | | | | | |
| 830 | 10820324 | --- | --- | 1.30485e-005 | 1.73218 |
| 831 | 10837170 | --- | --- | 0.000243981 | 1.61897 |
| 832 | 10903674 | --- | --- | 0.000445622 | 1.60202 |
| 833 | 10877667 | --- | --- | 1.81147e-005 | 1.36822 |
| 834 | 10787514 | --- | --- | 0.00344283 | 1.33239 |
| 835 | 10870565 | --- | --- | 0.00178697 | 1.31954 |
| 836 | 10799156 | --- | --- | 0.00102462 | -1.31995 |
| 837 | 10865103 | --- | --- | 0.00154585 | -1.34023 |
| 838 | 10857403 | --- | --- | 0.0011604 | -1.3598 |
| 839 | 10865336 | --- | --- | 6.3026e-005 | -1.3773 |
| 840 | 10821989 | --- | --- | 1.94247e-005 | -1.4008 |
