# Supplementary material for: Transcriptional profiling of left ventricle and peripheral blood mononuclear cells in a rat model of postinfarction heart failure
Source: BMC Med Genomics. 2013 Nov 8;6:49. doi: 10.1186/1755-8794-6-49 (PMC4226214; doi:10.1186/1755-8794-6-49)
Supplement: Additional file 3 — AmiGO Term Enrichment analysis of differentially expressed transcripts in LVs between rats with large-size of infarction and sham-operated ones. [file 1755-8794-6-49-S3.doc]

**Additional file 3: AmiGO Term Enrichment analysis of differentially expressed transcripts in LVs between rats with large-size of infarction and sham-operated ones**

| **Biological Process** | | |
| --- | --- | --- |
| **GO Term** | **P-value** | **Genes** |
| [GO:0010033 response to organic substance](http://amigo.geneontology.org/cgi-bin/amigo/term_details?term=GO:0010033&session_id=) | 3.39e-17 | Ltbp3Tgfb2Timp2Rpl3Abcg1Adcy7CtgfLtc4sEno2Gnao1Ccnd1Mmp2Mgst1Cxcl13ApoeInhbaAcadmCfhPtafrC5ar1Dgat2BckdhaHadhaGpamMyofTrdnCacna1gAspnFgfr2MgpLumPtgs1Pea15aSpp1Ifi204Cd55Fads1Dpysl3Serpinf1Gpihbp1Csf2rbSfrp1Col1a2LbpCyp2e1Ucp3Abcb4Ccr1CdaFgfr1CtshGot2Col3a1Creb3l2AcanTgfb1i1Plod2Clec7aUcp2Slit3PtgisAcacbCol1a1Acsl1GrnIl18PrkcdMlxiplTxn2Fgf7Aldh1a1Casp12Sdc2C1qbPdk3Sfrp4Cd14Cuzd1Runx1LitafCdh13Timp1Acot2Tgfb1Gnrh1Pfkfb1Ptgs2FigfCcbp2Chi3l1Adamtsl2Plscr1PtgfrXylt1Hsd11b1NppaStc2Sulf2Runx2RelnPrkcbP2ry1Abca1Sulf1AldobG6pdSmad7Ltbp2Tnfsf18Gpx3Csf1rAtp1a2TfDab2Anxa1Tnfrsf11bAxlMmeFzd2CnpCd44SelplgFn1C3Cd68Pdk4PpargEcm1Has2Cx3cr1Ildr2Ccl2Lpin1Trim16MdkNr4a3LoxAcadsIfitm1Tlr5Asah2Car4Mmp14C1sHadhCcr5Aff3Apobec1Htr1bOsmrFgf18CtssNcam1Thbs1Bmp6 |
| [GO:0009611 response to wounding](http://amigo.geneontology.org/cgi-bin/amigo/term_details?term=GO:0009611&session_id=) | 2.24e-15 | Tgfb2VcanLtc4sCd28Mmp2ApoeCfhPtafrC5ar1MyofPtprjSpp1C6Cd55Cysltr1Dpysl3Serpinf1LbpZyxCd46Ccr1Igsf10F13a1VwfCol3a1Efemp2PtgisCol1a1S1pr3Nt5eIl18PrkcdOlr1Nupr1Txn2Fgf7Sdc2Fcgr1aCd14Timp1Tgfb1Anxa2Alox5Csf1Ptgs2P2ry12Serpine2Chi3l1Plscr1Xylt1Ephx2RelnP2ry1Serping1Ncf1Fcer1gCsf1rTfFmodAnxa1AxlCd44AoahEntpd1Fn1C3PpargEcm1PllpAlox5apCcl2MdkLoxDdr1Ccr5Abhd2Ninj2Ncam1Thbs1Tgm2 |
| [GO:0007155 cell adhesion](http://amigo.geneontology.org/cgi-bin/amigo/term_details?term=GO:0007155&session_id=) | 7.95e-14 | Cadm3Tgfb2VcanEpha7CtgfVtnItgamFstl3Svep1Mmp2Cxcl13PtprjSpp1Antxr1Lyve1Fbln2Lamc1Itgbl1Aebp1VwfThy1ChadCol3a1AcanTnfrsf12aClec7aCol1a1Il18PrkcdOlr1Rnd1Hapln1Wisp2GpnmbCyth4Cdh13Tgfb1OmdCsf1Cntnap5aCd4P2ry12Emilin1Serpine2Thbs4Nrxn1Srpx2Dpp4Epdr1PostnRelnCpxm2Sulf1Smoc1Smad7Col8a1Dab2Itga11AxlCd44Col12a1SelplgCdh22Fn1CompHas2Spon1Ddr1LOC681309Mmp14Ccr5Ninj2Actn1Ncam1Nckap1lThbs1Tgm2 |
| [GO:0022610 biological adhesion](http://amigo.geneontology.org/cgi-bin/amigo/term_details?term=GO:0022610&session_id=) | 1.02e-13 | Cadm3Tgfb2VcanEpha7CtgfVtnItgamFstl3Svep1Mmp2Cxcl13PtprjSpp1Antxr1Lyve1Fbln2Lamc1Itgbl1Aebp1VwfThy1ChadCol3a1AcanTnfrsf12aClec7aCol1a1Il18PrkcdOlr1Rnd1Hapln1Wisp2GpnmbCyth4Cdh13Tgfb1OmdCsf1Cntnap5aCd4P2ry12Emilin1Serpine2Thbs4Nrxn1Srpx2Dpp4Epdr1PostnRelnCpxm2Sulf1Smoc1Smad7Col8a1Dab2Itga11AxlCd44Col12a1SelplgCdh22Fn1CompHas2Spon1Ddr1LOC681309Mmp14Ccr5Ninj2Actn1Ncam1Nckap1lThbs1Tgm2 |
| [GO:0044281 small molecule metabolic process](http://amigo.geneontology.org/cgi-bin/amigo/term_details?term=GO:0044281&session_id=) | 3.33e-13 | Car3Impa2Sptlc2AspdhTgfb2Timp2Abcg1Adcy7Acsf3Ltc4sGnao1EhhadhAtp1a3Mgst1Cxcl13ApoeBcat2AcadmGfpt2PtafrMe2Pon3Slc22a5Dgat2BckdhaHadhaGpamPtgs1Lrp1Acy1Uap1MecrFads1Dpysl3Soat1MceeUcp3AcadvlCdaTpm2DseGot2Npr3Thy1HpgdsGpcpd1Acsf2PtgisAcacbScpep1Nt5eAcsl1Akr1b10Dhrs4PrkcdTbxas1Txn2Fgf7Aldh1a1BgnMlycdPdk3Cpt2Ankrd23LipeSuclg1Nt5c1aHadhbAcot2Tgfb1Egln1GamtAlox5Acox1Ptgs2PthlhDecr1Plscr1Xylt1Hsd11b1NppaAmd1Pcbd1Sulf2Eci1ProdhEphx2Mthfd2Bcat1Fads2RelnPrkcbClpxP2ry1Abca1Ncf1Sulf1AldobG6pdMid1ip1Gna15Gpx3Atp1a2Abcd3Fdft1GdaMaoaAnxa1Macrod1Ch25hMmeFzd2CnpDkk3Qrsl1Entpd1Pde11aC3Pdk4PpargLrrc16aAlox5apPank1Lpin1AcadsCar4HadhApobec1Ech1Prtfdc1Htr1bCratAcot7GptNckap1lBmp6 |
| [GO:0070887 cellular response to chemical stimulus](http://amigo.geneontology.org/cgi-bin/amigo/term_details?term=GO:0070887&session_id=) | 5.06e-13 | Ltbp3Tgfb2Timp2Rpl3Adcy7CtgfLtc4sItgamFstl3Ccnd1Mmp2Mgst1Cxcl13ApoeInhbaCfhPtafrPon3C5ar1Dgat2GpamMyofPtprjCacna1gAspnFgfr2Etv5Spp1Ifi204S100a10Dpysl3Sfrp1Col1a2LbpCyp2e1Fndc1Ucp3Ccr1Fgfr1CtshCol3a1AcanTgfb1i1Plod2Ucp2Slit3Ccl6Nbl1PtgisCol1a1Acsl1Il18PrkcdMlxiplFgf7Casp12Pdk3C3ar1Cd14Cuzd1LitafCdh13Tgfb1Anxa2Ptgs2FigfCcbp2Chi3l1Adamtsl2Plscr1Hsd11b1Thbs4Stc2Sulf2Runx2RelnPrkcbAbca1Sulf1AldobG6pdSmad7Ltbp2Tnfsf18Fcer1gGpx3Csf1rTfDab2Anxa1AxlMmeFzd2Cd44SelplgFn1Cd68Pdk4Lpar1PpargEcm1Has2Cx3cr1Alox5apCcl2Lpin1Tlr5Ccr5Apobec1OsmrFgf18CtssNcam1Nckap1lThbs1 |
| [GO:0051239 regulation of multicellular organismal process](http://amigo.geneontology.org/cgi-bin/amigo/term_details?term=GO:0051239&session_id=) | 5.91e-13 | Ltbp3Tgfb2Timp2Loxl2Hcn4Agpat2CtgfTmem176aCd28Gnao1Fstl3Ccnd1Mmp2Fkbp4ApoeInhbaPolr3gSfrp2Slc22a5MafbGpamPtprjCacna1gAspnFgfr2MgpEtv5Ptgs1Spp1Ifi204C6Bex1Cysltr1Dpysl3Serpinf1Gpihbp1Sfrp1Tspan12LbpCcr1Fgfr1CtshTiam1Thy1Tgfb1i1Tnfrsf12aClec7aAplnNbl1PtgisCol1a1S1pr3GrnAsb4Il18Htr2aPrkcdMlxiplTbxas1Nupr1Fgf7C3ar1Sfrp4Cacna2d2Cd14FrzbRunx1Tlr7LitafPmp22Pdlim5Timp1Tgfb1RT1-DMaOmdEgln1Gnrh1Anxa2Bmp1GamtNfam1CorinAlox5Csf1Ptgs2Tmem176bFigfPthlhSerpine2Chi3l1Scn3bXylt1NppaThbs4Nrxn1Stc2Sulf2Srpx2Tbx3Runx2RelnPrkcbP2ry1Serping1Ncf1Sulf1G6pdNlrp10Smoc1Smad7Fcer1gCsf1rAtp1a2TfDab2Cpeb1Tnfrsf11bAxlFzd2Cd44Scn4bC3Lpar1PpargEcm1Cx3cr1Ccl2Itm2cTrim16AspaIfitm1Tlr5C1qcCcr5Htr1bFgf18CtssNcam1Nckap1lThbs1Bmp6 |
| [GO:0009605 response to external stimulus](http://amigo.geneontology.org/cgi-bin/amigo/term_details?term=GO:0009605&session_id=) | 8.89e-13 | Tgfb2Sema5aEpha7Ltc4sCd28ItgamCcnd1Mmp2Cxcl13ApoeAcadmCfhSfrp2PtafrC5ar1BckdhaGpamCybbPtprjMgpSpp1C6Cd55Cysltr1Fads1Serpinf1Sfrp1LbpZyxCd46Ucp3Ccr1CdaAcanClec7aSlit3Ccl6Nbl1EnahPtgisCol1a1Nt5eAcsl1Il18PrkcdTxn2Fgf7Lsp1C3ar1Ankrd23Fcgr1aTlr7Cdh13Acot2Tgfb1Pfkfb1Anxa2Alox5Ptgs2FigfSerpine2Chi3l1Xylt1Hsd11b1Thbs4Nrxn1Stc2RelnPrkcbP2ry1Abca1Serping1AldobG6pdEtv1Fcer1gAtp1a2Anxa1Tnfrsf11bAxlFzd2Cd44AoahCpC3Pdk4Lpar1PpargCx3cr1Alox5apCcl2Slc22a3Nr4a3AcadsTlr5Mmp14Ccr5Nckap1lThbs1Tgm2 |
| [GO:0048731 system development](http://amigo.geneontology.org/cgi-bin/amigo/term_details?term=GO:0048731&session_id=) | 1.39e-12 | Ltbp3Tgfb2Timp2Loxl2Sema5aFcgr2aVcanEpha7Agpat2CtgfTmem176aBokRaph1Cd28Gnao1ItgamFstl3Ccnd1Mmp2Mgst1Cxcl13Fkbp4ApoeBcat2InhbaAcadmSfrp2C5ar1Slc22a5Dgat2MafbPtprjTrdnAspnFgfr2MgpLumEtv5Lrp1Spp1Ifi204Ptcd2C6Bex1Cysltr1Dpysl3Serpinf1Sfrp1Tspan12Col1a2Lamc1Loxl3Ccr1Fgfr1CtshTiam1VwfFbn1Npr3Thy1ChadCol3a1Creb3l2AcanTgfb1i1Tnfrsf12aUcp2Slit3Nbl1Zfp238EnahPtgisFlnaCol1a1Nt5eGrnAsb4Il18Prrx2Unc93b1Rnd1Nupr1Col18a1Fgf7Aldh1a1TaglnSdc2Tgm1C1qbKyC3ar1 Sfrp4Cacna2d2Fcgr1aFrzbRunx1Pmp22GpnmbPdlim5Cdh13Timp1Tgfb1RT1-DMaOmdEgln1Gnrh1Pfkfb1Anxa2Bmp1GamtNfam1Csf1Cd4Ptgs2Tmem176bFigfP2ry12Cyp4b1Lcp1Irx4PthlhSerpine2Chi3l1Scn3bPlscr1Xylt1Hsd11b1NppaThbs4Nrxn1Stc2Dbn1Sulf2Srpx2Pls3Tbx3Runx2RelnPrkcbGas7Lgals3P2ry1Sulf1AldobG6pdEtv1Smoc1Smad7Fcer1gCol8a1Csf1rElnSat1Uchl1Tmem100Rab23MregTfAlx3Dab2FmodAnxa1Tnfrsf11bGpr183AxlMmeFzd2CnpCd44Prrx1CpDkk3Cdh22Fn1C3Lpar1CompPpargEcm1Has2Cx3cr1Ildr2PllpCcl2Itm2cCasp7Lpin1Trim16MdkNr4a3LoxAspaTlr5Ddr1Car4C1qcC1qtnf5LOC681309Mmp14C1sCcr5Fgf18CtssNcam1Nckap1lThbs1Tgm2Bmp6 |
| [GO:0009719 response to endogenous stimulus](http://amigo.geneontology.org/cgi-bin/amigo/term_details?term=GO:0009719&session_id=) | 2.22e-12 | Ltbp3Tgfb2Abcg1CtgfEno2Gnao1Ccnd1Mmp2Mgst1Cxcl13ApoeInhbaAcadmC5ar1BckdhaHadhaGpamCacna1gAspnFgfr2MgpPtgs1Pea15aSpp1Cd55Fads1Serpinf1Gpihbp1Sfrp1Col1a2Cyp2e1Ucp3Abcb4CdaFgfr1CtshCol3a1Tgfb1i1Plod2Ucp2Slit3Col1a1GrnIl18PrkcdTxn2Fgf7Aldh1a1Casp12Sdc2C1qbSfrp4Cuzd1Cdh13Timp1Acot2Tgfb1Gnrh1Pfkfb1Ptgs2Adamtsl2Hsd11b1NppaStc2Sulf2Runx2RelnPrkcbAbca1Sulf1AldobSmad7Ltbp2Gpx3Atp1a2TfDab2Anxa1Tnfrsf11bFzd2Cd44Fn1C3Pdk4PpargHas2Cx3cr1Ccl2Lpin1Trim16MdkNr4a3LoxAcadsCar4Mmp14C1sHadhApobec1Htr1bFgf18CtssNcam1Thbs1Bmp6 |
| [GO:0006950 response to stress](http://amigo.geneontology.org/cgi-bin/amigo/term_details?term=GO:0006950&session_id=) | 4.77e-12 | Car3Tgfb2Loxl2VcanCtgfLtc4sCd28Gnao1Ccnd1Mmp2Mgst1Cxcl13ApoeAcadmCfhPolr3gSfrp2PtafrC5ar1Tsc22d4GpamCybbMyofPtprjEtv5Ptgs1Spp1Ifi204C6Cd55Cysltr1Fads1Dpysl3Serpinf1Sfrp1LbpZyxCyp2e1Fndc1Cd46Ucp3Ccr1Cotl1Igsf10F13a1HampVwfCol3a1Creb3l2Tgfb1i1Plod2Clec7aUcp2Mapk8ip1Efemp2PtgisCol1a1S1pr3Nt5eIl18Htr2aPrkcdUnc93b1Olr1Nupr1Tp53inp1Col18a1Rad23aTxn2Fgf7Aldh1a1Casp12Lsp1Sdc2C1qbPdk3TfecSfrp4Fcgr1aCd14Runx1Tlr7Timp1Acot2Tgfb1Egln1Pfkfb1Anxa2Alox5Csf1Mapk12Cd4Ptgs2FigfP2ry12Scara5Serpine2Chi3l1Plscr1Xylt1NppaStc2Ephx2Mthfd2Fbxo31Dpp4RelnLgals3P2ry1Abca1Serping1Ncf1AldobLyz2G6pdNlrp10Smad7Fcer1gGpx3Csf1rRGD1305645Gpx8Uchl1TfDab2FmodAnxa1Phlda3AxlC1qaCd44AoahEntpd1Fn1C3Pdk4PpargEcm1Has2PllpAlox5apCcl2Hspb3MdkNr4a3LoxAcadsIfitm1Ddr1Exosc5C1qcMmp14C1sCcr5Apobec1Abhd2Ninj2Ncam1Thbs1Tgm2 |
| [GO:0006631 fatty acid metabolic process](http://amigo.geneontology.org/cgi-bin/amigo/term_details?term=GO:0006631&session_id=) | 9.82e-12 | Acsf3Ltc4sEhhadhAcadmHadhaGpamPtgs1MecrFads1Ucp3AcadvlHpgdsAcsf2PtgisAcacbAcsl1Tbxas1MlycdPdk3Cpt2Ankrd23HadhbAcot2Alox5Acox1Ptgs2Decr1Eci1Ephx2Fads2Ncf1Mid1ip1Abcd3Anxa1Ch25hC3Pdk4PpargAlox5apLpin1AcadsHadhEch1CratAcot7 |
| [GO:0044710 single-organism metabolic process](http://amigo.geneontology.org/cgi-bin/amigo/term_details?term=GO:0044710&session_id=) | 1.08e-11 | Car3Impa2Sptlc2AspdhTgfb2Timp2Ppp1r3dLoxl2Agpat2Abcg1Adcy7Acsf3CtgfLtc4sGnao1EhhadhAtp1a3Cyb5bMmp2Mgst1Cxcl13ApoeBcat2AcadmGfpt2PtafrMe2Pon3Slc22a5Dgat2BckdhaHadhaGpamCybbPtgs1Lrp1Acy1Uap1MecrFads1Dpysl3LbpSoat1MceeCyp2e1Ucp3AcadvlCdaPpp1r1aTpm2DseGot2Npr3Thy1Col3a1HpgdsGpcpd1Acsf2PtgisAcacbCol1a1Scpep1Nt5eAcsl1Akr1b10Il18Htr2aDhrs4PrkcdTbxas1Txn2Fgf7Aldh1a1BgnMlycdPdk3Cpt2Ankrd23LipeSuclg1Nt5c1aHadhbDgat1Timp1Acot2Pla2g2dTgfb1Egln1GamtAlox5Acox1Ptgs2Aox3PthlhDecr1LOC100361830Plscr1Xylt1Hsd11b1NppaAmd1Agpat9Pcbd1Sulf2Eci1ProdhEphx2Mthfd2Bcat1Fads2RelnFuca1PrkcbClpxP2ry1Abca1Ncf1Sulf1AldobG6pdMid1ip1Gna15Loxl1Gpx3Csf1rAtp1a2Abcd3Fdft1GdaMaoaAnxa1Macrod1Ch25hMmeFzd2CnpCd44AoahCpDkk3Qrsl1Entpd1Pde11aC3Pdk4PpargLrrc16aHas2Alox5apPank1Lpin1LoxAcadsAsah2Car4HadhApobec1Ech1Prtfdc1Htr1bCratAcot7GptNckap1lBmp6 |
| [GO:0032502 developmental process](http://amigo.geneontology.org/cgi-bin/amigo/term_details?term=GO:0032502&session_id=) | 2.63e-11 | Ltbp3Tgfb2Timp2Loxl2Sema5aFcgr2aVcanEpha7Hcn4Agpat2Csrp2CtgfTmem176aFhl1BokRaph1Cd28Gnao1ItgamFstl3Ccnd1Mmp2Mgst1Cxcl13Fkbp4ApoeBcat2InhbaAcadmHook1Sfrp2Fndc5C5ar1Slc22a5Dgat2MafbPtprjTrdnAspnFgfr2MgpLumEtv5Ptgs1Lrp1Spp1Ifi204Ptcd2S100a10Antxr1C6Msr1Bex1Cysltr1Fads1Dpysl3Serpinf1Sfrp1Tspan12Col1a2Soat1Lamc1Loxl3Ucp3Ccr1Igsf10Fgfr1CtshTiam1VwfFbn1Npr3Thy1ChadCol3a1Creb3l2AcanTgfb1i1Tnfrsf12aUcp2Slit3Nbl1Zfp238EnahPtgisFlnaCol1a1Nt5eGrnAsb4Il18Htr2aPrkcdPrrx2Unc93b1Rnd1Nupr1Col18a1Fgf7Aldh1a1Casp12Fndc3bTaglnSdc2Tgm1PrelpC1qbKyC3ar1Sfrp4Cacna2d2Fcgr1aFrzbRunx1Pmp22GpnmbPdlim5Cdh13Timp1Tgfb1RT1-DMaOmdEgln1Gnrh1Pfkfb1Anxa2Bmp1GamtNfam1Csf1Mapk12Cd4Ptgs2Tmem176bFigfFbln1P2ry12Cyp4b1Lcp1Irx4PthlhSerpine2Chi3l1Scn3bPlscr1Xylt1Hsd11b1NppaThbs4Nrxn1Amd1Stc2Dbn1Sulf2Srpx2Pls3Piwil2Tbx3PostnRunx2RelnPrkcbGas7Lgals3P2ry1Sulf1AldobG6pdEtv1Smoc1Fam3cSmad7Vat1Fcer1gCol8a1Csf1rRGD1305645ElnSat1Uchl1Tmem100Rab23MregTfAlx3Dab2FmodAnxa1Tnfrsf11bGpr183AxlMmeFzd2CnpCd44Prrx1CpDkk3Cdh22Slc26a5Fn1C3Lpar1CompPpargEcm1Has2Cx3cr1Ildr2PllpCcl2Itm2cCasp7Lpin1Trim16MdkNr4a3LoxAspaIfitm1Tlr5Ddr1Tmem119Car4C1qcAbca5C1qtnf5LOC681309Mmp14C1sCcr5Aff3Ninj2Olfml3Fgf18CtssNcam1Nckap1lThbs1Tgm2Bmp6 |
| [GO:0032787 monocarboxylic acid metabolic process](http://amigo.geneontology.org/cgi-bin/amigo/term_details?term=GO:0032787&session_id=) | 5.99e-11 | Acsf3Ltc4sEhhadhAcadmPon3Slc22a5HadhaGpamPtgs1MecrFads1Ucp3AcadvlDseHpgdsAcsf2PtgisAcacbScpep1Acsl1Tbxas1Aldh1a1MlycdPdk3Cpt2Ankrd23HadhbAcot2Egln1Alox5Acox1Ptgs2Decr1Eci1Ephx2Fads2Ncf1Mid1ip1Abcd3Anxa1Ch25hC3Pdk4PpargAlox5apLpin1AcadsHadhEch1CratAcot7 |
| [GO:0033993 response to lipid](http://amigo.geneontology.org/cgi-bin/amigo/term_details?term=GO:0033993&session_id=) | 7.54e-11 | Tgfb2Abcg1CtgfLtc4sEno2Ccnd1Mmp2Mgst1ApoeInhbaAcadmCfhPtafrC5ar1Dgat2BckdhaCacna1gMgpPtgs1Spp1Fads1Serpinf1Csf2rbSfrp1LbpUcp3Abcb4Ccr1CtshUcp2Slit3Col1a1Acsl1GrnIl18Aldh1a1C1qbPdk3Sfrp4Cd14Runx1LitafTgfb1Gnrh1Pfkfb1Ptgs2Plscr1PtgfrHsd11b1Stc2RelnPrkcbAbca1AldobGpx3Anxa1Tnfrsf11bAxlFzd2CnpCd44Fn1C3Pdk4PpargCx3cr1Ccl2Trim16MdkLoxAcadsTlr5Car4Mmp14Ccr5Htr1bThbs1Bmp6 |
| [GO:0006082 organic acid metabolic process](http://amigo.geneontology.org/cgi-bin/amigo/term_details?term=GO:0006082&session_id=) | 1.19e-10 | Acsf3Ltc4sEhhadhMgst1Bcat2AcadmGfpt2Me2Pon3Slc22a5BckdhaHadhaGpamPtgs1Acy1MecrFads1Ucp3AcadvlDseGot2HpgdsAcsf2PtgisAcacbScpep1Acsl1Tbxas1Aldh1a1BgnMlycdPdk3Cpt2Ankrd23Suclg1HadhbAcot2Egln1GamtAlox5Acox1Ptgs2Decr1Plscr1Xylt1Hsd11b1Pcbd1Sulf2Eci1ProdhEphx2Mthfd2Bcat1Fads2RelnNcf1Sulf1G6pdMid1ip1Gpx3Abcd3Anxa1Ch25hQrsl1C3Pdk4PpargLrrc16aAlox5apLpin1AcadsHadhEch1CratAcot7Gpt |
| [GO:0007275 multicellular organismal development](http://amigo.geneontology.org/cgi-bin/amigo/term_details?term=GO:0007275&session_id=) | 1.20e-10 | Ltbp3Tgfb2Timp2Loxl2Sema5aFcgr2aVcanEpha7Hcn4Agpat2Csrp2CtgfTmem176aFhl1BokRaph1Cd28Gnao1ItgamFstl3Ccnd1Mmp2Mgst1Cxcl13Fkbp4ApoeBcat2InhbaAcadmSfrp2C5ar1Slc22a5Dgat2MafbPtprjTrdnAspnFgfr2MgpLumEtv5Lrp1Spp1Ifi204Ptcd2C6Bex1Cysltr1Dpysl3Serpinf1Sfrp1Tspan12Col1a2Lamc1Loxl3Ccr1Igsf10Fgfr1CtshTiam1VwfFbn1Npr3Thy1ChadCol3a1Creb3l2AcanTgfb1i1Tnfrsf12aUcp2Slit3Nbl1Zfp238EnahPtgisFlnaCol1a1Nt5eGrnAsb4Il18Prrx2Unc93b1Rnd1Nupr1Col18a1Fgf7Aldh1a1TaglnSdc2Tgm1C1qbKyC3ar1Sfrp4Cacna2d2Fcgr1aFrzbRunx1Pmp22GpnmbPdlim5Cdh13Timp1Tgfb1RT1-DMaOmdEgln1Gnrh1Pfkfb1Anxa2Bmp1GamtNfam1Csf1Cd4Ptgs2Tmem176bFigfFbln1P2ry12Cyp4b1Lcp1Irx4PthlhSerpine2Chi3l1Scn3bPlscr1Xylt1Hsd11b1NppaThbs4Nrxn1Amd1Stc2Dbn1Sulf2Srpx2Pls3Tbx3Runx2RelnPrkcbGas7Lgals3P2ry1Sulf1AldobG6pdEtv1Smoc1Fam3cSmad7Fcer1gCol8a1Csf1rElnSat1Uchl1Tmem100Rab23MregTfAlx3Dab2FmodAnxa1Tnfrsf11bGpr183AxlMmeFzd2CnpCd44Prrx1CpDkk3Cdh22Fn1C3Lpar1CompPpargEcm1Has2Cx3cr1Ildr2PllpCcl2Itm2cCasp7Lpin1Trim16MdkNr4a3LoxAspaIfitm1Tlr5Ddr1Car4C1qcC1qtnf5LOC681309Mmp14C1sCcr5Aff3Olfml3Fgf18CtssNcam1Nckap1lThbs1Tgm2Bmp6 |
| [GO:0043436 oxoacid metabolic process](http://amigo.geneontology.org/cgi-bin/amigo/term_details?term=GO:0043436&session_id=) | 1.30e-10 | Acsf3Ltc4sEhhadhMgst1Bcat2AcadmGfpt2Me2Pon3Slc22a5BckdhaHadhaGpamPtgs1Acy1MecrFads1Ucp3AcadvlDseGot2HpgdsAcsf2PtgisAcacbScpep1Acsl1Tbxas1Aldh1a1BgnMlycdPdk3Cpt2Ankrd23Suclg1HadhbAcot2Egln1GamtAlox5Acox1Ptgs2Decr1Plscr1Xylt1Hsd11b1Pcbd1Sulf2Eci1ProdhEphx2Mthfd2Bcat1Fads2RelnNcf1Sulf1G6pdMid1ip1Gpx3Abcd3Anxa1Ch25hQrsl1C3Pdk4PpargAlox5apLpin1AcadsHadhEch1CratAcot7Gpt |
| [GO:0030198 extracellular matrix organization](http://amigo.geneontology.org/cgi-bin/amigo/term_details?term=GO:0030198&session_id=) | 1.90e-10 | Tgfb2Loxl2CtgfVtnSfrp2FapCol1a2Lamc1Col3a1AcanCol1a1Col18a1Tgfb1Anxa2Fbln1Emilin1Olfml2bSulf2Dpp4PostnLgals3Sulf1Smoc1ElnMfap5Tnfrsf11bFn1CompLoxDdr1 |
| [GO:0043062 extracellular structure organization](http://amigo.geneontology.org/cgi-bin/amigo/term_details?term=GO:0043062&session_id=) | 2.30e-10 | Tgfb2Loxl2CtgfVtnSfrp2FapCol1a2Lamc1Col3a1AcanCol1a1Col18a1Tgfb1Anxa2Fbln1Emilin1Olfml2bSulf2Dpp4PostnLgals3Sulf1Smoc1ElnMfap5Tnfrsf11bFn1CompLoxDdr1 |
| [GO:0044255 cellular lipid metabolic process](http://amigo.geneontology.org/cgi-bin/amigo/term_details?term=GO:0044255&session_id=) | 4.22e-10 | Impa2Sptlc2Agpat2Abcg1Acsf3Ltc4sEhhadhApoeAcadmDgat2HadhaGpamPtgs1MecrFads1Soat1Cyp2e1Ucp3AcadvlHpgdsAcsf2PtgisAcacbScpep1Acsl1Akr1b10Htr2aDhrs4PrkcdTbxas1Fgf7Aldh1a1MlycdPdk3Cpt2Ankrd23LipeHadhbDgat1Acot2Pla2g2dAlox5Acox1Ptgs2Decr1Plscr1Agpat9Eci1Ephx2Fads2Ncf1Mid1ip1Csf1rAbcd3Fdft1Anxa1Ch25hAoahC3Pdk4PpargAlox5apLpin1AcadsAsah2HadhEch1CratAcot7 |
| [GO:0048856 anatomical structure development](http://amigo.geneontology.org/cgi-bin/amigo/term_details?term=GO:0048856&session_id=) | 4.72e-10 | Ltbp3Tgfb2Timp2Loxl2Sema5aFcgr2aVcanEpha7Hcn4Agpat2Csrp2CtgfTmem176aBokRaph1Cd28Gnao1ItgamFstl3Ccnd1Mmp2Mgst1Cxcl13Fkbp4ApoeBcat2InhbaAcadmHook1Sfrp2C5ar1Slc22a5Dgat2MafbPtprjTrdnAspnFgfr2MgpLumEtv5Lrp1Spp1Ifi204Ptcd2Antxr1C6Bex1Cysltr1Dpysl3Serpinf1Sfrp1Tspan12Col1a2Lamc1Loxl3Ccr1Igsf10Fgfr1CtshTiam1VwfFbn1Npr3Thy1ChadCol3a1Creb3l2AcanTgfb1i1Tnfrsf12aUcp2Slit3Nbl1Zfp238EnahPtgisFlnaCol1a1Nt5eGrnAsb4Il18Prrx2Unc93b1Rnd1Nupr1Col18a1Fgf7Aldh1a1TaglnSdc2Tgm1C1qbKyC3ar1Sfrp4Cacna2d2Fcgr1aFrzbRunx1Pmp22GpnmbPdlim5Cdh13Timp1Tgfb1RT1-DMaOmdEgln1Gnrh1Pfkfb1Anxa2Bmp1GamtNfam1Csf1Mapk12Cd4Ptgs2Tmem176bFigfP2ry12Cyp4b1Lcp1Irx4PthlhSerpine2Chi3l1Scn3bPlscr1Xylt1Hsd11b1NppaThbs4Nrxn1Amd1Stc2Dbn1Sulf2Srpx2Pls3Piwil2Tbx3PostnRunx2RelnPrkcbGas7Lgals3P2ry1Sulf1AldobG6pdEtv1Smoc1Smad7Vat1Fcer1gCol8a1Csf1rElnSat1Uchl1Tmem100Rab23MregTfAlx3Dab2FmodAnxa1Tnfrsf11bGpr183AxlMmeFzd2CnpCd44Prrx1CpDkk3Cdh22Slc26a5Fn1C3Lpar1CompPpargEcm1Has2Cx3cr1Ildr2PllpCcl2Itm2cCasp7Lpin1Trim16MdkNr4a3LoxAspaIfitm1Tlr5Ddr1Car4C1qcC1qtnf5LOC681309Mmp14C1sCcr5Aff3Ninj2Fgf18CtssNcam1Nckap1lThbs1Tgm2Bmp6 |
| [GO:0001944 vasculature development](http://amigo.geneontology.org/cgi-bin/amigo/term_details?term=GO:0001944&session_id=) | 5.31e-10 | Tgfb2Loxl2Sema5aCtgfMmp2ApoeSfrp2PtprjFgfr2Lrp1C6Cysltr1Serpinf1Sfrp1Tspan12Col1a2Fgfr1CtshThy1Col3a1PtgisCol1a1Asb4Il18Prrx2Col18a1C3ar1Sfrp4Runx1Cdh13Egln1Anxa2Ptgs2FigfChi3l1Thbs4Nrxn1Srpx2Tbx3PrkcbSulf1Smad7Sat1Tmem100Fzd2Cd44Prrx1Fn1C3Ecm1Cx3cr1Ccl2LoxMmp14Fgf18Thbs1 |
| [GO:0019752 carboxylic acid metabolic process](http://amigo.geneontology.org/cgi-bin/amigo/term_details?term=GO:0019752&session_id=) | 5.51e-10 | Acsf3Ltc4sEhhadhMgst1Bcat2AcadmGfpt2Me2Pon3Slc22a5BckdhaHadhaGpamPtgs1Acy1MecrFads1Ucp3AcadvlDseGot2HpgdsAcsf2PtgisAcacbScpep1Acsl1Tbxas1Aldh1a1MlycdPdk3Cpt2Ankrd23Suclg1HadhbAcot2Egln1GamtAlox5Acox1Ptgs2Decr1Plscr1Hsd11b1Pcbd1Eci1ProdhEphx2Mthfd2Bcat1Fads2RelnNcf1G6pdMid1ip1Gpx3Abcd3Anxa1Ch25hQrsl1C3Pdk4PpargAlox5apLpin1AcadsHadhEch1CratAcot7Gpt |
| [GO:0044767 single-organism developmental process](http://amigo.geneontology.org/cgi-bin/amigo/term_details?term=GO:0044767&session_id=) | 6.00e-10 | Ltbp3Tgfb2Timp2Loxl2Sema5aFcgr2aVcanEpha7Hcn4Csrp2CtgfTmem176aBokRaph1Cd28Gnao1ItgamFstl3Ccnd1Mmp2Mgst1Cxcl13Fkbp4ApoeBcat2InhbaAcadmHook1Sfrp2C5ar1Slc22a5MafbPtprjTrdnAspnFgfr2MgpEtv5Ptgs1Lrp1Spp1Ifi204Ptcd2Antxr1C6Bex1Cysltr1Fads1Dpysl3Serpinf1Sfrp1Tspan12Col1a2Lamc1Loxl3Ucp3Ccr1Igsf10Fgfr1CtshTiam1VwfFbn1Thy1ChadCol3a1AcanTgfb1i1Tnfrsf12aUcp2Slit3Nbl1Zfp238EnahPtgisFlnaCol1a1Nt5eGrnAsb4Il18Htr2aPrkcdPrrx2Unc93b1Rnd1Nupr1Col18a1Fgf7Aldh1a1Casp12TaglnSdc2Tgm1PrelpC1qbKyC3ar1Sfrp4Cacna2d2Fcgr1aFrzbRunx1Pmp22Pdlim5Cdh13Timp1Tgfb1RT1-DMaEgln1Gnrh1Pfkfb1Anxa2GamtNfam1Csf1Mapk12Cd4Ptgs2Tmem176bFigfCyp4b1Lcp1Irx4PthlhSerpine2Chi3l1Plscr1Xylt1Hsd11b1NppaThbs4Nrxn1Amd1Stc2Sulf2Srpx2Pls3Piwil2Tbx3Runx2RelnPrkcbGas7P2ry1Sulf1AldobG6pdEtv1Smoc1Smad7Fcer1gCol8a1Csf1rRGD1305645ElnSat1Uchl1Tmem100Rab23MregTfAlx3Dab2FmodAnxa1Tnfrsf11bGpr183AxlMmeFzd2CnpCd44Prrx1CpDkk3Cdh22Slc26a5Fn1C3Lpar1CompPpargEcm1Has2Cx3cr1Ildr2Ccl2Itm2cCasp7Lpin1MdkNr4a3LoxAspaIfitm1Tlr5Ddr1C1qcC1qtnf5LOC681309Mmp14Ccr5Aff3Ninj2Fgf18CtssNcam1Nckap1lThbs1Tgm2Bmp6 |
| [GO:0071310 cellular response to organic substance](http://amigo.geneontology.org/cgi-bin/amigo/term_details?term=GO:0071310&session_id=) | 1.27e-09 | Ltbp3Tgfb2Timp2Rpl3Adcy7CtgfLtc4sCcnd1Mmp2Cxcl13ApoeInhbaCfhPtafrDgat2GpamMyofCacna1gAspnFgfr2Ifi204Dpysl3Sfrp1Col1a2LbpUcp3Ccr1Fgfr1CtshCol3a1AcanTgfb1i1Plod2Ucp2Slit3PtgisCol1a1Acsl1Il18PrkcdMlxiplFgf7Casp12Pdk3Cd14Cuzd1LitafCdh13Tgfb1Ptgs2FigfCcbp2Chi3l1Adamtsl2Plscr1Hsd11b1Stc2Sulf2Runx2RelnPrkcbAbca1Sulf1AldobSmad7Ltbp2Tnfsf18Csf1rTfDab2Anxa1AxlMmeFzd2Cd44SelplgFn1Cd68Pdk4PpargEcm1Has2Cx3cr1Ccl2Lpin1Tlr5Ccr5Apobec1OsmrFgf18CtssThbs1 |
| [GO:0050793 regulation of developmental process](http://amigo.geneontology.org/cgi-bin/amigo/term_details?term=GO:0050793&session_id=) | 1.42e-09 | Ltbp3Tgfb2Timp2Loxl2CtgfTmem176aCd28Fstl3Ccnd1Fkbp4ApoeInhbaSfrp2Fndc5MafbAspnFgfr2MgpEtv5Spp1Ifi204S100a10C6Msr1Bex1Cysltr1Dpysl3Serpinf1Sfrp1Tspan12Ccr1Fgfr1CtshTiam1Thy1Tgfb1i1Tnfrsf12aNbl1PtgisCol1a1GrnAsb4Il18Nupr1Fgf7Fndc3bC3ar1Sfrp4FrzbRunx1Pmp22Pdlim5Timp1Tgfb1RT1-DMaOmdEgln1Gnrh1Bmp1Nfam1Csf1Ptgs2Tmem176bFigfP2ry12PthlhSerpine2Chi3l1Xylt1Thbs4Nrxn1Srpx2Piwil2Tbx3Runx2RelnPrkcbGas7Sulf1Smoc1Vat1Csf1rTfDab2Tnfrsf11bAxlFzd2Cd44Slc26a5Fn1C3Lpar1PpargEcm1Cx3cr1Ccl2Itm2cLpin1Trim16AspaIfitm1C1qcAbca5Ccr5Fgf18Ncam1Nckap1lThbs1Bmp6 |
| [GO:1901700 response to oxygen-containing compound](http://amigo.geneontology.org/cgi-bin/amigo/term_details?term=GO:1901700&session_id=) | 2.21e-09 | Tgfb2Adcy7CtgfLtc4sEno2Gnao1Ccnd1Mmp2Mgst1ApoeInhbaCfhPtafrC5ar1Dgat2BckdhaGpamCacna1gPtgs1Spp1Fads1Serpinf1Gpihbp1Csf2rbSfrp1Col1a2LbpCyp2e1Ucp3Ccr1CtshGot2Col3a1AcanUcp2Slit3Col1a1Acsl1GrnIl18PrkcdMlxiplOlr1Col18a1Txn2Aldh1a1Casp12Pdk3Sfrp4Cd14Runx1LitafTgfb1Egln1Gnrh1Pfkfb1Ptgs2Plscr1PtgfrHsd11b1Stc2RelnPrkcbAbca1AldobG6pdGpx3TfAnxa1AxlFzd2CnpFn1C3Pdk4PpargCx3cr1Ildr2Ccl2Trim16Tlr5C1sCcr5Apobec1Htr1bNcam1Thbs1Bmp6 |
| [GO:0001568 blood vessel development](http://amigo.geneontology.org/cgi-bin/amigo/term_details?term=GO:0001568&session_id=) | 3.31e-09 | Tgfb2Loxl2Sema5aCtgfMmp2ApoeSfrp2PtprjFgfr2Lrp1C6Cysltr1Serpinf1Tspan12Col1a2Fgfr1CtshThy1Col3a1PtgisCol1a1Asb4Il18Prrx2Col18a1C3ar1Runx1Cdh13Egln1Anxa2Ptgs2FigfChi3l1Thbs4Nrxn1Srpx2Tbx3PrkcbSulf1Smad7Sat1Tmem100Cd44Prrx1Fn1C3Ecm1Cx3cr1Ccl2LoxMmp14Fgf18Thbs1 |
| [GO:0006954 inflammatory response](http://amigo.geneontology.org/cgi-bin/amigo/term_details?term=GO:0006954&session_id=) | 5.00e-09 | Cd28ApoeCfhPtafrC5ar1Spp1C6Cd55Cysltr1Serpinf1LbpZyxCd46Ccr1PtgisS1pr3Nt5eIl18Olr1Nupr1Fcgr1aCd14Tgfb1Alox5Csf1Ptgs2Chi3l1Plscr1Ephx2Serping1Ncf1Fcer1gCsf1rTfAnxa1AxlCd44AoahFn1C3PpargEcm1Alox5apCcl2Ccr5Thbs1Tgm2 |
| [GO:2000026 regulation of multicellular organismal development](http://amigo.geneontology.org/cgi-bin/amigo/term_details?term=GO:2000026&session_id=) | 5.42e-09 | Ltbp3Tgfb2Timp2Loxl2CtgfTmem176aCd28Fstl3Ccnd1Fkbp4ApoeInhbaSfrp2MafbAspnFgfr2MgpEtv5Spp1C6Bex1Cysltr1Dpysl3Serpinf1Sfrp1Tspan12Ccr1Fgfr1CtshTiam1Thy1Tgfb1i1Tnfrsf12aNbl1PtgisCol1a1GrnAsb4Nupr1Fgf7C3ar1Sfrp4FrzbRunx1Pmp22Pdlim5Timp1Tgfb1RT1-DMaOmdEgln1Gnrh1Bmp1Nfam1Csf1Ptgs2Tmem176bFigfPthlhSerpine2Chi3l1Xylt1Thbs4Nrxn1Srpx2Tbx3Runx2RelnPrkcbSulf1Csf1rTfDab2Tnfrsf11bAxlFzd2Cd44C3Lpar1PpargEcm1Cx3cr1Ccl2Itm2cTrim16AspaC1qcCcr5Fgf18Ncam1Nckap1lThbs1Bmp6 |
| [GO:0040011 locomotion](http://amigo.geneontology.org/cgi-bin/amigo/term_details?term=GO:0040011&session_id=) | 6.38e-09 | Tgfb2Loxl2Sema5aVcanEpha7CtgfVtnItgamMmp2Cxcl13ApoeSfrp2PtafrC5ar1PtprjFapLrp1Spp1Cysltr1Dpysl3Sfrp1LbpLamc1Dnah5Ccr1Fgfr1CtshP2ry6Thy1AcanTnfrsf12aSlit3Ccl6Nbl1EnahCol1a1Tp53inp1Col18a1Fgf7Lsp1C3ar1Cdh13Timp1Tgfb1Csf1Ptgs2FigfP2ry12Thbs4Srpx2Dpp4RelnP2ry1Sulf1Nlrp10Etv1Fcer1gCsf1rAtp1a2TfDab2Itga11AxlCd44SelplgFn1C3Lpar1Ecm1Lrrc16aHas2Cx3cr1Ccl2MdkNr4a3Ifitm1Ddr1Mmp14Ccr5Abhd2Sorl1Nckap1lThbs1 |
| [GO:0048513 organ development](http://amigo.geneontology.org/cgi-bin/amigo/term_details?term=GO:0048513&session_id=) | 1.02e-08 | Ltbp3Tgfb2Loxl2VcanEpha7Agpat2CtgfTmem176aBokCd28Gnao1Fstl3Ccnd1Mmp2Mgst1Cxcl13Fkbp4Bcat2InhbaAcadmSfrp2C5ar1Slc22a5Dgat2MafbPtprjTrdnAspnFgfr2MgpLumEtv5Spp1Ifi204Ptcd2Serpinf1Sfrp1Tspan12Col1a2Loxl3Ccr1Fgfr1CtshVwfFbn1Thy1ChadCol3a1Creb3l2AcanTgfb1i1Ucp2Slit3Zfp238FlnaCol1a1Nt5eIl18Prrx2Unc93b1Nupr1Fgf7Aldh1a1TaglnTgm1C1qbKySfrp4Cacna2d2Fcgr1aFrzbRunx1GpnmbPdlim5Timp1Tgfb1RT1-DMaOmdEgln1Gnrh1Pfkfb1Bmp1GamtNfam1Csf1Cd4Ptgs2Tmem176bCyp4b1Lcp1Irx4PthlhSerpine2Chi3l1Plscr1Hsd11b1NppaStc2Sulf2Pls3Tbx3Runx2RelnPrkcbP2ry1Sulf1AldobG6pdEtv1Smoc1Smad7Fcer1gCol8a1Csf1rElnMregTfAlx3Dab2FmodAnxa1Tnfrsf11bGpr183AxlMmeFzd2CnpCd44Prrx1CpDkk3Cdh22CompPpargEcm1Has2Cx3cr1Ildr2Ccl2Casp7Lpin1Trim16MdkNr4a3LoxTlr5Ddr1Car4C1qcC1qtnf5LOC681309Mmp14Ccr5Fgf18CtssNcam1Nckap1lThbs1Tgm2Bmp6 |
| [GO:0006952 defense response](http://amigo.geneontology.org/cgi-bin/amigo/term_details?term=GO:0006952&session_id=) | 1.42e-08 | Cd28Cxcl13ApoeCfhPolr3gPtafrC5ar1GpamCybbSpp1C6Cd55Cysltr1Serpinf1LbpZyxCd46Ccr1Cotl1HampClec7aPtgisS1pr3Nt5eIl18PrkcdUnc93b1Olr1Nupr1Lsp1C1qbFcgr1aCd14Tlr7Tgfb1Alox5Csf1Cd4Ptgs2Chi3l1Plscr1Ephx2Lgals3Serping1Ncf1Lyz2Nlrp10Fcer1gCsf1rTfAnxa1AxlC1qaCd44AoahFn1C3PpargEcm1Alox5apCcl2MdkIfitm1Exosc5C1qcC1sCcr5Apobec1Thbs1Tgm2 |
| [GO:0048518 positive regulation of biological process](http://amigo.geneontology.org/cgi-bin/amigo/term_details?term=GO:0048518&session_id=) | 1.57e-08 | Ltbp3Tgfb2Timp2Loxl2Epha7Agpat2Sec16bAbcg1Adcy7CtgfFhl1BokVtnCd28Fstl3Ccnd1Mmp2Cxcl13ApoeInhbaCfhPolr3gSfrp2PtafrFndc5C5ar1Slc22a5MafbGpamPtprjCacna1gFgfr2LumEtv5Ptgs1Lrp1Spp1Ifi204C6Msr1Cd55Bex1Cysltr1Elk1Dpysl3Serpinf1Gpihbp1Sfrp1LbpSoat1Fbln2Fndc1Cd46Tfb2mCcr1Fgfr1CtshTiam1P2ry6Thy1Creb3l2Zfp462Tgfb1i1Tnfrsf12aClec7aHfe2AplnUcp2Nbl1PtgisFlnaClec11aCol1a1Myo3bTrpc6Nt5eAcsl1GrnAsb4Il18Htr2aPrkcdTbxas1Prrx2Unc93b1Nupr1Tp53inp1Col18a1Rad23aFgf7Crlf1Aldh1a1Casp12Fndc3bMlycdC1qbC3ar1Sfrp4Cacna2d2Fcgr1aCd14FrzbRunx1Tlr7LitafCdh13Tgfb1RT1-DMaAnxa2Bmp1Nfam1Map2k6Alox5Csf1Cd4Acox1Ptgs2FigfP2ry12PthlhEmilin1Serpine2Chi3l1Scn3bPlscr1Thbs4Nrxn1Pcbd1Fitm1Sulf2Srpx2Ephx2Dpp4Piwil2Tbx3Runx2RelnPrkcbP2ry1Abca1Serping1Pdcd1lg2Sulf1Nlrp10Mid1ip1Etv1Smoc1Smad7Fcer1gCol8a1Csf1rTfDab2Anxa1Gpr183Phlda3AxlC1qaFzd2Cd44Prrx1Scn4bC3Lpar1PpargEcm1Has2Cx3cr1Alox5apCcl2Itm2cCasp7Lpin1Trim16MdkNr4a3S100a4AspaIfitm1Tlr5C1qcMmp14C1sCcr5Apobec1Avpi1OsmrFgf18Ncam1Nckap1lThbs1Tgm2Bmp6 |
| [GO:0051216 cartilage development](http://amigo.geneontology.org/cgi-bin/amigo/term_details?term=GO:0051216&session_id=) | 1.63e-08 | Ltbp3Tgfb2Loxl2CtgfSfrp2MgpLumFgfr1ChadCreb3l2AcanCol1a1Prrx2FrzbTimp1Tgfb1Bmp1PthlhSulf2Runx2Sulf1Cd44Prrx1CompLOC681309Fgf18Thbs1Bmp6 |
| [GO:0002682 regulation of immune system process](http://amigo.geneontology.org/cgi-bin/amigo/term_details?term=GO:0002682&session_id=) | 1.94e-08 | Tgfb2Fcgr2aTmem176aCd28Fstl3Cxcl13InhbaCfhPolr3gPtafrC5ar1MafbGpamPtprjIfi204C6Cd55Sfrp1LbpCd46Ccr1CtshThy1Col3a1Clec7aNbl1Il18Unc93b1C1qbC3ar1Fcgr1aRunx1Tlr7Tgfb1RT1-DMaNfam1Csf1Cd4Tmem176bFigfPlscr1Thbs4Dpp4PrkcbSerping1Pdcd1lg2Nlrp10Tnfsf18Fcer1gCsf1rAnxa1Tnfrsf11bGpr183AxlC1qaCd44C3PpargEcm1Ccl2Tlr5C1qcC1sNckap1lThbs1 |
| [GO:0006955 immune response](http://amigo.geneontology.org/cgi-bin/amigo/term_details?term=GO:0006955&session_id=) | 2.27e-08 | Tgfb2Fcgr2aVtnCd28Cxcl13CfhPolr3gPtafrC5ar1Prg4CybbIfi204C6Cd55LbpCd46Ccr1CtshThy1Col3a1Clec7aCcl6RT1-M1-2Il18PrkcdUnc93b1C1qbC3ar1Fcgr1aCd14Tlr7Tgfb1RT1-DMaNfam1Csf1Cd4Lcp1Plscr1Dpp4PrkcbLgals3Serping1Nlrp10Tnfsf18Fcer1gCsf1rTyrobpGpr183AxlC1qaC3PpargEcm1Cx3cr1Ccl2Ifitm1C1qcC1sCcr5CtssNckap1lThbs1Sectm1bBmp6 |
| [GO:0042493 response to drug](http://amigo.geneontology.org/cgi-bin/amigo/term_details?term=GO:0042493&session_id=) | 3.62e-08 | Tgfb2Grk5Timp2Aqp7Ltc4sEno2Gnao1Ccnd1Atp1a3Mmp2Mgst1AcadmSfrp2Slc22a5HadhaCybbSfrp1Cyp2e1Abcb4AcanAcacbAcsl1Htr2aPrkcdSlc38a3Col18a1Txn2Aldh1a1Casp12LipeAcot2Tgfb1Map2k6Ptgs2Cyp4b1RelnPrkcbAbca1AldobGpx3Abcd3Anxa1Tnfrsf11bPpargCcl2Slc22a3MdkLoxCar4GabreHadhApobec1Htr1bNckap1lThbs1 |
| [GO:0048584 positive regulation of response to stimulus](http://amigo.geneontology.org/cgi-bin/amigo/term_details?term=GO:0048584&session_id=) | 5.93e-08 | Tgfb2Timp2CtgfCd28Cxcl13InhbaCfhPolr3gSfrp2PtafrC5ar1PtprjFgfr2Ifi204C6Cd55Sfrp1LbpCd46Ccr1Fgfr1Thy1Tgfb1i1Tnfrsf12aClec7aPtgisFlnaCol1a1Htr2aPrkcdPrrx2Unc93b1Crlf1C1qbC3ar1Sfrp4Fcgr1aTlr7LitafCdh13Tgfb1RT1-DMaNfam1Map2k6Csf1Cd4Ptgs2FigfChi3l1Plscr1Thbs4Sulf2RelnPrkcbP2ry1Serping1Sulf1Nlrp10Fcer1gCsf1rTfDab2Gpr183AxlC1qaCd44Prrx1C3Lpar1Ecm1Alox5apCcl2Trim16S100a4Tlr5C1qcC1sCcr5Avpi1Fgf18Ncam1Nckap1lThbs1Tgm2Bmp6 |
| [GO:0016477 cell migration](http://amigo.geneontology.org/cgi-bin/amigo/term_details?term=GO:0016477&session_id=) | 8.24e-08 | Tgfb2Loxl2VcanCtgfVtnItgamMmp2Cxcl13ApoeSfrp2C5ar1PtprjFapLrp1Spp1Dpysl3Sfrp1LbpLamc1Ccr1Fgfr1CtshP2ry6Thy1AcanTnfrsf12aCcl6Nbl1Col1a1Tp53inp1Col18a1Fgf7C3ar1Cdh13Timp1Tgfb1Csf1Ptgs2FigfP2ry12Thbs4Srpx2Dpp4RelnP2ry1Sulf1Nlrp10Fcer1gCsf1rDab2Itga11AxlCd44SelplgFn1Lpar1Ecm1Lrrc16aCx3cr1Ccl2MdkIfitm1Ddr1Mmp14Ccr5Abhd2Sorl1Nckap1lThbs1 |
| [GO:0006629 lipid metabolic process](http://amigo.geneontology.org/cgi-bin/amigo/term_details?term=GO:0006629&session_id=) | 9.93e-08 | Impa2Sptlc2Agpat2Abcg1Acsf3Ltc4sEhhadhApoeAcadmDgat2HadhaGpamPtgs1Lrp1MecrFads1Soat1Cyp2e1Ucp3AcadvlHpgdsGpcpd1Acsf2PtgisAcacbScpep1Nt5eAcsl1Akr1b10Htr2aDhrs4PrkcdTbxas1Fgf7Aldh1a1MlycdPdk3Cpt2Ankrd23LipeHadhbDgat1Timp1Acot2Pla2g2dTgfb1Alox5Acox1Ptgs2Decr1Plscr1Hsd11b1Agpat9Eci1Ephx2Fads2Abca1Ncf1G6pdMid1ip1Csf1rAbcd3Fdft1Anxa1Ch25hAoahDkk3C3Pdk4PpargAlox5apLpin1AcadsAsah2HadhEch1CratAcot7Bmp6 |
| [GO:0051179 localization](http://amigo.geneontology.org/cgi-bin/amigo/term_details?term=GO:0051179&session_id=) | 1.21e-07 | Cadm3Tgfb2Loxl2VcanHcn4Scn1aSec16bAbcg1CtgfFhl1Aqp7VtnGnao1ItgamCcnd1Atp1a3Mmp2Cxcl13Fkbp4ApoeBcat2InhbaHook1Sfrp2Slc16a7Kcnk2C5ar1Slc22a5Dgat2GpamSlc25a15PtprjTrdnCacna1gFapFgfr2Etv5Ptgs1Pea15aLrp1Spp1Erc1Fxyd2Msr1Cysltr1Dpysl3Gpihbp1Sfrp1LbpSoat1Lamc1Clcn1Fndc1Ucp3Abcb4Dnah5Ccr1Fgfr1Rab3il1CtshGot2Npr3P2ry6Thy1Creb3l2AcanTnfrsf12aClec7aAplnUcp2Mrc2Mapk8ip1Ccl6Nbl1EnahFlnaCol1a1Pex19Osbpl6Trpc6Acsl1GrnIl18Htr2aPrkcdUnc93b1Slc38a3Tp53inp1Col18a1Fgf7Slc25a20Slc46a3Mrc1Slc6a6C3ar1Cpt2Sfrp4Atp6ap1lCacna2d2Slc25a34Fcgr1aCd14Runx1Tlr7LitafMyo5bCyth4Cdh13Dgat1Timp1Tgfb1Sv2cRT1-DMaS100a6Slc16a10Anxa2CorinCsf1Dhrs7cCd4Ptgs2FigfP2ry12Lcp1Scara5Serpine2Chi3l1Scn3bPlscr1Elmo1Thbs4Nrxn1Fitm1Stc2Dbn1Srpx2RilpDpp4Tbx3RelnPrkcbP2ry1Abca1Sulf1Nlrp10Smad7Fcer1gCsf1rAtp1a2Abcd3Kcnt2Uchl1Rab23TfDab2Anxa1Itga11AxlCnpCd44Scrn1CpSelplgTmed3Scn4bFn1C3Lpar1PpargEcm1Lrrc16aRab31LOC100361596Has2Cx3cr1Ildr2PllpAdhfe1Ccl2Slc22a3Slc4a1Piezo2Trim16MdkIfitm1Ddr1Car4Slc25a42Abca5C1qtnf5Mmp14GabreHadhCcr5Apobec1Abhd2Htr1bActn1Sorl1Slc43a2Fxyd3CratNcam1Nckap1lThbs1Tgm2 |
| [GO:0048514 blood vessel morphogenesis](http://amigo.geneontology.org/cgi-bin/amigo/term_details?term=GO:0048514&session_id=) | 1.23e-07 | Loxl2Sema5aCtgfMmp2ApoeSfrp2PtprjFgfr2Lrp1C6Cysltr1Serpinf1Tspan12Fgfr1CtshThy1PtgisAsb4Il18Prrx2Col18a1C3ar1Runx1Cdh13Egln1Anxa2Ptgs2FigfChi3l1Thbs4Nrxn1Srpx2PrkcbSulf1Smad7Sat1Tmem100Prrx1Fn1C3Ecm1Cx3cr1Ccl2Mmp14Fgf18Thbs1 |
| [GO:0048870 cell motility](http://amigo.geneontology.org/cgi-bin/amigo/term_details?term=GO:0048870&session_id=) | 1.41e-07 | Tgfb2Loxl2VcanCtgfVtnItgamMmp2Cxcl13ApoeSfrp2C5ar1PtprjFapLrp1Spp1Dpysl3Sfrp1LbpLamc1Dnah5Ccr1Fgfr1CtshP2ry6Thy1AcanTnfrsf12aCcl6Nbl1Col1a1Tp53inp1Col18a1Fgf7C3ar1Cdh13Timp1Tgfb1Csf1Ptgs2FigfP2ry12Thbs4Srpx2Dpp4RelnP2ry1Sulf1Nlrp10Fcer1gCsf1rTfDab2Itga11AxlCd44SelplgFn1Lpar1Ecm1Lrrc16aHas2Cx3cr1Ccl2MdkIfitm1Ddr1Mmp14Ccr5Abhd2Sorl1Nckap1lThbs1 |
| [GO:0051674 localization of cell](http://amigo.geneontology.org/cgi-bin/amigo/term_details?term=GO:0051674&session_id=) | 1.41e-07 | Tgfb2Loxl2VcanCtgfVtnItgamMmp2Cxcl13ApoeSfrp2C5ar1PtprjFapLrp1Spp1Dpysl3Sfrp1LbpLamc1Dnah5Ccr1Fgfr1CtshP2ry6Thy1AcanTnfrsf12aCcl6Nbl1Col1a1Tp53inp1Col18a1Fgf7C3ar1Cdh13Timp1Tgfb1Csf1Ptgs2FigfP2ry12Thbs4Srpx2Dpp4RelnP2ry1Sulf1Nlrp10Fcer1gCsf1rTfDab2Itga11AxlCd44SelplgFn1Lpar1Ecm1Lrrc16aHas2Cx3cr1Ccl2MdkIfitm1Ddr1Mmp14Ccr5Abhd2Sorl1Nckap1lThbs1 |
| [GO:0061448 connective tissue development](http://amigo.geneontology.org/cgi-bin/amigo/term_details?term=GO:0061448&session_id=) | 1.84e-07 | Ltbp3Tgfb2Loxl2CtgfSfrp2Dgat2MgpLumFgfr1ChadCreb3l2AcanCol1a1Prrx2FrzbTimp1Tgfb1Bmp1Csf1PthlhSulf2Runx2Sulf1Cd44Prrx1CompLOC681309Fgf18Thbs1Bmp6 |
| [GO:0072359 circulatory system development](http://amigo.geneontology.org/cgi-bin/amigo/term_details?term=GO:0072359&session_id=) | 2.14e-07 | Tgfb2Loxl2Sema5aVcanCtgfMmp2ApoeAcadmSfrp2Slc22a5PtprjFgfr2Lrp1Ptcd2C6Cysltr1Serpinf1Sfrp1Tspan12Col1a2Fgfr1CtshFbn1Thy1Col3a1PtgisCol1a1Asb4Il18Prrx2Col18a1C3ar1Sfrp4Runx1Pdlim5Cdh13Egln1Anxa2Ptgs2FigfIrx4Chi3l1NppaThbs4Nrxn1Srpx2Tbx3PrkcbSulf1Smad7Sat1Tmem100Fzd2Cd44Prrx1Fn1C3PpargEcm1Cx3cr1Ccl2Casp7LoxMmp14Fgf18Ncam1Thbs1 |
| [GO:0072358 cardiovascular system development](http://amigo.geneontology.org/cgi-bin/amigo/term_details?term=GO:0072358&session_id=) | 2.14e-07 | Tgfb2Loxl2Sema5aVcanCtgfMmp2ApoeAcadmSfrp2Slc22a5PtprjFgfr2Lrp1Ptcd2C6Cysltr1Serpinf1Sfrp1Tspan12Col1a2Fgfr1CtshFbn1Thy1Col3a1PtgisCol1a1Asb4Il18Prrx2Col18a1C3ar1Sfrp4Runx1Pdlim5Cdh13Egln1Anxa2Ptgs2FigfIrx4Chi3l1NppaThbs4Nrxn1Srpx2Tbx3PrkcbSulf1Smad7Sat1Tmem100Fzd2Cd44Prrx1Fn1C3PpargEcm1Cx3cr1Ccl2Casp7LoxMmp14Fgf18Ncam1Thbs1 |
| [GO:0065008 regulation of biological quality](http://amigo.geneontology.org/cgi-bin/amigo/term_details?term=GO:0065008&session_id=) | 3.57e-07 | Ltbp3Tgfb2Scn1aAbcg1Fhl1Aqp7Ccnd1Mmp2Cxcl13ApoeBcat2InhbaQsox1C5ar1Slc22a5Dgat2MafbGpamPtprjTrdnCacna1gEtv5Ptgs1Spp1Cd55Cysltr1Gpihbp1Sfrp1Col1a2AcadvlActr3bCcr1F13a1CtshHampVwfNpr3Thy1Tnfrsf12aHfe2AplnUcp2Nbl1Efemp2Zfp238FlnaPex19Scpep1Trpc6Akr1b10Il18Htr2aPrkcdMlxiplTbxas1Arpc1bCapgTxn2Fgf7Aldh1a1C3ar1Sfrp4Runx1Tlr7Pmp22Pdlim5Dgat1Timp1Tgfb1Egln1Anxa2CorinAlox5Csf1Dhrs7cCd4Acox1Ptgs2P2ry12Scara5PthlhSerpine2Scn3bPlscr1NppaNrxn1Fitm1Stc2Dbn1Ephx2Tbx3RelnPrkcbGas7P2ry1Abca1Serping1Ncf1G6pdGna15Smad7CtskFcer1gCsf1rAtp1a2ElnSypl2TfMaoaCpeb1Anxa1Tnfrsf11bAxlFzd2CnpCd44CpDkk3Entpd1Slc26a5Scn4bFn1C3Lpar1PpargLOC100361596Has2Ildr2PllpCcl2Slc22a3Piezo2AspaDdr1HadhCcr5Apobec1CtssNckap1lThbs1Tgm2Bmp6 |
| [GO:0032879 regulation of localization](http://amigo.geneontology.org/cgi-bin/amigo/term_details?term=GO:0032879&session_id=) | 4.88e-07 | Tgfb2Scn1aSec16bAbcg1Fhl1VtnGnao1Cxcl13ApoeInhbaSfrp2Kcnk2GpamPtprjTrdnCacna1gPtgs1Pea15aLrp1Msr1Dpysl3Sfrp1LbpFndc1Ccr1CtshP2ry6Thy1AcanClec7aAplnUcp2Nbl1FlnaCol1a1Trpc6Il18Htr2aPrkcdTp53inp1Col18a1Fgf7C3ar1Sfrp4Fcgr1aCd14Runx1Tlr7LitafMyo5bCdh13Timp1Tgfb1Anxa2CorinCsf1Dhrs7cPtgs2FigfP2ry12Lcp1Serpine2Scn3bThbs4Nrxn1Fitm1Stc2Srpx2RelnPrkcbP2ry1Abca1Sulf1Nlrp10Fcer1gCsf1rAtp1a2TfDab2Anxa1Scn4bC3Lpar1PpargEcm1Has2Cx3cr1Ccl2Trim16Ifitm1Mmp14HadhCcr5Abhd2Htr1bActn1Ncam1Nckap1lThbs1 |
| [GO:0002684 positive regulation of immune system process](http://amigo.geneontology.org/cgi-bin/amigo/term_details?term=GO:0002684&session_id=) | 5.06e-07 | Tgfb2Cd28Cxcl13CfhPolr3gPtafrC5ar1GpamPtprjIfi204C6Cd55LbpCd46Ccr1Thy1Clec7aIl18Unc93b1C1qbC3ar1Fcgr1aTlr7Tgfb1RT1-DMaNfam1Cd4FigfPlscr1Thbs4Dpp4PrkcbSerping1Pdcd1lg2Nlrp10Fcer1gGpr183AxlC1qaC3Ccl2Tlr5C1qcC1sNckap1lThbs1 |
| [GO:0009725 response to hormone stimulus](http://amigo.geneontology.org/cgi-bin/amigo/term_details?term=GO:0009725&session_id=) | 6.60e-07 | Tgfb2CtgfEno2Ccnd1Mmp2ApoeInhbaAcadmBckdhaHadhaGpamCacna1gMgpPtgs1Spp1Cd55Fads1Serpinf1Sfrp1Ucp3Abcb4CtshPlod2Ucp2Slit3Col1a1GrnIl18PrkcdTxn2Aldh1a1C1qbSfrp4Cuzd1Timp1Acot2Tgfb1Gnrh1Pfkfb1Ptgs2Hsd11b1NppaStc2RelnPrkcbAldobGpx3TfAnxa1Tnfrsf11bFzd2Fn1C3Pdk4PpargCcl2Lpin1Trim16MdkNr4a3LoxAcadsCar4Mmp14HadhApobec1Htr1bCtssThbs1Bmp6 |
| [GO:0009653 anatomical structure morphogenesis](http://amigo.geneontology.org/cgi-bin/amigo/term_details?term=GO:0009653&session_id=) | 7.41e-07 | Ltbp3Tgfb2Loxl2Sema5aVcanEpha7CtgfRaph1Ccnd1Mmp2ApoeInhbaSfrp2C5ar1MafbPtprjAspnFgfr2MgpEtv5Lrp1Spp1Ptcd2Antxr1C6Cysltr1Serpinf1Sfrp1Tspan12Col1a2Lamc1Loxl3Igsf10Fgfr1CtshTiam1VwfThy1ChadAcanTgfb1i1Tnfrsf12aUcp2Slit3Nbl1EnahPtgisFlnaCol1a1Asb4Il18Prrx2Col18a1Fgf7Aldh1a1Sdc2Tgm1C3ar1Sfrp4FrzbRunx1Pmp22Pdlim5Cdh13Tgfb1Egln1Pfkfb1Anxa2GamtCsf1Ptgs2FigfLcp1Irx4PthlhSerpine2Chi3l1Xylt1Thbs4Nrxn1Srpx2Tbx3Runx2RelnPrkcbGas7Sulf1Etv1Smad7Vat1Col8a1Csf1rSat1Uchl1Tmem100Rab23Alx3Dab2FmodTnfrsf11bAxlFzd2CnpCd44Prrx1Slc26a5Fn1C3CompPpargEcm1Cx3cr1Ccl2Lpin1Nr4a3Ifitm1Ddr1LOC681309Mmp14Ccr5Aff3Ninj2Fgf18Ncam1Thbs1Tgm2Bmp6 |
| [GO:0051094 positive regulation of developmental process](http://amigo.geneontology.org/cgi-bin/amigo/term_details?term=GO:0051094&session_id=) | 8.93e-07 | Tgfb2Timp2Loxl2CtgfApoeInhbaSfrp2Fndc5Fgfr2Etv5Ifi204C6Msr1Bex1Cysltr1Serpinf1Sfrp1Ccr1Fgfr1CtshTiam1Tgfb1i1Tnfrsf12aNbl1PtgisCol1a1GrnAsb4Fndc3bC3ar1Sfrp4FrzbRunx1Tgfb1RT1-DMaBmp1Csf1Ptgs2FigfSerpine2Chi3l1Nrxn1Srpx2Runx2RelnPrkcbCsf1rDab2AxlC3Lpar1PpargEcm1Cx3cr1Trim16AspaIfitm1Ccr5Fgf18Ncam1Nckap1lThbs1Bmp6 |
| [GO:0048583 regulation of response to stimulus](http://amigo.geneontology.org/cgi-bin/amigo/term_details?term=GO:0048583&session_id=) | 1.14e-06 | Tgfb2Grk5Timp2Fcgr2aEpha7CtgfCd28Fstl3Ccnd1Cxcl13ApoeInhbaCfhPolr3gSfrp2PtafrC5ar1MyofPtprjAspnFgfr2Lrp1Spp1Ifi204C6Cd55Serpinf1Sfrp1LbpZyxCd46Ccr1Fgfr1CtshThy1Col3a1Tgfb1i1Tnfrsf12aClec7aUcp2Slit3Mapk8ip1Nbl1PtgisFlnaCol1a1Nt5eIl18Htr2aPrkcdPrrx2Unc93b1Crlf1C1qbC3ar1Sfrp4Fcgr1aFrzbRunx1Tlr7LitafCyth4Cdh13Tgfb1RT1-DMaAnxa2Nfam1Map2k6Csf1Cd4Ptgs2FigfSerpine2Chi3l1Adamtsl2Plscr1Xylt1Thbs4Nrxn1Agpat9Sulf2Dpp4PostnRunx2RelnPrkcbP2ry1Abca1Serping1Sulf1Nlrp10Smad7Fcer1gCsf1rUchl1Rab23TfDab2Anxa1Gpr183Phlda3AxlC1qaCd44AoahPrrx1Dkk3C3Lpar1PpargEcm1Alox5apCcl2Trim16MdkS100a4CilpTlr5Ddit4lC1qcC1sCcr5Htr1bAvpi1Fgf18Ncam1Nckap1lThbs1Tgm2Bmp6 |
| [GO:0001525 angiogenesis](http://amigo.geneontology.org/cgi-bin/amigo/term_details?term=GO:0001525&session_id=) | 1.19e-06 | Loxl2Sema5aCtgfMmp2Sfrp2Fgfr2C6Cysltr1Serpinf1Tspan12Fgfr1CtshThy1PtgisIl18Col18a1C3ar1Runx1Cdh13Egln1Anxa2Ptgs2FigfChi3l1Thbs4Nrxn1Srpx2PrkcbSulf1Sat1Tmem100Fn1C3Ecm1Cx3cr1Ccl2Mmp14Fgf18Thbs1 |
| [GO:0009888 tissue development](http://amigo.geneontology.org/cgi-bin/amigo/term_details?term=GO:0009888&session_id=) | 1.55e-06 | Ltbp3Tgfb2Loxl2Sema5aAgpat2CtgfCcnd1Mmp2InhbaAcadmSfrp2Dgat2TrdnAspnFgfr2MgpLumEtv5Spp1Ptcd2Sfrp1Col1a2Loxl3Ccr1Igsf10Fgfr1CtshVwfChadCol3a1Creb3l2AcanTgfb1i1Zfp238EnahFlnaCol1a1Prrx2Unc93b1Nupr1Col18a1Fgf7Aldh1a1Tgm1KySfrp4Cacna2d2Fcgr1aFrzbRunx1GpnmbTimp1Tgfb1RT1-DMaOmdEgln1Bmp1Csf1Ptgs2Cyp4b1PthlhSerpine2Stc2Sulf2Tbx3PostnRunx2Sulf1Smad7Fcer1gCsf1rElnTmem100MregDab2Anxa1AxlFzd2Cd44Prrx1CompPpargEcm1Trim16Nr4a3Ddr1LOC681309Ninj2Fgf18Ncam1Thbs1Tgm2Bmp6 |
| [GO:0006928 cellular component movement](http://amigo.geneontology.org/cgi-bin/amigo/term_details?term=GO:0006928&session_id=) | 1.78e-06 | Tgfb2Loxl2VcanCtgfVtnItgamMmp2Cxcl13ApoeSfrp2C5ar1PtprjFapLrp1Spp1Dpysl3Sfrp1LbpLamc1Dnah5Ccr1Fgfr1CtshP2ry6Thy1AcanTnfrsf12aCcl6Nbl1EnahCol1a1Tp53inp1Col18a1Fgf7C3ar1Asb14Cdh13Timp1Tgfb1Tubb6Csf1Ptgs2FigfP2ry12Elmo1Thbs4Srpx2Dpp4RelnP2ry1Sulf1Nlrp10Fcer1gCsf1rAtp1a2Uchl1TfDab2Itga11AxlCd44SelplgFn1Lpar1Ecm1Lrrc16aHas2Cx3cr1Ccl2MdkIfitm1Ddr1Mmp14Ccr5Abhd2Actn1Sorl1Nckap1lThbs1 |
| [GO:0051270 regulation of cellular component movement](http://amigo.geneontology.org/cgi-bin/amigo/term_details?term=GO:0051270&session_id=) | 1.88e-06 | Tgfb2VtnCxcl13ApoeSfrp2PtprjLrp1Dpysl3Sfrp1LbpCcr1CtshP2ry6Thy1AcanNbl1Col1a1Tp53inp1Col18a1Fgf7C3ar1Cdh13Timp1Tgfb1Csf1Ptgs2FigfScn3bThbs4Srpx2Sulf1Csf1rAtp1a2TfDab2Scn4bLpar1Ecm1Has2Cx3cr1Ccl2Ifitm1Mmp14Ccr5Abhd2Actn1Nckap1lThbs1 |
| [GO:0032101 regulation of response to external stimulus](http://amigo.geneontology.org/cgi-bin/amigo/term_details?term=GO:0032101&session_id=) | 2.00e-06 | Cd28Cxcl13ApoeCfhPtprjSpp1C6Cd55Serpinf1LbpZyxCd46Ccr1Nbl1PtgisNt5ePrkcdC3ar1Fcgr1aCdh13Tgfb1Anxa2Ptgs2FigfSerpine2Xylt1Thbs4Nrxn1Serping1Fcer1gAnxa1AoahC3Lpar1PpargAlox5apCcl2Ccr5Nckap1lThbs1Tgm2 |
| [GO:0009062 fatty acid catabolic process](http://amigo.geneontology.org/cgi-bin/amigo/term_details?term=GO:0009062&session_id=) | 2.96e-06 | EhhadhAcadmHadhaAcadvlMlycdCpt2HadhbAcot2Acox1Decr1Eci1Abcd3Lpin1AcadsHadhEch1Acot7 |
| [GO:0014070 response to organic cyclic compound](http://amigo.geneontology.org/cgi-bin/amigo/term_details?term=GO:0014070&session_id=) | 3.51e-06 | Tgfb2CtgfEno2Gnao1Ccnd1Mmp2ApoeInhbaAcadmBckdhaTrdnCacna1gMgpLumPtgs1Pea15aSpp1Fads1Serpinf1Sfrp1Ucp3Abcb4AcanSlit3AcacbCol1a1Acsl1GrnIl18PrkcdTxn2Aldh1a1Casp12Sdc2C1qbSfrp4Tgfb1Gnrh1Pfkfb1Ptgs2Hsd11b1Stc2RelnPrkcbAbca1AldobG6pdGpx3Atp1a2TfAnxa1Tnfrsf11bFzd2Cd44Fn1C3PpargCcl2MdkLoxAcadsCar4Mmp14C1sCcr5Htr1bNcam1Thbs1Bmp6 |
| [GO:0072329 monocarboxylic acid catabolic process](http://amigo.geneontology.org/cgi-bin/amigo/term_details?term=GO:0072329&session_id=) | 3.63e-06 | EhhadhAcadmPon3HadhaAcadvlMlycdCpt2HadhbAcot2Acox1Decr1Eci1Abcd3Lpin1AcadsHadhEch1Acot7 |
| [GO:0050776 regulation of immune response](http://amigo.geneontology.org/cgi-bin/amigo/term_details?term=GO:0050776&session_id=) | 5.38e-06 | Tgfb2Fcgr2aCd28CfhPolr3gPtafrC5ar1PtprjIfi204C6Cd55LbpCd46Ccr1CtshThy1Col3a1Clec7aUnc93b1C1qbC3ar1Fcgr1aTlr7Tgfb1RT1-DMaNfam1Plscr1Dpp4PrkcbSerping1Nlrp10Fcer1gC1qaC3PpargEcm1Tlr5C1qcC1sNckap1l |
| [GO:0022603 regulation of anatomical structure morphogenesis](http://amigo.geneontology.org/cgi-bin/amigo/term_details?term=GO:0022603&session_id=) | 5.77e-06 | Tgfb2ApoeSfrp2AspnFgfr2Etv5Spp1C6Cysltr1Serpinf1Sfrp1Tspan12Fgfr1CtshTiam1Thy1Tgfb1i1Tnfrsf12aPtgisCol1a1Asb4Il18Fgf7C3ar1Runx1Pdlim5Tgfb1Egln1Csf1Ptgs2FigfChi3l1Xylt1Thbs4Srpx2Runx2RelnPrkcbGas7Sulf1Vat1Csf1rDab2Tnfrsf11bFzd2Cd44Slc26a5Fn1C3Ecm1Cx3cr1Ccl2Ccr5Thbs1 |
| [GO:2000145 regulation of cell motility](http://amigo.geneontology.org/cgi-bin/amigo/term_details?term=GO:2000145&session_id=) | 5.87e-06 | Tgfb2VtnCxcl13ApoeSfrp2PtprjLrp1Dpysl3Sfrp1LbpCcr1CtshP2ry6Thy1AcanNbl1Col1a1Tp53inp1Col18a1Fgf7C3ar1Cdh13Timp1Tgfb1Csf1Ptgs2FigfThbs4Srpx2Sulf1Csf1rTfDab2Lpar1Ecm1Has2Cx3cr1Ccl2Ifitm1Mmp14Ccr5Abhd2Nckap1lThbs1 |
| [GO:0044282 small molecule catabolic process](http://amigo.geneontology.org/cgi-bin/amigo/term_details?term=GO:0044282&session_id=) | 6.37e-06 | Impa2Acsf3EhhadhApoeBcat2AcadmPon3BckdhaHadhaAcadvlGot2Akr1b10PrkcdMlycdCpt2HadhbAcot2Acox1Decr1Eci1ProdhBcat1Abcd3MaoaLpin1AcadsHadhEch1Acot7Gpt |
| [GO:0044712 single-organism catabolic process](http://amigo.geneontology.org/cgi-bin/amigo/term_details?term=GO:0044712&session_id=) | 6.37e-06 | Impa2Acsf3EhhadhApoeBcat2AcadmPon3BckdhaHadhaAcadvlGot2Akr1b10PrkcdMlycdCpt2HadhbAcot2Acox1Decr1Eci1ProdhBcat1Abcd3MaoaLpin1AcadsHadhEch1Acot7Gpt |
| [GO:0002376 immune system process](http://amigo.geneontology.org/cgi-bin/amigo/term_details?term=GO:0002376&session_id=) | 8.49e-06 | Tgfb2Fcgr2aTmem176aVtnCd28ItgamFstl3Cxcl13InhbaCfhPolr3gSfrp2PtafrC5ar1MafbPrg4GpamCybbPtprjFgfr2Spp1Ifi204C6Cd55Sfrp1LbpCd46Ccr1CtshThy1Col3a1Clec7aCcl6Nbl1RT1-M1-2Il18PrkcdUnc93b1C1qbC3ar1Fcgr1aCd14Runx1Tlr7Timp1Tgfb1RT1-DMaNfam1Csf1Cd4Tmem176bFigfLcp1Plscr1Thbs4Dpp4Runx2PrkcbLgals3Serping1Ncf1Pdcd1lg2G6pdNlrp10Tnfsf18Fcer1gCsf1rTyrobpTfAnxa1Tnfrsf11bGpr183AxlC1qaCd44SelplgC3PpargEcm1Cx3cr1Ccl2Ifitm1Tlr5Exosc5C1qcC1sCcr5Apobec1CtssNckap1lThbs1Sectm1bBmp6 |
| [GO:0002673 regulation of acute inflammatory response](http://amigo.geneontology.org/cgi-bin/amigo/term_details?term=GO:0002673&session_id=) | 9.10e-06 | CfhC6Cd55Cd46Ccr1Fcgr1aPtgs2Serping1Fcer1gAnxa1C3PpargAlox5apCcr5 |
| [GO:0001501 skeletal system development](http://amigo.geneontology.org/cgi-bin/amigo/term_details?term=GO:0001501&session_id=) | 9.50e-06 | Ltbp3Tgfb2Loxl2CtgfMmp2Sfrp2Fgfr2MgpLumSfrp1Col1a2Fgfr1Fbn1Npr3ChadCol3a1Creb3l2AcanCol1a1Prrx2FrzbRunx1Timp1Tgfb1Bmp1PthlhSulf2Tbx3Runx2Lgals3Sulf1Alx3Cd44Prrx1CompLOC681309Fgf18Thbs1Bmp6 |
| [GO:0042592 homeostatic process](http://amigo.geneontology.org/cgi-bin/amigo/term_details?term=GO:0042592&session_id=) | 1.02e-05 | Ltbp3Tgfb2Scn1aAbcg1Fhl1Aqp7Cxcl13ApoeInhbaQsox1C5ar1Slc22a5Dgat2MafbGpamTrdnCacna1gSpp1Cd55Gpihbp1AcadvlCcr1CtshHampThy1Hfe2AplnUcp2Zfp238Trpc6Htr2aMlxiplTbxas1Txn2Fgf7C3ar1Sfrp4Pmp22Dgat1Timp1Tgfb1Egln1CorinCsf1Dhrs7cAcox1Ptgs2Scara5PthlhScn3bNppaNrxn1Stc2Ephx2RelnPrkcbP2ry1Abca1G6pdGna15CtskFcer1gCsf1rAtp1a2Sypl2TfAnxa1Tnfrsf11bAxlFzd2Cd44CpSlc26a5Scn4bFn1Lpar1PpargHas2Ildr2PllpCcl2Piezo2AspaCcr5CtssNckap1lTgm2 |
| [GO:0046395 carboxylic acid catabolic process](http://amigo.geneontology.org/cgi-bin/amigo/term_details?term=GO:0046395&session_id=) | 1.20e-05 | Acsf3EhhadhBcat2AcadmPon3BckdhaHadhaAcadvlGot2MlycdCpt2HadhbAcot2Acox1Decr1Eci1ProdhBcat1Abcd3Lpin1AcadsHadhEch1Acot7Gpt |
| [GO:0016054 organic acid catabolic process](http://amigo.geneontology.org/cgi-bin/amigo/term_details?term=GO:0016054&session_id=) | 1.20e-05 | Acsf3EhhadhBcat2AcadmPon3BckdhaHadhaAcadvlGot2MlycdCpt2HadhbAcot2Acox1Decr1Eci1ProdhBcat1Abcd3Lpin1AcadsHadhEch1Acot7Gpt |
| [GO:0050778 positive regulation of immune response](http://amigo.geneontology.org/cgi-bin/amigo/term_details?term=GO:0050778&session_id=) | 1.33e-05 | Tgfb2Cd28CfhPolr3gPtafrC5ar1PtprjIfi204C6Cd55LbpCd46Thy1Clec7aUnc93b1C1qbC3ar1Fcgr1aTlr7RT1-DMaNfam1Plscr1PrkcbSerping1Nlrp10Fcer1gC1qaC3Tlr5C1qcC1sNckap1l |
| [GO:0030155 regulation of cell adhesion](http://amigo.geneontology.org/cgi-bin/amigo/term_details?term=GO:0030155&session_id=) | 1.47e-05 | Tgfb2Epha7VtnFstl3Mmp2Cxcl13PtprjSpp1Fbln2Col1a1Il18PrkcdRnd1Cyth4Cdh13Tgfb1Csf1Emilin1Serpine2Dpp4Smoc1Smad7Col8a1Dab2Cd44Has2Ddr1Mmp14Ccr5Nckap1lThbs1Tgm2 |
| [GO:0001503 ossification](http://amigo.geneontology.org/cgi-bin/amigo/term_details?term=GO:0001503&session_id=) | 1.55e-05 | Ltbp3Tgfb2CtgfFstl3Mmp2Sfrp2AspnFgfr2MgpSpp1Ifi204Sfrp1Ccr1Igsf10Fgfr1AcanCol1a1Il18GpnmbTgfb1OmdCsf1Ptgs2PthlhRunx2Smoc1CtskFn1Ecm1Ifitm1Tmem119LOC681309Mmp14Fgf18Bmp6 |
| [GO:0030334 regulation of cell migration](http://amigo.geneontology.org/cgi-bin/amigo/term_details?term=GO:0030334&session_id=) | 1.67e-05 | Tgfb2VtnCxcl13ApoeSfrp2PtprjLrp1Dpysl3Sfrp1LbpCcr1CtshP2ry6Thy1AcanNbl1Col1a1Tp53inp1Col18a1Fgf7C3ar1Cdh13Timp1Tgfb1Csf1Ptgs2FigfThbs4Srpx2Sulf1Csf1rDab2Lpar1Ecm1Cx3cr1Ccl2Ifitm1Mmp14Ccr5Abhd2Nckap1lThbs1 |
| [GO:0002526 acute inflammatory response](http://amigo.geneontology.org/cgi-bin/amigo/term_details?term=GO:0002526&session_id=) | 1.68e-05 | CfhC6Cd55LbpCd46Ccr1Nupr1Fcgr1aAlox5Ptgs2Plscr1Serping1Fcer1gTfAnxa1Fn1C3PpargAlox5apCcr5 |
| [GO:0002253 activation of immune response](http://amigo.geneontology.org/cgi-bin/amigo/term_details?term=GO:0002253&session_id=) | 2.74e-05 | CfhPtafrC5ar1PtprjIfi204C6Cd55LbpCd46Thy1Clec7aUnc93b1C1qbC3ar1Tlr7Nfam1Plscr1PrkcbSerping1Fcer1gC1qaC3Tlr5C1qcC1sNckap1l |
| [GO:0006635 fatty acid beta-oxidation](http://amigo.geneontology.org/cgi-bin/amigo/term_details?term=GO:0006635&session_id=) | 2.90e-05 | EhhadhAcadmHadhaAcadvlMlycdCpt2HadhbAcox1Decr1Eci1Abcd3AcadsHadhEch1 |
| [GO:0071495 cellular response to endogenous stimulus](http://amigo.geneontology.org/cgi-bin/amigo/term_details?term=GO:0071495&session_id=) | 4.06e-05 | Ltbp3Tgfb2CtgfMmp2Cxcl13InhbaGpamCacna1gAspnFgfr2Sfrp1Col1a2Ucp3Fgfr1CtshCol3a1Tgfb1i1Plod2Ucp2Slit3Col1a1Il18PrkcdFgf7Cuzd1Cdh13Tgfb1Ptgs2Adamtsl2Hsd11b1Sulf2Runx2RelnPrkcbSulf1AldobSmad7Ltbp2TfDab2Anxa1Fzd2Cd44Fn1Pdk4PpargHas2Cx3cr1Ccl2Lpin1Apobec1Fgf18CtssThbs1 |
| [GO:0044283 small molecule biosynthetic process](http://amigo.geneontology.org/cgi-bin/amigo/term_details?term=GO:0044283&session_id=) | 4.21e-05 | Impa2Sptlc2Tgfb2Abcg1Acsf3Ltc4sApoeBcat2AcadmPtafrPtgs1MecrFads1AcadvlDseGot2HpgdsPtgisAcacbTbxas1Fgf7Aldh1a1MlycdGamtAlox5Ptgs2Plscr1ProdhMthfd2Bcat1Fads2P2ry1G6pdMid1ip1Fdft1Anxa1Ch25hDkk3Pdk4Alox5apBmp6 |
| [GO:0040012 regulation of locomotion](http://amigo.geneontology.org/cgi-bin/amigo/term_details?term=GO:0040012&session_id=) | 6.00e-05 | Tgfb2VtnCxcl13ApoeSfrp2PtprjLrp1Dpysl3Sfrp1LbpCcr1CtshP2ry6Thy1AcanNbl1Col1a1Tp53inp1Col18a1Fgf7C3ar1Cdh13Timp1Tgfb1Csf1Ptgs2FigfThbs4Srpx2Sulf1Csf1rTfDab2Lpar1Ecm1Has2Cx3cr1Ccl2Ifitm1Mmp14Ccr5Abhd2Nckap1lThbs1 |
| [GO:0031589 cell-substrate adhesion](http://amigo.geneontology.org/cgi-bin/amigo/term_details?term=GO:0031589&session_id=) | 6.16e-05 | CtgfVtnPtprjSpp1Antxr1Fbln2Lamc1Itgbl1VwfThy1Col3a1Tnfrsf12aCol1a1Cdh13Csf1Emilin1Epdr1Smoc1Col8a1Dab2AxlFn1Ddr1Mmp14Actn1Thbs1 |
| [GO:0070848 response to growth factor stimulus](http://amigo.geneontology.org/cgi-bin/amigo/term_details?term=GO:0070848&session_id=) | 8.92e-05 | Ltbp3Tgfb2CtgfCxcl13ApoeMyofAspnFgfr2LumSfrp1Col1a2Fgfr1Col3a1AcanTgfb1i1Col1a1Fgf7Acot2Tgfb1FigfAdamtsl2Sulf2Runx2PrkcbP2ry1Sulf1Smad7Ltbp2Dab2Fzd2Cd44Fn1Has2Cx3cr1Ccl2Fgf18Thbs1 |
| [GO:0044711 single-organism biosynthetic process](http://amigo.geneontology.org/cgi-bin/amigo/term_details?term=GO:0044711&session_id=) | 9.17e-05 | Impa2Sptlc2Tgfb2Abcg1Acsf3Ltc4sApoeBcat2AcadmPtafrPtgs1MecrFads1AcadvlDseGot2HpgdsPtgisAcacbTbxas1Fgf7Aldh1a1MlycdGamtAlox5Ptgs2Plscr1ProdhMthfd2Bcat1Fads2P2ry1G6pdMid1ip1Fdft1Anxa1Ch25hDkk3Pdk4Alox5apBmp6 |
| [GO:0048646 anatomical structure formation involved in morphogenesis](http://amigo.geneontology.org/cgi-bin/amigo/term_details?term=GO:0048646&session_id=) | 9.83e-05 | Ltbp3Tgfb2Loxl2Sema5aVcanEpha7CtgfRaph1Mmp2ApoeInhbaSfrp2MafbPtprjAspnFgfr2MgpEtv5Spp1Ptcd2C6Cysltr1Serpinf1Sfrp1Tspan12Col1a2Fgfr1CtshTiam1Thy1Tnfrsf12aSlit3Nbl1EnahPtgisFlnaCol1a1Asb4Il18Prrx2Col18a1Fgf7Aldh1a1Sdc2C3ar1Sfrp4FrzbRunx1Pmp22Pdlim5Cdh13Tgfb1Egln1Anxa2Csf1Ptgs2FigfIrx4PthlhSerpine2Chi3l1Xylt1Thbs4Nrxn1Srpx2Tbx3Runx2RelnPrkcbGas7Sulf1Etv1Smad7Sat1Uchl1Tmem100Rab23Fzd2CnpCd44Prrx1Fn1C3CompEcm1Cx3cr1Ccl2Nr4a3Ifitm1Ddr1LOC681309Mmp14Ccr5Fgf18Ncam1Thbs1Tgm2Bmp6 |
| [GO:0050896 response to stimulus](http://amigo.geneontology.org/cgi-bin/amigo/term_details?term=GO:0050896&session_id=) | 1.01e-04 | Car3Ltbp3Tgfb2Grk5Timp2Loxl2Sema5aFcgr2aVcanEpha7Rpl3Scn1aAbcg1Adcy7CtgfAqp7BokVtnLtc4sRaph1Cd28Eno2Gnao1ItgamFstl3Ccnd1Atp1a3Mmp2Mgst1Cxcl13Fkbp4ApoeXpr1InhbaAcadmCfhPolr3gSfrp2Kcnk2PtafrFndc5Pon3C5ar1Slc22a5Dgat2Tsc22d4BckdhaPrg4HadhaGpamCybbMyofPtprjTrdnCacna1gAspnFgfr2MgpLumEtv5Ptgs1Pea15aLrp1Spp1Ifi204S100a10Emr1Erc1Arl11C6Msr1Cd55Bex1Cysltr1Fads1Dpysl3Serpinf1Asb10Gpihbp1Csf2rbLyve1Sfrp1Tspan12Col1a2LbpZyxCyp2e1Fndc1Cd46Itgbl1Asb12Ucp3Abcb4Ccr1CdaPpp1r1aCotl1Igsf10Fgfr1F13a1CtshTiam1HampVwfGot2Npr3P2ry6Thy1Col3a1Creb3l2AcanTgfb1i1Plod2Tnfrsf12aClec7aHfe2AplnUcp2Slit3Mapk8ip1Ccl6Nbl1Efemp2EnahPtgisAcacbFlnaCol1a1RT1-M1-2Pdk1S1pr3Nt5eAcsl1GrnAsb4Il18Htr2aPrkcdMlxiplPrrx2Unc93b1Slc38a3Olr1Rnd1Nupr1Tp53inp1Col18a1Rad23aTxn2Fgf7Crlf1Aldh1a1Casp12Lsp1Sdc2Mrc1C1qbPdk3C3ar1TfecSfrp4Ankrd23Asb14LipeFcgr1aCd14FrzbCuzd1Runx1Tlr7LitafCyth4Cdh13Timp1Acot2Tgfb1RT1-DMaEgln1Gnrh1Pfkfb1Anxa2Nfam1Map2k6Alox5Csf1Cntnap5aStk38lMapk12Cd4Ptgs2FigfCcbp2P2ry12Cyp4b1Lcp1Scara5PthlhSerpine2Chi3l1Adamtsl2Plscr1Elmo1PtgfrXylt1Hsd11b1NppaThbs4Nrxn1Kcnj3Agpat9Stc2Sulf2Ephx2Mthfd2Fbxo31Dpp4PostnRunx2RelnPrkcbGpr64Lgals3P2ry1Abca1Serping1Ncf1Sulf1AldobLyz2G6pdNlrp10Gna15Etv1Smad7Ltbp2Tnfsf18Fcer1gGpx3Csf1rAtp1a2TyrobpRGD1305645Abcd3Gpx8Uchl1Tmem100Rab23TfDab2FmodAnxa1Tnfrsf11bItga11Gpr183Phlda3AxlMmeC1qaFzd2CnpCd44AoahPrrx1CpDkk3Entpd1SelplgFn1Pde11aC3Cd68Pdk4Lpar1PpargEcm1Rab31Has2Cx3cr1Ildr2PllpAlox5apCcl2Casp7Hspb3Slc22a3Lpin1Trim16MdkNr4a3LoxS100a4AcadsCilpIfitm1Tlr5Ddr1Asah2Car4Exosc5Ddit4lC1qcMmp14GabreC1sHadhCcr5Aff3Apobec1Abhd2Htr1bNinj2Avpi1OsmrOlr995Fgf18CtssNcam1Nckap1lThbs1Sectm1bTgm2Bmp6 |
| [GO:0031960 response to corticosteroid stimulus](http://amigo.geneontology.org/cgi-bin/amigo/term_details?term=GO:0031960&session_id=) | 1.21e-04 | CtgfCcnd1AcadmBckdhaCacna1gMgpPtgs1Serpinf1Ucp3Abcb4Slit3Col1a1C1qbSfrp4Tgfb1Gnrh1Pfkfb1Ptgs2RelnAldobGpx3Anxa1Fn1C3Ccl2MdkAcadsHtr1bBmp6 |
| [GO:0030154 cell differentiation](http://amigo.geneontology.org/cgi-bin/amigo/term_details?term=GO:0030154&session_id=) | 1.22e-04 | Ltbp3Tgfb2Timp2Loxl2Sema5aVcanEpha7Csrp2CtgfTmem176aFhl1BokRaph1Cd28Gnao1ItgamFstl3Ccnd1Mmp2Mgst1Fkbp4ApoeInhbaAcadmHook1Sfrp2Fndc5MafbPtprjTrdnFgfr2MgpEtv5Spp1Ifi204Ptcd2S100a10Antxr1Msr1Bex1Dpysl3Serpinf1Sfrp1Soat1Lamc1Loxl3Ccr1Igsf10Fgfr1Tiam1Thy1Creb3l2AcanTgfb1i1Tnfrsf12aSlit3Nbl1Zfp238EnahFlnaCol1a1GrnAsb4Il18Rnd1Nupr1Col18a1Fndc3bSdc2Tgm1KySfrp4Cacna2d2FrzbRunx1Pmp22GpnmbPdlim5Timp1Tgfb1RT1-DMaNfam1Csf1Mapk12Cd4Ptgs2Tmem176bFigfP2ry12PthlhSerpine2Plscr1Xylt1Thbs4Dbn1Sulf2Pls3Piwil2Tbx3Runx2RelnGas7Lgals3P2ry1Sulf1G6pdEtv1Smoc1Csf1rUchl1Tmem100MregTfDab2Anxa1Tnfrsf11bGpr183AxlFzd2CnpCd44Fn1Lpar1PpargIldr2Ccl2Itm2cLpin1Trim16MdkNr4a3AspaIfitm1Tmem119C1qcAbca5Mmp14C1sCcr5Fgf18Ncam1Nckap1lBmp6 |
| [GO:0034097 response to cytokine stimulus](http://amigo.geneontology.org/cgi-bin/amigo/term_details?term=GO:0034097&session_id=) | 1.37e-04 | Tgfb2Timp2Rpl3Gnao1Mmp2ApoeCfhIfi204Dpysl3Sfrp1Ccr1Col3a1Slit3PtgisAcsl1Il18Cd14Timp1Ptgs2FigfCcbp2Chi3l1Plscr1Xylt1AldobTnfsf18Csf1rAnxa1AxlMmeSelplgFn1PpargEcm1Has2Cx3cr1Ccl2Ifitm1Ccr5Aff3OsmrThbs1 |
| [GO:0097305 response to alcohol](http://amigo.geneontology.org/cgi-bin/amigo/term_details?term=GO:0097305&session_id=) | 1.37e-04 | Tgfb2Adcy7CtgfEno2Ccnd1Mmp2ApoeInhbaCacna1gPtgs1Spp1Sfrp1Cyp2e1Got2Slit3GrnPrkcdAldh1a1Casp12Sfrp4Cd14Tgfb1Gnrh1Ptgs2Hsd11b1Stc2RelnPrkcbAbca1G6pdGpx3Anxa1Fzd2C3Ccl2Ccr5Apobec1Htr1bThbs1 |
| [GO:0042221 response to chemical stimulus](http://amigo.geneontology.org/cgi-bin/amigo/term_details?term=GO:0042221&session_id=) | 1.70e-04 | Ltbp3Tgfb2Grk5Timp2Sema5aEpha7Rpl3Abcg1Adcy7CtgfAqp7Ltc4sEno2Gnao1ItgamFstl3Ccnd1Atp1a3Mmp2Mgst1Cxcl13ApoeInhbaAcadmCfhSfrp2PtafrPon3C5ar1Slc22a5Dgat2BckdhaHadhaGpamCybbMyofPtprjTrdnCacna1gAspnFgfr2MgpLumEtv5Ptgs1Pea15aSpp1Ifi204S100a10Msr1Cd55Cysltr1Fads1Dpysl3Serpinf1Gpihbp1Csf2rbSfrp1Col1a2LbpCyp2e1Fndc1Ucp3Abcb4Ccr1CdaFgfr1CtshGot2Col3a1Creb3l2AcanTgfb1i1Plod2Clec7aUcp2Slit3Ccl6Nbl1EnahPtgisAcacbCol1a1Nt5eAcsl1GrnIl18Htr2aPrkcdMlxiplSlc38a3Olr1Nupr1Col18a1Txn2Fgf7Aldh1a1Casp12Lsp1Sdc2C1qbPdk3C3ar1Sfrp4LipeCd14Cuzd1Runx1Tlr7LitafCdh13Timp1Acot2Tgfb1Egln1Gnrh1Pfkfb1Anxa2Map2k6Alox5Ptgs2FigfCcbp2Cyp4b1Chi3l1Adamtsl2Plscr1PtgfrXylt1Hsd11b1NppaThbs4Stc2Sulf2Ephx2Runx2RelnPrkcbP2ry1Abca1Sulf1AldobG6pdEtv1Smad7Ltbp2Tnfsf18Fcer1gGpx3Csf1rAtp1a2Abcd3TfDab2Anxa1Tnfrsf11bAxlMmeFzd2CnpCd44CpSelplgFn1C3Cd68Pdk4Lpar1PpargEcm1Has2Cx3cr1Ildr2Alox5apCcl2Slc22a3Lpin1Trim16MdkNr4a3LoxAcadsIfitm1Tlr5Asah2Car4Mmp14GabreC1sHadhCcr5Aff3Apobec1Htr1bOsmrOlr995Fgf18CtssNcam1Nckap1lThbs1Bmp6 |
| [GO:0051271 negative regulation of cellular component movement](http://amigo.geneontology.org/cgi-bin/amigo/term_details?term=GO:0051271&session_id=) | 3.03e-04 | Cxcl13ApoeSfrp2PtprjLrp1Dpysl3Sfrp1Thy1AcanNbl1Tp53inp1Timp1Tgfb1Sulf1Cx3cr1Ccl2Ifitm1Ccr5Abhd2Actn1Thbs1 |
| [GO:0008610 lipid biosynthetic process](http://amigo.geneontology.org/cgi-bin/amigo/term_details?term=GO:0008610&session_id=) | 3.25e-04 | Sptlc2Agpat2Abcg1Acsf3Ltc4sApoeDgat2GpamPtgs1MecrFads1AcadvlHpgdsPtgisAcacbNt5eAcsl1Htr2aPrkcdTbxas1Fgf7Aldh1a1MlycdDgat1Timp1Alox5Ptgs2Plscr1Hsd11b1Agpat9Fads2G6pdMid1ip1Fdft1Anxa1Ch25hDkk3C3Pdk4Alox5apBmp6 |
| [GO:0071363 cellular response to growth factor stimulus](http://amigo.geneontology.org/cgi-bin/amigo/term_details?term=GO:0071363&session_id=) | 3.53e-04 | Ltbp3Tgfb2CtgfCxcl13ApoeMyofAspnFgfr2Sfrp1Col1a2Fgfr1Col3a1AcanTgfb1i1Col1a1Fgf7Tgfb1FigfAdamtsl2Sulf2Runx2PrkcbSulf1Smad7Ltbp2Dab2Fzd2Cd44Fn1Has2Cx3cr1Ccl2Fgf18Thbs1 |
| [GO:0019395 fatty acid oxidation](http://amigo.geneontology.org/cgi-bin/amigo/term_details?term=GO:0019395&session_id=) | 3.58e-04 | EhhadhAcadmHadhaAcadvlMlycdCpt2HadhbAcox1Decr1Eci1Abcd3PpargAcadsHadhEch1 |
| [GO:0051240 positive regulation of multicellular organismal process](http://amigo.geneontology.org/cgi-bin/amigo/term_details?term=GO:0051240&session_id=) | 3.70e-04 | Ltbp3Tgfb2Agpat2CtgfInhbaPolr3gSlc22a5GpamFgfr2Ptgs1Spp1Cysltr1Gpihbp1LbpFgfr1Clec7aAplnIl18Htr2aTbxas1C3ar1Cacna2d2Cd14Tlr7Tgfb1Alox5Csf1Ptgs2FigfSerpine2Scn3bNrxn1Sulf2Runx2RelnP2ry1Sulf1Fcer1gCsf1rTfC3Ccl2Trim16Tlr5Ccr5Ncam1Thbs1Bmp6 |
| [GO:0045785 positive regulation of cell adhesion](http://amigo.geneontology.org/cgi-bin/amigo/term_details?term=GO:0045785&session_id=) | 4.01e-04 | Tgfb2VtnFstl3Cxcl13PtprjSpp1Fbln2Cdh13Csf1Emilin1Smoc1Smad7Col8a1Dab2Cd44Has2Ccr5Nckap1lThbs1Tgm2 |
| [GO:0032844 regulation of homeostatic process](http://amigo.geneontology.org/cgi-bin/amigo/term_details?term=GO:0032844&session_id=) | 4.25e-04 | Ltbp3Tgfb2Fhl1InhbaSlc22a5MafbGpamTrdnCacna1gSpp1Ccr1Thy1AplnUcp2Htr2aMlxiplTgfb1CorinDhrs7cAcox1Ptgs2Scn3bFcer1gCsf1rTfAnxa1Tnfrsf11bCd44Scn4bCcr5Nckap1l |
| [GO:0044765 single-organism transport](http://amigo.geneontology.org/cgi-bin/amigo/term_details?term=GO:0044765&session_id=) | 4.38e-04 | Tgfb2Hcn4Scn1aSec16bAbcg1CtgfFhl1Aqp7Gnao1Ccnd1Atp1a3Fkbp4ApoeBcat2InhbaHook1Sfrp2Slc16a7Kcnk2Slc22a5GpamTrdnCacna1gFgfr2Ptgs1Pea15aLrp1Erc1Fxyd2Msr1Cysltr1Gpihbp1Sfrp1LbpSoat1Clcn1Ucp3Abcb4Ccr1Got2Npr3P2ry6Thy1Creb3l2Clec7aAplnUcp2FlnaPex19Osbpl6Trpc6Acsl1GrnIl18Htr2aPrkcdSlc38a3Fgf7Slc46a3Slc6a6Cpt2Sfrp4Atp6ap1lCacna2d2Fcgr1aCd14Runx1Tlr7LitafDgat1Tgfb1Sv2cS100a6Slc16a10Anxa2CorinDhrs7cPtgs2P2ry12Scara5Serpine2Chi3l1Scn3bPlscr1Elmo1Nrxn1Stc2RilpTbx3RelnPrkcbP2ry1Abca1Nlrp10Fcer1gCsf1rAtp1a2Abcd3Kcnt2Uchl1TfDab2Anxa1AxlCnpScrn1CpScn4bC3PpargRab31Has2Ildr2PllpAdhfe1Ccl2Slc22a3Slc4a1Piezo2Trim16Ddr1Car4Slc25a42Abca5C1qtnf5GabreHadhCcr5Apobec1Htr1bSlc43a2Fxyd3Ncam1Nckap1lThbs1Tgm2 |
| [GO:0042060 wound healing](http://amigo.geneontology.org/cgi-bin/amigo/term_details?term=GO:0042060&session_id=) | 5.01e-04 | Tgfb2ApoeMyofPtprjIgsf10F13a1VwfCol3a1Efemp2Col1a1PrkcdFgf7Sdc2Timp1Tgfb1Anxa2P2ry12Serpine2P2ry1Serping1Fcer1gFmodAxlCd44Entpd1Fn1C3LoxDdr1Ninj2Thbs1 |
| [GO:0030336 negative regulation of cell migration](http://amigo.geneontology.org/cgi-bin/amigo/term_details?term=GO:0030336&session_id=) | 5.95e-04 | Cxcl13ApoeSfrp2PtprjLrp1Dpysl3Sfrp1Thy1AcanNbl1Tp53inp1Timp1Tgfb1Sulf1Cx3cr1Ccl2Ifitm1Ccr5Abhd2Thbs1 |
| [GO:0034440 lipid oxidation](http://amigo.geneontology.org/cgi-bin/amigo/term_details?term=GO:0034440&session_id=) | 6.36e-04 | EhhadhAcadmHadhaAcadvlMlycdCpt2HadhbAcox1Decr1Eci1Abcd3PpargAcadsHadhEch1 |
| [GO:0006810 transport](http://amigo.geneontology.org/cgi-bin/amigo/term_details?term=GO:0006810&session_id=) | 6.37e-04 | Tgfb2Hcn4Scn1aSec16bAbcg1CtgfFhl1Aqp7VtnGnao1Ccnd1Atp1a3Fkbp4ApoeBcat2InhbaHook1Sfrp2Slc16a7Kcnk2Slc22a5GpamSlc25a15TrdnCacna1gFgfr2Ptgs1Pea15aLrp1Erc1Fxyd2Msr1Cysltr1Gpihbp1Sfrp1LbpSoat1Clcn1Fndc1Ucp3Abcb4Ccr1Rab3il1Got2Npr3P2ry6Thy1Creb3l2Clec7aAplnUcp2Mrc2Mapk8ip1EnahFlnaCol1a1Pex19Osbpl6Trpc6Acsl1GrnIl18Htr2aPrkcdUnc93b1Slc38a3Fgf7Slc25a20Slc46a3Mrc1Slc6a6Cpt2Sfrp4Atp6ap1lCacna2d2Slc25a34Fcgr1aCd14Runx1Tlr7LitafMyo5bCyth4Cdh13Dgat1Tgfb1Sv2cRT1-DMaS100a6Slc16a10Anxa2CorinDhrs7cPtgs2P2ry12Lcp1Scara5Serpine2Chi3l1Scn3bPlscr1Elmo1Nrxn1Stc2RilpTbx3RelnPrkcbP2ry1Abca1Nlrp10Fcer1gCsf1rAtp1a2Abcd3Kcnt2Uchl1Rab23TfDab2Anxa1AxlCnpScrn1CpTmed3Scn4bC3PpargRab31Has2Ildr2PllpAdhfe1Ccl2Slc22a3Slc4a1Piezo2Trim16Ddr1Car4Slc25a42Abca5C1qtnf5GabreHadhCcr5Apobec1Htr1bSlc43a2Fxyd3CratNcam1Nckap1lThbs1Tgm2 |
| [GO:1901342 regulation of vasculature development](http://amigo.geneontology.org/cgi-bin/amigo/term_details?term=GO:1901342&session_id=) | 6.59e-04 | Sfrp2C6Cysltr1Serpinf1Tspan12CtshPtgisAsb4C3ar1Runx1Egln1Ptgs2FigfChi3l1Thbs4Srpx2PrkcbSulf1C3Ecm1Cx3cr1Ccl2Thbs1 |
| [GO:0044242 cellular lipid catabolic process](http://amigo.geneontology.org/cgi-bin/amigo/term_details?term=GO:0044242&session_id=) | 6.77e-04 | EhhadhAcadmHadhaAcadvlAkr1b10PrkcdMlycdCpt2LipeHadhbAcot2Acox1Decr1Eci1Abcd3Lpin1AcadsHadhEch1Acot7 |
| [GO:0048545 response to steroid hormone stimulus](http://amigo.geneontology.org/cgi-bin/amigo/term_details?term=GO:0048545&session_id=) | 7.11e-04 | Tgfb2CtgfEno2Ccnd1Mmp2AcadmBckdhaCacna1gMgpPtgs1Spp1Serpinf1Sfrp1Ucp3Abcb4Slit3Col1a1GrnAldh1a1C1qbSfrp4Tgfb1Gnrh1Pfkfb1Ptgs2Hsd11b1RelnAldobGpx3Anxa1Tnfrsf11bFn1C3PpargCcl2MdkLoxAcadsCar4Mmp14Htr1bThbs1Bmp6 |
| [GO:0045595 regulation of cell differentiation](http://amigo.geneontology.org/cgi-bin/amigo/term_details?term=GO:0045595&session_id=) | 7.11e-04 | Ltbp3Tgfb2Timp2Loxl2CtgfTmem176aFstl3Ccnd1Fkbp4ApoeInhbaSfrp2Fndc5MafbFgfr2Etv5Spp1Ifi204S100a10Msr1Bex1Dpysl3Serpinf1Sfrp1Ccr1Fgfr1Tiam1Thy1Tgfb1i1Tnfrsf12aNbl1Col1a1GrnAsb4Fndc3bSfrp4FrzbRunx1Pmp22Pdlim5Tgfb1RT1-DMaNfam1Csf1Ptgs2Tmem176bP2ry12PthlhSerpine2Xylt1Piwil2Tbx3Runx2RelnSmoc1Csf1rDab2Tnfrsf11bAxlLpar1PpargItm2cLpin1Trim16AspaIfitm1C1qcAbca5Ccr5Fgf18Nckap1lBmp6 |
| [GO:0045765 regulation of angiogenesis](http://amigo.geneontology.org/cgi-bin/amigo/term_details?term=GO:0045765&session_id=) | 8.32e-04 | Sfrp2C6Cysltr1Serpinf1Tspan12CtshPtgisC3ar1Runx1Egln1Ptgs2FigfChi3l1Thbs4Srpx2PrkcbSulf1C3Ecm1Cx3cr1Ccl2Thbs1 |
| [GO:0009887 organ morphogenesis](http://amigo.geneontology.org/cgi-bin/amigo/term_details?term=GO:0009887&session_id=) | 8.45e-04 | Ltbp3Tgfb2CtgfCcnd1Mmp2InhbaSfrp2C5ar1MafbAspnFgfr2Etv5Ptcd2Sfrp1Tspan12Col1a2Fgfr1CtshThy1ChadAcanUcp2Slit3Col1a1Prrx2Fgf7Aldh1a1Tgm1FrzbRunx1Tgfb1Egln1Pfkfb1GamtCsf1Lcp1Irx4PthlhTbx3Runx2PrkcbSulf1Smad7Col8a1Alx3FmodTnfrsf11bAxlFzd2Cd44Prrx1CompPpargCcl2Lpin1Nr4a3Ddr1LOC681309Fgf18Ncam1Thbs1Bmp6 |
| [GO:2000146 negative regulation of cell motility](http://amigo.geneontology.org/cgi-bin/amigo/term_details?term=GO:2000146&session_id=) | 8.73e-04 | Cxcl13ApoeSfrp2PtprjLrp1Dpysl3Sfrp1Thy1AcanNbl1Tp53inp1Timp1Tgfb1Sulf1Cx3cr1Ccl2Ifitm1Ccr5Abhd2Thbs1 |
| [GO:0032103 positive regulation of response to external stimulus](http://amigo.geneontology.org/cgi-bin/amigo/term_details?term=GO:0032103&session_id=) | 9.20e-04 | Cd28Cxcl13PtprjLbpCcr1C3ar1Fcgr1aCdh13Tgfb1Ptgs2FigfThbs4Fcer1gC3Lpar1Alox5apCcl2Ccr5Nckap1lThbs1Tgm2 |
| [GO:0030258 lipid modification](http://amigo.geneontology.org/cgi-bin/amigo/term_details?term=GO:0030258&session_id=) | 9.90e-04 | Impa2Abcg1EhhadhApoeAcadmHadhaSoat1AcadvlMlycdCpt2HadhbAcox1Decr1Eci1Ephx2Abcd3PpargAcadsHadhEch1 |
| [GO:0007568 aging](http://amigo.geneontology.org/cgi-bin/amigo/term_details?term=GO:0007568&session_id=) | 1.04e-03 | Tgfb2Timp2CtgfGnao1Mmp2ApoePtgs1Lrp1Fads1Serpinf1Ucp3AcanUcp2Htr2aPrkcdCasp12PrelpC1qbTimp1Tgfb1Tbx3P2ry1RGD1305645MmeCnpCpCcl2Casp7Ccr5Ncam1 |
| [GO:0006636 unsaturated fatty acid biosynthetic process](http://amigo.geneontology.org/cgi-bin/amigo/term_details?term=GO:0006636&session_id=) | 1.04e-03 | Ltc4sPtgs1Fads1HpgdsPtgisTbxas1Alox5Ptgs2Fads2Anxa1Alox5ap |
| [GO:0048771 tissue remodeling](http://amigo.geneontology.org/cgi-bin/amigo/term_details?term=GO:0048771&session_id=) | 1.12e-03 | Ltbp3Tgfb2Mmp2Spp1Sfrp1Il18BgnSfrp4Tgfb1CtskCsf1rElnTfAnxa1Tnfrsf11bAxlMmp14Htr1bCtssTgm2 |
| [GO:2000147 positive regulation of cell motility](http://amigo.geneontology.org/cgi-bin/amigo/term_details?term=GO:2000147&session_id=) | 1.19e-03 | Tgfb2VtnCxcl13PtprjLbpCcr1CtshP2ry6Col1a1Col18a1Fgf7C3ar1Cdh13Tgfb1Csf1Ptgs2FigfThbs4Srpx2Csf1rTfDab2Lpar1Has2Ccl2Mmp14Nckap1lThbs1 |
| [GO:0051234 establishment of localization](http://amigo.geneontology.org/cgi-bin/amigo/term_details?term=GO:0051234&session_id=) | 1.22e-03 | Tgfb2Hcn4Scn1aSec16bAbcg1CtgfFhl1Aqp7VtnGnao1Ccnd1Atp1a3Fkbp4ApoeBcat2InhbaHook1Sfrp2Slc16a7Kcnk2Slc22a5GpamSlc25a15TrdnCacna1gFgfr2Ptgs1Pea15aLrp1Erc1Fxyd2Msr1Cysltr1Gpihbp1Sfrp1LbpSoat1Clcn1Fndc1Ucp3Abcb4Ccr1Rab3il1Got2Npr3P2ry6Thy1Creb3l2Clec7aAplnUcp2Mrc2Mapk8ip1EnahFlnaCol1a1Pex19Osbpl6Trpc6Acsl1GrnIl18Htr2aPrkcdUnc93b1Slc38a3Fgf7Slc25a20Slc46a3Mrc1Slc6a6Cpt2Sfrp4Atp6ap1lCacna2d2Slc25a34Fcgr1aCd14Runx1Tlr7LitafMyo5bCyth4Cdh13Dgat1Tgfb1Sv2cRT1-DMaS100a6Slc16a10Anxa2CorinDhrs7cPtgs2P2ry12Lcp1Scara5Serpine2Chi3l1Scn3bPlscr1Elmo1Nrxn1Stc2RilpDpp4Tbx3RelnPrkcbP2ry1Abca1Nlrp10Fcer1gCsf1rAtp1a2Abcd3Kcnt2Uchl1Rab23TfDab2Anxa1AxlCnpScrn1CpTmed3Scn4bC3PpargRab31Has2Ildr2PllpAdhfe1Ccl2Slc22a3Slc4a1Piezo2Trim16Ddr1Car4Slc25a42Abca5C1qtnf5GabreHadhCcr5Apobec1Htr1bSlc43a2Fxyd3CratNcam1Nckap1lThbs1Tgm2 |
| [GO:0050727 regulation of inflammatory response](http://amigo.geneontology.org/cgi-bin/amigo/term_details?term=GO:0050727&session_id=) | 1.25e-03 | Cd28ApoeCfhC6Cd55Serpinf1LbpZyxCd46Ccr1PtgisNt5eFcgr1aPtgs2Serping1Fcer1gAnxa1AoahC3PpargAlox5apCcr5Tgm2 |
| [GO:0046394 carboxylic acid biosynthetic process](http://amigo.geneontology.org/cgi-bin/amigo/term_details?term=GO:0046394&session_id=) | 1.32e-03 | Acsf3Ltc4sBcat2AcadmPtgs1MecrFads1AcadvlDseGot2HpgdsPtgisAcacbTbxas1Aldh1a1MlycdGamtAlox5Ptgs2Plscr1ProdhMthfd2Bcat1Fads2Mid1ip1Anxa1Ch25hPdk4Alox5ap |
| [GO:0016053 organic acid biosynthetic process](http://amigo.geneontology.org/cgi-bin/amigo/term_details?term=GO:0016053&session_id=) | 1.32e-03 | Acsf3Ltc4sBcat2AcadmPtgs1MecrFads1AcadvlDseGot2HpgdsPtgisAcacbTbxas1Aldh1a1MlycdGamtAlox5Ptgs2Plscr1ProdhMthfd2Bcat1Fads2Mid1ip1Anxa1Ch25hPdk4Alox5ap |
| [GO:0033559 unsaturated fatty acid metabolic process](http://amigo.geneontology.org/cgi-bin/amigo/term_details?term=GO:0033559&session_id=) | 1.40e-03 | Ltc4sPtgs1Fads1HpgdsPtgisTbxas1Alox5Acox1Ptgs2Ephx2Fads2Ncf1Anxa1Alox5ap |
| [GO:0051704 multi-organism process](http://amigo.geneontology.org/cgi-bin/amigo/term_details?term=GO:0051704&session_id=) | 1.71e-03 | Tgfb2Adcy7Fgl2Ltc4sMmp2Mgst1Cxcl13Fkbp4Xpr1CfhPtafrC5ar1Slc22a5GpamCd55Csf2rbLbpCcr1Cotl1HampClec7aUcp2GrnIl18PrkcdRad23aSfrp4LipeFcgr1aCd14Cuzd1Tlr7LitafTimp1Tgfb1Gnrh1CorinCd4Ptgs2Fbln1Serpine2Plscr1PtgfrNppaNrxn1Stc2RelnP2ry1Abca1Ncf1Lyz2Nlrp10Fcer1gGpx3AxlCnpFn1Cx3cr1Ccl2Ifitm1Tlr5Ddr1Exosc5Ccr5Apobec1 |
| [GO:0006935 chemotaxis](http://amigo.geneontology.org/cgi-bin/amigo/term_details?term=GO:0006935&session_id=) | 1.98e-03 | Tgfb2Sema5aEpha7ItgamCxcl13PtafrC5ar1PtprjSpp1Cysltr1LbpCcr1Slit3Ccl6Nbl1EnahFgf7Lsp1C3ar1Cdh13Tgfb1FigfThbs4RelnEtv1Fcer1gC3Lpar1Cx3cr1Ccl2Nr4a3Nckap1lThbs1 |
| [GO:0006958 complement activation, classical pathway](http://amigo.geneontology.org/cgi-bin/amigo/term_details?term=GO:0006958&session_id=) | 2.00e-03 | C6Cd55C1qbSerping1C1qaC3C1qcC1s |
| [GO:0048878 chemical homeostasis](http://amigo.geneontology.org/cgi-bin/amigo/term_details?term=GO:0048878&session_id=) | 2.12e-03 | Tgfb2Scn1aAbcg1Fhl1Aqp7Cxcl13ApoeC5ar1Dgat2GpamTrdnCacna1gCd55Gpihbp1Ccr1CtshHampThy1Hfe2Ucp2Trpc6Htr2aMlxiplTbxas1Fgf7C3ar1Sfrp4Pmp22Dgat1Tgfb1Egln1CorinDhrs7cAcox1Scara5PthlhScn3bNppaNrxn1Stc2Ephx2RelnPrkcbP2ry1Abca1Gna15Atp1a2Sypl2TfFzd2CpSlc26a5Scn4bFn1Lpar1PpargHas2PllpCcl2Piezo2AspaCcr5Tgm2 |
| [GO:0042330 taxis](http://amigo.geneontology.org/cgi-bin/amigo/term_details?term=GO:0042330&session_id=) | 2.12e-03 | Tgfb2Sema5aEpha7ItgamCxcl13PtafrC5ar1PtprjSpp1Cysltr1LbpCcr1Slit3Ccl6Nbl1EnahFgf7Lsp1C3ar1Cdh13Tgfb1FigfThbs4RelnEtv1Fcer1gC3Lpar1Cx3cr1Ccl2Nr4a3Nckap1lThbs1 |
| [GO:0071396 cellular response to lipid](http://amigo.geneontology.org/cgi-bin/amigo/term_details?term=GO:0071396&session_id=) | 2.13e-03 | Ltc4sMmp2ApoeInhbaCfhPtafrDgat2Cacna1gSfrp1LbpCol1a1Il18Pdk3Cd14LitafTgfb1Plscr1Hsd11b1RelnAbca1Anxa1AxlFzd2Fn1Pdk4Cx3cr1Ccl2Tlr5Ccr5 |
| [GO:0051272 positive regulation of cellular component movement](http://amigo.geneontology.org/cgi-bin/amigo/term_details?term=GO:0051272&session_id=) | 2.14e-03 | Tgfb2VtnCxcl13PtprjLbpCcr1CtshP2ry6Col1a1Col18a1Fgf7C3ar1Cdh13Tgfb1Csf1Ptgs2FigfThbs4Srpx2Csf1rTfDab2Lpar1Has2Ccl2Mmp14Nckap1lThbs1 |
| [GO:0045766 positive regulation of angiogenesis](http://amigo.geneontology.org/cgi-bin/amigo/term_details?term=GO:0045766&session_id=) | 2.15e-03 | Sfrp2C6Cysltr1CtshPtgisC3ar1Runx1Ptgs2FigfChi3l1Srpx2PrkcbC3Ecm1Cx3cr1Thbs1 |
| [GO:0016043 cellular component organization](http://amigo.geneontology.org/cgi-bin/amigo/term_details?term=GO:0016043&session_id=) | 2.28e-03 | Tgfb2Loxl2Sema5aCnn1VcanEpha7Fhl3Sec16bAbcg1CtgfFhl1VtnRaph1Cd28Gnao1Mmp2Mgst1Cxcl13Fkbp4ApoeInhbaAcadmHook1Sfrp2NovSlc22a5MyofPtprjFapFgfr2MgpEtv5Lrp1Spp1Ptcd2S100a10Fxyd2Antxr1Dpysl3Sfrp1Col1a2Soat1Lamc1Loxl3Tfb2mActr3bCdaFgfr1Tiam1VwfThy1Col3a1Zfp462AcanTgfb1i1Tnfrsf12aClec7aSlit3Nbl1EnahAcacbFlnaCol1a1Pex19Myo3bGrnDhrs4PrkcdArpc1bRnd1Nupr1CapgCol18a1Fgf7Panx3Aldh1a1Sdc2KySfrp4Wisp2Cacna2d2Fcgr1aCd14FrzbPmp22Tppp3Pdlim5Cdh13Dgat1Tgfb1RT1-DMaSnx10Tubb6Anxa2Stk38lAcox1Fbln1P2ry12Lcp1Scara5Decr1Emilin1Serpine2Scn3bPlscr1Elmo1Olfml2bXylt1NppaThbs4Nrxn1Pcbd1Fitm1Dbn1Sulf2Dpp4PostnRelnPrkcbGas7Lgals3P2ry1Abca1Sulf1AldobMid1ip1Etv1Smoc1Prdm5Smad7Vat1Fcer1gGpx3Csf1rAbcd3ElnUchl1Igfbp6TfMfap5Dab2Cpeb1Anxa1Tnfrsf11bAxlPfkpFzd2CnpCd44Atpaf1Qrsl1Slc26a5Fn1C3Lpar1CompPpargLrrc16aLOC100361596Alox5apCcl2Itm2cCasp7Lpin1Trim16Nr4a3LoxAcadsDdr1Abca5Acot13C1qtnf5Mmp14Ccr5Apobec1Actn1Ncam1Nckap1lThbs1Tgm2 |
| [GO:0040017 positive regulation of locomotion](http://amigo.geneontology.org/cgi-bin/amigo/term_details?term=GO:0040017&session_id=) | 2.51e-03 | Tgfb2VtnCxcl13PtprjLbpCcr1CtshP2ry6Col1a1Col18a1Fgf7C3ar1Cdh13Tgfb1Csf1Ptgs2FigfThbs4Srpx2Csf1rTfDab2Lpar1Has2Ccl2Mmp14Nckap1lThbs1 |
| [GO:1901701 cellular response to oxygen-containing compound](http://amigo.geneontology.org/cgi-bin/amigo/term_details?term=GO:1901701&session_id=) | 2.53e-03 | Adcy7Ltc4sMmp2Mgst1ApoeInhbaCfhPtafrDgat2Cacna1gSfrp1Col1a2LbpCol3a1Ucp2Col1a1Il18MlxiplPdk3Cd14LitafTgfb1Ptgs2Plscr1Hsd11b1RelnPrkcbAbca1Gpx3TfAnxa1AxlFzd2Fn1Pdk4Cx3cr1Ccl2Tlr5Ccr5 |
| [GO:0001816 cytokine production](http://amigo.geneontology.org/cgi-bin/amigo/term_details?term=GO:0001816&session_id=) | 2.68e-03 | Tgfb2Agpat2Cd28Polr3gPtafrGpamLbpClec7aS1pr3Il18PrkcdC3ar1Cd14Tlr7LitafTgfb1Cd4Ptgs2FigfChi3l1Sulf2Abca1Sulf1G6pdNlrp10Fcer1gCsf1rAxlC3PpargCcl2Trim16Tlr5Ccr5Nckap1lThbs1 |
| [GO:0008283 cell proliferation](http://amigo.geneontology.org/cgi-bin/amigo/term_details?term=GO:0008283&session_id=) | 3.14e-03 | Tgfb2Timp2Loxl2CtgfBokCd28ItgamCcnd1ApoeInhbaSfrp2C5ar1GpamPtprjFgfr2Etv5Ptgs1Lrp1Ifi204Fxyd2Bex1Serpinf1Sfrp1Fgfr1CtshNpr3Thy1AplnSlit3Clec11aGrnIl18Htr2aPrkcdPrrx2Nupr1Tp53inp1Col18a1Fgf7Crlf1Sfrp4Wisp2FrzbRunx1Pmp22Cdh13Tgfb1Csf1Ptgs2FigfPthlhSerpine2Thbs4Dbn1Dpp4Tbx3Runx2Ncf1Pdcd1lg2Sulf1Tnfsf18Col8a1Csf1rSat1Uchl1Anxa1Gpr183AxlPrrx1PpargEcm1Has2Ccl2Ifitm1Ddr1Mmp14Ccr5Apobec1Sorl1OsmrFgf18Ncam1Nckap1lThbs1Tgm2Bmp6 |
| [GO:0045597 positive regulation of cell differentiation](http://amigo.geneontology.org/cgi-bin/amigo/term_details?term=GO:0045597&session_id=) | 3.24e-03 | Tgfb2Timp2Loxl2CtgfApoeInhbaSfrp2Fndc5Etv5Ifi204Msr1Bex1Serpinf1Sfrp1Ccr1Fgfr1Tiam1Tgfb1i1Tnfrsf12aNbl1Col1a1Asb4Fndc3bSfrp4FrzbRunx1Tgfb1RT1-DMaCsf1Ptgs2Serpine2Runx2Csf1rDab2AxlPpargTrim16AspaIfitm1Ccr5Fgf18Nckap1lBmp6 |
| [GO:0006956 complement activation](http://amigo.geneontology.org/cgi-bin/amigo/term_details?term=GO:0006956&session_id=) | 3.44e-03 | CfhC6Cd55Cd46C1qbSerping1C1qaC3C1qcC1s |
| [GO:0040007 growth](http://amigo.geneontology.org/cgi-bin/amigo/term_details?term=GO:0040007&session_id=) | 3.62e-03 | Tgfb2CtgfFhl1Cd53Raph1Mmp2ApoeInhbaSfrp2NovGpamPtprjFgfr2Spp1S100a10Fxyd2Sfrp1LbpCdaIgsf10Fgfr1VwfTnfrsf12aSlit3Nupr1Fgf7Wisp2Cacna2d2FrzbCdh13Tgfb1GamtCsf1Ptgs2PthlhSerpine2NppaStc2PrkcbG6pdEmp1Igfbp6MregDab2AxlCd44C3CompPpargDdr1LOC681309Mmp14Ccr5Ninj2Ncam1Thbs1Bmp6 |
| [GO:0048869 cellular developmental process](http://amigo.geneontology.org/cgi-bin/amigo/term_details?term=GO:0048869&session_id=) | 3.68e-03 | Ltbp3Tgfb2Timp2Loxl2Sema5aVcanEpha7Csrp2CtgfTmem176aFhl1BokRaph1Cd28Gnao1ItgamFstl3Ccnd1Mmp2Mgst1Fkbp4ApoeInhbaAcadmHook1Sfrp2Fndc5MafbPtprjTrdnFgfr2MgpEtv5Spp1Ifi204Ptcd2S100a10Antxr1Msr1Bex1Dpysl3Serpinf1Sfrp1Soat1Lamc1Loxl3Ccr1Igsf10Fgfr1Tiam1Thy1Creb3l2AcanTgfb1i1Tnfrsf12aSlit3Nbl1Zfp238EnahFlnaCol1a1GrnAsb4Il18Rnd1Nupr1Col18a1Fndc3bSdc2Tgm1KySfrp4Cacna2d2FrzbRunx1Pmp22GpnmbPdlim5Timp1Tgfb1RT1-DMaNfam1Csf1Mapk12Cd4Ptgs2Tmem176bFigfP2ry12PthlhSerpine2Plscr1Xylt1Thbs4Dbn1Sulf2Pls3Piwil2Tbx3Runx2RelnGas7Lgals3P2ry1Sulf1G6pdEtv1Smoc1Csf1rUchl1Tmem100MregTfDab2Anxa1Tnfrsf11bGpr183AxlFzd2CnpCd44Slc26a5Fn1Lpar1PpargIldr2Ccl2Itm2cLpin1Trim16MdkNr4a3AspaIfitm1Tmem119C1qcAbca5Mmp14C1sCcr5Fgf18Ncam1Nckap1lBmp6 |
| [GO:0072330 monocarboxylic acid biosynthetic process](http://amigo.geneontology.org/cgi-bin/amigo/term_details?term=GO:0072330&session_id=) | 4.08e-03 | Acsf3Ltc4sAcadmPtgs1MecrFads1AcadvlDseHpgdsPtgisAcacbTbxas1Aldh1a1MlycdAlox5Ptgs2Fads2Mid1ip1Anxa1Ch25hPdk4Alox5ap |
| [GO:0009628 response to abiotic stimulus](http://amigo.geneontology.org/cgi-bin/amigo/term_details?term=GO:0009628&session_id=) | 4.10e-03 | Tgfb2Loxl2CtgfCcnd1Atp1a3Mmp2AcadmSfrp2Tsc22d4MyofMgpSfrp1Fndc1Ucp3Col3a1AcanTgfb1i1Plod2Ucp2PtgisCol1a1Il18PrkcdCol18a1Txn2Casp12Sdc2Pdk3TfecAnkrd23Cd14Tlr7Acot2Tgfb1Egln1Alox5Ptgs2FigfScara5Serpine2Chi3l1Plscr1Xylt1Kcnj3Stc2Mthfd2Dpp4RelnP2ry1Etv1Atp1a2TfAnxa1MmeLpar1PpargCcl2Casp7Tlr5Mmp14Ccr5Apobec1Htr1bThbs1 |
| [GO:0010876 lipid localization](http://amigo.geneontology.org/cgi-bin/amigo/term_details?term=GO:0010876&session_id=) | 4.27e-03 | Abcg1ApoeInhbaDgat2Lrp1Msr1Gpihbp1LbpSoat1Got2Osbpl6Acsl1PrkcdCpt2Runx1Dgat1Plscr1Fitm1Abca1Abcd3Anxa1C3PpargAbca5Thbs1 |
| [GO:0001558 regulation of cell growth](http://amigo.geneontology.org/cgi-bin/amigo/term_details?term=GO:0001558&session_id=) | 4.37e-03 | Tgfb2CtgfFhl1ApoeInhbaSfrp2NovPtprjSpp1S100a10Fxyd2Sfrp1CdaTnfrsf12aSlit3Wisp2FrzbCdh13Tgfb1Serpine2NppaIgfbp6Dab2Cd44PpargDdr1Mmp14Ccr5 |
| [GO:0006633 fatty acid biosynthetic process](http://amigo.geneontology.org/cgi-bin/amigo/term_details?term=GO:0006633&session_id=) | 4.53e-03 | Acsf3Ltc4sPtgs1MecrFads1AcadvlHpgdsPtgisAcacbTbxas1MlycdAlox5Ptgs2Fads2Mid1ip1Anxa1Ch25hPdk4Alox5ap |
| [GO:0048522 positive regulation of cellular process](http://amigo.geneontology.org/cgi-bin/amigo/term_details?term=GO:0048522&session_id=) | 4.64e-03 | Tgfb2Timp2Loxl2Epha7Adcy7CtgfBokVtnCd28Fstl3Ccnd1Mmp2Cxcl13ApoeInhbaSfrp2Fndc5MafbGpamPtprjCacna1gFgfr2LumEtv5Spp1Ifi204C6Msr1Bex1Elk1Dpysl3Serpinf1Sfrp1LbpSoat1Fbln2Fndc1Tfb2mCcr1Fgfr1CtshTiam1P2ry6Thy1Creb3l2Zfp462Tgfb1i1Tnfrsf12aClec7aHfe2AplnUcp2Nbl1PtgisFlnaClec11aCol1a1Myo3bAcsl1GrnAsb4Il18Htr2aPrkcdPrrx2Nupr1Tp53inp1Col18a1Rad23aFgf7Crlf1Aldh1a1Casp12Fndc3bMlycdC3ar1Sfrp4Fcgr1aCd14FrzbRunx1Tlr7LitafCdh13Tgfb1RT1-DMaAnxa2Nfam1Map2k6Csf1Cd4Ptgs2FigfPthlhEmilin1Serpine2Chi3l1Plscr1Thbs4Nrxn1Pcbd1Sulf2Srpx2Dpp4Piwil2Tbx3Runx2RelnPrkcbP2ry1Abca1Pdcd1lg2Sulf1Nlrp10Mid1ip1Etv1Smoc1Smad7Fcer1gCol8a1Csf1rTfDab2Anxa1Gpr183Phlda3AxlFzd2Cd44Prrx1C3Lpar1PpargEcm1Has2Ccl2Itm2cCasp7Lpin1Trim16MdkNr4a3S100a4AspaIfitm1Tlr5Mmp14Ccr5Apobec1Avpi1OsmrFgf18Ncam1Nckap1lThbs1Tgm2Bmp6 |
| [GO:0007584 response to nutrient](http://amigo.geneontology.org/cgi-bin/amigo/term_details?term=GO:0007584&session_id=) | 4.78e-03 | Tgfb2Ltc4sCcnd1AcadmSfrp2BckdhaCybbMgpSpp1Fads1Sfrp1Ucp3Col1a1Acsl1Txn2Tgfb1Alox5Ptgs2Stc2PrkcbAbca1Tnfrsf11bFzd2Cd44CpPpargCcl2 |
| [GO:0040008 regulation of growth](http://amigo.geneontology.org/cgi-bin/amigo/term_details?term=GO:0040008&session_id=) | 4.93e-03 | Tgfb2CtgfFhl1Cd53ApoeInhbaSfrp2NovGpamPtprjFgfr2Spp1S100a10Fxyd2Sfrp1LbpCdaFgfr1Tnfrsf12aSlit3Wisp2Cacna2d2FrzbCdh13Tgfb1GamtCsf1Serpine2NppaStc2PrkcbG6pdIgfbp6Dab2Cd44C3PpargDdr1Mmp14Ccr5Ncam1 |
| [GO:0042127 regulation of cell proliferation](http://amigo.geneontology.org/cgi-bin/amigo/term_details?term=GO:0042127&session_id=) | 5.00e-03 | Tgfb2Timp2CtgfCd28Ccnd1ApoeInhbaSfrp2GpamPtprjFgfr2Etv5Ptgs1Ifi204Fxyd2Bex1Serpinf1Sfrp1Fgfr1CtshNpr3Thy1AplnSlit3Clec11aGrnIl18Htr2aPrrx2Nupr1Tp53inp1Col18a1Fgf7Crlf1Sfrp4Wisp2FrzbRunx1Pmp22Cdh13Tgfb1Csf1Ptgs2FigfPthlhSerpine2Thbs4Dpp4Tbx3Runx2Pdcd1lg2Sulf1Tnfsf18Csf1rSat1Anxa1Gpr183Prrx1PpargEcm1Has2Ccl2Ifitm1Ddr1Ccr5Apobec1OsmrFgf18Ncam1Nckap1lThbs1Tgm2Bmp6 |
| [GO:0051128 regulation of cellular component organization](http://amigo.geneontology.org/cgi-bin/amigo/term_details?term=GO:0051128&session_id=) | 5.03e-03 | Tgfb2CtgfFhl1VtnCd28Cxcl13Fkbp4ApoeInhbaSfrp2NovPtprjFapFgfr2Etv5Lrp1Spp1S100a10Fxyd2Dpysl3Sfrp1Actr3bCdaFgfr1Tiam1Thy1Tgfb1i1Tnfrsf12aClec7aSlit3Col1a1Myo3bGrnPrkcdArpc1bCapgSfrp4Wisp2Fcgr1aCd14FrzbPmp22Pdlim5Cdh13Tgfb1Anxa2Stk38lSerpine2Xylt1NppaNrxn1Dpp4RelnGas7Mid1ip1Vat1Fcer1gCsf1rElnIgfbp6Dab2Anxa1Cd44Slc26a5Fn1C3Lpar1PpargLOC100361596Ccl2Itm2cLpin1Ddr1Mmp14Ccr5Apobec1Ncam1Nckap1l |
| [GO:0031347 regulation of defense response](http://amigo.geneontology.org/cgi-bin/amigo/term_details?term=GO:0031347&session_id=) | 5.08e-03 | Cd28ApoeCfhPolr3gPtafrC6Cd55Serpinf1LbpZyxCd46Ccr1Clec7aPtgisNt5eUnc93b1Fcgr1aTlr7Ptgs2Plscr1Serping1Fcer1gAnxa1AoahC3PpargAlox5apTlr5Ccr5Tgm2 |
| [GO:0007167 enzyme linked receptor protein signaling pathway](http://amigo.geneontology.org/cgi-bin/amigo/term_details?term=GO:0007167&session_id=) | 6.20e-03 | Ltbp3Tgfb2Epha7CtgfFstl3InhbaSfrp2MyofPtprjAspnFgfr2Lrp1Bex1Sfrp1Col1a2Fgfr1Tiam1Col3a1Tgfb1i1Hfe2Nbl1PrkcdFgf7Cdh13Tgfb1FigfAdamtsl2NppaSulf2Runx2PrkcbSulf1Smad7Ltbp2Csf1rTfDab2AxlPdk4Ccl2CilpDdr1Fgf18Thbs1Bmp6 |
| [GO:0048519 negative regulation of biological process](http://amigo.geneontology.org/cgi-bin/amigo/term_details?term=GO:0048519&session_id=) | 6.51e-03 | Ltbp3Tgfb2Grk5Timp2Loxl2Abcg1CtgfTmem176aFhl1Gnao1Fstl3Ccnd1Mmp2Cxcl13Fkbp4ApoeInhbaSfrp2C5ar1MafbGpamPtprjTrdnFapAspnFgfr2HhatlPtgs1Pea15aLrp1Spp1Ifi204Bace2Cd55Bex1Dpysl3Serpinf1Sfrp1LbpCd46Loxl3AcadvlCcr1CdaFgfr1CtshNpr3Thy1Col3a1AcanTgfb1i1AplnUcp2Slit3Mapk8ip1Nbl1Zfp238PtgisFlnaCol1a1Nt5eHtr2aPrkcdRnd1Nupr1CapgTp53inp1Crlf1Sfrp4Wisp2FrzbRunx1LitafPmp22Cdh13Timp1Tgfb1Gnrh1Anxa2Dhrs7cMapk12Ptgs2Tmem176bP2ry12PthlhSerpine2Adamtsl2Plscr1PtgfrXylt1NppaThbs4Nrxn1Stc2Sulf2Fbxo31Dpp4Piwil2Tbx3Runx2PrkcbP2ry1Serping1Ncf1Pdcd1lg2Sulf1G6pdMid1ip1Prdm5Smad7Vat1Fcer1gAtp1a2RGD1305645Uchl1Rab23Dab2Anxa1Tnfrsf11bPhlda3AxlCd44AoahPrrx1Dkk3Fn1Pdk4Lpar1CompPpargEcm1LOC100361596Cx3cr1Ccl2Itm2cSesn3Lpin1Nr4a3CilpIfitm1Ddr1Ddit4lC1qcAbca5Mmp14HadhCcr5Apobec1Abhd2Htr1bActn1Ncam1Nckap1lThbs1 |
| [GO:0051384 response to glucocorticoid stimulus](http://amigo.geneontology.org/cgi-bin/amigo/term_details?term=GO:0051384&session_id=) | 6.54e-03 | Ccnd1AcadmBckdhaCacna1gMgpPtgs1Serpinf1Ucp3Abcb4Slit3C1qbSfrp4Tgfb1Pfkfb1Ptgs2RelnAldobGpx3Anxa1Fn1C3Ccl2MdkAcadsBmp6 |
| [GO:0006811 ion transport](http://amigo.geneontology.org/cgi-bin/amigo/term_details?term=GO:0006811&session_id=) | 6.81e-03 | Tgfb2Hcn4Scn1aCtgfFhl1Gnao1Atp1a3Fkbp4Slc16a7Kcnk2Slc22a5TrdnCacna1gFxyd2Cysltr1Clcn1Ucp3Ccr1Got2P2ry6Thy1Trpc6Acsl1Htr2aPrkcdSlc38a3Slc6a6Cpt2Sfrp4Atp6ap1lCacna2d2Tgfb1S100a6Dhrs7cPtgs2P2ry12Scara5Serpine2Scn3bNrxn1Stc2RelnPrkcbP2ry1Fcer1gAtp1a2Abcd3Kcnt2TfAnxa1CpScn4bPpargPllpSlc22a3Slc4a1Piezo2Car4Slc25a42GabreCcr5Htr1bSlc43a2Fxyd3Thbs1 |
| [GO:0040013 negative regulation of locomotion](http://amigo.geneontology.org/cgi-bin/amigo/term_details?term=GO:0040013&session_id=) | 6.93e-03 | Cxcl13ApoeSfrp2PtprjLrp1Dpysl3Sfrp1Thy1AcanNbl1Tp53inp1Timp1Tgfb1Sulf1Cx3cr1Ccl2Ifitm1Ccr5Abhd2Thbs1 |
| [GO:0010942 positive regulation of cell death](http://amigo.geneontology.org/cgi-bin/amigo/term_details?term=GO:0010942&session_id=) | 7.35e-03 | Tgfb2Epha7CtgfBokMmp2ApoeInhbaSfrp2Ifi204C6Sfrp1Fndc1Tnfrsf12aUcp2PtgisIl18PrkcdNupr1Tp53inp1Col18a1Aldh1a1Casp12Sfrp4FrzbTgfb1Map2k6Ptgs2Plscr1TfAnxa1Phlda3Cd44Lpar1PpargItm2cCasp7Nr4a3Ccr5Thbs1Tgm2 |
| [GO:0016049 cell growth](http://amigo.geneontology.org/cgi-bin/amigo/term_details?term=GO:0016049&session_id=) | 7.69e-03 | Tgfb2CtgfFhl1Raph1Mmp2ApoeInhbaSfrp2NovPtprjSpp1S100a10Fxyd2Sfrp1CdaTnfrsf12aSlit3Nupr1Wisp2FrzbCdh13Tgfb1Serpine2NppaEmp1Igfbp6Dab2Cd44PpargDdr1Mmp14Ccr5 |
| [GO:0010243 response to organic nitrogen](http://amigo.geneontology.org/cgi-bin/amigo/term_details?term=GO:0010243&session_id=) | 8.46e-03 | Tgfb2CtgfGnao1Ccnd1Mmp2Mgst1ApoeC5ar1BckdhaHadhaGpamPtgs1Pea15aCd55Fads1Gpihbp1Sfrp1Col1a2Cyp2e1Ucp3CdaCol3a1Ucp2Col1a1Il18PrkcdCasp12Sdc2Sfrp4Timp1Pfkfb1Ptgs2NppaStc2RelnPrkcbAldobAtp1a2TfAnxa1Fzd2Pdk4PpargCcl2Lpin1Trim16Nr4a3C1sHadhApobec1Htr1bNcam1 |
| [GO:0008347 glial cell migration](http://amigo.geneontology.org/cgi-bin/amigo/term_details?term=GO:0008347&session_id=) | 8.92e-03 | Tgfb2VcanP2ry12P2ry1Fn1Ccl2Mmp14 |
| [GO:0031667 response to nutrient levels](http://amigo.geneontology.org/cgi-bin/amigo/term_details?term=GO:0031667&session_id=) | 9.02e-03 | Tgfb2Ltc4sCcnd1AcadmSfrp2BckdhaGpamCybbMgpSpp1Fads1Sfrp1Ucp3Col1a1Acsl1PrkcdTxn2Acot2Tgfb1Pfkfb1Alox5Ptgs2Hsd11b1Stc2PrkcbAbca1AldobG6pdTnfrsf11bFzd2Cd44CpPdk4PpargCcl2Slc22a3Acads |
| [GO:0030335 positive regulation of cell migration](http://amigo.geneontology.org/cgi-bin/amigo/term_details?term=GO:0030335&session_id=) | 9.08e-03 | Tgfb2VtnCxcl13PtprjLbpCcr1CtshP2ry6Col1a1Col18a1Fgf7C3ar1Cdh13Tgfb1Csf1Ptgs2FigfThbs4Srpx2Csf1rDab2Lpar1Ccl2Mmp14Nckap1lThbs1 |
| [GO:0002062 chondrocyte differentiation](http://amigo.geneontology.org/cgi-bin/amigo/term_details?term=GO:0002062&session_id=) | 9.24e-03 | Ltbp3Loxl2CtgfSfrp2Fgfr1Creb3l2AcanTgfb1PthlhSulf2Runx2Sulf1Fgf18 |
| **Cellular Component** | | |
| **GO Term** | **P-value** | **Genes** |
| [GO:0005576 extracellular region](http://amigo.geneontology.org/cgi-bin/amigo/term_details?term=GO:0005576&session_id=) | 6.09e-35 | Ltbp3Tgfb2Timp2Loxl2CdnfVcanCtgfRnase4VtnMup4Fstl3Svep1Mmp2PcolceCxcl13ApoeInhbaCfhQsox1Sfrp2Fndc5Pon3NovMyofObp3CtszFapAspnMgpLumSpp1Gdf15C6Dpysl3Serpinf1Sfrp1Col1a2LbpLamc1Fndc1Itgbl1Aebp1Loxl3FibinIgsf10F13a1CtshHampVwfFbn1ChadCol3a1AcanHfe2AplnSlit3Prrg4Ccl6Nbl1PtgisFlnaClec11aCol1a1Scpep1Nt5eGrnIl18Prss23Olr1CapgCol18a1Fgf7Crlf1BgnLOC259244PrelpC1qbPi16Fstl1Hapln1Sfrp4Wisp2LipeCd14FrzbRunx1LitafCdh13Timp1Pla2g2dTgfb1OmdGnrh1Anxa2Bmp1CorinCol14a1Csf1FigfFbln1PthlhEmilin1Serpine2Chi3l1Adamtsl2Colec11PtgfrNppaThbs4PdgfrlStc2Dpp4Epdr1PostnRelnCpxm2Lgals3Abca1Serping1Sulf1Lyz2Smoc1Fam3cLtbp2Tnfsf18CtskGpx3Col8a1RGD1305645ElnIgfbp6TfMfap5FmodAnxa1Tnfrsf11bMyocAxlScrg1C1qaCnpCol12a1CpDkk3Entpd1Fn1C3CompEcm1Ccl2MdkLoxCilpSpon1C1qcC1qtnf5LOC681309C1sActn1Olfml3Fgf18Thbs1Bmp6 |
| [GO:0044421 extracellular region part](http://amigo.geneontology.org/cgi-bin/amigo/term_details?term=GO:0044421&session_id=) | 1.48e-30 | Tgfb2Timp2Loxl2VcanCtgfVtnMup4Fstl3Mmp2PcolceApoeInhbaCfhQsox1Sfrp2Pon3MyofCtszFapAspnMgpLumSpp1Gdf15C6Dpysl3Serpinf1Sfrp1Col1a2LbpLamc1Aebp1CtshVwfFbn1ChadCol3a1AcanHfe2AplnSlit3Ccl6PtgisFlnaCol1a1Nt5eGrnIl18Col18a1Fgf7Crlf1BgnPrelpC1qbHapln1Sfrp4Wisp2LipeCd14FrzbRunx1Cdh13Timp1Tgfb1OmdGnrh1Anxa2Bmp1Col14a1Csf1FigfFbln1Emilin1Serpine2Chi3l1Adamtsl2Colec11NppaThbs4Dpp4PostnRelnCpxm2Lgals3Abca1Serping1Sulf1Smoc1Ltbp2Tnfsf18CtskGpx3Col8a1RGD1305645ElnIgfbp6TfMfap5FmodAnxa1Tnfrsf11bMyocAxlC1qaCnpCol12a1CpEntpd1Fn1C3CompEcm1Ccl2LoxCilpSpon1C1qcC1qtnf5Actn1Thbs1Bmp6 |
| [GO:0031012 extracellular matrix](http://amigo.geneontology.org/cgi-bin/amigo/term_details?term=GO:0031012&session_id=) | 8.12e-30 | Tgfb2Timp2Loxl2VcanCtgfVtnMmp2PcolceNovAspnFgfr2MgpLumSerpinf1Sfrp1Col1a2Lamc1Aebp1VwfFbn1ChadCol3a1AcanTgfb1i1Col1a1Col18a1BgnPrelpC1qbHapln1Mmp23Runx1Timp1Tgfb1OmdAnxa2Bmp1Col14a1Fbln1Emilin1Serpine2Adamtsl2Plscr1Colec11Olfml2bThbs4PostnRelnCpxm2Mfap4Lgals3Smoc1Ltbp2Loxl1Col8a1ElnTfMfap5FmodTnfrsf11bC1qaCol12a1Entpd1Fn1CompEcm1LoxCilpSpon1C1qcC1qtnf5Mmp14Thbs1 |
| [GO:0005615 extracellular space](http://amigo.geneontology.org/cgi-bin/amigo/term_details?term=GO:0005615&session_id=) | 4.15e-21 | Tgfb2Timp2VcanCtgfVtnMup4Fstl3Mmp2PcolceApoeInhbaCfhQsox1Sfrp2Pon3CtszFapMgpLumSpp1Gdf15C6Dpysl3Serpinf1Sfrp1Col1a2LbpAebp1CtshFbn1Col3a1Hfe2AplnSlit3Ccl6PtgisCol1a1GrnIl18Col18a1Fgf7Crlf1C1qbSfrp4Wisp2LipeCd14FrzbCdh13Tgfb1Gnrh1Bmp1Col14a1Csf1FigfFbln1Serpine2Chi3l1NppaThbs4RelnCpxm2Abca1Serping1Sulf1Ltbp2Tnfsf18CtskGpx3RGD1305645Igfbp6TfFmodAnxa1Tnfrsf11bMyocAxlCnpCol12a1CpFn1C3CompEcm1Ccl2LoxCilpSpon1C1qtnf5Thbs1Bmp6 |
| [GO:0005578 proteinaceous extracellular matrix](http://amigo.geneontology.org/cgi-bin/amigo/term_details?term=GO:0005578&session_id=) | 4.49e-21 | Tgfb2Timp2Loxl2VcanCtgfVtnMmp2AspnLumSfrp1Col1a2Lamc1VwfFbn1ChadCol3a1AcanCol1a1Col18a1BgnPrelpC1qbHapln1Runx1Timp1Tgfb1OmdAnxa2Bmp1Fbln1Emilin1Adamtsl2Colec11Thbs4PostnRelnLgals3Smoc1Col8a1ElnTfMfap5FmodTnfrsf11bC1qaCol12a1Entpd1Fn1CompEcm1LoxSpon1C1qcC1qtnf5 |
| [GO:0044420 extracellular matrix part](http://amigo.geneontology.org/cgi-bin/amigo/term_details?term=GO:0044420&session_id=) | 1.83e-11 | Tgfb2Timp2Loxl2LumCol1a2Lamc1Fbn1Col3a1AcanCol1a1Col18a1C1qbRunx1Timp1Anxa2Bmp1Fbln1Colec11Thbs4Smoc1Col8a1ElnTfMfap5C1qaCol12a1Entpd1Fn1LoxCilpC1qcC1qtnf5 |
| [GO:0009986 cell surface](http://amigo.geneontology.org/cgi-bin/amigo/term_details?term=GO:0009986&session_id=) | 1.11e-08 | Tgfb2Timp2Abcg1Cd53Cd28ItgamApoeC5ar1PtprjFgfr2Bace2Emr1Cd55Gpihbp1Sfrp1Cd46Ccr1VwfGot2Thy1Tnfrsf12aClec7aHfe2Nt5eGrnBgnMrc1Sfrp4Wisp2Fcgr1aCd14Cdh13Tgfb1Nfam1CorinCd4P2ry12Scara5Serpine2Nrxn1Kcnj3Sulf2Dpp4Abca1Sulf1Tnfsf18Fcer1gCsf1rAxlCd44Lpar1Car4Ccr5CtssNcam1Thbs1 |
| [GO:0044444 cytoplasmic part](http://amigo.geneontology.org/cgi-bin/amigo/term_details?term=GO:0044444&session_id=) | 6.84e-08 | Sptlc2Tgfb2Ppp1r3dRpl3Fhl3Scn1aAgpat2Ccdc3Sec16bAbcg1Acsf3CtgfBokLtc4sEno2EhhadhFstl3CmblAtp1a3Laptm5mrpl24Cyb5bMmp2Mgst1Fkbp4ApoeBcat2AcadmQsox1Hook1Fndc5Me2Dgat2BckdhaHadhaGpamCybbMyofSlc25a15TrdnCtszCacna1gFgfr2Pcp4HhatlMgpIsoc1Ptgs1Lrp1Spp1Ptcd2Bace2S100a10Slc9a9Erc1Msr1Uap1MecrFads1Dpysl3Sfrp1Soat1InmtHsdl2MceeCyp2e1Fndc1Cd46Tfb2mUcp3Abcb4AcadvlFibinCdaP2ry13Fgfr1Nudt6PhyhiplCtshSphkapPxmp2DseHampVwfGot2Myl9Thy1Tmem143Creb3l2Plod2LOC100174910Gpcpd1AplnAcsf2Ucp2Slit3Fkbp7Mapk8ip1Prrg4PtgisAcacbFlnaAtp10aSlc16a1Pex19Scpep1Pdk1ChpfAcsl1Akr1b10GrnHtr2aDhrs4L2hgdhPrkcdTmod4Epn3MlxiplTbxas1Unc93b1CapgOsgepl1Tp53inp1Txn2Fgf7Myl1Zdhhc9Slc25a20Casp12BgnLsp1Fndc3bMrc1MlycdPdk3Cpt2Ankrd23Mmp23LipeSlc25a34Suclg1Cuzd1Tlr7LitafGpnmbPdlim5Myo5bCdh13HadhbDgat1Acot2Tgfb1Sv2cRT1-DMaS100a6Snx10Egln1Pfkfb1Anxa2St3gal4CorinAlox5Csf1Dhrs7cMapk12Cd4Acox1Ptgs2P2ry12Cyp4b1Lcp1PthlhDecr1Serpine2Chi3l1Scn3bPlscr1Xylt1Hsd11b1NppaNrxn1Agpat9Fitm1Stc2Sulf2Eci1ProdhEphx2Mthfd2RilpLOC691083Bcat1Dpp4Epdr1Fads2Piwil2Fuca1PrkcbClpxLgals3P2ry1Abca1Ncf1Sulf1AldobLyz2G6pdLactb2Mid1ip1Fam3cVat1CtskTmbim1Tmem117Atp1a2RGD1305645Abcd3ElnUchl1Fdft1Sypl2Igfbp6Tmem100TfMaoaDab2Cpeb1Anxa1Nt5dc3MyocMacrod1Rpl3lCh25hMmePfkpCnpLdhdAtpaf1Lrrc10Qrsl1Tmed3MyotFn1Pde11aPdk4Lpar1PpargRab31Fahd1Cx3cr1Alox5apAdhfe1Itm2cCasp7Fam198bSlc4a1Lpin1S100a4AcadsTmem119Asah2Car4Slc25a42Abca5Acot13C1qtnf5LOC681309Oxnad1Mmp14Zadh2HadhCcr5Ech1Prtfdc1Actn1Sorbs2Sorl1Fxyd3Adam12CratGpr37Acot7CtssNckap1lThbs1Tgm2 |
| [GO:0044459 plasma membrane part](http://amigo.geneontology.org/cgi-bin/amigo/term_details?term=GO:0044459&session_id=) | 1.72e-06 | Epha7Scn1aAbcg1Aqp7Cd53Cd28Gnao1ItgamAtp1a3ApoeKcnk2Slc22a5CybbMyofPtprjCacna1gFgfr2Lrp1S100a10Emr1Fxyd2Antxr1C6Cd55Cysltr1Gpihbp1Tspan12Cd46Itgbl1Abcb4Ccr1VwfNpr3P2ry6Thy1Clec7aHfe2PtgisPex19RT1-M1-2Il18Slc38a3Slc6a6Cacna2d2LipeFcgr1aGpnmbCdh13RT1-DMaS100a6Slc16a10Anxa2CorinCsf1Cd4Ptgs2P2ry12Lcp1Scara5Serpine2Scn3bPlscr1Kcne4Kcnj3Dpp4PrkcbGpr64P2ry1Abca1Ncf1G6pdNlrp10Gna15Smad7Fcer1gAtp1a2Kcnt2TfDab2Anxa1Itga11Fzd2Cd44CpSlc26a5Scn4bFn1Has2Cx3cr1Slc4a1Ddr1Car4C1qtnf5Ccr5OsmrNcam1Thbs1 |
| [GO:0070062 extracellular vesicular exosome](http://amigo.geneontology.org/cgi-bin/amigo/term_details?term=GO:0070062&session_id=) | 3.51e-06 | PcolceMyofCol1a2Lamc1FlnaNt5eAnxa2Emilin1Dpp4Ltbp2Anxa1MyocCol12a1C3Actn1Thbs1 |
| [GO:0043230 extracellular organelle](http://amigo.geneontology.org/cgi-bin/amigo/term_details?term=GO:0043230&session_id=) | 1.24e-05 | PcolceMyofCol1a2Lamc1FlnaNt5eAnxa2Emilin1Dpp4Ltbp2Anxa1MyocCol12a1C3Actn1Thbs1 |
| [GO:0065010 extracellular membrane-bounded organelle](http://amigo.geneontology.org/cgi-bin/amigo/term_details?term=GO:0065010&session_id=) | 1.24e-05 | PcolceMyofCol1a2Lamc1FlnaNt5eAnxa2Emilin1Dpp4Ltbp2Anxa1MyocCol12a1C3Actn1Thbs1 |
| [GO:0005737 cytoplasm](http://amigo.geneontology.org/cgi-bin/amigo/term_details?term=GO:0005737&session_id=) | 4.62e-05 | Car3Impa2Sptlc2Tgfb2Grk5Ppp1r3dRpl3Fhl3Scn1aAgpat2Ccdc3Sec16bAbcg1Acsf3CtgfFhl1Aqp7BokLtc4sEno2EhhadhFstl3CmblCcnd1Atp1a3Laptm5Svep1mrpl24Cyb5bMmp2Mgst1Fkbp4ApoeBcat2AcadmCfhAnks1bQsox1Hook1Gfpt2Fndc5Me2Dgat2Actg2Tsc22d4BckdhaHadhaGpamCybbMyofSlc25a15TrdnCtszCacna1gFgfr2Pcp4HhatlMgpIsoc1Ptgs1Pea15aLrp1Spp1Rccd1Ifi204Ptcd2Bace2S100a10Slc9a9Erc1Acy1Msr1Uap1MecrBex1Fads1Dpysl3Sfrp1Soat1ZyxInmtHsdl2MceeDusp13Cyp2e1Fndc1Cd46Aebp1Tfb2mUcp3Abcb4AcadvlFibinCdaP2ry13Cotl1Fgfr1Tpm2F13a1Nudt6PhyhiplCtshSphkapPxmp2DseHampVwfGot2Myl9Thy1Tmem143Creb3l2HpgdsTgfb1i1Plod2LOC100174910Gpcpd1AplnAcsf2Ucp2Mif4gdSlit3Fkbp7Mapk8ip1Prrg4EnahPtgisAcacbFlnaCol1a1Atp10aSlc16a1Pex19Scpep1Pdk1ChpfNt5eAcsl1Akr1b10GrnIl18Htr2aDhrs4L2hgdhPrkcdTmod4Epn3MlxiplTbxas1Unc93b1Arpc1bSbsnCapgOsgepl1Tp53inp1Txn2Fgf7Crlf1Myl1Zdhhc9Aldh1a1Slc25a20Casp12BgnLsp1Fndc3bTaglnMrc1MlycdPdk3Cpt2Sfrp4Ankrd23Mmp23Wisp2LipeSlc25a34Suclg1Cuzd1Tlr7LitafGpnmbTppp3Pdlim5Nt5c1aMyo5bCdh13HadhbDgat1Acot2Tgfb1Sv2cRT1-DMaS100a6Snx10Egln1Tubb6Pfkfb1Anxa2St3gal4CorinAlox5Csf1Dhrs7cStk38lMapk12Cd4Acox1Ptgs2Klhdc8bP2ry12Cyp4b1Lcp1PthlhDecr1Serpine2Chi3l1Scn3bPlscr1Elmo1PtgfrXylt1Hsd11b1NppaNrxn1Agpat9Pcbd1Fitm1Stc2Dbn1Sulf2Eci1ProdhSrpx2Pls3Ephx2Mthfd2RilpLOC691083Bcat1Dpp4Epdr1Fads2Piwil2Runx2RelnFuca1PrkcbClpxGas7Gpr64Lgals3P2ry1Abca1Ncf1Sulf1AldobLyz2G6pdLactb2Nlrp10Mid1ip1Fam3cSmad7Vat1CtskTmbim1Tmem117Atp1a2RGD1305645Abcd3ElnUchl1Fdft1Sypl2Igfbp6Tmem100Cd248TfMaoaDab2Cpeb1Anxa1Nt5dc3MyocMacrod1Rpl3lCh25hPhlda3MmePfkpFzd2CnpLdhdScrn1Atpaf1Spag1Lrrc10Qrsl1Tmed3MyotFn1Pde11aPdk4Lpar1PpargLrrc16aRab31Fahd1LOC100361596Cx3cr1Alox5apAdhfe1Ccl2Csdc2Itm2cCasp7Fam198bHspb3Slc4a1Lpin1Trim16S100a4Trim7AspaAcadsTmem119Asah2Car4Cdkl2Ddit4lSlc25a42Abca5Acot13Tsc22d1C1qtnf5LOC681309Oxnad1Mmp14Zadh2HadhCcr5Apobec1Ech1Prtfdc1Htr1bActn1Sntb2Sorbs2Sorl1Fxyd3Adam12CratGpr37Acot7GptCtssNckap1lThbs1Tgm2Bmp6 |
| [GO:0005581 collagen](http://amigo.geneontology.org/cgi-bin/amigo/term_details?term=GO:0005581&session_id=) | 8.28e-05 | LumCol1a2Col3a1Col1a1Col18a1C1qbBmp1Colec11Col8a1C1qaCol12a1LoxC1qcC1qtnf5 |
| [GO:0005739 mitochondrion](http://amigo.geneontology.org/cgi-bin/amigo/term_details?term=GO:0005739&session_id=) | 6.17e-04 | Sptlc2Abcg1Acsf3BokEhhadhmrpl24Cyb5bMgst1Bcat2AcadmMe2Dgat2BckdhaHadhaGpamCybbSlc25a15Ptcd2MecrHsdl2MceeCyp2e1Fndc1Tfb2mUcp3AcadvlNudt6PhyhiplSphkapPxmp2Got2Tmem143LOC100174910Acsf2Ucp2Slit3Mapk8ip1AcacbSlc16a1Pdk1Acsl1Akr1b10GrnDhrs4L2hgdhPrkcdOsgepl1Txn2Slc25a20MlycdPdk3Cpt2LipeSlc25a34Suclg1HadhbAcot2Mapk12Acox1P2ry12Decr1Eci1ProdhMthfd2RilpLOC691083Bcat1ClpxLgals3P2ry1Lactb2Vat1Abcd3ElnTfMaoaAnxa1Nt5dc3Macrod1CnpLdhdAtpaf1Lrrc10Qrsl1Pdk4Fahd1Adhfe1Lpin1AcadsAsah2Slc25a42Acot13Oxnad1Zadh2HadhEch1Adam12CratTgm2 |
| [GO:0005604 basement membrane](http://amigo.geneontology.org/cgi-bin/amigo/term_details?term=GO:0005604&session_id=) | 8.23e-04 | Tgfb2Timp2Loxl2Lamc1Fbn1AcanCol18a1Runx1Timp1Anxa2Fbln1Thbs4Smoc1TfEntpd1Fn1 |
| [GO:0005783 endoplasmic reticulum](http://amigo.geneontology.org/cgi-bin/amigo/term_details?term=GO:0005783&session_id=) | 3.22e-03 | Sptlc2Agpat2Ccdc3Sec16bLtc4sAtp1a3Mgst1Fndc5Dgat2CybbTrdnCtszHhatlMgpPtgs1Bace2S100a10Fads1Soat1Cyp2e1FibinP2ry13DseVwfThy1Creb3l2Plod2Fkbp7Mapk8ip1PtgisAtp10aAcsl1PrkcdTbxas1Unc93b1Casp12Fndc3bMmp23Tlr7HadhbDgat1Dhrs7cCd4Ptgs2Cyp4b1Chi3l1Xylt1Hsd11b1Agpat9Fitm1Stc2Sulf2Dpp4Fads2Ncf1Sulf1AldobLyz2Tmem117Uchl1Fdft1MyocCh25hTmed3Alox5apTmem119Car4Fxyd3CratGpr37 |
| **Molecular Function** | | |
| **GO Term** | **P-value** | **Genes** |
| [GO:0097367 carbohydrate derivative binding](http://amigo.geneontology.org/cgi-bin/amigo/term_details?term=GO:0097367&session_id=) | 4.65e-12 | VcanCtgfItgamPcolceCxcl13ApoeCfhDpysl3Lyve1Sfrp1Fgfr1AcanFgf7BgnPrelpFstl1Hapln1GpnmbPfkfb1Serpine2Chi3l1Thbs4PostnLtbp2PfkpCd44Fn1CompCcl2MdkLOC681309Ncam1Thbs1 |
| [GO:0005539 glycosaminoglycan binding](http://amigo.geneontology.org/cgi-bin/amigo/term_details?term=GO:0005539&session_id=) | 4.81e-11 | VcanCtgfItgamPcolceCxcl13ApoeCfhDpysl3Lyve1Sfrp1Fgfr1AcanFgf7BgnPrelpFstl1Hapln1GpnmbSerpine2Thbs4PostnLtbp2Cd44Fn1CompCcl2MdkLOC681309Ncam1Thbs1 |
| [GO:1901681 sulfur compound binding](http://amigo.geneontology.org/cgi-bin/amigo/term_details?term=GO:1901681&session_id=) | 3.12e-09 | CtgfLtc4sItgamPcolceMgst1Cxcl13ApoeCfhDpysl3Sfrp1Fgfr1AcacbFgf7PrelpSlc6a6Fstl1GpnmbSerpine2Thbs4PostnLtbp2Gpx3Fn1CompCcl2MdkLOC681309Ncam1Thbs1 |
| [GO:0005488 binding](http://amigo.geneontology.org/cgi-bin/amigo/term_details?term=GO:0005488&session_id=) | 1.93e-08 | Car3Impa2Cadm3AspdhTgfb2Grk5Timp2Ppp1r3dCndp1Loxl2CdnfFcgr2aCnn1VcanEpha7Hcn4Wbp5Fhl3Scn1aAbcg1Csrp2CtgfLy49si2Fhl1BokRnase4VtnLtc4sRaph1Cd28Eno2Gnao1EhhadhItgamMup4Fstl3Ccnd1Atp1a3Svep1Cyb5bMmp2PcolceMgst1Cxcl13Fkbp4ApoeInhbaAcadmCfhAnks1bHook1Sfrp2Gfpt2PtafrFndc5Me2Pon3NovC5ar1Slc22a5Dgat2Actg2BckdhaMafbPrg4HadhaCybbMyofPtprjObp3Cacna1gFapAspnFgfr2Pcp4MgpClec3aLumEtv5Ptgs1Lrp1Spp1Ifi204S100a10Emr1Erc1Arl11Adam1aGdf15Tcea3Fxyd2Antxr1Acy1Msr1Uap1MecrBex1Elk1Fads1Dpysl3Gpihbp1Lyve1Sfrp1Col1a2LbpSoat1Fbln2Lamc1Hsdl2Cyp2e1Cd46Itgbl1Aebp1Loxl3Abcb4AcadvlActr3bCcr1CdaCotl1Fgfr1Tpm2F13a1Rab3il1CtshTiam1SphkapPxmp2HampVwfFbn1Got2Myl9Npr3Klrb1cThy1Col3a1Creb3l2Zfp462HpgdsAcanTgfb1i1Plekho2Plod2Efhd2Clec7aGpcpd1AplnAcsf2Mif4gdMrc2Slit3Fkbp7Mapk8ip1Prrg4Ccl6Nbl1Efemp2Zfp238EnahPtgisAcacbFlnaClec11aCol1a1Slc16a1Pex19Osbpl6Myo3bTrpc6Pdk1Mybl1Nt5eAcsl1Akr1b10GrnAsb4Il18Htr2aDhrs4PrkcdTmod4Epn3MlxiplTbxas1Prrx2Unc93b1RGD1559864Olr1Arpc1bRnd1Nupr1CapgCol18a1Rad23aTxn2Fgf7Crlf1Myl1Zdhhc9Aldh1a1Casp12BgnLOC259244TaglnSdc2Tgm1Rnf207Mrc1MlycdPrelpSlc6a6C1qbPdk3Fstl1Hapln1C3ar1TfecSfrp4Ankrd23Mmp23Wisp2Cacna2d2LipeNav3Fcgr1aSuclg1Cd14FrzbRunx1Tlr7LitafGpnmbTppp3Pdlim5Nt5c1aMyo5bCyth4Cdh13HadhbDgat1Timp1Acot2Pla2g2dTgfb1S100a6Snx10LOC689800Egln1Tubb6Gnrh1Pfkfb1Anxa2Bmp1St3gal4GamtMap2k6Alox5Csf1Dhrs7cCntnap5aStk38lMapk12Cd4Acox1Ptgs2FigfCcbp2Fbln1Aox3Cyp4b1Pmepa1Myl6Lcp1Irx4PthlhDecr1Emilin1Serpine2Chi3l1Zim1Adamtsl2Scn3bPlscr1Elmo1Colec11Fxyd5Olfml2bHsd11b1NppaThbs4Nrxn1Pcbd1Stc2Dbn1Eci1ProdhSrpx2Pls3Ephx2Mthfd2Fbxo31RilpBcat1Dpp4Epdr1Fads2Piwil2Tbx3PostnRunx2RelnCpxm2Fuca1PrkcbClpxGas7Sdr39u1Lgals3P2ry1Abca1Serping1Sulf1AldobG6pdLactb2Nlrp10Mid1ip1Gna15Etv1Smoc1Prdm5Smad7Vat1Dip2cLtbp2Tnfsf18Fcer1gLonrf1Gpx3Csf1rAtp1a2TyrobpAbcd3Kcnt2ElnSat1Uchl1Igfbp6GdaRab23Cd248TfMaoaDab2Cpeb1Anxa1Nt5dc3Tnfrsf11bPpicGpr183Ch25hPhlda3AxlMmePfkpFzd2CnpCd44LdhdPrrx1CpSpag1Qrsl1Entpd1Cdh22Klrb1aSlc26a5Scn4bFn1Pde11aC3Pdk4Lpar1CompPpargEcm1Rab31Fahd1LOC100361596Alox5apAdhfe1Ccl2Csdc2Itm2cPank1Slc22a3Slc4a1Lpin1Trim16MdkNr4a3LoxS100a4Trim7AspaAcadsGba3Tlr5Ddr1Car4Cdkl2Abca5C1qtnf5LOC681309Raver2Mmp14Zadh2C1sHadhCcr5Aff3Apobec1Ech1Prtfdc1Htr1bActn1Sntb2Sorbs2Fkbp10Fxyd3OsmrAdam12CratLOC688582Fgf18GptNcam1Nckap1lThbs1Rbp7Tgm2Bmp6 |
| [GO:0008201 heparin binding](http://amigo.geneontology.org/cgi-bin/amigo/term_details?term=GO:0008201&session_id=) | 6.63e-08 | CtgfItgamPcolceCxcl13ApoeCfhSfrp1Fgfr1Fgf7PrelpFstl1GpnmbSerpine2Thbs4PostnLtbp2Fn1CompCcl2MdkLOC681309Ncam1Thbs1 |
| [GO:0005515 protein binding](http://amigo.geneontology.org/cgi-bin/amigo/term_details?term=GO:0005515&session_id=) | 7.65e-08 | Impa2Cadm3Tgfb2Timp2Ppp1r3dLoxl2CdnfFcgr2aCnn1Epha7Hcn4Wbp5Fhl3Abcg1CtgfFhl1BokVtnLtc4sCd28Eno2Gnao1EhhadhItgamFstl3Ccnd1Atp1a3Mmp2PcolceMgst1Cxcl13Fkbp4ApoeInhbaAcadmCfhAnks1bHook1Sfrp2Fndc5Pon3NovC5ar1Slc22a5Dgat2BckdhaMafbHadhaCybbPtprjCacna1gFapFgfr2Pcp4MgpLumLrp1Spp1S100a10Erc1Gdf15Fxyd2Antxr1MecrBex1Dpysl3Gpihbp1Sfrp1Col1a2LbpCyp2e1Cd46Itgbl1Aebp1Abcb4Actr3bCcr1CdaCotl1Fgfr1Tpm2Rab3il1CtshTiam1SphkapPxmp2HampVwfGot2Npr3Thy1Col3a1HpgdsTgfb1i1Clec7aAplnMif4gdSlit3Mapk8ip1Ccl6Nbl1EnahFlnaClec11aCol1a1Slc16a1Pex19Pdk1GrnAsb4Il18Htr2aDhrs4PrkcdTmod4MlxiplPrrx2Unc93b1Arpc1bRnd1CapgCol18a1Rad23aTxn2Fgf7Crlf1Aldh1a1Casp12TaglnSdc2MlycdC1qbC3ar1TfecSfrp4Ankrd23Wisp2LipeFcgr1aSuclg1Cd14FrzbRunx1LitafGpnmbTppp3Pdlim5Myo5bCdh13HadhbAcot2Tgfb1S100a6Gnrh1Pfkfb1Anxa2St3gal4GamtMap2k6Alox5Csf1Cntnap5aMapk12Cd4Acox1Ptgs2FigfCcbp2Pmepa1Lcp1PthlhEmilin1Serpine2Scn3bPlscr1Elmo1Fxyd5Olfml2bNppaThbs4Nrxn1Pcbd1Stc2Dbn1Eci1Srpx2Pls3Ephx2Fbxo31RilpBcat1Dpp4Runx2RelnPrkcbClpxGas7Lgals3P2ry1Abca1Serping1AldobG6pdMid1ip1Gna15Prdm5Smad7Dip2cLtbp2Tnfsf18Fcer1gCsf1rAtp1a2TyrobpAbcd3Uchl1Igfbp6Dab2Anxa1Tnfrsf11bAxlFzd2Cd44Prrx1CpSlc26a5Scn4bFn1C3Lpar1CompPpargEcm1LOC100361596Alox5apCcl2Slc22a3Slc4a1Lpin1Trim16MdkS100a4Tlr5Ddr1C1qtnf5Mmp14Ccr5Apobec1Ech1Prtfdc1Actn1Fxyd3OsmrAdam12CratFgf18Ncam1Nckap1lThbs1Tgm2Bmp6 |
| [GO:0005102 receptor binding](http://amigo.geneontology.org/cgi-bin/amigo/term_details?term=GO:0005102&session_id=) | 1.29e-07 | Tgfb2Timp2CdnfEpha7CtgfVtnGnao1EhhadhCxcl13Fkbp4ApoeInhbaAnks1bSfrp2Fndc5NovPtprjSpp1Gdf15MecrSfrp1LbpItgbl1Tiam1HampVwfThy1Tgfb1i1AplnSlit3Ccl6FlnaClec11aGrnIl18Dhrs4Unc93b1Rnd1Fgf7Crlf1MlycdGpnmbMyo5bAcot2Tgfb1Gnrh1Csf1Cntnap5aAcox1FigfPthlhSerpine2Plscr1NppaThbs4Nrxn1Stc2Srpx2Ephx2Dpp4RelnPrkcbP2ry1Gna15Smad7Tnfsf18Uchl1Dab2Tnfrsf11bCd44C3PpargEcm1Ccl2Lpin1MdkS100a4Tlr5Mmp14Ech1Actn1CratFgf18Ncam1Thbs1Bmp6 |
| [GO:0046872 metal ion binding](http://amigo.geneontology.org/cgi-bin/amigo/term_details?term=GO:0046872&session_id=) | 1.58e-06 | Car3Impa2Timp2Cndp1Loxl2VcanScn1aEno2Gnao1Atp1a3Svep1Cyb5bApoeMe2Pon3BckdhaAspnMgpPtgs1Lrp1S100a10Emr1Antxr1Acy1Fads1Col1a2Cyp2e1Loxl3F13a1Fbn1Myl9Col3a1HpgdsAcanPlod2Efhd2Slit3Fkbp7Prrg4Efemp2PtgisCol1a1Nt5ePrkcdTbxas1RGD1559864Myl1Tgm1Fstl1Cacna2d2Runx1Tppp3Nt5c1aTimp1Pla2g2dS100a6Egln1Anxa2Bmp1Alox5Stk38lMapk12Ptgs2Fbln1Aox3Cyp4b1Myl6Lcp1Plscr1Thbs4Nrxn1Pls3Ephx2Mthfd2Epdr1Fads2RelnClpxSulf1Lactb2Gna15Ltbp2Atp1a2Cd248TfAnxa1Nt5dc3Ch25hPfkpCpCdh22Fn1Pde11aCompFahd1Adhfe1LoxS100a4AspaDdr1LOC681309Mmp14C1sEch1Prtfdc1Actn1Fkbp10LOC688582Tgm2 |
| [GO:0005201 extracellular matrix structural constituent](http://amigo.geneontology.org/cgi-bin/amigo/term_details?term=GO:0005201&session_id=) | 3.47e-05 | Col1a2Lamc1Fbn1Col3a1AcanCol1a1Emilin1ElnCol12a1Fn1Comp |
| [GO:0043167 ion binding](http://amigo.geneontology.org/cgi-bin/amigo/term_details?term=GO:0043167&session_id=) | 1.70e-04 | Car3Impa2Grk5Timp2Cndp1Loxl2VcanEpha7Hcn4Fhl3Scn1aAbcg1Csrp2CtgfFhl1Ltc4sEno2Gnao1ItgamAtp1a3Svep1Cyb5bMmp2PcolceMgst1Cxcl13Fkbp4ApoeAcadmCfhMe2Pon3Slc22a5Actg2BckdhaHadhaCybbAspnMgpPtgs1Lrp1S100a10Emr1Arl11Adam1aTcea3Antxr1Acy1MecrFads1Dpysl3Sfrp1Col1a2Soat1Cyp2e1Aebp1Loxl3Abcb4AcadvlActr3bCdaFgfr1F13a1Fbn1Got2Myl9Col3a1Zfp462HpgdsAcanTgfb1i1Plod2Efhd2Acsf2Slit3Fkbp7Prrg4Efemp2Zfp238PtgisAcacbCol1a1Myo3bTrpc6Pdk1Nt5eAcsl1Akr1b10Htr2aPrkcdTbxas1RGD1559864Rnd1Fgf7Myl1Zdhhc9Tgm1Rnf207PrelpPdk3Fstl1Mmp23Cacna2d2Nav3Suclg1Runx1GpnmbTppp3Pdlim5Nt5c1aMyo5bHadhbDgat1Timp1Pla2g2dS100a6Snx10Egln1Tubb6Pfkfb1Anxa2Bmp1Map2k6Alox5Stk38lMapk12Cd4Acox1Ptgs2Fbln1Aox3Cyp4b1Myl6Lcp1Decr1Serpine2Chi3l1Zim1Adamtsl2Plscr1Thbs4Nrxn1ProdhPls3Ephx2Mthfd2Epdr1Fads2PostnRunx2RelnCpxm2Fuca1PrkcbClpxP2ry1Abca1Sulf1AldobLactb2Nlrp10Gna15Prdm5Vat1Ltbp2Lonrf1Gpx3Csf1rAtp1a2Abcd3Kcnt2Sat1GdaRab23Cd248TfMaoaDab2Anxa1Nt5dc3Ch25hPhlda3AxlMmePfkpLdhdCpSpag1Qrsl1Entpd1Cdh22Fn1Pde11aPdk4CompPpargRab31Fahd1Alox5apAdhfe1Ccl2Itm2cPank1Trim16MdkNr4a3LoxS100a4Trim7AspaAcadsGba3Ddr1Car4Cdkl2Abca5LOC681309Mmp14Zadh2C1sHadhApobec1Ech1Prtfdc1Htr1bActn1Sorbs2Fkbp10Adam12LOC688582GptNcam1Thbs1Tgm2 |
| [GO:0005509 calcium ion binding](http://amigo.geneontology.org/cgi-bin/amigo/term_details?term=GO:0005509&session_id=) | 1.42e-03 | VcanSvep1AspnMgpLrp1S100a10Emr1Fbn1Myl9HpgdsAcanEfhd2Slit3Fkbp7Prrg4Efemp2RGD1559864Myl1Fstl1Runx1Tppp3Pla2g2dS100a6Anxa2Bmp1Fbln1Myl6Lcp1Plscr1Thbs4Nrxn1Pls3Epdr1Sulf1Ltbp2Cd248Anxa1Cdh22CompS100a4LOC681309Mmp14C1sActn1Fkbp10LOC688582 |
| [GO:0016491 oxidoreductase activity](http://amigo.geneontology.org/cgi-bin/amigo/term_details?term=GO:0016491&session_id=) | 1.90e-03 | AspdhLoxl2EhhadhMgst1AcadmQsox1Me2BckdhaHadhaCybbPtgs1MecrFads1Hsdl2Cyp2e1Loxl3AcadvlPlod2PtgisAkr1b10Dhrs4L2hgdhTbxas1Txn2Aldh1a1HadhbEgln1Alox5Dhrs7cAcox1Ptgs2Aox3Cyp4b1Decr1Hsd11b1Pcbd1ProdhMthfd2Fads2Ncf1G6pdVat1Loxl1Gpx3Gpx8Fdft1MaoaCh25hLdhdCpAlox5apAdhfe1LoxAcadsOxnad1Zadh2Hadh |
