# Supplementary material for: Transcriptional profiling of left ventricle and peripheral blood mononuclear cells in a rat model of postinfarction heart failure
Source: BMC Med Genomics. 2013 Nov 8;6:49. doi: 10.1186/1755-8794-6-49 (PMC4226214; doi:10.1186/1755-8794-6-49)
Supplement: Additional file 4 — Differentially expressed genes related to GO:0031012: extracellular matrix. [file 1755-8794-6-49-S4.doc]

**Additional 4.** Differentially expressed genes related to GO:0031012: extracellular matrix

| **Affimetrix**  **Transcript Cluster ID** | **Gene name** | **Gene Symbol** | **RefSeq** | **p-value** | **Fold change**  **L-MI vs Sham** |
| --- | --- | --- | --- | --- | --- |
| 10797660 | asporin | Aspn | NM_001014008 | 1.61654e-007 | 1.8357 |
| 10774015 | AE binding protein 1 | Aebp1 | NM_001100970 | 1.8951e-005 | 1.41087 |
| 10834670 | ADAMTS-like 2 | Adamtsl2 | ENSRNOT00000036995 | 3.07191e-007 | 1.84132 |
| 10911287 | annexin A2 | Anxa2 | NM_019905 | 2.04229e-005 | 1.37475 |
| 10708021 | aggrecan | Acan | NM_022190 | 0.00413875 | 1.57095 |
| 10909442 | C1q and tumor necrosis factor related protein 5 | C1qtnf5 | NM_001012123 | 6.61582e-007 | 1.47017 |
| 10880731 | complement component 1, q subcomponent, C chain | C1qc | NM_001008524 | 1.10845e-005 | 1.89957 |
| 10880727 | complement component 1, q subcomponent, B chain | C1qb | NM_019262 | 3.13341e-006 | 1.90866 |
| 10750685 | collagen, type VIII, alpha 1 | Col8a1 | NM_001107100 | 3.06533e-008 | 2.12977 |
| 10853559 | collagen, type I, alpha 2 | Col1a2 | NM_053356 | 5.62041e-005 | 1.57612 |
| 10737532 | collagen, type I, alpha | Col1a1 | NM_053304 | 6.06025e-006 | 2.31371 |
| 10829378 | collagen, type XVIII, alpha 1 | Col18a1 | NM_053489 | 7.6146e-005 | 1.3063 |
| 10918869 | collagen, type XII, alpha 1 | Col12a1 | ENSRNOT00000051159 | 3.37048e-008 | 2.91044 |
| 10880734 | complement component 1, q subcomponent, A chain | C1qa | NM_001008515 | 1.71445e-005 | 1.76752 |
| 10896558 | collagen, type XIV, alpha 1 | Col14a1 | NM_001130548 | 1.01042e-007 | 1.95689 |
| 10923052 | collagen, type III, alpha 1 | Col3a1 | NM_032085 | 1.35234e-005 | 1.46442 |
| 10889446 | collectin sub-family member 11 | Colec11 | BC158664 | 3.64341e-007 | 1.96674 |
| 10910926 | cartilage intermediate layer protein | Cilp | NM_001108161 | 1.44333e-005 | 2.36627 |
| 10737513 | chondroadherin | Chad | NM_019164 | 0.00282446 | 1.71542 |
| 10726255 | carboxypeptidase X | Cpxm2 | NM_001106306 | 0.00374554 | 1.5229 |
| 10717233 | connective tissue growth factor | Ctgf | NM_022266 | 6.73292e-006 | 3.11136 |
| 10888953 | elastin microfibril interfacer 1 | Emilin1 | NM_001106710 | 2.92703e-006 | 1.52083 |
| 10757726 | elastin | Eln | NM_012722 | 1.81801e-005 | 1.40551 |
| 10825022 | extracellular matrix protein 1 | Ecm1 | NM_053882 | 4.72019e-007 | 1.77236 |
| 10715258 | ectonucleoside triphosphate diphosphohydrolase 1 | Entpd1 | NM_022587 | 1.37729e-005 | 1.42507 |
| 10764050 | fibromodulin | Fmod | NM_080698 | 1.47187e-005 | 2.21249 |
| 10849327 | fibrillin 1 | Fbn1 | NM_031825 | 5.21593e-007 | 2.2484 |
| 10898315 | fibulin 1 | Fbln1 | NM_001127547 | 1.19089e-005 | 1.50028 |
| 10726172 | fibroblast growth factor receptor 2 | Fgfr2 | NM_012712 | 0.00143484 | 1.51584 |
| 10928761 | fibronectin 1 | Fn1 | NM_019143 | 6.14622e-008 | 2.93094 |
| 10781304 | lysyl oxidase-like 2 | Loxl2 | NM_001106047 | 1.5788e-007 | 1.65636 |
| 10891165 | latent transforming growth factor beta binding protein 2 | Ltbp2 | NM_021586 | 7.85083e-009 | 2.60937 |
| 10917883 | lysyl oxidase-like 1 | Loxl1 | NM_001012125 | 6.55422e-008 | 2.85464 |
| 10768668 | laminin, gamma 1 | Lamc1 | NM_053966 | 5.06972e-007 | 1.31875 |
| 10895083 | lumican | Lum | NM_031050 | 0.000138683 | 1.36682 |
| 10882168 | matrix metallopeptidase 23 | Mmp23 | NM_053606 | 0.000132951 | 1.31047 |
| 10809540 | matrix metallopeptidase | Mmp2 | NM_031054 | 7.47691e-007 | 1.3867 |
| 10780205 | matrix metallopeptidase 14 | Mmp14 | NM_031056 | 0.00186355 | 1.33807 |
| 10815369 | periostin, osteoblast specific factor | Postn | NM_001108550 | 1.23232e-009 | 7.99435 |
| 10760971 | procollagen C-endopeptidase enhancer | Pcolce | NM_019237 | 2.75247e-007 | 1.52959 |
| 10912218 | phospholipid scramblase 1 | Plscr1 | NM_057194 | 5.33785e-005 | 1.37863 |
| 10767763 | proline/arginine-rich end leucine-rich repeat protein | Prelp | NM_053385 | 2.94075e-006 | 1.59115 |
| 10853020 | reelin | Reln | NM_080394 | 0.000202246 | 1.30906 |
| 10753222 | runt-related transcription factor 1 | Runx1 | NM_017325 | 6.98171e-006 | 1.53917 |
| 10744939 | serine (or cysteine) peptidase inhibitor, clade F | Serpinf1 | NM_177927 | 2.74527e-008 | 1.87879 |
| 10929288 | serine (or cysteine) peptidase inhibitor, clade E | Serpine2 | NM_019197 | 1.16775e-005 | 1.75071 |
| 10885680 | SPARC related modular calcium binding 1 | Smoc1 | NM_001002835 | 4.71424e-005 | 1.37661 |
| 10792344 | secreted frizzled-related protein 1 | Sfrp1 | ENSRNOT00000024128 | 1.25497e-009 | 3.4753 |
| 10710067 | spondin 1, extracellular matrix protein | Spon1 | NM_172067 | 0.000603473 | 1.30047 |
| 10797657 | osteomodulin | Omd | NM_031817 | 1.22814e-005 | 1.56323 |
| 10765480 | olfactomedin-like 2B | Olfml2b | NM_001107195 | 0.000393199 | 1.497 |
| 10820434 | thrombospondin 4 | Thbs4 | ENSRNOT00000065224 | 8.81769e-009 | 6.1197 |
| 10749484 | TIMP metallopeptidase inhibitor 2 | Timp2 | NM_021989 | 4.63114e-007 | 1.47623 |
| 10936482 | TIMP metallopeptidase inhibitor 1 | Timp1 | NM_053819 | 2.79585e-007 | 2.34969 |
| 10770577 | transforming growth factor, beta 2 | Tgfb2 | NM_031131 | 7.8036e-005 | 2.01264 |
| 10705213 | transforming growth factor, beta 1 | Tgfb1 | NM_021578 | 1.79043e-005 | 1.31202 |
| 10711364 | transforming growth factor beta 1 induced transcript | Tgfb1i1 | NM_001191840 | 0.00118498 | 1.30778 |
| 10734242 | microfibrillar-associated protein 4 | Mfap4 | NM_001034124 | 5.00403e-006 | 1.49139 |
| 10804463 | lysyl oxidase | Lox | NM_017061 | 2.46257e-006 | 1.72888 |
| 10779673 | lectin, galactoside-binding, soluble, 3 | Lgals3 | NM_031832 | 5.78048e-006 | 1.67512 |
| 10896541 | nephroblastoma overexpressed gene | Nov | NM_030868 | 0.000351537 | 1.43374 |
| 10736273 | vitronectin | Vtn | NM_019156 | 2.24722e-006 | -1.43319 |
| 10820282 | versican | Vcan | NM_001170558 | 1.92515e-006 | 2.23098 |
| 10785063 | bone morphogenetic protein | Bmp1 | NM_031323 | 2.7309e-005 | 1.35244 |
| 10812399 | hyaluronan and proteoglycan link protein 1 | Hapln1 | NM_019189 | 0.00191204 | 1.52535 |
| 10935890 | biglycan | Bgn | NM_017087 | 8.8887e-010 | 2.21586 |
| 10866512 | matrix Gla protein | Mgp | NM_012862 | 5.70129e-009 | 1.64017 |
| 10858886 | Vwf | Vwf | ENSRNOT00000026643 | 9.78058e-006 | 1.33454 |
| 10940446 | Cartilage oligomeric matrix protein | Comp | NM_012834.1 | 1.06381e-005 | 1.95396 |
| 10940627 | Thrombospondin 1 | Thbs1 | NM_021989 | 4.7904e-006 | 2.7268 |
| 10858499 | microfibrillar associated protein 5 | Mfap5 | NM_001108644 | 7.56962e-009 | 2.29512 |
| 10919637 | transferrin | Tf | NM_001013110 | 1.0152e-005 | 1.59417 |
| 10903725 | tumor necrosis factor receptor superfamily, member 11b | Tnfrsf11b | NM_012870 | 2.02959e-008 | 2.32907 |
