# Supplementary material for: Transcriptional profiling of left ventricle and peripheral blood mononuclear cells in a rat model of postinfarction heart failure
Source: BMC Med Genomics. 2013 Nov 8;6:49. doi: 10.1186/1755-8794-6-49 (PMC4226214; doi:10.1186/1755-8794-6-49)
Supplement: Additional file 5 — Transcripts differentially expressed in PBMCs between rats with large-size of infarction and sham-operated ones. [file 1755-8794-6-49-S5.doc]

**Additional file 5: Transcripts differentially expressed in PBMCs between rats with large-size of infarction and sham-operated ones. P-value have been adjusted using False Discovery Rate (FDR) of 5%**

| No. | Transcript Cluster ID | Gene_assignment | Gene Symbol | p-value | Fold-Change |
| --- | --- | --- | --- | --- | --- |
| Adnnotated transcripts ID | | | | | |
| 1 | 10866052 | NM_001029908 // Klrc3 // killer cell lectin-like receptor subfamily C, member 3 | Klrc3 | 0.0112993 | 1.9013 |
| 2 | 10866056 | NM_019261 // Klrc2 // killer cell lectin-like receptor subfamily C, member 2 | Klrc2 | 0.0154334 | 1.83363 |
| 3 | 10866076 | NM_001012648 // Klri2 // killer cell lectin-like receptor family I member 2 | Klri2 | 0.0442444 | 1.822 |
| 4 | 10866061 | NM_001037441 // Klrc1 // killer cell lectin-like receptor subfamily C, member 1 | Klrc1 | 0.016142 | 1.71645 |
| 5 | 10859090 | ENSRNOT00000049620 // LOC689800 // similar to osteoclast inhibitory lectin | LOC689800 | 0.0135265 | 1.56126 |
| 6 | 10814703 | NR_001567 // Terc // telomerase RNA component | Terc | 0.00561934 | 1.38365 |
| 7 | 10866144 | XM_002726462 // LOC100364751 // immunoreceptor Ly49si3-like | LOC100364751 | 0.0484937 | 1.37806 |
| 8 | 10858133 | NM_001107882 // March8 // membrane-associated ring finger (C3HC4) 8 | March8 | 0.0229928 | 1.35752 |
| 9 | 10798509 | NM_021860 // Olr1654 // olfactory receptor 1654 | Olr1654 | 0.0414028 | 1.34466 |
| 10 | 10796440 | NM_022224 // Pter // phosphotriesterase related | Pter | 0.03816 | 1.32955 |
| 11 | 10726408 | ENSRNOT00000038176 // Mki67 // antigen identified by monoclonal antibody Ki-67 | Mki67 | 0.0224917 | 1.31316 |
| 12 | 10749495 | NM_139096 // Lgals3bp // lectin, galactoside-binding, soluble, 3 binding protein | Lgals3bp | 0.0449322 | 1.31179 |
| 13 | 10779638 | NM_001106028 // Cdkn3 // cyclin-dependent kinase inhibitor 3 | Cdkn3 | 0.0305426 | 1.29692 |
| 14 | 10827292 | NM_021664 // Dnase2b // deoxyribonuclease II beta | Dnase2b | 0.0471796 | 1.29657 |
| 15 | 10914607 | NM_001102587 // Cxcr6 // chemokine receptor CXCR6 | Cxcr6 | 0.0159315 | 1.29411 |
| 16 | 10786755 | NM_031339 // Parg // poly (ADP-ribose) glycohydrolase | Parg | 0.00575851 | 1.29349 |
| 17 | 10771998 | ENSRNOT00000065079 // RGD1559459 // similar to Expressed sequence AI788959 | RGD1559459 | 0.0149153 | 1.27953 |
| 18 | 10849275 | NM_031664 // Slc28a2 // solute carrier family 28 | Slc28a2 | 0.0298301 | 1.27687 |
| 19 | 10886890 | Rattus norvegicus TL0AAA50YE17 mRNA sequence | Bsr IncRNA | 0.0035248 | 1.27574 |
| 20 | 10847111 | NM_001001059 // Olr630 // olfactory receptor 630 | Olr630 | 0.0193393 | 1.2747 |
| 21 | 10811768 | NM_001108455 // Fanca // Fanconi anemia, complementation group A | Fanca | 0.00145346 | 1.26576 |
| 22 | 10819946 | ENSRNOT00000012667 // Depdc1 // DEP domain containing 1 | Depdc1 | 0.033205 | 1.26449 |
| 23 | 10838741 | ENSRNOT00000067081 // Casc5 // cancer susceptibility candidate 5 | Casc5 | 0.0366092 | 1.26122 |
| 24 | 10772189 | NM_001008870 // Spink2 // serine peptidase inhibitor, Kazal type 2 | Spink2 | 0.04781 | 1.25781 |
| 25 | 10764551 | NM_017232 // Ptgs2 // prostaglandin-endoperoxide synthase 2 | Ptgs2 | 0.0366884 | 1.25669 |
| 26 | 10746976 | NM_022183 // Top2a // topoisomerase (DNA) II alpha | Top2a | 0.0310942 | 1.25042 |
| 27 | 10729444 | NM_001012743 // Pip5k1b // phosphatidylinositol-4-phosphate 5-kinase, type I, beta | Pip5k1b | 0.0130579 | 1.24117 |
| 28 | 10867714 | NM_001024900 // Tmem55a // transmembrane protein 55A | Tmem55a | 0.0473773 | 1.23712 |
| 29 | 10901771 | NM_001108081 // Actr6 // ARP6 actin-related protein 6 homolog (yeast) | Actr6 | 0.0269632 | 1.2352 |
| 30 | 10708591 | BC168993 // Sytl2 // synaptotagmin-like 2 | Sytl2 | 0.0390971 | 1.23024 |
| 31 | 10814430 | NM_012532 // Cp // ceruloplasmin | Cp | 0.0190683 | 1.22965 |
| 32 | 10779790 | NM_001000091 // Olr1627 // olfactory receptor 1627 | Olr1627 | 0.0368724 | 1.22656 |
| 33 | 10840910 | NM_001107790 // Tpx2 // TPX2, microtubule-associated, homolog (Xenopus laevis) | Tpx2 | 0.0195738 | 1.21079 |
| 34 | 10819005 | NM_134383 // Elovl6 // ELOVL family member 6, elongation of long chain fatty acid | Elovl6 | 0.0409503 | 1.20805 |
| 35 | 10930790 | NM_001106873 // Slc25a23 // solute carrier family 25 | Slc25a23 | 0.00530795 | 1.20459 |
| 36 | 10840102 | PREDICTED: Rattus norvegicus hypothetical protein LOC681292 | LOC681292 | 0.0143568 | 1.20382 |
| 37 | 10872850 | Rattus norvegicus TL0AEA86YB08 mRNA sequence |  | 0.0327168 | 1.2022 |
| 38 | 10864371 | ENSRNOT00000010717 // Frmd4b // FERM domain containing 4B | Frmd4b | 0.0105114 | 1.20164 |
| 39 | 10794320 | [ENSRNOT00000020914/  LOC679342](https://www.affymetrix.com/analysis/netaffx/exon/rna.affx?pk=135120963) | LOC679342 | 0.042328 | -1.20355 |
| 40 | 10772699 | BC100271 // RGD1311122 // similar to RIKEN cDNA 1110003E01 | RGD1311122 | 0.0129155 | -1.20443 |
| 41 | 10847213 | NM_001000625 // Olr711 // olfactory receptor 711 | Olr711 | 0.00733759 | -1.20512 |
| 42 | 10753959 | NM_213630 // Btla // B and T lymphocyte associated | Btla | 0.0381906 | -1.206 |
| 43 | 10787505 | NM_138875 // Jund // jun D proto-oncogene | Jund | 0.0351352 | -1.20765 |
| 44 | 10790670 | NM_001007684 // Klf2 // Kruppel-like factor 2 (lung) | Klf2 | 0.0229003 | -1.20799 |
| 45 | 10755544 | NM_053932 // B3gnt5 // UDP-GlcNAc:betaGal beta-1,3-N-acetylglucosaminyltransfera | B3gnt5 | 0.0172706 | -1.20823 |
| 46 | 10936211 | ENSRNOT00000009755 // Thoc2 // THO complex 2 | Thoc2 | 0.0160656 | -1.2088 |
| 47 | 10938824 | NR_031947 // Mir421 // microRNA mir-421 | Mir421 | 0.0424093 | -1.21669 |
| 48 | 10809909 | ENSRNOT00000050888 // LOC685424 // hypothetical protein LOC685424 | LOC685424 | 0.0207366 | -1.22232 |
| 49 | 10756147 | NM_001108849 // Clec4m // C-type lectin domain family 4, member M | Clec4m | 0.0270391 | -1.23214 |
| 50 | 10730659 | [GENSCAN00000037730](https://www.affymetrix.com/analysis/netaffx/exon/rna.affx?pk=135112607) |  | 0.0313393 | -1.24201 |
| 51 | 10721388 | NM_001024286 // Spib // Spi-B transcription factor (Spi-1/PU.1 related) | Spib | 0.042053 | -1.24949 |
| 52 | 10718766 | NM_001169149 // Nlrp9 // NLR family, pyrin domain containing 9 | Nlrp9 | 0.0037744 | -1.25069 |
| 53 | 10797811 | NM_001108410 // Cd83 // CD83 molecule | Cd83 | 0.045385 | -1.2515 |
| 54 | 10809899 | ENSRNOT00000044801 // RGD1566322 // similar to Serine/threonine protein phosphatase | RGD1566322 | 0.0135153 | -1.25892 |
| 55 | 10813949 | ENSRNOT00000014423 // Fam134b // family with sequence similarity 134, member B | Fam134b | 0.0100942 | -1.26536 |
| 56 | 10787334 | NM_001100908 // Fam129c // family with sequence similarity 129, member C | Fam129c | 0.0248402 | -1.27166 |
| 57 | 10765038 | Rattus norvegicus strain BN/SsNHsdMCW chromosome 13 | [Gas5 Inc RNA](http://www.ncbi.nlm.nih.gov/entrez/query.fcgi?db=gene&cmd=search&term=Gas5) | 0.00208016 | -1.27172 |
| 58 | 10925757 | ENSRNOT00000046179 // RGD1559955 // similar to 40S ribosomal protein S17 | RGD1559955 | 0.0270363 | -1.28091 |
| 59 | 10930618 | ENSRNOT00000051268 // ND6 // NADH dehydrogenase subunit 6 | ND6 | 0.0142515 | -1.2868 |
| 60 | 10866199 | NM_152848 // Ly49i2 // Ly49 inhibitory receptor 2 | Ly49i2 | 0.0311321 | -1.32447 |
| 61 | 10878112 | NM_021835 // Jun // Jun oncogene | Jun | 0.0391387 | -1.32633 |
| 62 | 10752259 | XM_003751094 // immunoglobulin lambda-like polypeptide 5-like | [LOC680329](http://www.ncbi.nlm.nih.gov/entrez/query.fcgi?db=gene&cmd=search&term=LOC680329) | 0.0180948 | -1.33034 |
| 63 | 10892521 | ENSRNOT00000044584 // RGD1564284 // similar to immunoglobulin 4G6 heavy chain variant | RGD1564284 | 0.0209071 | -1.3515 |
| 64 | 10756161 | NM_001105904 // Cd209a // CD209a molecule | Cd209a | 0.0334574 | -1.35529 |
| 65 | 10930775 | J03786 // Cyp2c12 // cytochrome P450, family 2, subfamily c, polypeptide 12 | Cyp2c12 | 0.00492151 | -1.35608 |
| 66 | 10816901 | NM_001191900 // Nup210l // nucleoporin 210-like | Nup210l | 0.0356503 | -1.42334 |
| 67 | 10930616 | ATP synthase F0 subunit 6, Mitochondrion | ATP6 | 0.00333629 | -1.42627 |
| 68 | 10765040 | Rattus norvegicus TL0AEA5YO19 mRNA sequence | [Gas5 Inc RNA](http://www.ncbi.nlm.nih.gov/entrez/query.fcgi?db=gene&cmd=search&term=Gas5) | 0.00140857 | -1.42983 |
| 69 | 10867318 | [Rattus norvegicus small nucleolar RNA U87, complete sequence](http://blast.ncbi.nlm.nih.gov/Blast.cgi#alnHdr_8927541) | [SNHG6 IncRNA](https://www.affymetrix.com/analysis/netaffx/exon/rna.affx?pk=129136427) | 0.0380562 | -1.47944 |
| 70 | 10866236 | NM_198746 // Klra5 // killer cell lectin-like receptor, subfamily A, member 5 | Klra5 | 0.0079603 | -1.56507 |
| 71 | 10861213 | NM_001015026 // Tspan12 // tetraspanin 12 | Tspan12 | 0.0111336 | -2.97188 |
| No assignment transcripts ID | | | | | |
| 72 | 10720272 | --- | --- | 0.0225735 | 1.56509 |
| 73 | 10797937 | --- | --- | 0.00462912 | 1.40093 |
| 74 | 10801781 | --- | --- | 0.0392634 | -1.20198 |
| 75 | 10802541 | --- | --- | 0.0158301 | -1.22227 |
| 76 | 10845605 | --- | --- | 0.0192866 | -1.24712 |
| 77 | 10859162 | --- | --- | 0.000873516 | -1.25227 |
| 78 | 10865347 | --- | --- | 0.0133308 | -1.33638 |
| 79 | 10940504 | --- | --- | 0.0167031 | -1.50091 |
| ESTs | | | | | |
| 80 | 10893338 | ENSRNOT00000004531 | --- | 0.0309853 | 1.40634 |
| 81 | 10811706 | [ENSRNOT00000031737](https://www.affymetrix.com/analysis/netaffx/exon/rna.affx?pk=135153544) | --- | 0.0306189 | 1.36217 |
| 82 | 10784892 | [ENSRNOT00000041154](https://www.affymetrix.com/analysis/netaffx/exon/rna.affx?pk=135128832) | --- | 0.0465285 | 1.28844 |
| 83 | 10839417 | [ENSRNOT00000042949](https://www.affymetrix.com/analysis/netaffx/exon/rna.affx?pk=135151931) | --- | 0.0413465 | 1.28764 |
| 84 | 10848103 | [ENSRNOT00000044536](https://www.affymetrix.com/analysis/netaffx/exon/rna.affx?pk=135153549) | --- | 0.0420909 | 1.28457 |
| 85 | 10862978 | [ENSRNOT00000046293](https://www.affymetrix.com/analysis/netaffx/exon/rna.affx?pk=135125328) | --- | 0.0338941 | 1.26434 |
| 86 | 10857177 | [ENSRNOT00000047030](https://www.affymetrix.com/analysis/netaffx/exon/rna.affx?pk=135126328) | --- | 0.0206723 | 1.23617 |
| 87 | 10775798 | [ENSRNOT00000047214](https://www.affymetrix.com/analysis/netaffx/exon/rna.affx?pk=135139276) | --- | 0.0116439 | 1.21946 |
| 88 | 10793387 | [ENSRNOT00000047350](https://www.affymetrix.com/analysis/netaffx/exon/rna.affx?pk=135120033) | --- | 0.0266443 | 1.21364 |
| 89 | 10734287 | [ENSRNOT00000049189](https://www.affymetrix.com/analysis/netaffx/exon/rna.affx?pk=135135857) | --- | 0.00821713 | 1.21068 |
| 90 | 10806301 | [ENSRNOT00000050750](https://www.affymetrix.com/analysis/netaffx/exon/rna.affx?pk=135131645) | --- | 0.0311872 | 1.20987 |
| 91 | 10729267 | [ENSRNOT00000050860](https://www.affymetrix.com/analysis/netaffx/exon/rna.affx?pk=135154555) | --- | 0.0227623 | 1.20783 |
| 92 | 10802708 | [ENSRNOT00000052462](https://www.affymetrix.com/analysis/netaffx/exon/rna.affx?pk=135156593) | --- | 0.040746 | 1.20761 |
| 93 | 10834602 | [ENSRNOT00000052628](https://www.affymetrix.com/analysis/netaffx/exon/rna.affx?pk=135157960) | --- | 0.0211564 | -1.20121 |
| 94 | 10744981 | [ENSRNOT00000052867](https://www.affymetrix.com/analysis/netaffx/exon/rna.affx?pk=135158640) | --- | 0.011665 | -1.20686 |
| 95 | 10929592 | ENSRNOT00000052932 | --- | 0.00619164 | -1.21747 |
| 96 | 10801135 | [ENSRNOT00000052964](https://www.affymetrix.com/analysis/netaffx/exon/rna.affx?pk=135155833) | --- | 0.0440974 | -1.21894 |
| 97 | 10919224 | ENSRNOT00000053015 | --- | 0.0459562 | -1.22613 |
| 98 | 10721698 | [ENSRNOT00000053402](https://www.affymetrix.com/analysis/netaffx/exon/rna.affx?pk=135157570) | --- | 0.0354724 | -1.23346 |
| 99 | 10820217 | [ENSRNOT00000053470](https://www.affymetrix.com/analysis/netaffx/exon/rna.affx?pk=135159122) | --- | 0.00939193 | -1.23501 |
| 100 | 10847488 | [ENSRNOT00000053571](https://www.affymetrix.com/analysis/netaffx/exon/rna.affx?pk=135156821) | --- | 0.0104015 | -1.23632 |
| 101 | 10936319 | ENSRNOT00000053859 | --- | 0.0256128 | -1.23818 |
| 102 | 10743606 | [ENSRNOT00000054079](https://www.affymetrix.com/analysis/netaffx/exon/rna.affx?pk=135158256) | --- | 0.00631291 | -1.23862 |
| 103 | 10713602 | [ENSRNOT00000054147](https://www.affymetrix.com/analysis/netaffx/exon/rna.affx?pk=135157775) | --- | 0.00427095 | -1.23889 |
| 104 | 10721696 | [ENSRNOT00000054185](https://www.affymetrix.com/analysis/netaffx/exon/rna.affx?pk=135157275) | --- | 0.0466009 | -1.24614 |
| 105 | 10891392 | ENSRNOT00000054609 | --- | 0.00236988 | -1.25236 |
| 106 | 10936019 | ENSRNOT00000056343 | --- | 0.00877702 | -1.25888 |
| 107 | 10902413 | [ENSRNOT00000058469](https://www.affymetrix.com/analysis/netaffx/exon/rna.affx?pk=135123725) | --- | 0.00499035 | -1.25906 |
| 108 | 10901407 | ENSRNOT00000059637 | --- | 0.000369468 | -1.26976 |
| 109 | 10801256 | [ENSRNOT00000060470](https://www.affymetrix.com/analysis/netaffx/exon/rna.affx?pk=135121440) | --- | 0.0242722 | -1.27361 |
| 110 | 10867371 | [ENSRNOT00000061902](https://www.affymetrix.com/analysis/netaffx/exon/rna.affx?pk=135127896) | --- | 0.040248 | -1.27713 |
| 111 | 10852054 | [ENSRNOT00000063671](https://www.affymetrix.com/analysis/netaffx/exon/rna.affx?pk=135159217) | --- | 0.00047967 | -1.27737 |
| 112 | 10909843 | ENSRNOT00000063731 | --- | 0.00195872 | -1.32872 |
| 113 | 10920741 | ENSRNOT00000069418 | --- | 0.0173812 | -1.34254 |
| 114 | 10906451 | [ENSRNOT00000070022](https://www.affymetrix.com/analysis/netaffx/exon/rna.affx?pk=135158222) | --- | 0.0343232 | -1.36248 |
| 115 | 10751209 | ENSRNOT00000070029 | --- | 0.022505 | -1.36888 |
| 116 | 10805227 | [ENSRNOT00000070327](https://www.affymetrix.com/analysis/netaffx/exon/rna.affx?pk=135157525) | --- | 0.00131982 | -1.37127 |
