# Supplementary material for: Transcriptional profiling of left ventricle and peripheral blood mononuclear cells in a rat model of postinfarction heart failure
Source: BMC Med Genomics. 2013 Nov 8;6:49. doi: 10.1186/1755-8794-6-49 (PMC4226214; doi:10.1186/1755-8794-6-49)
Supplement: Additional file 6 — Chart report from DAVID analysis to determine GO term enrichment of BP (biological process), MF (molecular function) and CC (cellular component) and their associated genes differentially expressed in PBMCs between L-MI and sham operated group. [file 1755-8794-6-49-S6.doc]

**Additional file 6**: Chart report from DAVID analysis to determine GO term enrichment of BP (biological process), MF (molecular function) and CC (cellular component) and their associated genes differentially expressed in PBMCs between L-MI and sham operated group

| Category | Term | Count | % | p-value | FDR | Gene Name | Gene Symbol |
| --- | --- | --- | --- | --- | --- | --- | --- |
| GOTERM_MF_ALL | GO:0005529~sugar binding | 6 | 8,82 | 5e-4 | 0,64 | [CD209a molecule, CD209d antigen, killer cell lectin-like receptor family I member 2, killer cell lectin-like receptor subfamily C, member 2; killer cell lectin-like receptor subfamily C, member 3, olfactory receptor 1627, similar to osteoclast inhibitory lectin](http://david.abcc.ncifcrf.gov/geneReportFull.jsp?rowids=412729) | Cd209a,  Clec4m,  Klri2, Klrc2, Klrc3,  Olr1627, Klra7 |
| GOTERM_MF_ALL | GO:0030246~carbohydrate binding | 6 | 8,82 | 3e-3 | 4,59 | [CD209a molecule, CD209d antigen, killer cell lectin-like receptor family I member 2, killer cell lectin-like receptor subfamily C, member 2; killer cell lectin-like receptor subfamily C, member 3, olfactory receptor 1627, similar to osteoclast inhibitory lectin](http://david.abcc.ncifcrf.gov/geneReportFull.jsp?rowids=412729) | Cd209a,  Clec4m,  Klri2, Klrc2, Klrc3,  Olr1627, Klra7 |
| GOTERM_BP_ALL | GO:0007568~aging | 4 | 5,88 | 0,01 | 8,74 | Jun D proto-oncogene, Jun oncogene, ceruloplasmin, telomerase RNA component | Jund, Jun, Cp, Terc |
| GOTERM_BP_ALL | GO:0014070~response to organic cyclic substance | 4 | 5,88 | 0,02 | 20,70 | CD83 molecule, Jun D proto-oncogene, Jun oncogene, prostaglandin-endoperoxide synthase 2 | CD38, Jund, Jun, Ptgs2 |
| GOTERM_BP_ALL | GO:0009617~response to bacterium | 4 | 5,88 | 0,02 | 20,94 | CD83 molecule, Jun D proto-oncogene, Jun oncogene, prostaglandin-endoperoxide synthase 3 | CD38, Jund, Jun, Ptgs2 |
| GOTERM_BP_ALL | GO:0051707~response to other organism | 4 | 5,88 | 0,03 | 32,62 | Jun D proto-oncogene, Jun oncogene, poly (ADP-ribose) glycohydrolase, prostaglandin-endoperoxide synthase 2 | Jund, Jun, Parg, Ptgs2 |
| GOTERM_BP_ALL | GO:0032496~response to lipopolysaccharide | 3 | 4,41 | 0,04 | 40,44 | Jun D proto-oncogene, Jun oncogene, prostaglandin-endoperoxide synthase 3 | Jund, Jun, Ptgs2 |
| GOTERM_BP_ALL | GO:0002237~response to molecule of bacterial origin | 3 | 4,41 | 0,04 | 45,78 | Jun D proto-oncogene, Jun oncogene, prostaglandin-endoperoxide synthase 4 | Jund, Jun, Pts2 |
| GOTERM_CC_ALL | GO:0016020~membrane | 25 | 36,76 | 0,04 | 39,22 | ATP synthase protein 8; B and T lymphocyte associated; CD83 molecule, ELOVL family member 6; elongation of long chain fatty acids (yeast); ND6; UDP-GlcNAc:betaGal beta-1,3-N-acetylglucosaminyltransferase 5; ceruloplasmin; chemokine receptor CXCR6; cytochrome P450, family 2, subfamily c, polypeptide 12; family with sequence similarity 134, member B; zinc finger protein 622; killer cell lectin-like receptor family I member 2; killer cell lectin-like receptor subfamily C, member 2; killer cell lectin-like receptor subfamily C, member 3; lectin, galactoside-binding, soluble, 3 binding protein; olfactory receptor 1627; olfactory receptor 1654; olfactory receptor 630; olfactory receptor 711; phosphatidylinositol-4-phosphate 5-kinase, type I, beta; prostaglandin-endoperoxide synthase 2; similar to RIKEN cDNA 1110003E01; similar to osteoclast inhibitory lectin; similar to solute carrier family 28, member 2; solute carrier family 28 (sodium-coupled nucleoside transporter), member 2; solute carrier family 25 (mitochondrial carrier; phosphate carrier), member 23; tetraspanin 12; transmembrane protein 55A | ATP6, Btla, CD83, Elovl6, ND6, B3gnt5, Cp, Cxcr6, Cyp2c12, Fam134b, Klri2, Klrc2, Klrc3, Lgals3b, Olr1654, Olr154, Olr630, Olr1711, Pip5k1b, Ptgs2, RGD1311122, Klra7, Slc28a2, Slc25a2, Tspan12, Tmem55a |
| GOTERM_BP_ALL | GO:0009607~response to biotic stimulus | 4 | 5,88 | 0,04 | 48,59 | Jun D proto-oncogene, Jun oncogene, poly (ADP-ribose) glycohydrolase, prostaglandin-endoperoxide synthase 2 | Jund, Jun, Parg, Ptgs2 |
| GOTERM_MF_ALL | GO:0005537~mannose binding | 2 | 2,94 | 0,05 | 45,55 | CD209a molecule, CD209d antigen | Cd209a,  Clec4m |
