# Supplementary material for: Transcriptional profiling of left ventricle and peripheral blood mononuclear cells in a rat model of postinfarction heart failure
Source: BMC Med Genomics. 2013 Nov 8;6:49. doi: 10.1186/1755-8794-6-49 (PMC4226214; doi:10.1186/1755-8794-6-49)
Supplement: Additional file 7 — Genes that were altered similarly in LVs and PBMCs - microarray results. [file 1755-8794-6-49-S7.doc]

**Additional file 7:** Genes that were altered similarly in LVs and PBMCs – microarray results

| Transcript Cluster  ID | gene_assignment | Gene Symbol | p-value  (L-MI vs Sham in PBMCs) | Fold-Change  (L-MI vs Sham in PBMCs) | p-value  (L-MI vs Sham i LVs) | Fold-Change  (L-MI vs Sham in LVs) | p-value  (M-MI vs Sham in LVs) | Fold-Change  (M-MI vs Sham in LVs) | p-value  (S-MI vs Sham in LVs) | Fold-Change  (S-MI vs Sham in LVs) |
| --- | --- | --- | --- | --- | --- | --- | --- | --- | --- | --- |
| 10764551 | NM_017232 // Ptgs2 // prostaglandin-endoperoxide synthase 2 | Ptgs2 | 0.036689 | 1.25669 | 9.90e-006 | 2.16193 | 0.47587 | 1.09037 | 0.750453 | 1.0391 |
| 10814430 | NM_012532 // Cp // ceruloplasmin | Cp | 0.019069 | 1.22965 | 1.85e-009 | 2.28215 | 0.611234 | 1.03597 | 0.277342 | 1.07959 |
| 10859090 | EU128749.1// LOC689800 // C-type lectin-related protein 7 | LOC689800/  Clr7 | 0.013527 | 1.56126 | 2.41e-006 | 1.73519 | 0.067906 | 1.15968 | 0.001359 | 1.33715 |
| 10861213 | NM_001015026 // Tspan12 // tetraspanin 12 | Tspan12 | 0.011134 | -2.97188 | 7.98e-006 | -1.43463 | 0.069434 | -1.11207 | 0.059028 | -1.1173 |
| 10866144 | XM_002726462 // LOC100364751 // killer cell lectin-like receptor, subfamily A, member 7 | LOC100364751/  Klra7 | 0.048494 | 1.37806 | 0.000328 | 1.30594 | 0.001825 | 1.23296 | 5.08e-005 | 1.35661 |
