# Supplementary material for: Transcriptional profiling of left ventricle and peripheral blood mononuclear cells in a rat model of postinfarction heart failure
Source: BMC Med Genomics. 2013 Nov 8;6:49. doi: 10.1186/1755-8794-6-49 (PMC4226214; doi:10.1186/1755-8794-6-49)
Supplement: Additional file 8 — Comparison of results from RT-qPCR of genes that were altered similarly in LVs and PBMCs. [file 1755-8794-6-49-S8.doc]

**Additional file 8:** Comparison of results from RT-qPCR of genes that were altered similarly in LVs and PBMCs

|  | | **Results from RT- qPCR** | | | | | | | | | | | | | | | | | | |
| --- | --- | --- | --- | --- | --- | --- | --- | --- | --- | --- | --- | --- | --- | --- | --- | --- | --- | --- | --- | --- |
|  | | **PBMCs** | | | | | | | | | **LVs** | | | | | | | | | |
|  |  | **Large-MI vs Sham** | | | **Moderate-MI vs Sham** | | | **Small-MI vs Sham** | | | **Large-MI vs Sham** | | | **Moderate-MI vs Sham** | | | | **Small-MI vs Sham** | | |
| **Gene symbol** | **GenBank ID** | **Fold Change** | ***P*-value** | **Signifi-cance** | **Fold Change** | ***P*-value** | **Signifi-cance** | **Fold Change** | ***P*-value** | **Signifi-cance** | **Fold Change** | ***P*-value** | **Signifi-cance** | **Fold Change** | ***P*-value** | **Signifi-cance** | **Fold Change** | | ***P*-value** | **Signifi-cance** |
| **Clr7** | **EU128749.1** | **1.4** | 0.129 | ns | **1.2** | 0.637 | ns | **1.4** | 0.339 | ns | **1.5** | 0.046 | * | **1.3** | 0.018 | * | **1.3** | | 0.113 | ns |
| **Cp** | **NM_012532** | **2.3** | 0.039 | * | **-1.1** | 0.801 | ns | **1.5** | 0.415 | ns | **3.3** | 0.001 | ** | **1.1** | 0.483 | ns | **1.1** | | 0.463 | ns |
| **Klra7** | **XM_578407.4** | **1.6** | 0.112 | ns | **1.1** | 0.610 | ns | **1.9** | 0.055 | near | **2.3** | 0.001 | ** | **1.6** | 0.022 | * | **2** | | 0.003 | ** |
| **Ptgs2** | **NM_017232** | **2.4** | 0.073 | near | **-1.1** | 0.843 | ns | **2.1** | 0.214 | ns | **3.5** | 0.001 | ** | **1** | 0.816 | ns | **1** | | 0.658 | ns |
| **Tspan12** | **NM_001015026** | **-3.22** | 0.019 | * | **1** | 0.957 | ns | **-1.4** | 0.544 | ns | **1.1** | 0.734 | ns | **-1.1** | 0.665 | ns | **-1.1** | | 0.950 | ns |

Statistical significance: **P*<0.05, ***P*<0.01, ns = non significant, near = near significance. Results were normalized to *Gapdh* and *Hprt*.
